# Total Synthesis of the Norcembranoid Scabrolide B and Its Transformation into Sinuscalide C, Ineleganolide, and Horiolide

**DOI:** 10.1021/jacs.4c09467

**Published:** 2024-08-21

**Authors:** Davy S. Lin, Georg Späth, Zhanchao Meng, Lianne H. E. Wieske, Christophe Farès, Alois Fürstner

**Affiliations:** Max-Planck-Institut für Kohlenforschung, 45470 Mülheim/Ruhr, Germany

## Abstract

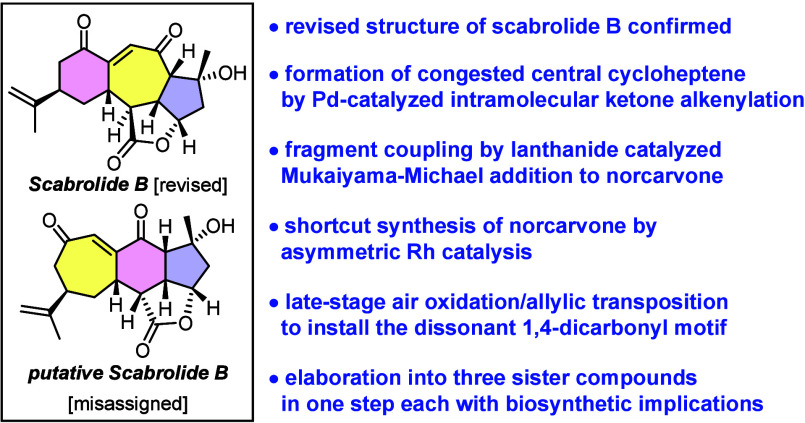

It was recognized only recently that the sister norcembranoids scabrolides A and B have notably different carbotricyclic scaffolds. Therefore, our synthesis route leading to scabrolide A could not be extended to its sibling. Rather, a conceptually new approach had to be devised that relied on a challenging intramolecular alkenylation of a ketone to forge the congested central cycloheptene ring at the bridgehead enone site; the required cyclization precursor was attained by a lanthanide-catalyzed Mukaiyama–Michael addition. The dissonant 1,4-oxygenation pattern was then installed by allylic rearrangement/oxidation of the enone, followed by suprafacial 1,3-transposition. Synthetic scabrolide B was transformed into sinuscalide C by dehydration and into ineleganolide by base-mediated isomerization/oxa-Michael addition, which has potential biosynthetic implications; under basic conditions, the latter compound converts into horiolide by an intricate biomimetic cascade.

The final stages of our total synthesis of scabrolide A (**1**),^1−5^ an intriguing norcembranoid derived from soft corals of the genus *Sinularia*,^6^ capitalized on earlier biosynthetic considerations which had suggested that **1** derives from a sister compound **2** named “scabrolide B” by double-bond isomerization (Figure 1).^7−10^ While this transformation was indeed achieved in essentially quantitative yield, we noticed a perplexing incongruence:^1^ synthetic **1** corresponded perfectly to scabrolide A, but its precursor **2** did not match presumed scabrolide B at all. The proposed biosynthesis of **1** might hence be correct, but authentic scabrolide B is not on the pathway; its structure had been misassigned in the original isolation paper.^6,11^

**Figure 1:**
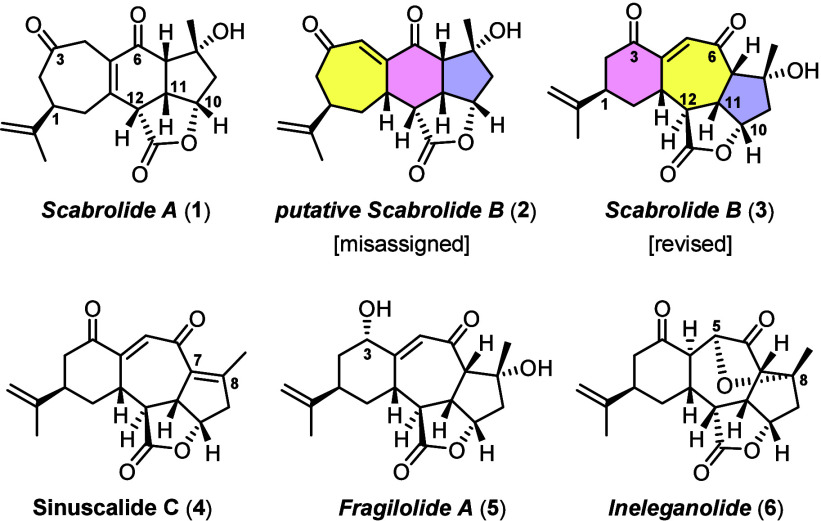
Selected polycyclic norcembranoid diterpenoids from *Sinularia* and related soft corals.

The available data did not allow us to firmly revise the structure of scabrolide B. Therefore, we resorted to an in silico screening, in which the spectra of all possible stereomers of types **2** and **3** were computed at the DFT level and compared to the experimental data set of scabrolide B; the match/mismatch was assessed using the DP4+ probabilistic tool.^12−14^ Since the validity of this approach could be convincingly demonstrated,^15^ the excellent score for isomer **3** encouraged us to embark on a new total synthesis project to confirm the reassignment.^16^ This goal, however, became obsolete soon thereafter when scabrolide B was reisolated and its structure established by X-ray diffraction analysis.^17^ Actually, it seems that the compound was independently obtained a second time but published under the name “sinuscalide D”, the data of which perfectly match those of scabrolide B.^18^ Suffice it to say that these reisolation campaigns confirmed our computational prediction.

Natural scabrolide B (**3**) differs significantly from scabrolide A (**1**) in that it features a 6–7–5 rather than 7–6–5 carbotricyclic skeleton; as such, it is closely related to sinuscalide C (**4**) as its dehydrated sibling^18^ as well as to fragilolide A (**5**),^19^ in which the C3 ketone is reduced. In addition to this constitutional disparity, it is noteworthy that the C12 stereocenters of **1** and **3** are of opposite configuration. This apparent subtlety has significant (bio)synthetic implications (see below); it also sets scabrolide B (**3**) apart from ineleganolide (**6**),^20^ which otherwise has the same 6–7–5 core structure spanned by an additional tetrahydrofuran ring that could derive from a transannular oxa-Michael addition of the C8–OH group onto C5 of the enone subunit, although the proposed biosynthesis suggests otherwise.^21^ The remarkable topological complexity and dense functionalization rendered ineleganolide an iconic target within this intriguing family of polycyclic norcembranoides; although it had captured attention of the synthetic community for decades, successful conquests of **6** and its equally demanding relatives were reported only lately.^22−26^ In light of the new results summarized below, it is relevant to note that control over the C12 stereocenter had thwarted one otherwise seminal approach toward this intricate target.^25^

Initially, we had hoped that some fairly straightforward adjustments of our successful route to scabrolide A (**1**) might also bring scabrolide B (**3**) into reach. Specifically, the central six-membered ring of **1** had been forged by ring-closing metathesis (RCM). The resulting alkene **8** was subjected to hydroxy-directed epoxidation followed by base-induced ring opening to set the dissonant 1,4-dioxygenation pattern (Scheme 1A); a few steps then sufficed to convert compound **10** into the target.^1^ We had to learn, however, that this strategy could not be extrapolated to scabrolide B (Scheme 1B):^12^ while diene **11** underwent ring closure without incident, all attempts at selective (hydroxy-directed) oxidation of one or the other double bond of the resulting 1,3-diene **12** met with failure. In stark contrast, the delicate β,γ-unsaturated ketone **13** could not be engaged in RCM even under forcing conditions; poor conversions into complex mixtures were observed, which contained no **14** but traces of an isomeric cycloheptene.^12,27^

**Scheme 1:**
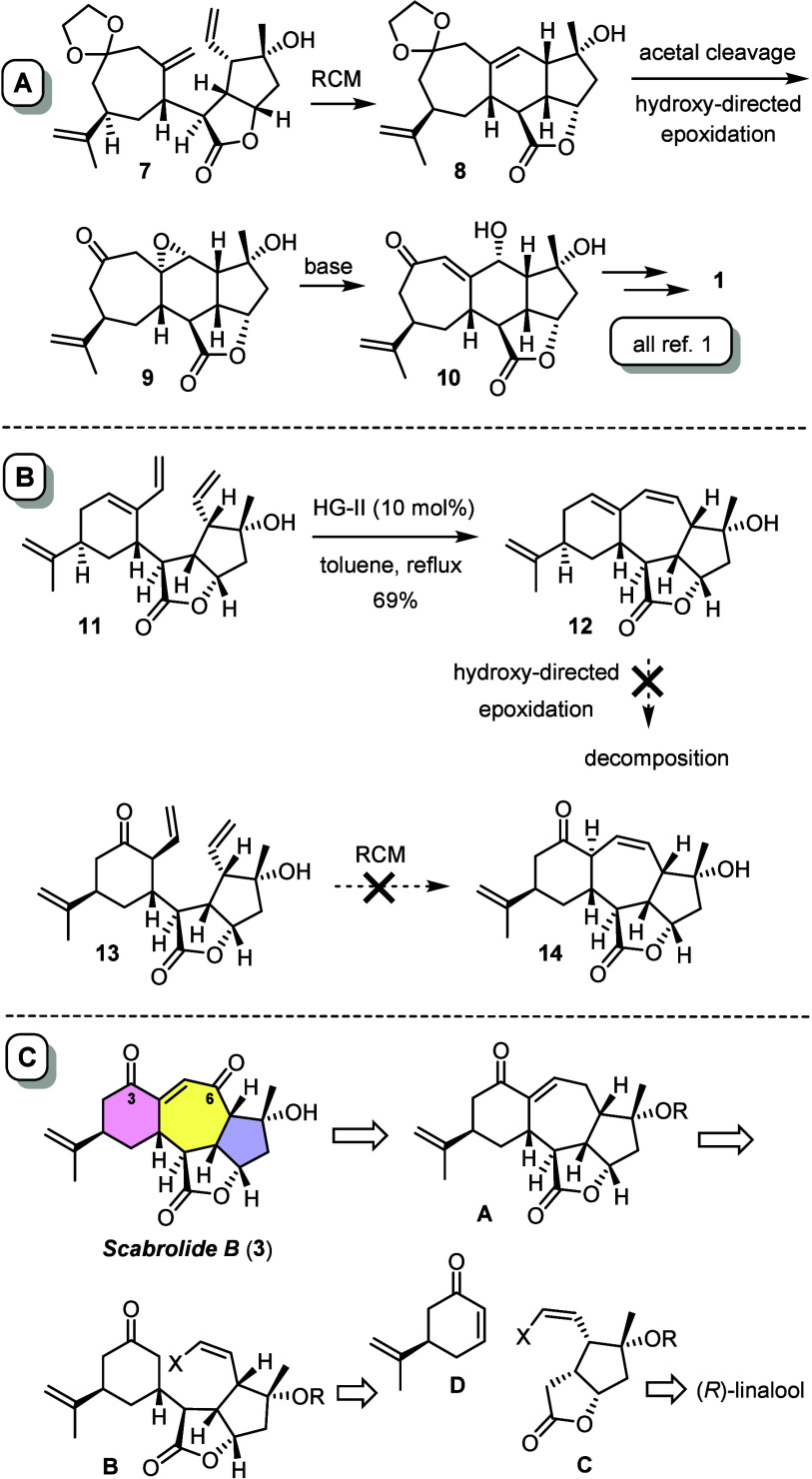
(A) Literature Precedent, (B) Intelligence Gathering, and (C) Retrosynthetic Analysis

Therefore, a substantial revision of the synthetic plan was mandatory (Scheme 1C). After careful consideration, we opted for enolate alkenylation as the way to form the central ring;^28^ ideally, it would come along with double-bond isomerization to furnish an enone of type **A**. This novel strategy based on the formation of the arguably challenging C4–C5 bond^29^ bore considerable risk: first, both C–H acidic sites flanking the C3 carbonyl group of **B** are equally well accessible. Hence, the reaction can work only if enolization is reversible; both enolates would form and be able to revert to **B**, but one of them can also cyclize; in doing so, the desired product **A** might accumulate in a meaningful yield. However, the failed attempts at making the closely related compound **14** by RCM implied that **A** comprising a bridgehead alkene is almost certainly highly congested;^29^ while intramolecular enolate alkenylations, though not particularly widespread, have a good track record in closing five- and six-membered rings,^28,30,31^ applications to strained and/or hindered products are rare.^32−34^ If successful, however, only an allylic oxidation would be needed to convert a product of type **A** into **3**. Another argument in favor of the envisaged plan was the fact that the cyclization precursor **B** should be readily accessible by Michael addition of lactone **C** to enone **D**. Since we had previously developed a scalable route to terminal alkene **C** (X = H),^1^ the analogous alkenyl halide **C** (X = I, Br), as required in this project, seemed easy to attain in optically pure form.

(*R*)-Norcarvone (**19**) as the envisaged Michael acceptor is known in the literature, but the published synthesis takes at least seven steps;^35^ therefore, we were prompted to find a shortcut (Scheme 2). To this end, an asymmetric rhodium-catalyzed 1,4-addition of commercial boronate **16** to cyclohexenone (**15**) was adapted from the literature,^36,37^ which furnished **17** with excellent optical purity (94% ee) on gram scale.^38^ Subsequent deprotonation with bulky LiTMP followed by a TMSCl quench gave silyl enol ether **18** as the major isomer (rr ≥ 5:1). The subsequent Saegusa-type oxidation worked best with Pd_2_(dba)_3_ as the catalyst in the absence of any extra ligand and diallyl carbonate as the terminal oxidant.^39,40^ A short-path distillation allowed the resulting product to be separated from (coeluting) dba, thus securing good quantities of analytically pure **19**.

**Scheme 2:**
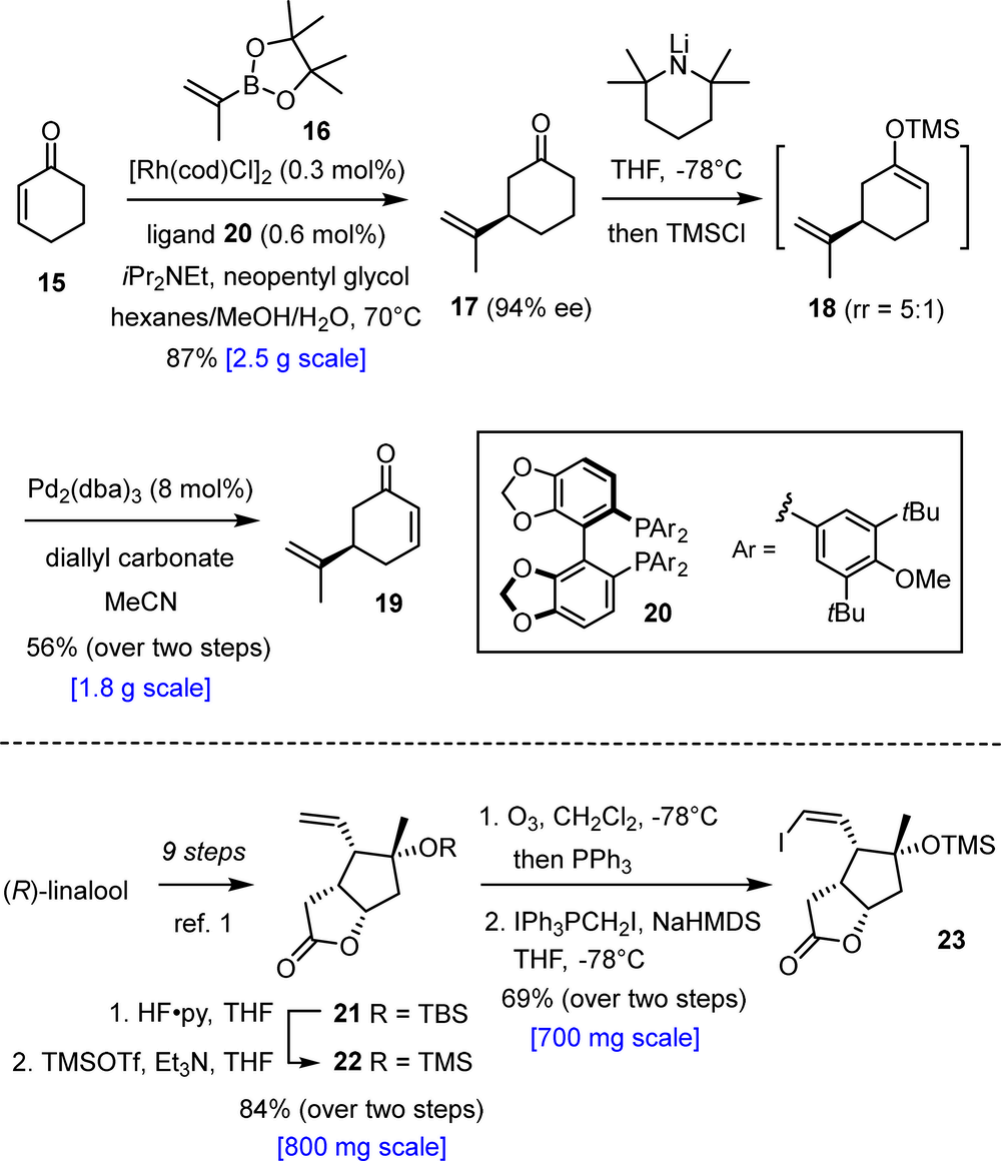
Preparation of the Building Blocks

Lactone **21** was prepared from (*R*)-linalool as previously described.^1,41^ Because we had to learn at a later stage of the project that the bulky silyl ether at the tertiary C8–OH position thwarted the envisaged end game but a protecting group was needed, the TBS group was swapped to a TMS ether prior to ozonolytic cleavage of the double bond in **22**.^42^ The resulting aldehyde was instantly subjected to Stork–Zhao olefination to give the required *Z*-configured alkenyl iodide **23** in good yield.^43,44^

At the stage of fragment coupling (Scheme 3), we were beneficiaries of earlier work that had shown that Mukaiyama–Michael addition reactions^45^ to carvone derivatives work well when catalyzed by lanthanum salts.^25,46^ In fact, the silyl ketene acetal generated in situ from **23** under soft enolization conditions reacted with **19** in the presence of La(OTf)_3_ to give fragile **24**, which was briefly exposed to TBAF at −78 °C to entail selective cleavage of the silyl enol ether without harming the labile −OTMS group; after some optimization,^47^ the desired product **25** was obtained in 70% yield. As one might expect, the conjugate addition proceeded via axial attack of the nucleophile that transiently formed onto the lowest-energy conformer of **19**. The critically important configuration of the newly formed stereocenters at the overcrowded C12–C13 bond was inferred from a set of characteristic NOEs and *J*_H,H_ coupling constants^12^ and confirmed by X-ray diffraction analysis (Figure 2).

**Scheme 3:**
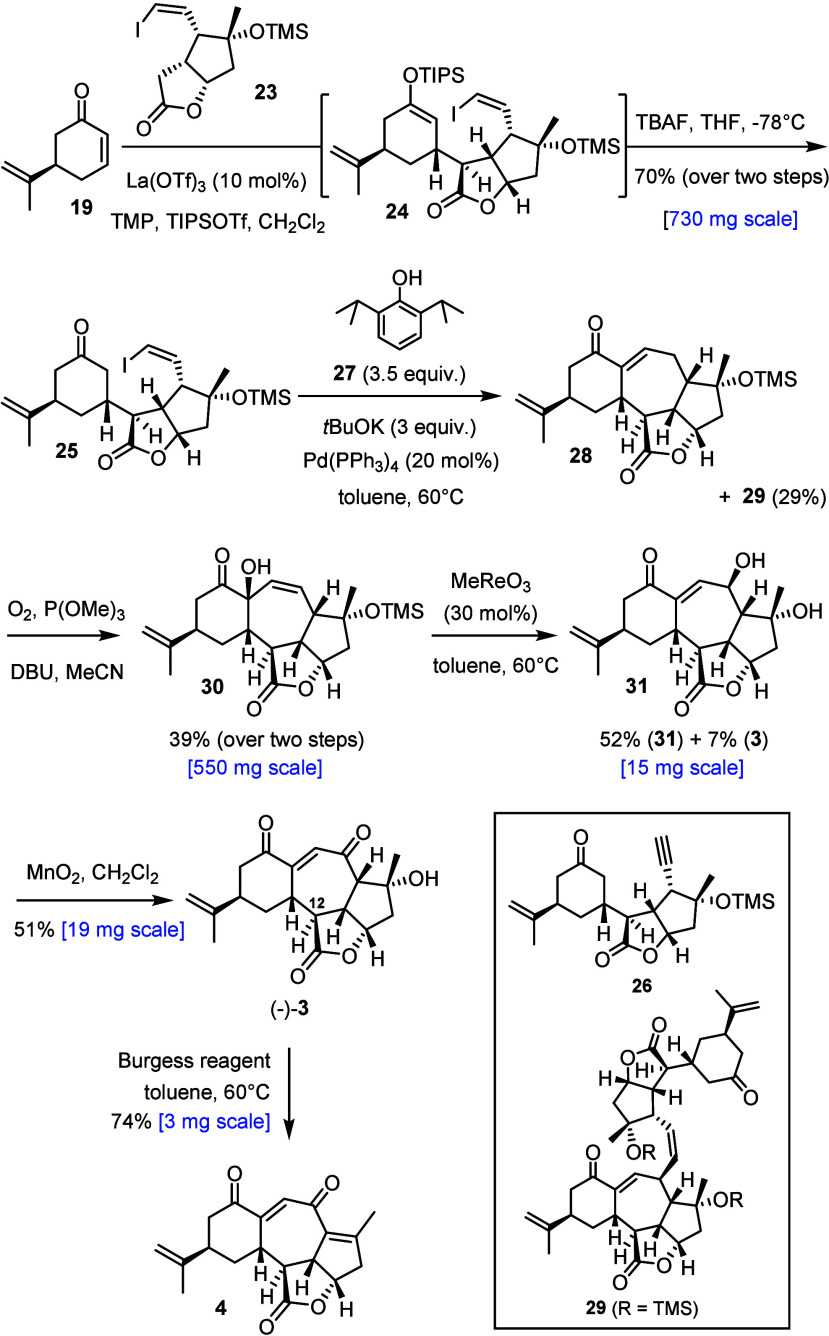
Completion of the Total Synthesis

**Figure 2:**
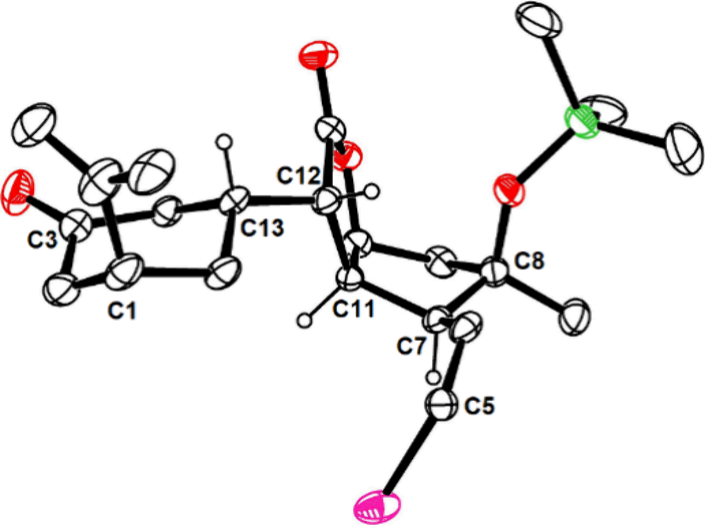
Structure of **25** in the solid state (scabrolide numbering scheme).^48^

As expected, the subsequent closure of the central seven-membered ring of scabrolide B (**3**) was challenging in the first place. Attempts at engaging silyl enol ether **24** directly into ring closure resulted in decomposition.^49^ When using the derived ketone **25**, the choice of base and solvent had to meet the boundary conditions outlined above; therefore, a number of common procedures for palladium-catalyzed enolate alkenylations were sorted out as nonviable in the present case. This included the use of *t*BuOK or TBAF,^28,31,50,51^ which caused dehydrohalogenation with formation of alkyne **26**; K_2_CO_3_ in MeOH (with or without Bu_4_NBr) also failed.^31b−31f^ A first hit was obtained with PhOH/*t*BuOK,^52,53^ although **28** was only one of several products formed in low yield (≤20%). However, this result was deemed encouraging. Upon careful optimization, it was found that sterically hindered 2,6-diisopropylphenol (**27**) (3.5 equiv) in combination with *t*BuOK (3 equiv) in toluene (2 mM) at 60 °C was an adequate promoter in combination with Pd(PPh_3_)_4_ as catalyst. Under these conditions, the congested tricyclic enone **28** was formed in respectable yield (∼60%) together with dimeric side product **29** (29%) formed by a second, now intermolecular alkenylation at the vinylogous C6 of **28**. Since separation required HPLC, it was best to engage crude **28** in allylic γ-oxidation. While several standard oxidants failed to effect this seemingly straightforward transformation, the method used by the Sarlah group in their total synthesis of scabrolide A (**1**) proved to be viable.^3,54^ Thus, stirring of a solution of crude **28** in MeCN under an O_2_ atmosphere in the presence of P(OMe)_3_ and DBU resulted in allylic rearrangement/oxidation with formation of **30** as a single diastereomer without affecting the olefin branching off the cyclohexane ring. For the then necessary oxidative transposition of the allylic alcohol into the desired 1,4-diketone, Sarlah and co-workers had used PCC,^3^ which failed in our case. Therefore we had to resort to a stepwise procedure, commencing with a suprafacial 1,3-allylic rearrangement of **30** into **31** catalyzed by MeReO_3_, which led to concomitant cleavage of the tertiary −OTMS ether (and also afforded a first small crop of **3**).^55,56^ Finally, **31** was oxidized with MnO_2_ to give the targeted compound (−)-**3**. The analytical and spectral data of the synthetic material were in full accord with those of authentic scabrolide B (“sinuscalide D”);^17,18^ an X-ray structure analysis excluded any doubt (Figure 3).

**Figure 3:**
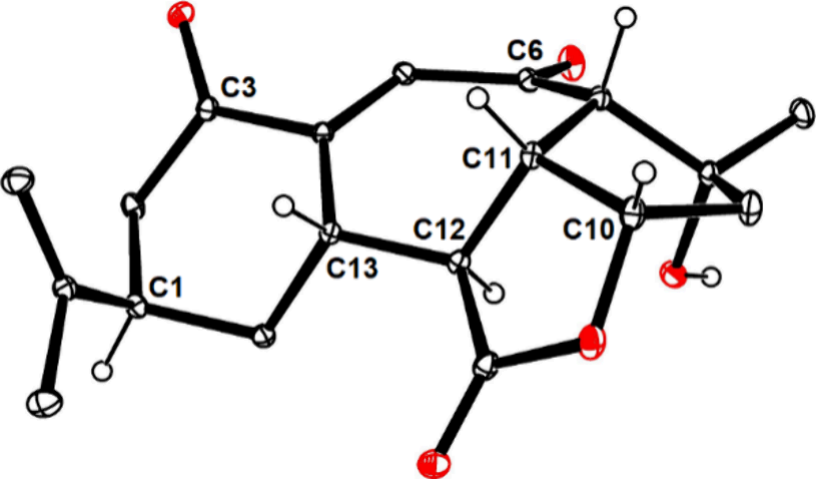
Structure of **3** in the solid state.

Treatment of **3** with Burgess reagent^57^ afforded sinuscalide C (**4**) in good yield, the data of which also nicely matched the literature.^18^ Finally, an attempt was made to transform synthetic **3** into ineleganolide (**6**) (Scheme 4). Although the proposed biosynthesis of **6** does not pass through scabrolide B,^21^ this foray was inspired by an observation previously made en route to scabrolide A (**1**) that certain compounds featuring a *cis*,*trans*-annelated butenolide ring could be epimerized to the corresponding *cis*,*cis* isomers under basic conditions.^1^ Indeed, stirring of a solution of **3** in Et_3_N/MeOH/MeCN at 60 °C triggered a cascade comprising an oxa-Michael addition of the C8–OH group onto the enone with formation of the signature tetrahydrofuran ring of ineleganolide (**6**) and epimerization of the C12 stereocenter; this observation has potential biosynthetic implications.^58^ Somewhat unfortunately, **6** turned out to be only metastable under the chosen conditions (see below); therefore, the reaction was stopped at incomplete conversion, and unreacted **3** was recovered. While the net yield of ineleganolide (**6**) per round was low,^59^ the recorded data matched nicely.^20,22^

**Scheme 4:**
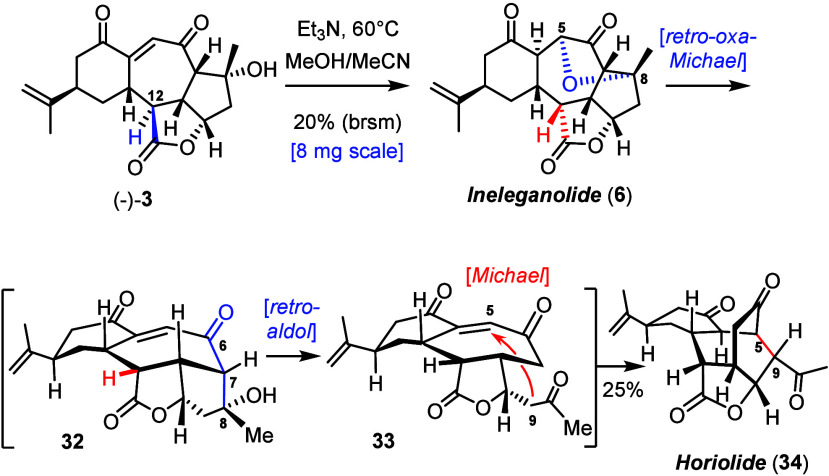
Syntheses of Ineleganolide and Horiolide

When the reaction was left stirring, a new product slowly emerged at the expense of **6**, which was identified as horiolide (**34**).^60^ Its formation implies that the ether ring of **6** can be cleaved in a retro-oxa-Michael fashion under the chosen conditions but the resulting compound **32** does not revert to **3** by epimerization of C12;^61^ rather, it adopts a conformation that allows the C7–C8 σ orbital to overlap with the C6–O π* orbital. The ensuing retro-aldol reaction affords **33**, which instantly succumbs to a proximity-driven intramolecular Michael addition to form the new C5–C9 bond.^62^ The involved course of this step mirrors the proposed biosynthetic pathway.^7^

In summary, we describe the first conquest of scabrolide B (**3**) in 19 steps (longest linear sequence) and its elaboration into sinuscalide C (**4**), ineleganolide (**6**), and horiolide (**34**). Key to success was a challenging intramolecular alkenylation of an almost symmetrical ketone, which allowed the congested seven-membered ring with the inscribed bridgehead olefin to be forged; in this embodiment, the reaction has arguably no precedent but obviously much potential. Of equal relevance is the fact that the successful conversion of scabrolide B into ineleganolide might emulate a previously unrecognized biogenetic link between these emblematic marine norcembranoids that merits further study.^21,58,61,63^

## Supporting Information Available

Experimental Section including characterization data and NMR spectra of new compounds as well as a crystallographic abstract (PDF)
