# Supplementary material for: Total Synthesis of the Norcembranoid Scabrolide B and Its Transformation into Sinuscalide C, Ineleganolide, and Horiolide
Source: J Am Chem Soc. 2024 Aug 21;146(35):24250–6. doi: 10.1021/jacs.4c09467 (PMC11378282; doi:10.1021/jacs.4c09467)
Supplement: Supplementary file 1 — ja4c09467_si_001.pdf [file ja4c09467_si_001.pdf]

SUPPORTING INFORMATION

Total Synthesis of the Norcembranoid Scabrolide B and its Transformation into Sinuscalide C, Ineleganolide and Horiolide

Davy S. Lin, Georg Späth, Zhanchao Meng, Lianne H. E. Wieske, Christophe Farès, and Alois Fürstner*

Max-Planck-Institut für Kohlenforschung, 45470 Mülheim/Ruhr, Germany

Email: fuerstner@kofo.mpg.de

Table of Contents

Crystallographic Information	S2
DP4+ Probability Analyses of Scabrolide B	S4
Intelligence Gathering (Schemes)	S12
General Information	S15
Building Blocks	S15
Intelligence Gathering (Procedures)	S19
Fragment Coupling	S22
Completion of the Total Synthesis	S23
Copies of NMR Spectra of New Compounds	S39
References	S94

Crystallographic Information

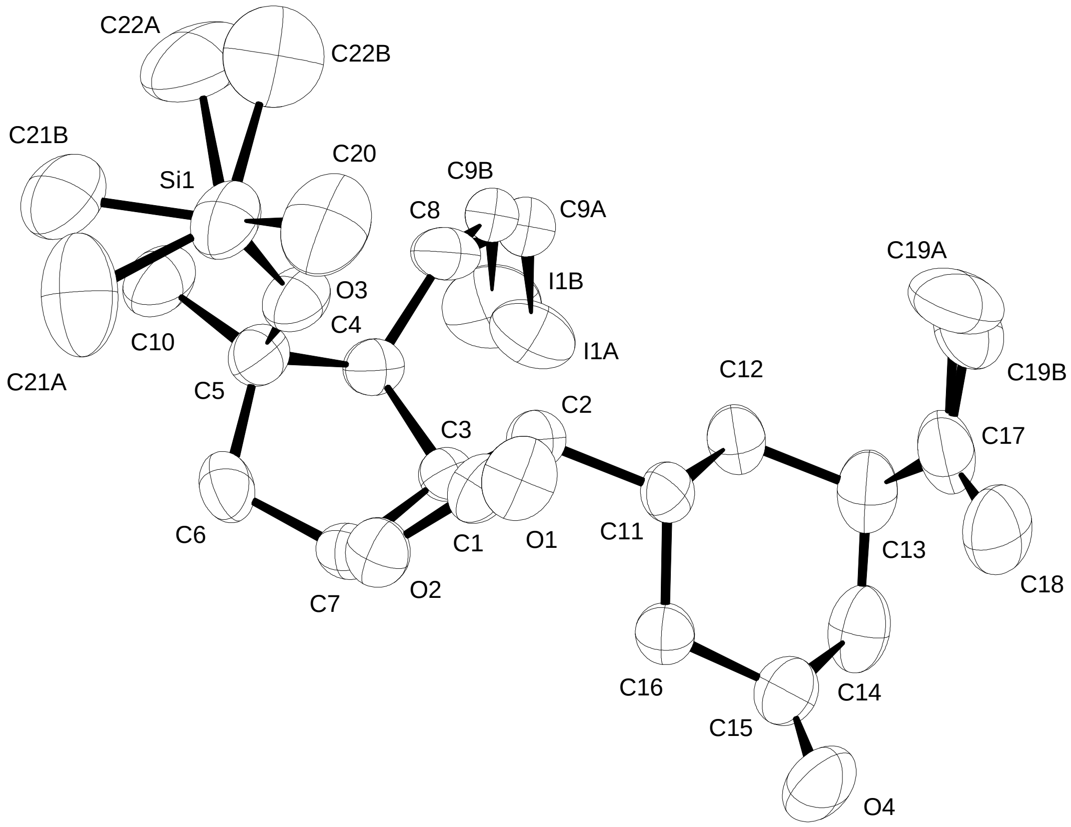

Figure S1. Structure of compound **25** in the solid state (crystallographic numbering scheme).

X-ray Crystal Structure Analysis of Compound 25: $C_{22}H_{34}IO_4Si$, M_r = 517.48 g · mol^{-1}, colorless plate, crystal size 0.110 x 0.104 x 0.021 mm^3, orthorhombic, space group $P2_12_12_1$ [19], a = 9.7885(13) Å, b = 10.6475(14) Å, c = 23.806(3) Å, V = 2481.2(6) Å^3, T = 150(2) K, Z = 4, D_{calc} = 1.385 g · cm^3, λ = 0.71073 Å, μ(Mo-K_α) = 1.361 mm^{-1}, analytical absorption correction (T_{min} = 0.87, T_{max} = 1.00), Bruker-AXS D8 Venture diffractometer with Photon-II detector and IµS micro focus X-ray source, 2.095 < θ < 28.699°, 74135 measured reflections, 6415 independent reflections, 4127 reflections with $I > 2\sigma(I)$, R_{int} = 0.1007, 299 parameters, S = 1.016, absolute structure parameter = 0.010(13), residual electron density +0.4 (1.18 Å from I1A) / –0.4 (1.09 Å from C9A) e · Å^{-3}. The structure was solved by *SHELXT* and refined by full-matrix least-squares (*SHELXL*) against F^2 to R_1 = 0.047 [$I > 2\sigma(I)$], wR_2 = 0.118. **CCDC-2366849**

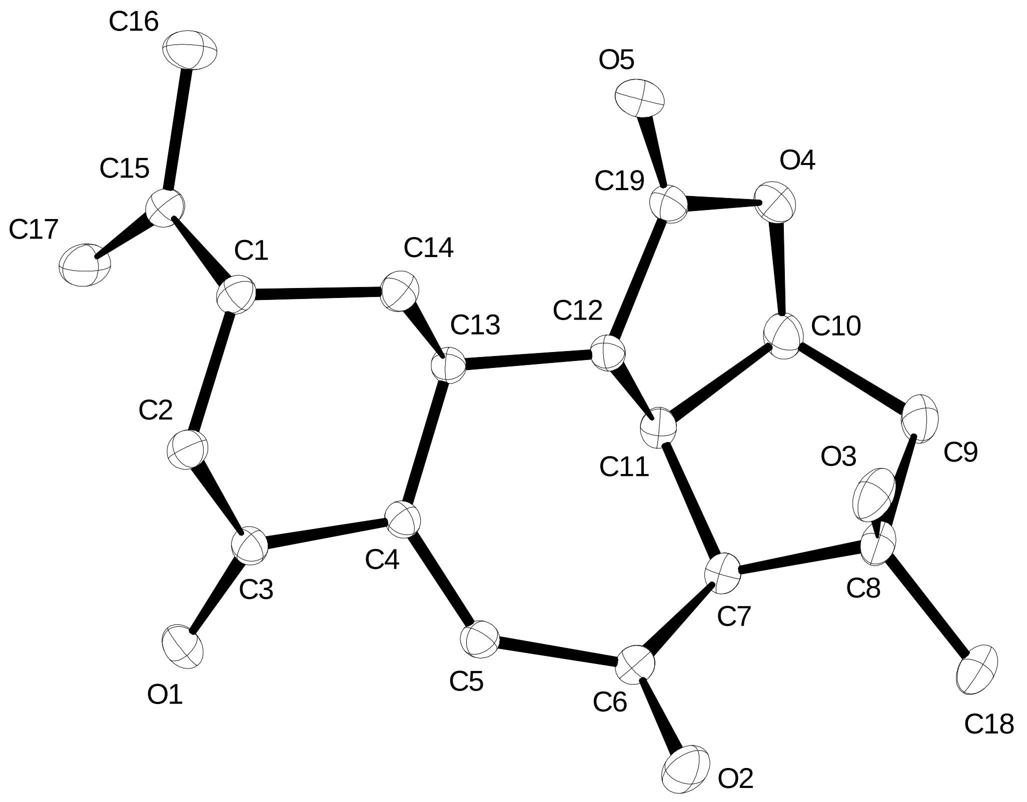

Figure S2. Structure of scabrolide B ((–)-**3**) in the solid state

X-ray Crystal Structure Analysis of Compound (–)-3: $C_{19} H_{22} O_5$, M_r = 330.36 g · mol^{-1}, translucent colorless prism, crystal size 0.381 x 0.213 x 0.120 mm^3, tetragonal, space group *P4₁2₁2* [92], *a* = 11.6959(4) Å, *b* = 11.6959(4) Å, *c* = 23.5582(10) Å, *V* = 3222.6(2) Å^3, *T* = 100(2) K, *Z* = 8, D_{calc} = 1.362 g · cm^3, λ = 0.71073 Å, $\mu(Mo\text{-}K_\alpha)$ = 0.098 mm^{-1}, analytical absorption correction (T_{min} = 0.94, T_{max} = 0.99), Bruker-AXS D8 Venture diffractometer with Photon-II detector and IµS micro focus X-ray source, 1.944 <θ< 45.363°, 689801 measured reflections, 13541 independent reflections, 12402 reflections with *I* > 2σ(*I*), R_{int} = 0.0988, 255 parameters, *S* = 1.074, absolute structure parameter = 0.04(10), residual electron density +0.4 (0.68 Å from C6) / –0.2 (0.21 Å from H5) e · Å^{-3}. The structure was solved by *SHELXT* and refined by full-matrix least-squares (*SHELXL*) against F^2 to R_1 = 0.032 [$I > 2\sigma(I)$], wR_2 = 0.090. **CCDC-2366850**.

DP4+ Probability Analyses of Scabrolide B

Overview and Context. The first characterization in 2002 of the marine natural product scabrolide B proposed the structure **2** based on NMR data.[1] It was however established by total synthesis in 2022 that its correct structure differed from the one proposed.[2] It is well known that NMR-based elucidations of novel structures suffer from sparse and semi-quantitative data and it is not uncommon that erroneously reported natural product structures are later revised to a constitutional isomer or diastereoisomer.[3-5] For scabrolide B, it was even postulated that the inverted chirality at position C_{12} was a likely solution, but the synthetic 12-*epi*-**2** did not produce a matching NMR spectrum either.[2]

In the last 20 years, there has been some important progress in the ability to evaluate structure candidates against a set of experimental NMR data. The popular parameter DP4 scores the probability of structure candidates by correlating experimental and calculated NMR shifts (and couplings) using Bayesian statistics.[6, 7] With the advent of efficient DFT prediction of NMR parameters, the accuracy of this approach has made it a convenient tool in structure elucidation.

In the absence of access to a sample of authentic scabrolide B or to the original NMR spectra from 2002,[1] we reasoned that a statistical evaluation of the data available in the original article might give us a new lead to consider for a second attempt at its total synthesis. With the nominal scabrolide B having potentially one or more wrongly assigned stereocentres out of seven, we decided to evaluate systematically all possible diastereoisomers. Furthermore, a close look at the original assignment revealed nearly overlapping ^{13}C shifts for the two ketones at positions C3 and C6 (202.6 vs 202.3 ppm). This situation could lead to the misinterpretation of a decisive long-range C-H correlation; indeed, the correlation from H13 to one of the ketones is crucial to identifying the olefinic end (C4 or C5) at which the ring-closing bond from C13 is formed. We therefore rationalized that our statistical evaluation should also consider the constitutional isomer (and its diastereoisomers) for which a bond exists between C13 and C4 (rather than C5) leading to a 6-7-5 fused ring system rather than the proposed 7-6-5 (Figure S3).

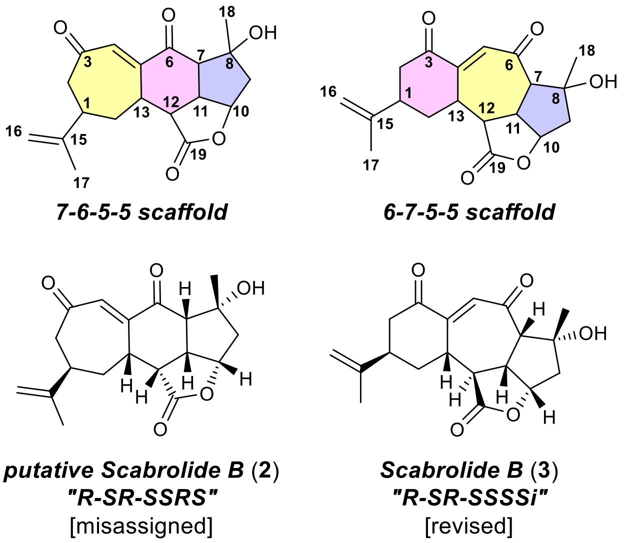

Figure S3. Basic ring scaffolds 7-6-5 and 6-7-5 with carbon numbering considered in the probabilistic analysis and the two reported constitutional isomers of scabrolide B: the original structure proposal **2** (2002)[1] (and the revised structure **3** (2023)).[8] The configuration nomenclature ***R-SR-SSSS*** refers to [1***R***, 7***S***, 8***R***, 10***S***, 11***S***, 12***S***, 13***S***]; the suffix ***i*** refers to the alternate scaffold 6-7-5.

Here we report the application of a thorough DFT-based probabilistic study to identify the correct constitutional isomer of scabrolide B in the correct stereochemical format based on the available NMR ^{1}H, ^{13}C chemical shifts combined with J_{HH} coupling constants. The plausible scabrolide B constitutional isomers (7-6-5 and 6-7-5) comprise seven stereocentres, each resulting in 128 alternative diastereoisomers, which are grouped into 64 pairs of NMR-equivalent enantiomers. Therefore, the DFT calculations aiming to determine Boltzmann-averaged ^{1}H and ^{13}C chemical shieldings and coupling constants over an ensemble of interchanging conformers were applied to a total of 128 configurations (64 for each of the two constitutional isomers). The results were compared to the values of natural scabrolide B reported by Sheu *et al.*[1] and evaluated with a Bayesian analysis, the J-DP4+ tool as proposed by the group of Sarotti.[6, 7]

The results of this approach, which we benchmarked on the known rigid terpenoid fragilolide A (see below), points to a single compound with very high confidence. It features a 6-7-5 scaffold and is characterized by the stereochemistry ***R-SR-SSSSi*** as shown in Figure S3. This result supports our intuition that scabrolide B is a constitutional isomer of the nominal structure rather than a diastereoisomer. This convincing result launched us onto a new total synthesis towards this target.

The revision of the structure of scabrolide B reported in 2023 by X-ray crystallography[8] preceded the completion of our total synthesis reported herein but confirmed that the correct target was predicted and pursued.

Methods. The strategy for the probabilistic study comprised the following steps

1. Initial conformation ensemble generation (MCMM or CREST)
2. DFT-based energetic sorting (CENSO/ORCA + GFN-xTB)
   a. Cheap pre-screening
   b. Pre-screening
   c. DFT-Optimization
   d. Higher DFT Refinement
   e. NMR parameter calculation
3. Bayesian probabilistic evaluation (J-DP4+)

and was implemented on fragilolide A – a known terpenoid for benchmarking – and on scabrolide B to determine it structure.

1. Initial conformation ensemble generation (MCMM or CREST).

All configurations for conformational sampling were generated using Maestro 13.7 by Schrödinger.[9] Monte Carlo Multiple Minimum (MCMM) conformational searches were performed using MacroModel[10, 11] as implemented in the Schrödinger package.[12] The conformational searches were performed using the OPLS4 force field with $CHCl_3$ as solvation model, 5.000 iterations for the minimization, 10.000 search steps, an RMSD cutoff of 0.4 Å and an energy window for saving structures of 25.1 kJ/mol (6.00 kcal/mol). Typically, this procedure produced between 6 and 125 conformers for the numerous diastereoisomers with differing flexibilities.

For the benchmarking (fragilolide A) of the J-DP4+ method, CREST-GFN1[13] and CREST-GFN2[14] conformational searches were also performed as an alternative using an energy window of 6 kcal/mol (25.1 kJ/mol), an RMSD cutoff of 0.4 Å, and chloroform as a solvation model.

2. DFT-based Energetic Sorting (CENSO/ORCA + GFN-xTB)

The various conformer ensembles were then submitted to two pre-screening steps. The first cheap pre-screening was used to discard high-lying conformers and rotamers based on single-point energies (>4.0 kcal/mol) calculated with B3LYP-D3/def2-SV(P). The resulting conformers were further pre-screened by single-point calculations including accurate electronic and solvation energies at the r2SCAN-3c level keeping only conformers below a threshold of 3.5 kcal/mol. These were then optimized at the r2SCAN-3c level and their Boltzmann weights were calculated; those contained within a 2.5 kcal/mol window and contributing to a total Boltzmann population of 99% were further considered. Then, the Boltzmann weights were refined at a higher (hybrid) DFT level (pw6b95/def2-TZVPD) and again Boltzmann populations up to 99% were preserved. Finally, the ensemble-averaged NMR properties (^{1}H and ^{13}C shieldings and coupling constants) were calculated (pbe0/def2-TZVP) for the populated conformers based on their Boltzmann weights. For all computations except the initial B3LYP-D3/def2-SV(P) single-point calculations and the NMR shift calculations, thermostatic contributions were calculated within the rigid rotor harmonic oscillator approximation at the GFN2-xTB level, while the solvent was implicitly considered using the CPCM model[15] for chloroform. Conformer sorting, optimization, and NMR calculations were performed using the CENSO v1.1.2 code[16] linked to the ORCA 5.0.1[17, 18] and xTB 6.5.0[19] codes.

3. Bayesian probabilistic evaluation (J-DP4+)

All shielding values were extracted from the CENSO results and converted directly to unscaled chemical shifts by subtracting the reference shielding values of TMS (^{1}H = 31.545 ppm and ^{13}C = 188.653 ppm), calculated with the same energetic sorting procedure. The relevant $^{2}J_{HH}$ and $^{3}J_{HH}$ coupling constants were also directly extracted. For the J-DP4+ analysis the assigned ^{1}H and ^{13}C chemical shifts and ^{1}H-^{1}H couplings available from the literature were introduced in the Excel spreadsheets freely available at https://sarotti-nmr.weebly.com. Custom J-DP4+ settings are shown in Table S1 and are based on custom probability distribution parameters, evaluated with the custom-DP4+ Excel sheet.[20]

Table S1. J-DP4+ custom settings used

	TMS	σ	ν
**H**	31.545	0.185	14.18
**C**	188.653	2.306	11.38
**J**	-	0.992	3.06
	**Slope scaling J**	0.9509	
	**Intercept scaling J**	-0.1405	

Results. As far as we know, the use of the ensemble-averaged NMR parameters calculated with the open-access package CENSO/ORCA (rather than the commonly used Gauge-including-atomic-orbital (GIAO) NMR shielding calculations (e.g. Gaussian)) in the J-DP4+ evaluation tool was not reported previously. This combination was therefore benchmarked using NMR data of the known terpenoid fragilolide A (8 stereocenters).[21] The alternative 7-6-5 scaffold (Figure S4), similar to the one reported for scabrolide B[1] was created in addition to the reported structure, to generate a representative structure pool for the problem at hand. The conformational searches for all diastereoisomeric configurations of the 6-7-5 and 7-6-5 scaffolds were performed**,** the results were optimized and the NMR data were extracted as described.

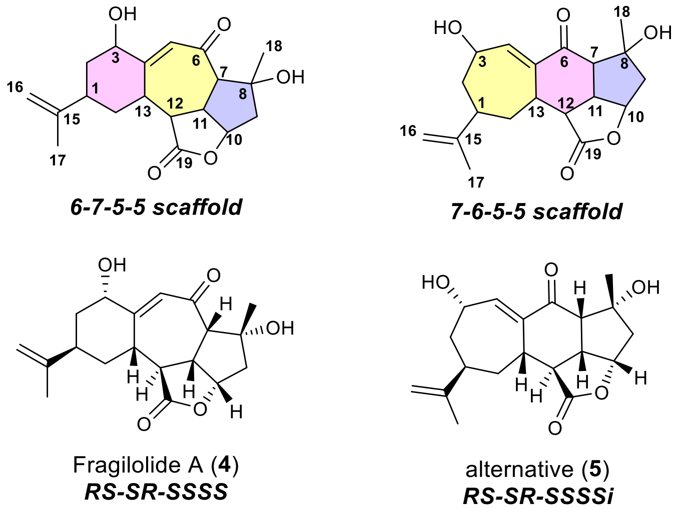

Figure S4. Basic ring scaffolds 6-7-5 and 7-6-5 with carbon numbering used in the test run. The target known structure of fragilolide A (**5**)[21] and a generated alternative constitutional isomer **5*i***. The configuration nomenclature ***RS-SR-SSSS*** refers to [1***R***, 3***S***,7***S***, 8***R***, 10***S***, 11***S***, 12***S***, 13***S***]; the suffix ***i*** refers to the alternate scaffold 7-6-5.

The two scaffolds in Figure S4 result in 256 diastereoisomers each, or 128 pairs of enantiomers, which were submitted to conformational sampling. Given the low degree of flexibility of these fused ring-systems, we anticipated that low-level Monte Carlo Multiple Minima (MCMM) conformational searches using MacroModel as implemented in the Schrödinger package[12] would be able to give a suitable ensemble for this purpose. Besides MCMM, we used Conformer-Rotamer Ensemble Sampling Tool (CREST) using two different semi-empirical quantum mechanical methods to compute Geometries, Frequencies and Non-covalent (GFN) interactions. We generated CREST ensembles using both GFN1[13] and GFN2[14] for the 6-7-5 isomers in addition to the MCMM searches. For the 7-6-5 isomer, only MCMM searches were performed.

As shown in Figure S5, the J-DP4+ probability scores of more than 99+% clearly identifies the correct isomer of fragilolide A (**5**) out of all 256 possibilities. The method used to generate the conformational ensemble (CREST-GFN1, CREST-GFN2 or MCMM) did not influence the probabilities significantly for the top five selected conformations. Only conformations from the 6-7-5 scaffold were selected from the 256 MCMM generated ensemble (Figure S5c). In all cases, the correct diastereoisomer ***RS-SR-SSSS*** was given a probability score over 99.99%, independent of the conformational search method employed. When comparing the calculated NMR parameters from all three conformational search methods on the diastereoisomer ***RS-SR-SSSS*** with J-DP4+, all ensembles were selected with significant probabilities (Figure S5d). This indicates that the three conformational search methods all give reliable results and so only MCMM was used for the configurational question at hand regarding scabrolide B. Table S2 summarizes the experimental and back-calculated ^{1}H and ^{13}C chemical shifts for the three different methods for the best fitting configuration, with a probability of 99%+ according the J-DP4+ analysis.

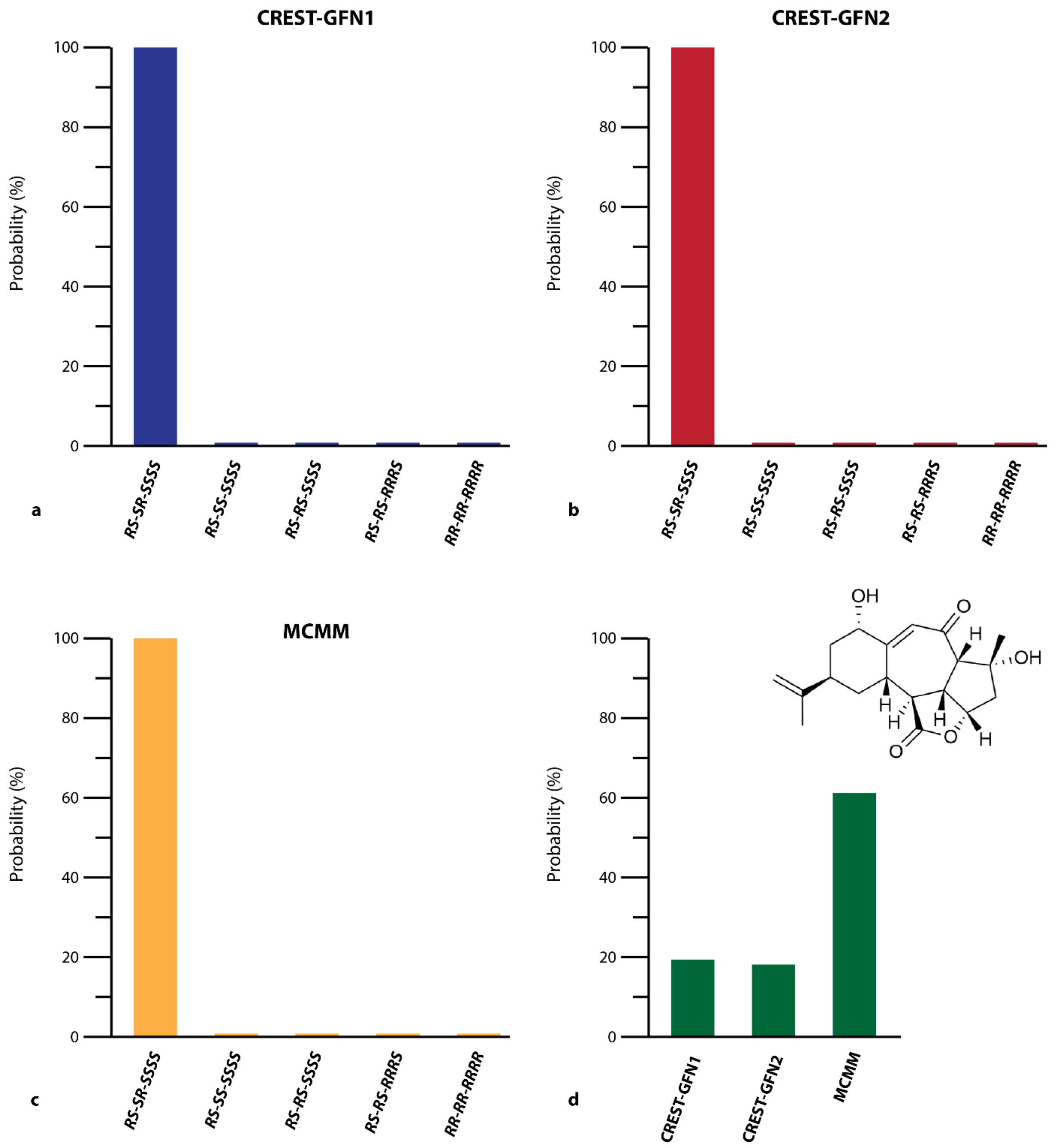

Figure S5. The five best J-DP4+ probability scores for the structure candidates based on the NMR data of fragilolide A using three different conformation sampling methods: a) CREST-GFN1 in blue, b) CREST-GFN2 in red and c) MCMM in yellow. Only the latter considered both constitutional isomers. The same five diastereoisomers were selected by all three methods, and ***RS-SR-SSSS*** was selected with a probability over 99.99% in each case. Interestingly, only constitutional isomers corresponding to the 6-7-5 scaffold were selected among the five best (c). The graph in green (d) shows the J-DP4+ probability scores of ***RS-SR-SSSS*** from the different conformational search methods.

Table S2. Assignment of fragilolide A ***RS-SR-SSSS*** (**5**)[21] and back-calculated shifts according the J-DP4+ analysis for the three different conformational sampling methods (CREST-GFN1, CREST-GFN2 and MCMM).

	**Experimental**		**CREST-GFN1**[a]		**CREST-GFN2**[a]		**MCMM**	
**Atom**	**^{13}C (ppm)**	**^{1}H (ppm)**	**Calc. ^{13}C (ppm)**	**Calc. ^{1}H (ppm)**	**Calc. ^{13}C (ppm)**	**Calc. ^{1}H (ppm)**	**Calc. ^{13}C (ppm)**	**Calc. ^{1}H (ppm)**
**1**	38.3	2.59	40.18	2.60	40.26	2.58	40.07	2.60
**2a**	40.0	2.58	39.45	2.63	40.20	2.64	41.14	2.58
**2b**		1.64		1.59		1.54		1.62
**3**	69.1	4.35	69.32	4.36	69.36	4.28	68.90	4.31
**4**	160.2	-	165.92	-	165.99	-	163.41	-
**5**	122.0	6.27	117.84	6.15	117.96	6.14	118.76	6.18
**6**	203.6	-	202.05	-	201.90	-	203.03	-
**7**	62.6	2.94	62.91	3.07	62.35	3.06	63.08	3.08
**8**	81.0	-	80.88	-	80.94	-	80.66	-
**9a**	47.4	2.38	46.19	2.20	46.28	2.28	45.80	2.13
**9b**		2.22		2.16		2.13		2.17
**10**	79.5	4.98	80.39	4.79	80.10	4.80	80.62	4.79
**11**	45.2	3.12	46.00	3.10	46.09	3.09	45.44	3.15
**12**	45.0	3.38	45.52	3.46	44.81	3.53	46.24	3.46
**13**	41.5	2.48	43.24	2.56	42.71	2.60	43.02	2.62
**14a**	34.5	3.35	35.15	3.50	35.40	3.47	35.06	3.55
**14b**		1.34		1.28		1.29		1.27
**15**	145.3	-	148.77	-	148.67	-	148.96	-
**16a**	111.5	5.02	107.10	5.11	107.20	5.12	107.23	5.10
**16b**		5.02		5.10		5.14		5.09
**17**	22.5	1.87	22.25	1.94	22.11	1.94	22.16	1.93
**18**	30.5	1.63	28.06	1.52	28.63	1.52	27.35	1.53
**19**	176.6	-	175.08	-	175.35	-	175.35	-

[a]For CREST-GFN1 and CREST-GFN2 only the 128 diastereoisomers corresponding to the 6-7-5 scaffold were included, whereas for the MCMM analysis 256 configurations were included coming from both the 6-7-5 and the 7-6-5 scaffold.

Since this method precisely selected the correct structure of fragilolide A with unanimous J-DP4+ scores of nearly 100%, we employed the same conformation sampling, DFT-based energetic sorting, NMR parameters calculation and J-DP4+ analysis on all the constitutional and stereoisomers of scabrolide B (Figure S3, 128 configurations) with the hope of clearly identifying a dominant candidate. Based on the comparison of the calculated and experimental NMR parameters (Table S2), the configuration ***R-SR-SSSSi*** (or its enantiomeric partner) corresponding to structure **3** was determined to be the best fit. As for fragilolide A, the J-DP4+ analysis resulted in a clear-cut conclusion in favour of this structure with a probability close to 100% (Figure S6). This structure corresponds to a 6-7-5 isomer of nominal scabrolide B, where a covalent bond exist between C13 and C4; the stereocenter C12 is inverted compared to that in nominal scabrolide B. Table S3 summarizes the experimental data and the back-calculated NMR data for the best fitting diastereoisomer.

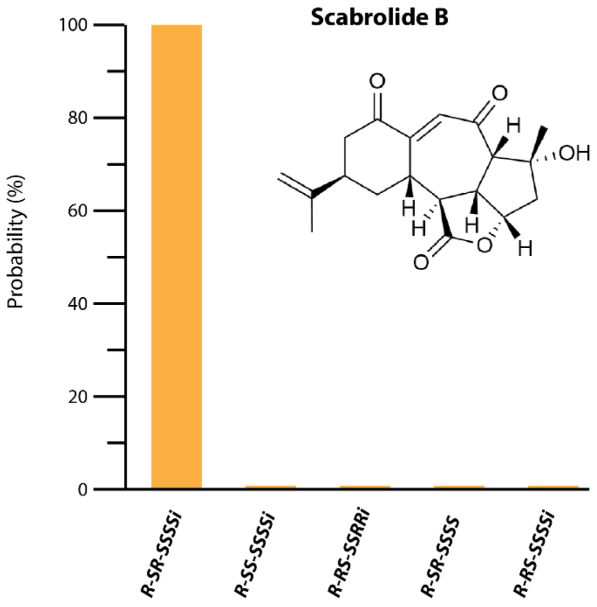

Figure S6. The five best J-DP4+ probability scores for the structure candidates of scabrolide B, based on the originally published NMR parameters (^{1}H and ^{13}C chemical shifts and ^{1}H homonuclear coupling constants).[1] The candidate pool includes all possible alternate diastereoisomers of **2** (7-6-5 scaffold) as well as of the constitutional isomer **3** (6-7-5 scaffold). The selected structure (**R-SR-SSSS*i*** (99%+)) corresponds to the scabrolide B structure with 6-7-5 scaffold recently obtained by X-ray crystallography.[8]

Table S3. Assignment of scabrolide B ***R-SR-SSSSi*** (**3**) and back-calculated shifts according the J-DP4+ analysis, with the data as reported by Sheu *et al.*[1]

Atom	Exp. ^{13}C (ppm)	Exp. ^{1}H (ppm)	Multiplet	Exp. J_{HH} in Hz (identified coupling partner)	Calc. ^{13}C (ppm)	Calc. ^{1}H (ppm)	Calc. J_{HH} in Hz (identified coupling partner)
**1**	38.9	2.81	m		41.12	2.89	
**2a**	45.0	2.90	ddd	16.0 (2b)<br>4.0<br>2.5 (5)	45.24	3.05	16.95 (2b)<br>0.29 (5)
**2b**		2.60	dd	16.0 (2a)<br>6.0		2.70	16.95 (2a)
**3**	202.2	-	-	-	201.91	-	-
**4**[a]	150.8	-	-	-	152.64	-	-
**5**[a]	130.5	6.34	d	3.0 (2a)	127.73	6.37	0.29 (2a)
**6**	202.5	-	-	-	203.17	-	-
**7**	62.4	2.93	d	7.2	63.25	3.18	7.05 (11)
**8**	81.3	-	-	-	80.51	-	-
**9a**	47.4	3.36	d	16.0 (9b)<br>2.5 (10)	46.01	2.25	16.26 (9b)<br>0.97 (10)
**9b**		2.21	ddd	16.0 (9a)<br>9.0 (10)<br>1.6		2.14	16.26 (9a)<br>7.91 (10)
**10**	79.5	4.98	td	2.5 (9a)<br>9.0 (9b)	80.41	4.88	0.97 (9a)<br>7.91 (9b)
**11**[b]	45.3	3.45	t	10.5 (12)	45.56	3.19	8.70 (12)
**12**[b]	45.3	3.14	dt	10.5 (11)<br>7.8	47.10	3.40	8.70 (11)
**13**	41.6	2.75	m		43.67	2.83	-
**14a**	30.5	3.30	dtd	10.0 (14b)<br>4.0<br>2.5	30.25	3.45	14.12 (14b)
**14b**		1.71	tdd	10.0 (14a)<br>5.0<br>2.5		1.74	14.12 (14a)
**15**	146.4	-	-	-	150.51	-	-
**16a**	112.7	4.95	s	-	108.82	4.92	-
**16b**		4.72	s	-		5.14	-
**17**	21.8	1.83	s		21.38	1.93	-
**18**	30.0	1.63	s		26.59	1.56	-
**19**	175.9	-	-	-	174.22	-	-

[a] The atom numbers of 4 and 5 are switched for the correct constitutional isomer (**3**) as compared to Sheu et al.[1] because of the difference in atom-labeling between the two isomers.

[b] Chemical shift for 11 and 12 were used as reported by Sheu et al. for the J-DP4+ analysis.[1] Once the total synthesis of the scabrolide B was successful, it turned out the chemical shifts of 11 and 12 were assigned incorrectly in the original publication.

Intelligence Gathering

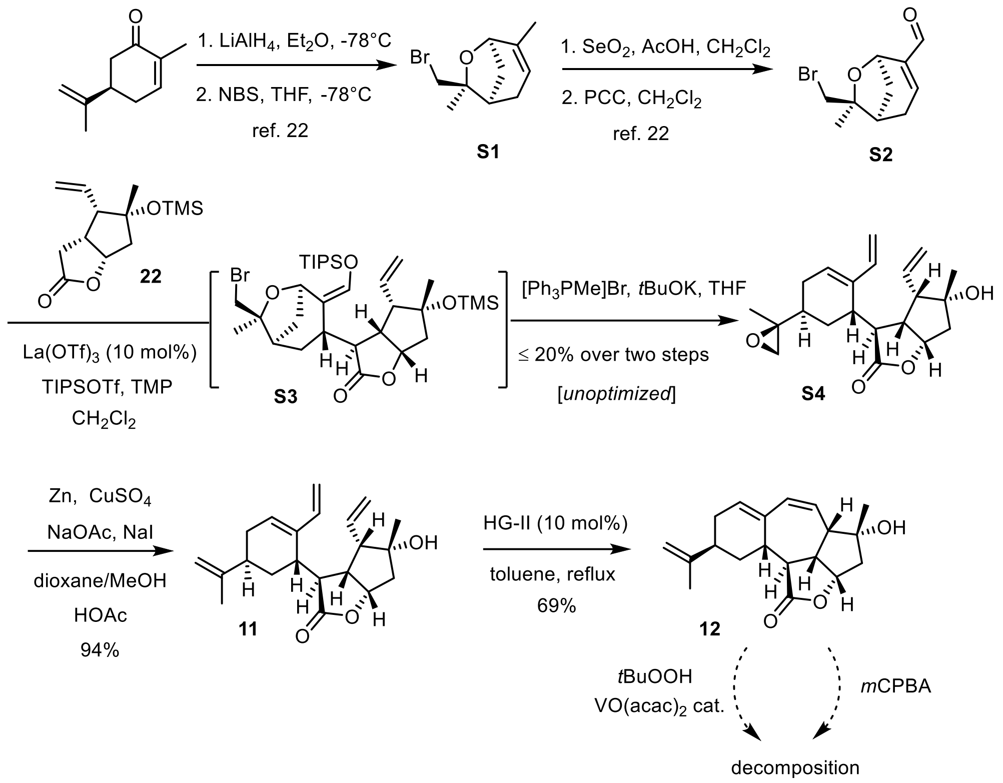

Scheme S1. Attempted synthesis of scabrolide B via compound **11**; the first steps were adapted from ref. 22

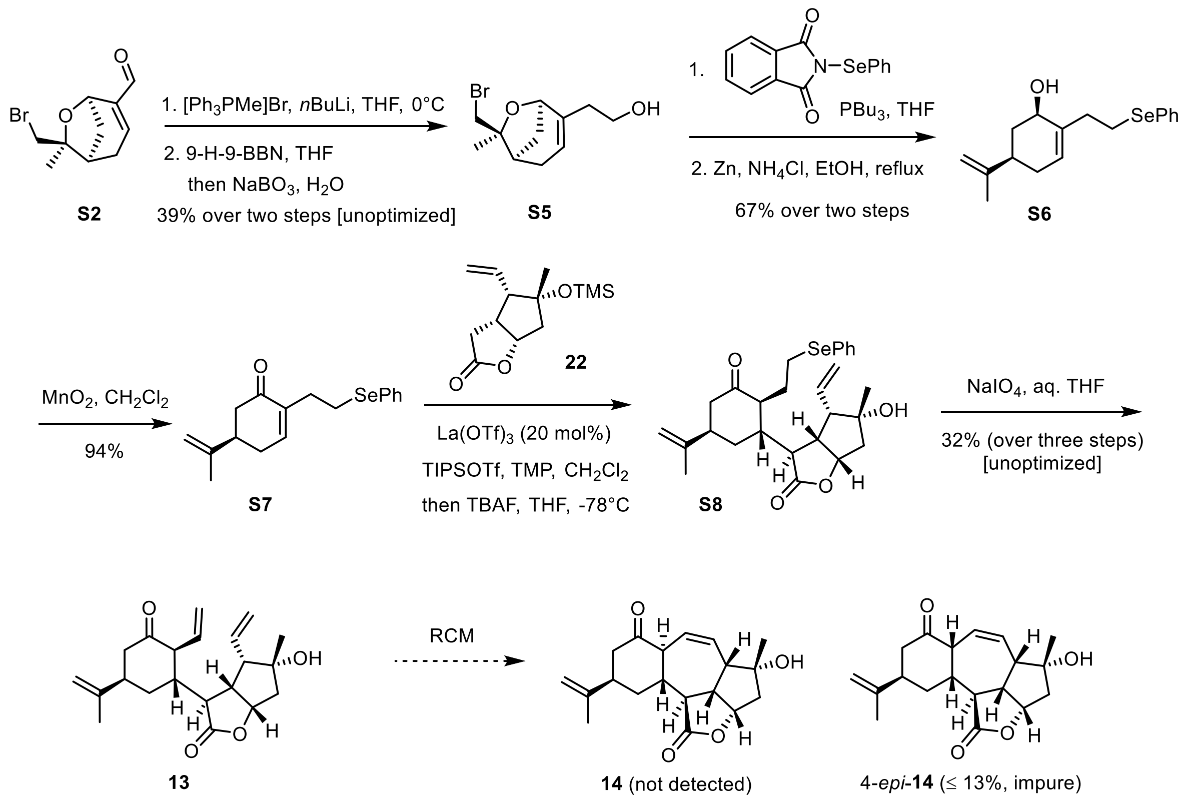

Scheme S2. Attempted synthesis of scabrolide B by RCM of compound **13**

While the double bond of **11** to be metathesized is conjugated to a flattened cyclohexene ring, the vinyl group in **13** branches off a *trans*-disubstituted cyclohexane; the latter substrate did not cyclize to afford any of the *trans*-fused cycloheptane derivative **14**; traces of 4-*epi*-**14** formed by epimerization prior to RCM were detected in a few runs (see Table S4). Attempts to drive the epimerization by addition of Bronsted or Lewis acids to the mixture were to no avail.

The fact that 4-*epi*-**14** featuring a *cis*-fused ring system was formed, though in low yield, suggested that intramolecular alkenylation might be viable, since the reaction could initially lead to a less strained *cis*-fused skeleton before isomerization of the double bond into conjugation might take place.

Table S4. Attempted ring-closing metathesis of compound **13**; the reactions were carried out at a concentration of 4.5 mM unless stated otherwise; each batch of catalyst corresponds to 20 mol%

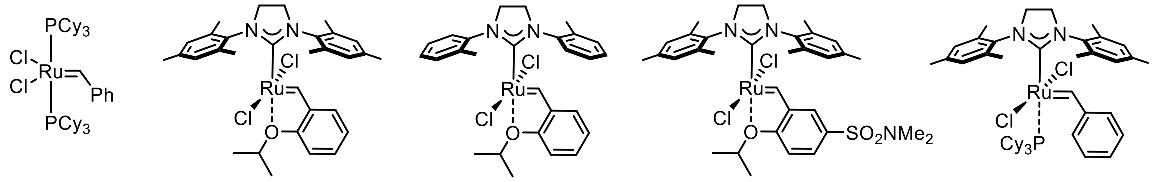

Ru-1 **Ru-2** **Ru-3** **Ru-4** **Ru-5**

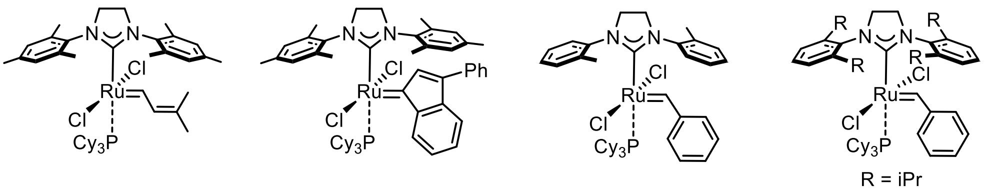

Ru-6 **Ru-7** **Ru-8** **Ru-9**

Entry	Conditions	Results
1	**Ru-1**, CH_2Cl_2, 40 °C, 18 h	no reaction
2	**Ru-1**, Ti(*i*OPr)$_4$,[23] CH_2Cl_2, 40 °C, 18 h	no reaction
3	**Ru-1**, toluene, 100 °C, 72 h	no reaction
4	**Ru-2**, toluene, 100 °C, 18 h	no reaction
5	**Ru-3**, CH_2Cl_2, 40 °C, 18 h	no reaction
6	**Ru-3** (x 3), toluene, 100 °C, 96 h	no reaction
7	**Ru-4** (x 5), toluene, 100 °C, 120 h	slow decomposition
8	**Ru-5**, CH_2Cl_2, 40 °C ,18 h	no reaction
9	**Ru-5**, 1,2-dichloroethane, 80 °C, 18 h	no reaction
10	**Ru-5** (x 2), toluene, 100 °C, 40 h	partial decomposition, **4-*epi*-14** (≈12%, NMR)
11	**Ru-5**, toluene, 120 °C, 18 h	decomposition
12	**Ru-5** (x 4), toluene, 100 °C, 96 h, c = 0.45 mM	partial decomposition, **4-*epi*-14** (≈13%, NMR)
13	**Ru-5**, TsOH, toluene, 100 °C, 17 h	decomposition
14	**Ru-5** (x 2), AcOH, toluene, 100 °C, 40 h	decomposition
15	**Ru-5** (x 2), benzoquinone, toluene, 100 °C, 40 h	decomposition
16	**Ru-5** (x 2), Ti(*i*OPr)$_4$, toluene, 100 °C, 40 h	no reaction
17	**Ru-6** (x 3), toluene, 100 °C, 96 h	slow decomposition
18	**Ru-7** (x 3), toluene, 100 °C, 96 h	slow decomposition
19	**Ru-8** (x 3), toluene, 100 °C, 96 h	slow decomposition
20	**Ru-9** (x 3), toluene, 100 °C, 96 h	slow decomposition

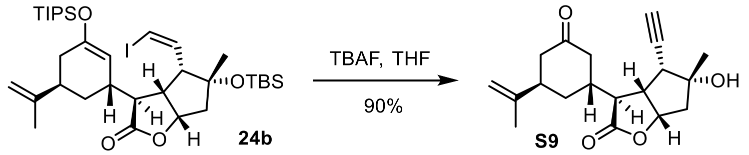

Scheme S3. Attempted cleavage of the TIPS enol ether in presence of the tertiary –OTBS ether led to global deprotection and concomitant dehydrohalogenation

The late-stage cleavage of a C8-OTBS ether under various conditions as the second last step of the total synthesis of scabrolide A reported by the Sarlah group was fairly low yielding.[24] For the problem shown in Scheme S3 and this literature precedent, the alcohol protecting group was changed to C8-OTMS.

Table S5. Studies on ring closure by palladium catalyzed intramolecular alkenylation; the reactions were performed at a concentration of 10 mM, unless stated otherwise

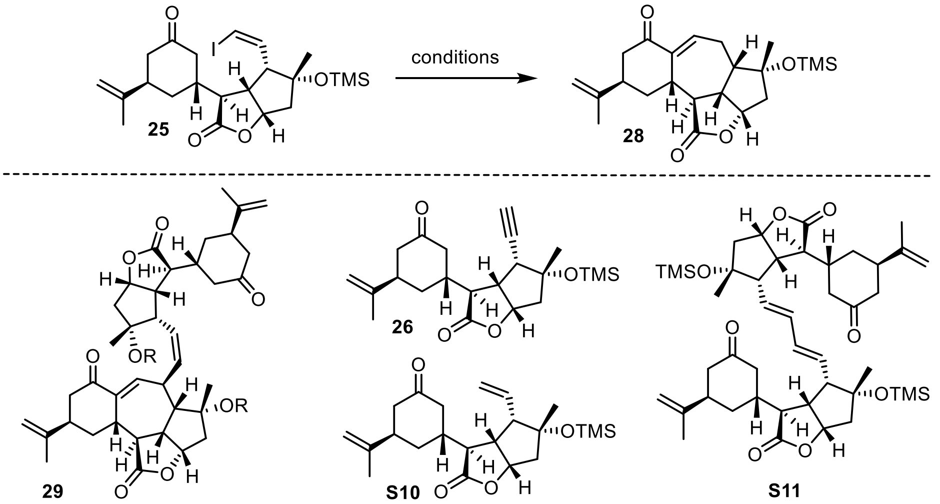

Entry	Conditions	Results[a]
1	$Pd(PPh_3)_4$ 0.2 eq, PhOH 1.7 eq, *t*BuOK 1.5 eq, toluene, 65 °C, 2 h	**28** (20%)
2	$Pd(PPh_3)_4$ 0.2 eq, PhOH 3.5 eq, *t*BuOK 3.0 eq, toluene, 65 °C, 2 h	**28** (30%)
3	$Pd_2(dba)_3$ 0.2 eq, SPhos 0.6 eq, PhOH 3.5 eq, *t*BuOK 3 eq, toluene, 65 °C, 2 h	**28** (30%)
4	$Pd(PPh_3)_4$ 0.2 eq, *t*BuOK 3.0 eq toluene, 65 °C, 1.5 h	**26** and **S10**
5	$Pd(PPh_3)_4$ 0.2 eq, PhOH 3.5 eq, *t*BuOK 3 eq, THF, 60 °C, 20 h	**26** and **S10**
6	$Pd(PPh_3)_4$ 0.2 eq, *t*BuOK 3.0 eq, THF, RT, 5 min	unidentified product
7	$Pd(PPh_3)_4$ 0.2 eq, Cs_2CO_3 3.0 eq, toluene, 65 °C, 2.5 h	**S11**
8	$PdCl_2(PPh_3)_2$ 0.2 eq, Cs_2CO_3 3.0 eq, TEA 3.0 eq, toluene, 90 °C, 24 h	**S10** and **S11**
9	$Pd(PPh_3)_4$ 0.2 eq, *t*BuONa 3.0 eq, dioxane, 60 °C, 20 h	**S10** and **S11**
10	$PdCl_2$dppf 0.2 eq, K_2CO_3 3.0 eq, MeOH, 60 °C, 2 h	**S10** and **S11**
11	$Pd(OAc)_2$ 0.05 eq, PPh_3 0.3 eq, TBAB 1 eq, K_2CO_3 4.0 eq, DMF:H_2O (10:1), 90 °C, 20 h	**S10** and **S11**
12	$Pd(PPh_3)_4$ 0.2 eq, 2,6- dimethylphenol 3.5 eq, *t*BuOK 3.0 eq, toluene, 65 °C, 70 min	**28** (21%), **29** (32%)
13	$Pd(PPh_3)_4$ 0.2 eq, 2,6- diisopropylphenol 3.5 eq, *t*BuOK 3.0 eq, toluene, 65 °C, 70 min	**28** (46%), **29** (13%)
14	$Pd(PPh_3)_4$ 0.2 eq, 2,6-di-*tert*-butylphenol 3.5 eq, *t*BuOK 3.0 eq, toluene, 65 °C, 70 min	**28** (7%), **29** (4%)
15	$Pd(PPh_3)_4$ 0.2 eq, 2,6- diisopropylphenol 3.5 eq, *t*BuOK 3.0 eq, toluene, 60 °C, 70 min, c = 2 mM	**28** (57%), **29** (29%)

[a] Yields of isolated products; for entries, in which no yields are reported, the analysis is solely based on the inspection of the crude product by NMR

General Information

Unless stated otherwise, all reactions were carried out in flame-dried glassware using anhydrous solvents under argon atmosphere.

The solvents were purified by distillation over the indicated drying agents and were transferred under argon: THF, Et_2O (Mg/anthracene); acetonitrile, 2,6-lutidine, CH_2Cl_2, 1,2-dichloroethane, nitromethane (CaH_2); toluene (Na/K alloy); MeOH (Mg, stored over MS 3 Å). DMSO, DMF, Et_3N, pentane and pyridine were dried by an adsorption solvent purification system based on molecular sieves.

Thin layer chromatography (TLC): Macherey-Nagel pre-coated plates (POLYGRAM®SIL/UV254); detection was achieved under UV-light (254 nm) and by staining with cerium ammonium molybdenate. Flash chromatography: Merck silica gel 60 (40–63 µm) with distilled or HPLC grade solvents.

NMR: Spectra were recorded on Bruker AV 400, AV 500, AVIII 600 or AVneo 600 spectrometers in the indicated solvents; chemical shifts (δ) are given in ppm relative to TMS, coupling constants (*J*) in Hz. All spectra were recorded at 25 °C. The solvent signals were used as references and the chemical shifts converted to the TMS scale ($CDCl_3$: δ_C = 77.16 ppm; residual $CHCl_3$ in $CDCl_3$: δ_H = 7.26 ppm; CD_2Cl_2: δ_C = 53.84 ppm, residual $CDHCl_2$: δ_H = 5.32 ppm). Multiplicities are indicated by the following abbreviations: s: singlet, d: doublet, t: triplet, q: quartet, p: pentet, hept: heptet, m: multiplet, br. s: broad singlet. ^{13}C NMR spectra were recorded in ^1H decoupled manner and the values of the chemical shifts are rounded to one decimal point. Signal assignments were established using HSQC, HMBC, COSY and NOESY experiments.

IR: Spectra were recorded on an Alpha Platinum ATR instrument (Bruker), wave numbers ($\tilde{\nu}$) in cm^{-1}.

MS (ESI-MS): Finnigan MAT 8200 (70 eV), ESI-MS: ESQ3000 (Bruker), accurate mass determinations: Bruker APEX III FTMS (7 T magnet) or Mat 95 (Finnigan).

Optical rotations ($[\alpha]_D$) were measured at the indicated temperature with an A-Krüss Optronic Model P8000-t polarimeter at a wavelength of 589 nm.

Unless stated otherwise, commercially available compounds (Alfa Aesar, Sigma Aldrich, TCI, Strem Chemicals, ChemPUR) and used as received.

Building Blocks

(*R*)-3-(Prop-1-en-2-yl)cyclohexan-1-one (17). *Catalyst solution*: A mixture comprising $[Rh(cod)Cl]_2$ (27.5 mg, 55.8 µmol), *(R)*-DTBM SEGPHOS (**20**) (136 mg, 0.115 mmol) and methanol (2.5 mL) was stirred for 20 min at room temperature to give a homogeneous dark orange solution.

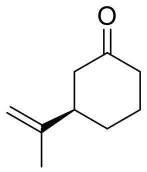

A 100-mL two-necked flask equipped with a reflux condenser was charged with 2,2-dimethyl-1,3-propanediol (2.13 g, 20.5 mmol), hexanes (15 mL), isopropenylboronic acid pinacol ester (**16**) (3.8 mL, 20 mmol), *N,N*-diisopropylethylamine (1.6 mL, 9.2 mmol) and water (5.4 mL). Argon was bubbled through the mixture for five minutes before 2-cyclohexen-1-one (**15**) (1.8 mL, 19 mmol) and the catalyst solution (see above, rinsing with an additional amount of methanol: 0.30 mL) were introduced. The resulting biphasic mixture was stirred at 60 °C (bath temperature) for 24 h. The mixture was cooled to room temperature, diluted with diethyl ether (15 mL), and the layers were separated. The organic layer was washed with aq. HCl (1 M, 20 mL) and water (2 × 15 mL), and the combined aqueous phases were extracted with diethyl ether (3 × 15 mL). The combined extracts

were dried over anhydrous sodium sulfate, the drying agent was filtered off, and the solvent was carefully removed under reduced pressure (200 mbar, 36 °C). The dark amber residue was purified by flash chromatography (silica gel; pentane/diethyl ether, 8:1) to furnish the title compound after careful evaporation of the solvents (150 mbar, 36 °C) as a yellow liquid (2.23 g, 87% yield, 94% *ee*). $[\alpha]_D^{20}$ = +18.2 (c = 1.1, CH_2Cl_2). ^1H NMR (400 MHz, $CDCl_3$): δ = 4.80 – 4.76 (m, 1H), 4.75 – 4.71 (m, 1H), 2.48 - 2.22 (m, 5H), 2.13 – 2.02 (m, 1H), 1.98 – 1.88 (m, 1H), 1.76 – 1.73 (m, 3H), 1.73 - 1.55 (m, 2H); ^{13}C NMR (101 MHz, $CDCl_3$): δ = 211.8, 147.6, 110.2, 46.9, 45.8, 41.4, 30.2, 25.3, 20.8; IR (film) ν/cm^{-1}: 3079, 2938, 2865, 1710, 1645, 1448, 1423, 1376, 1346, 1316, 1271, 1251, 1223, 1182, 1102, 1056, 890, 754, 548, 473; HRMS (GC-EI): calcd. for $C_9H_{14}O$ [M^+]: 138.1039; found: 138.1042.

The enantiomeric purity was determined by GC. The racemic sample needed for comparison was obtained by copper-catalyzed addition of isopropenylmagnesium bromide (CuI, THF, 0 °C) to **15**. The analytical data are in agreement with those reported in the literature.[25]

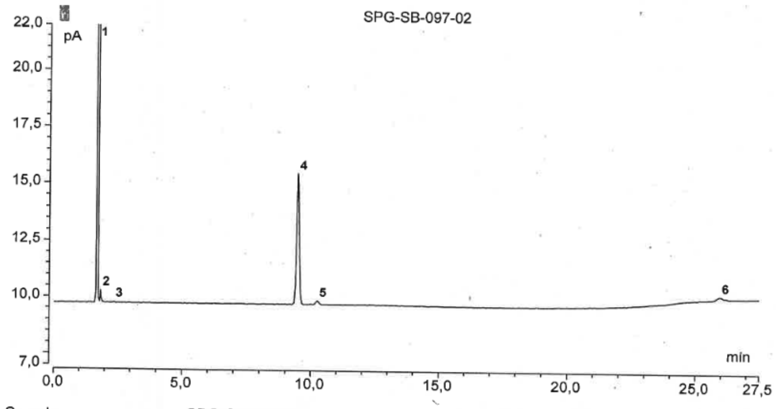

Sample: SPG-SB-097-02
Sequenz: 9299 SPG-SB PH
Sequenz date: 26.09.22

Instrument: GC_413
Measured: 26.09.22 14:00
Processing M.: MPI
Report-File: Verhältnis

Enantiomerenverhältnis
Zuordnung nach Razemat

No.	Ret.Time min	Rel.Area %	Peak Name
4	9,48	97,00	.
5	10,25	3,00	.

Instrument parameters:
Column: 25,0 m Hydrodex-beta-TBDAc-CD 0,25/?df G/681
Temperature: 220 / 120, 12 min iso 8/min 220, 3 min iso / 350
Gas: 0,50 bar Hydrogen
Sample size: 0,2 µL

***(R)*-5-(Prop-1-en-2-yl)cyclohex-2-en-1-one (*(R)*-Norcarvone) (19).** In a 100-mL two-necked flask, *n*-butyllithium (1.6 M in hexanes, 9.8 mL, 16 mmol) was added to a solution of 2,2,6,6-tetramethylpiperidine (2.8 mL, 17 mmol) in THF (32 mL) at 0 °C, and the resulting solution was stirred at this temperature for 20 min before it was cooled to −78 °C. Trimethylsilyl chloride (2.4 mL, 18 mmol) was introduced, followed by slow addition of ketone **17** (1.79 g, 13.0 mmol). The resulting mixture was stirred for 30 min at −78 °C before half-saturated aqueous $NaHCO_3$ (50 mL) was added at this temperature and the mixture was warmed to ambient temperature. The mixture was diluted with diethyl ether (30 mL), the layers were separated, and the organic phase was washed with water (50 mL) and brine (20 mL). The aqueous layers were extracted with diethyl ether (3 × 25 mL), the combined organic phases were dried over sodium sulfate, the drying agent was filtered off, and the solvent was removed under reduced pressure

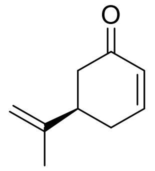

to give a mixture of the corresponding silyl enol ethers (ca. 5:1 by GC) which were directly used in the next step without further purification.

In a 100-mL two-necked flask, diallyl carbonate (2.4 mL, 17 mmol) and $Pd_2(dba)_3$ (949 mg, 1.04 mmol) were added to a solution of the crude material in acetonitrile (40 mL) at room temperature. The resulting dark suspension turned into a dark green, homogeneous mixture within a few minutes. Stirring was continued for 24 h at room temperature before diethyl ether (25 mL) and water (10 mL) were introduced. The mixture was filtered through a pad of Celite®, rinsing with diethyl ether. The organic layer was washed with water (3 × 25 mL) and brine (1 × 25 mL), the combined aqueous phases were extracted with diethyl ether (3 × 15 mL), the organic layers were dried over anhydrous sodium sulfate, the drying agent was filtered off, and the solvent was carefully removed under reduced pressure (100 mbar, 36 °C, ca. 10 min). The residue was then purified by distillation under high vacuum ($2.5 \cdot 10^{-2}$ mbar, 65 °C) through a short-path connection into a receiving flask cooled to −78 °C, leaving behind an orange solid (dibenzylideneacetone). The colorless condensate was further purified by flash chromatography (silica; pentane/diethyl ether, 6:1) to furnish the title compound as a colorless liquid after careful evaporation of the solvents at 150 mbar and 36 °C (998 mg, 56% yield). $[\alpha]_D^{20} = -33.8$ (c = 1.5, $CHCl_3$). ^1H NMR (400 MHz, $CDCl_3$): δ = 7.01 (ddd, *J* = 10.0, 5.7, 2.5 Hz, 1H), 6.04 (ddd, *J* = 10.0, 2.8, 1.1 Hz, 1H), 4.84 – 4.81 (m, 1H), 4.79 – 4.76 (m, 1H), 2.78 – 2.76 (m, 1H), 2.61 – 2.43 (m, 2H), 2.38 – 2.25 (m, 2H), 1.77 – 1.75 (m, 3H); ^{13}C NMR (101 MHz, $CDCl_3$): δ = 199.8, 149.8, 146.6, 129.7, 110.9, 43.2, 42.2, 31.1, 20.6; IR (film) ν/cm^{-1}: 2968, 1677, 1430, 1388, 1246, 892, 738; HRMS (GC-EI): calcd. for $C_9H_{12}O$ $[M]^+$: 136.0883; found: 136.0883.

The analytical data are in agreement with those reported in the literature for the enantiomer.[26]

As a minor modification of the literature route to compound **21** reported in ref. 2, the ozonolysis step was replaced by a catalytic dihydroxylation followed by periodate cleavage. This modified procedure is described below. All other steps followed exactly the literature.

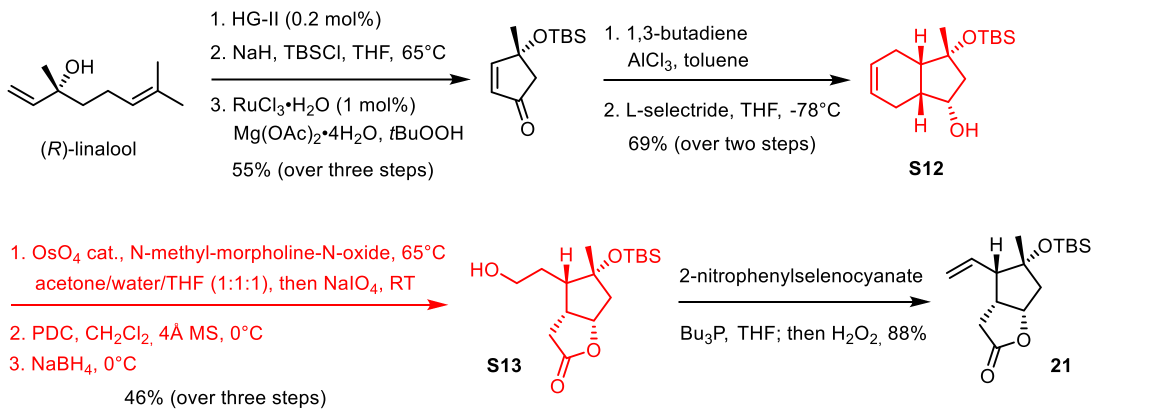

Compound S13. N-Methylmorpholine N-oxide (2.5 g, 21 mmol) and OsO_4 (4% *w/w* in water, 134 µL, 21 µmol) were added to a suspension of (1*S*,3*R*,3a*S*,7a*R*)-3-((*tert*-butyldimethylsilyl)oxy)-3-methyl-2,3,3a,4,7,7a-hexahydro-1*H*-inden-1-ol (**S12**) (2.0 g, 7 mmol)[2] in acetone/THF/water (12 mL each). The mixture was stirred at 65°C for 65 h and then cooled to room temperature. $NaIO_4$ (7.5 g, 35 mmol) was added and stirring continued at ambient temperature for 20 min. For work-up, the mixture was cooled to 0 °C before sat. aq. sodium thiosulfate (20 mL) was introduced. The aqueous phase was extracted with EtOAc (3 x 100 mL) and the combined organic layers were washed with brine (20 mL), dried over $MgSO_4$, filtered, and concentrated under reduced pressure. The residue was purified by flash

chromatography, eluting with a gradient of 0-30% EtOAc in hexanes to afford an unstable colorless oil, which was immediately used in the next step.

MS 4 Å (3.8 g) and PDC (3.7 g, 9.8 mmol) were successively added to a solution of this compound in CH_2Cl_2 (30 mL) at 0 °C. The mixture was stirred at this temperature for 1 h before it was quickly passed through a pad of silica, eluting with hexane:acetone (1:0 to 2:1).

Evaporation of the combined filtrates gave a yellow oil, which was dissolved in THF (25 mL). $NaBH_4$ (400 mg, 10.6 mmol) was added in portions to this solution at 0 °C and the mixture was stirred at this temperature for 15 min before the reaction was quenched with sat. NH_4Cl (50 mL). The resulting mixture was extracted EtOAc (3 x 200 mL), the combined organic layers were washed with brine (50 mL), dried with $MgSO_4$, filtered, and evaporated. The residue was purified by flash chromatography on silica gel (hexanes:acetone, 4:1 to 1:1) to afford the title compound as a white solid (1.03 g, 46% over 3 steps).

The analytical and spectroscopic data fully matched the literature.[2]

Lactone 22. In a plastic vial, HF·pyr (70% *w/w*, 11 mL) was added to a solution of silyl ether **21** (804 mg, 2.70 mmol)[2] in THF (11 mL) at 0 °C. The mixture was stirred at room temperature for 6 h before it was poured into a saturated aqueous solution of $NaHCO_3$ (100 mL) at 0 °C. The aqueous phase was extracted with EtOAc (5 x 100 mL), the combined organic layers were washed with brine (15 mL), dried over $MgSO_4$, filtered and concentrated. The residue was purified by flash chromatography on silica gel, eluting with a gradient of 0-40% EtOAc in hexanes, to afford the corresponding alcohol as a yellow solid.

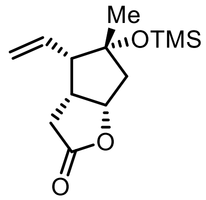

Triethylamine (1.2 mL, 8.3 mmol) was added to a solution of this product in THF (11 mL). The mixture was cooled to 0 °C before trimethylsilyl trifluoromethanesulfonate (1.1 mL, 6.1 mmol) was added and stirring continued at room temperature for 2 h. The mixture was cooled to 0 °C before TBAF (1 M in THF, 5.6 mL, 5.6 mmol) was added, and the resulting mixture stirred at that temperature *for 20 seconds*. The reaction was instantly quenched with saturated aqueous NH_4Cl (20 mL), the mixture was extracted with EtOAc (3 x 50 mL), the combined organic layers were washed with brine (10 mL), dried over $MgSO_4$, filtered and concentrated under reduced pressure. The residue was purified by flash chromatography on silica, eluting with a gradient of 0-20% EtOAc in hexanes, to afford the title compound as a yellow oil (614 mg, 84% yield over two steps). $[\alpha]_D^{20}$ = −12.6 (c = 1.08, $CHCl_3$). ^1H NMR (400 MHz, $CDCl_3$): δ = 5.93 (ddd, *J* = 17.3, 10.4, 8.7 Hz, 1H), 5.25 (ddd, *J* = 10.4, 2.1, 0.6 Hz, 1H), 5.14 (ddd, *J* = 17.2, 2.0, 0.9 Hz, 1H), 5.04 (dd, *J* = 7.9, 6.8 Hz, 1H), 3.15–3.06 (m, 1H), 2.82 (dd, *J* = 18.6, 4.8 Hz, 1H), 2.44 (dd, *J* = 18.6, 12.0 Hz, 1H), 2.30 (d, *J* = 15.0 Hz, 1H), 2.23 (t, *J* = 8.7 Hz, 1H), 1.74 (ddd, *J* = 15.0, 6.9, 0.6 Hz, 1H), 1.28 (s, 3H), 0.11 (s, 9H); ^{13}C NMR (101 MHz, $CDCl_3$): δ = 178.2, 134.3, 119.0, 84.0, 83.1, 57.2, 47.3, 42.7, 30.8, 25.1, 2.1; IR (film) ν/cm^{-1}: 3077, 2959, 2898, 1766, 1638, 1453, 1415, 1377, 1364, 1321, 1251, 1194, 1172, 1112, 1005, 890, 841, 754; HRMS (GC-EI): calcd for $C_{13}H_{22}O_3Si$ $[M]^+$: 254.13327; found: 254.13286.

Alkenyl Iodide 23. O_3 was bubbled through a solution of alkene **22** (705 mg, 2.77 mmol) in dichloromethane (25 mL) at −78 °C for 10 min until a deep blue color persisted. The mixture was warmed to 0 °C, argon was bubbled through it for 15 min, followed by addition of triphenylphosphine (2.2 g, 8.4 mmol). The mixture was stirred at room temperature for 3 h before it was concentrated under reduced pressure and the residue purified by flash chromatography on silica, eluting with a gradient of 0-40% EtOAc in hexanes, to afford the corresponding aldehyde as a yellow oil, which was used in the next step without delay.

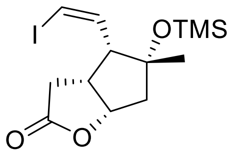

Sodium bis(trimethylsilyl)amide (NaHMDS, 575 mg, 3.13 mmol) was added to a suspension of [PPh_3CH_2I]I (1.80 g, 3.40 mmol) in THF (25 mL), the resulting mixture was stirred at room temperature for 15 min and then cooled to −78 °C. A solution of the aldehyde in THF (5 mL) was added dropwise over 4 min (rinsing the flask with THF (5 x 2 mL)) at −78 °C and stirring continued at this temperature for 30 min. Saturated aqueous NH_4Cl (20 mL) was added, the mixture was warmed to room temperature, extracted with *tert*-butyl methyl ether (3 x 50 mL), the combined organic layers were washed with brine (20 mL), dried over $MgSO_4$, filtered and concentrated. The residue was purified by flash chromatography on silica, eluting with a gradient of 0-20% *tert*-butyl methyl ether in hexanes, to afford the title compound as a yellow oil (730 mg, 69% yield over two steps). $[\alpha]_D^{20}$ = +76.9 (c = 1.09, $CHCl_3$). ^{1}H NMR (400 MHz, $CDCl_3$) δ = 6.53 (dd, *J* = 7.5, 0.7 Hz, 1H), 6.47 (t, *J* = 7.9 Hz, 1H), 5.09 (dd, *J* = 7.9, 6.6 Hz, 1H), 3.41 – 3.31 (m, 1H), 2.69 – 2.57 (m, 2H), 2.46 (dd, *J* = 18.6, 11.9 Hz, 1H), 2.36 (d, *J* = 15.1 Hz, 1H), 1.82 (ddd, *J* = 15.1, 6.6, 0.5 Hz, 1H), 1.30 (s, 3H), 0.13 (s, 9H); ^{13}C NMR (101 MHz, $CDCl_3$) δ = 177.8, 138.1, 85.9, 84.0, 83.3, 57.9, 47.0, 40.6, 31.1, 25.4, 2.1; IR (film) ν/cm^{-1}: 2958, 2897, 1766, 1608, 1450, 1414, 1377, 1366, 1301, 1252, 1190, 1174, 1113, 1004, 891, 840, 754; HRMS (ESI): calcd for $C_{13}H_{21}IO_3SiNa$ [M+Na]$^+$: 403.01969; found: 403.02000.

Intelligence Gathering

Note: The reactions in the pathfinding studies and failed attempts (Schemes S1-S3) have not been optimized.

Compound S4. 2,2,6,6-Tetramethylpiperidine (189 µL, 1.12 mmol) was added to a solution of lactone

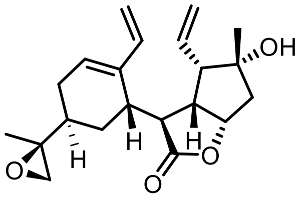

22 (117.3 mg, 0.461 mmol) in dichloromethane (2 mL). The mixture was cooled to 0 °C before triisopropylsilyl trifluormethanesulfonate (170 µL, 0.633 mmol) was added. The mixture was warmed to room temperature and stirred for 4 h. A solution of aldehyde **S2** (360 mg, 1.47 mmol)[22] in CH_2Cl_2 (1.0 mL) was added, followed by La(OTf)$_3$ (18 mg, 0.03 mmol). The resulting mixture was stirred at that temperature for 24 h before it was quenched with HCl (2 M, 5 mL). After stirring for 10 min, the aqueous phase was extracted with EtOAc (5 mL x 3), the combined extracts were washed with brine (2 mL), dried over Na_2SO_4, and filtered. After evaporation of the solvent, the residue was quickly passed through a short plug silica, eluting with acetone:hexanes (1:3), to provide a rather unstable colorless oil.

tert-BuOK (130 mg, 1.16 mmol) was added to a solution of methyltriphenylphosphonium bromide (418 mg, 1.17 mmol) in THF (5 mL) at 0°C and the resulting mixture was stirred for 30 min. A solution of the crude aldehyde obtained in the previous step in THF (0.8 mL) was added. Stirring was continued at 0°C for 15 min and then at room temperature for another 12 h. Water (0.1 mL) was added to quench the reaction. All volatiles were removed and the crude material was purified by flash chromatography on silica (hexane/acetone 4:1) to afford the title compound as a colorless oil (26 mg, 16% over two steps). ^{1}H NMR (400 MHz, $CDCl_3$): δ = 6.27 (dd, *J* = 17.7, 11.0 Hz, 1H), 6.06–5.86 (m, 2H), 5.31 (dd, *J* = 10.2, 2.2 Hz, 1H), 5.24–5.09 (m, 2H), 4.98 (d, *J* = 11.0 Hz, 1H), 4.89 (dd, *J* = 7.6, 6.1 Hz, 1H), 3.04 (t, J = 3.5 Hz, 1H), 2.96 (ddd, J = 9.2, 7.6, 3.7 Hz, 2H), 2.67–2.55 (m, 1H), 2.53 (d, J = 4.7 Hz, 1H), 2.39 (t, J = 9.4 Hz, 1H), 2.28–2.09 (m, 4H), 1.98–1.85 (m, 2H), 1.84–1.77 (m, 1H), 1.77 –1.71 (m, 1H), 1.25 (s, 3H), 1.22 (s, 3H) ppm; ^{13}C NMR (101 MHz, $CDCl_3$): δ = 180.4, 138.3, 136.3, 133.6, 130.4, 120.6, 112.4, 83.1, 80.5, 58.9, 56.6, 53.0, 47.6, 47.4, 45.4, 36.4, 34.2, 29.6, 29.4, 27.6, 25.6, 18.6 ppm; MS (ESI)*:* 367 [M+Na$^+$]; HRMS (ESI): *m/z:* calcd. for $C_{21}H_{28}O_4Na$ [*M*+Na$^+$]: 367.18798, found: 367.18758.

Compound 11. To a solution of compound **S4** (22.4 mg, 0.065 mmol) in 1,4-dioxane and MeOH (1.2 mL, *v/v* = 1:1) were added zinc dust (85 mg, 1.30 mmol), $CuSO_4$ (10.0 mg, 0.0627 mmol), NaI (230 mg, 1.53 mmol), NaOAc (48.0 mg, 0.585 mmol), and HOAc (0.1 mL, 96 mmol). The resulting suspension was stirred at room temperature for 12 h before it was filtered through a pad of Celite, rinsing with EtOAc (15 mL). The combined filtrates were washed with sat. aq. $NaHCO_3$ (2 mL) and brine (2 mL), dried over Na_2SO_4 and evaporated. The residue purified by flash chromatography on silica (hexane:acetone, 7:1 to 5:1) to afford the title compound as a colorless oil (20.0 mg, 94%). ^{1}H NMR (400 MHz, $CDCl_3$): δ = 6.27 (dd, *J* = 17.6, 11.0 Hz, 1H), 6.08–5.85 (m, 2H), 5.30 (dd, *J* = 10.3, 2.1 Hz, 1H), 5.25–5.08 (m, 2H), 4.97 (d, *J* = 11.0 Hz, 1H), 4.86 (dd, *J* = 7.4, 6.1 Hz, 1H), 4.75–4.63 (m, 2H), 3.11–2.88 (m, 3H), 2.52–2.21 (m, 3H), 2.17 (d, *J* = 15.0 Hz, 1H), 2.04–1.87 (m, 2H), 1.81 (dd, *J* = 15.0, 6.2 Hz, 1H), 1.76–1.65 (m, 4H), 1.22 (s, 3H) ppm; ^{13}C NMR (101 MHz, $CDCl_3$): δ = 180.8, 149.2, 138.4, 136.1, 133.7, 131.0, 120.5, 112.5, 109.2, 83.2, 80.5, 56.6, 47.4, 47.3, 45.9, 37.1, 35.5, 33.1, 31.1, 25.6, 20.7 ppm; MS (ESI)*:* 351 [M+Na$^+$]; HRMS (ESI): *m/z:* calcd. for $C_{21}H_{28}O_3Na$ [*M*+Na$^+$]: 351.19306, found: 351.19329.

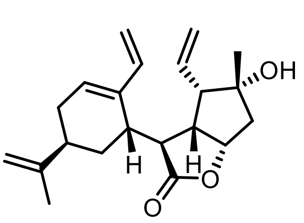

Compound 12. Second-generation Hoveyda-Grubbs catalyst (3.8 mg, 0.0061 mmol) was added to a solution of compound **11** (20.0 mg, 0.0609 mmol) in toluene (12.0 mL). The mixture was stirred at 100 °C (bath temperature) for 5 h. After cooling to ambient temperature, the solvent was removed under vacuum, and the residue was purified by flash chromatography on silica (hexanes:acetone, 10:1 to 8:1) to afford the title compound as a colorless oil (12.6 mg, 69%). ^{1}H NMR (400 MHz, $CDCl_3$): δ = 6.26–6.16 (m, 1H), 6.00–5.86 (m, 1H), 5.28 (dd, *J* = 12.8, 4.9 Hz, 1H), 4.91 (dt, *J* = 8.0, 6.0 Hz, 1H), 4.82 (q, *J* = 1.6 Hz, 1H), 4.60 (dt, *J* = 2.0, 1.0 Hz, 1H), 3.52 (t, *J* = 10.7 Hz, 1H), 2.97 (ddd, *J* = 11.0, 8.0, 6.6 Hz, 1H), 2.77–2.69 (m, 1H), 2.62–2.51 (m, 1H), 2.49–2.38 (m, 3H), 2.34–2.25 (m, 2H), 2.26–2.18 (m, 2H), 1.80–1.65 (m, 4H), 1.37 (s, 3H) ppm; ^{13}C NMR (101 MHz, $CDCl_3$): δ = 177.4, 147.8, 138.7, 137.4, 135.5, 118.3, 109.8, 81.1, 79.2, 52.6, 51.9, 47.1, 42.5, 37.7, 37.0, 30.4, 28.8, 28.2, 22.3 ppm; HRMS (ESI): calcd for $C_{19}H_{24}O_3Na$ [M+Na]$^+$: 323.16176; found: 323.16199.

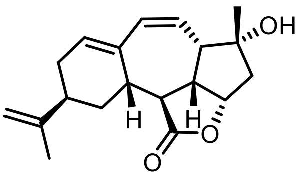

Compound S5. *n*-Butyllithium (1.6 M in hexanes, 7.0 mL, 11.1 mmol) was added to a solution of PPh_3MeBr (4.24 g, 11.9 mmol) in THF (12 mL) at 0 °C. The resulting mixture was stirred for 5 min at this temperature and for 1 h at room temperature before it was cooled to 0 °C. A solution of aldehyde **S2** (2.0 g, 7.91 mmol)[22] in THF (12 mL) was added dropwise and stirring continued at that temperature for 1 h. Sat. aq. NH_4Cl (10 mL) was introduced, the mixture was extracted with hexanes (3 x 100 mL), the combined organic layers were washed with brine (10 mL), dried over $MgSO_4$, filtered and concentrated under reduced pressure. The residue was triturated with hexanes, the remaining precipitate was filtered off, and the solution was concentrated. The residue was purified by flash chromatography on silica, eluting with a gradient of 0-5% *tert*-butyl methyl ether in hexanes to afford a colorless oil.

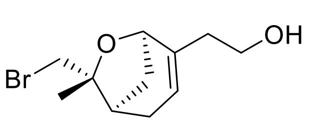

A solution of 9-BBN (0.5 M in THF, 9.0 mL, 4.5 mmol) was added dropwise over 5 min to a solution of that product in THF (42 mL) at 0 °C. The resulting mixture was stirred at that temperature for 5 min and then for 4 h at ambinent temperature. The mixture was cooled to 0 °C before water (1.0 mL) and $NaBO_3 \cdot H_2O$ (2.66 g, 13.3 mmol) were slowly added. After stirring for 17 h at room temperature, the mixture was poured into a sat. aq. sodium thiosulfate (100 mL) at 0 °C. The aqueous layer was extracted with EtOAc (3 x 100 mL), the combined organics phases were washed with brine, dried over $MgSO_4$, filtered and concentrated. The residue was purified by flash chromatography on silica, eluting with a gradient of 0-50% EtOAc in hexanes to afford the title compound as a colorless oil (815 mg, 39%

over two steps). $[\alpha]_D^{20}$ = −2.2 (c = 0.92, $CHCl_3$). ^1H NMR (400 MHz, $CDCl_3$) δ = 5.43 (dhept, *J* = 3.8, 1.3 Hz, 1H), 4.26 (dd, *J* = 4.9, 1.3 Hz, 1H), 3.80 (dtd, *J* = 11.2, 7.0, 5.0 Hz, 1H), 3.64 (dtd, *J* = 11.4, 6.4, 5.1 Hz, 1H), 3.50 (s, 3H), 2.58 – 2.16 (m, 6H), 1.97 (d, *J* = 10.8 Hz, 1H), 1.40 (s, 3H). ^{13}C NMR (101 MHz, $CDCl_3$) δ = 141.1, 124.4, 85.4, 76.4, 61.6, 41.1, 39.3, 38.7, 34.8, 29.8, 26.5. IR (film) ν/cm^{-1}: 3418, 2945, 2889, 2847, 1430, 1373, 1298, 1228, 1091, 1050, 1004, 926, 904, 644. HRMS (GC-EI): calcd. for $C_{11}H_{17}O_2Br$ $[M]^+$: 260.04066; found: 260.04068.

Compound S6. Tributylphosphine (2.3 mL, 9.36 mmol) was added to a suspension of *N*-(phenylseleno)-

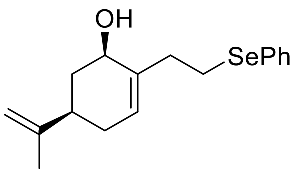

phtalimide (2.8 g, 9.36 mmol) in THF (6 mL). The mixture was stirred at room temperature for 5 min and then cooled to 0 °C before alcohol **S5** (815 mg, 3.12 mmol) was added. Stirring was continued at room temperature for 17 h. The mixture was concentrated under a stream of argon and the residue purified by flash chromatography on silica, eluting with 0-6% acetone in hexanes to afford the desired selenide as a yellowish oil, which was directly used in the next step.

Zn powder (4.0 g, 61.2 mmol) and NH_4Cl (1.6 g, 29.9 mmol) were added to a solution of this compound in EtOH (10 mL). The resulting mixture was stirred at reflux temperature for 150 min. The suspension was filtered through a pad of Celite®, rinsing with EtOAc (5 x 20 mL), and the combined filtrates were concentrated under reduced pressure. The residue was purified by flash chromatography on silica, eluting with a gradient of 0-30% *tert*-butyl methyl ether in hexanes to afford the title compound as a colorless oil (670 mg, 67% over two steps). $[\alpha]_D^{20}$ = −34.6 (c = 0.6, $CHCl_3$). ^1H NMR (400 MHz, $CDCl_3$) δ = 7.56 – 7.47 (m, 2H), 7.33 – 7.19 (m, 3H), 5.63 – 5.55 (m, 1H), 4.79 – 4.71 (m, 2H), 4.30 (s, 1H), 3.20 – 3.00 (m, 2H), 2.81 – 2.67 (m, 1H), 2.51 – 2.37 (m, 1H), 2.32 – 2.20 (m, 1H), 2.19 – 2.09 (m, 2H), 2.05 – 1.92 (m, 1H), 1.76 (s, 3H), 1.63 – 1.43 (m, 2H). ^{13}C NMR (101 MHz, $CDCl_3$) δ = 148.8, 139.0, 132.4, 130.6, 129.0, 126.7, 125.1, 109.3, 69.2, 40.1, 38.1, 33.5, 31.0, 26.6, 20.7. IR (film) ν/cm^{-1}: 3330, 3070, 2920, 2855, 1644, 1579, 1477, 1437, 1375, 1259, 1228, 1073, 1023, 917, 890, 735, 691. HRMS (GC-EI): calcd. for $C_{17}H_{22}OSe$ $[M]^+$: 322.08303; found: 322.08301.

Compound S7. MnO_2 (3.6 g, 41.4 mmol) was added to a solution of alcohol **S6** (670 mg, 2.1 mmol) in

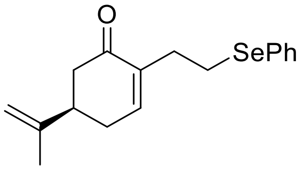

dichloromethane (10 mL) and the resulting suspension was stirred at room temperature for 17 h. The mixture was filtered through a pad of Celite®, rinsing with EtOAc (5 x 10 mL), and the combined filtrates were concentrated under reduced pressure to give the title compound as a yellow oil (627 mg, 94% yield). $[\alpha]_D^{20}$ = −16.9 (c = 0.8, $CHCl_3$). ^1H NMR (400 MHz, $CDCl_3$) δ = 7.52 – 7.47 (m, 2H), 7.30 – 7.20 (m, 3H) 6.77 (ddt, *J* = 6.1, 2.4, 1.1 Hz, 1H), 4.85 – 4.78 (m, 1H), 4.75 (s, 1H), 3.15 – 2.99 (m, 2H), 2.64 (t, *J* = 7.6 Hz, 2H), 2.60 – 2.55 (m, 1H), 2.52 (ddd, *J* = 15.8, 3.8, 1.7 Hz, 1H), 2.44 (dt, *J* = 18.4, 5.2 Hz, 1H), 2.30 – 2.21 (m, 2H), 1.75 (s, 3H). ^{13}C NMR (101 MHz, $CDCl_3$) δ = 199.0, 146.6, 146.2, 137.7, 132.3, 132.2, 132.2, 130.5, 129.1, 126.6, 110.6, 43.2, 42.1, 31.2, 31.2, 26.4, 20.5. IR (film) ν/cm^{-1}: 3071, 2968, 2933, 1672, 1579, 1478, 1436, 1378, 1243, 1133, 1023, 897, 737, 691. HRMS (ESI): calcd for $C_{17}H_{20}OSeNa$ $[M+Na]^+$: 343.05716; found: 343.05715.

Triene 13. 2,2,6,6-Tetramethylpiperidine (189 µL, 1.12 mmol) was added to a solution of lactone

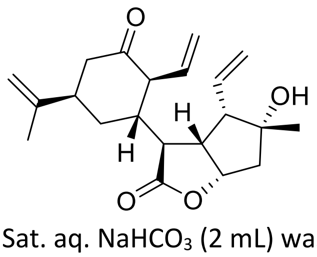

22 (94.8 mg, 0.37 mmol) in dichloromethane (2 mL). The mixture was cooled to 0 °C before triisopropylsilyl trifluormethanesulfonate (200 µL, 0.74 mmol) was added. The mixture was warmed to room temperature and stirred for 4 h. Selenide **S7** (178 mg, 0.56 mmol) was added and the mixture was cooled to 0 °C before $La(OTf)_3$ (44 mg, 0.073 mmol) was introduced. The resulting mixture was stirred at that temperature for 1 h. Sat. aq. $NaHCO_3$ (2 mL) was added, the aqueous phase was extracted with *tert*-butyl methyl ether (3 x

5 mL), the combined organic layers were washed with brine (5 mL), dried over $MgSO_4$, filtered and concentrated under reduced pressure. The residue was triturated with hexanes (10 mL), the remaining precipitate was filtered off, and the solution concentrated under reduced pressure to give an orange oil.

A solution of TBAF (1 M in THF, 2 mL, 2 mmol) was added to a solution of this crude product in THF (2 mL) at 0 °C and the resulting mixture was stirred at room temperature for 2 h. Sat. aq. NH_4Cl (5 mL) was introduced at 0 °C, the aqueous phase was extracted with EtOAc (3 x 10 mL), the combined organic layers were washed with brine (5 mL), dried over $MgSO_4$, filtered and concentrated under reduced pressure. The residue was purified by silica gel chromatography, eluting with 15% EtOAc in hexanes to afford product **S8** as a yellow oil; for the limited stability, the compound was immediately used without further characterization.

In a round bottomed-flask under air, H_2O (1.5 mL) and $NaIO_4$ (347 mg, 1.6 mmol) were successively added to a solution of **S8** in THF (3 mL). The resulting mixture was stirred at room temperature for 17 h before sat. aq. sodium thiosulfate (3 mL) was introduced. The aqueous phase was extracted with EtOAc (3 x 20 mL), the combined organic layers were washed with brine (10 mL), dried over $MgSO_4$, filtered and concentrated under reduced pressure. The residue was purified by flash chromatography, on silica, eluting with a gradient of 0-15% EtOAc in toluene to afford the title compound as a yellow gum (40 mg, 32% over 3 steps). $[\alpha]_D^{20}$ = –10.3 (c = 1.02, $CHCl_3$). ^1H NMR (600 MHz, CD_2Cl_2) δ = 5.83 (ddd, *J* = 17.1, 10.3, 9.1 Hz, 1H), 5.65 (ddd, *J* = 17.3, 10.3, 9.1 Hz, 1H), 5.31 (ddd, *J* = 10.2, 2.0, 0.5 Hz, 1H), 5.28 (ddd, *J* = 10.3, 2.0, 0.5 Hz, 1H), 5.19 (ddd, *J* = 16.9, 1.9, 0.8 Hz, 1H), 5.16 (ddd, *J* = 17.2, 1.9, 0.7 Hz, 1H), 4.93 (ddd, *J* = 7.9, 6.8, 0.5 Hz, 1H), 4.89 (h, *J* = 1.4 Hz, 1H), 4.68 (dp, *J* = 1.7, 0.8 Hz, 1H), 3.41 (dd, *J* = 11.3, 8.8 Hz, 1H), 3.19 (dd, *J* = 4.9, 3.0 Hz, 1H), 2.81 (h, *J* = 4.6 Hz, 1H), 2.71 (td, *J* = 8.3, 4.9 Hz, 1H), 2.61 (ddd, *J* = 14.8, 3.4, 2.0 Hz, 1H), 2.53 (ddd, *J* = 14.8, 6.3, 1.0 Hz, 1H), 2.40 (t, *J* = 8.9 Hz, 1H), 2.13 (d, *J* = 15.2 Hz, 1H), 1.99 (dddd, *J* = 13.3, 4.1, 3.1, 2.0 Hz, 1H), 1.88 (ddd, *J* = 15.2, 6.7, 0.5 Hz, 1H), 1.83 (tt, *J* = 11.8, 3.0 Hz, 1H), 1.77 (dd, *J* = 13.2, 4.5 Hz, 1H), 1.75 (dt, *J* = 1.5, 0.7 Hz, 3H), 1.22 (s, 3H). ^{13}C NMR (151 MHz, CD_2Cl_2) δ = 210.1, 178.7, 146.6, 135.0, 133.3, 120.5, 120.3, 113.0, 82.5, 80.7, 58.2, 56.4, 49.0, 47.8, 44.8, 43.3, 41.2, 40.5, 28.6, 25.7, 22.3. IR (film) ν/cm^{-1}: 3476, 3077, 2964, 2928, 1749, 1707, 1643, 1452, 1375, 1192, 1116, 1018, 923. HRMS (ESI): calcd for $C_{21}H_{28}O_4Na$ $[M+Na]^+$: 367.18798; found: 367.18791.

Fragment Coupling

Compound 25. 2,2,6,6-Tetramethylpiperidine (2.0 mL, 11.9 mmol) was added to a solution of lactone **23** (730 mg, 1.92 mmol) in dichloromethane (10 mL). The mixture was cooled to 0 °C before triisopropylsilyl trifluormethansulfonate (2.0 mL, 7.5 mmol) was added. The mixture was warmed to room temperature and stirred for 4 h. $La(OTf)_3$ (225 mg, 0.381 mmol) and *(R)*-norcarvone (**19**) (650 mg, 4.80 mmol) were successively added and stirring continued at room temperature for 17 h. The mixture was filtered through a pad of Celite®, rinsing with dichloromethane (5 x 10 mL), and the combined filtrates were concentrated under reduced pressure. The residue was triturated with hexanes (20 mL), the resulting precipitate was filtered off, and the remaining solution was concentrated under reduced pressure. The crude product was purified by flash chromatography on silica, eluting with a gradient of 0-3% *tert*-butyl methyl ether in hexanes, to afford the silyl enol ether **24** as a grey solid.

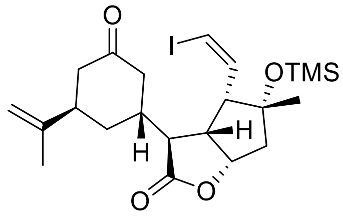

TBAF (1 M in THF, 3.0 mL, 3.0 mmol) was added dropwise over 5 min to a solution of this product in THF (10 mL) at – 78 °C. The mixture was stirred at this temperature for 1 h before saturated aqueous NH_4Cl (10 mL) was added and the mixture warmed to room temperature. The mixture was extracted with EtOAc (3 x 40 mL), the combined organic layers were washed with brine (20 mL), dried over $MgSO_4$, filtered and concentrated under reduced pressure. The residue was purified by flash chromatography on silica, eluting with a gradient of 0-20% *tert*-butyl methyl ether in hexanes, to give the title compound as a white solid (695 mg, 70% yield over two steps). $[\alpha]_D^{20}$ = +142.8 (c = 1.64, $CHCl_3$). ^1H NMR (600 MHz, $CDCl_3$) δ = 6.57 (dd, *J* = 7.5, 0.9 Hz, 1H), 6.46 (dd, *J* = 8.0, 7.5 Hz, 1H), 4.96 (dd, *J* = 7.9, 6.5 Hz, 1H), 4.89 (p, *J* = 1.2 Hz, 1H), 4.66 (dt, *J* = 1.6, 0.8 Hz, 1H), 3.04 (td, *J* = 8.5, 4.5 Hz, 1H), 2.79 (p, *J* = 4.8 Hz, 1H), 2.64 (td, *J* = 8.5, 0.9 Hz, 1H), 2.60 (t, *J* = 4.5 Hz, 1H), 2.59 (ddt, *J* = 15.0, 3.5, 1.9 Hz, 1H), 2.51 (ddd, *J* = 15.1, 12.2, 1.0 Hz, 1H), 2.44 (ddd, *J* = 15.2, 6.2, 1.0 Hz, 1H), 2.34 (d, *J* = 15.0 Hz, 1H), 2.30 (ddt, *J* = 15.0, 4.1, 2.0 Hz, 1H), 2.09 (tq, *J* = 12.1, 4.1 Hz, 1H), 1.97 (dtt, *J* = 13.6, 3.9, 1.9 Hz, 1H), 1.81 (ddd, *J* = 15.1, 6.7, 0.5 Hz, 1H), 1.74 (dt, *J* = 1.4, 0.7 Hz, 3H), 1.67 (ddd, *J* = 13.6, 11.9, 4.7 Hz, 1H), 1.29 (s, 3H), 0.12 (s, 9H); ^{13}C NMR (151 MHz, $CDCl_3$) δ = 210.4, 178.0, 145.9, 137.8, 113.3, 87.1, 82.9, 82.6, 58.3, 47.0, 46.4, 45.4, 44.6, 44.6, 40.2, 35.6, 29.5, 25.5, 22.4, 2.2; IR (film) ν/cm^{-1}: 2957, 1757, 1710, 1645, 1449, 1377, 1301, 1260, 1185, 1117, 1003, 902, 842, 754; HRMS (ESI): calcd for $C_{22}H_{33}IO_4SiNa$ $[M+Na]^+$: 539.10851; found: 539.10879.

Completion of the Total Syntheses

Enone 28 and α-Hydroxyketone 30. *Intramolecular Alkenylation/Isomerization:* Potassium *tert*-butoxide (370 mg, 3.31 mmol) was added to a solution of 2,6-diisopropylphenol (717 µL, 3.86 mmol) in toluene (550 mL) at 60 °C (bath temperature). The resulting suspension was stirred at that temperature for 90 min before alkenyl iodide **25** (550 mg, 1.06 mmol) and $Pd(PPh_3)_4$ (255 mg, 0.221 mmol) were successively added. Stirring was continued at 60 °C for 75 min before the mixture was cooled to room temperature and filtered through a pad of silica, rinsing with EtOAc (10 x 50 mL). The combined filtrates were concentrated under reduced pressure and the residue was purified by flash chromatography on silica, eluting with a gradient of 0-30% EtOAc in hexanes, to afford enone **28** as a red solid and a second fraction containing dimer **29** (120 mg, 29% yield) as an orange solid (the data of the dimer are compiled below).

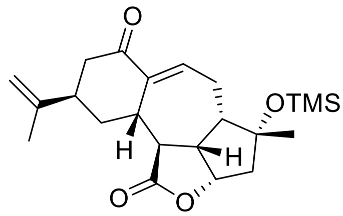

For analytical purposes, an aliquot was purified by HPLC [(150 mm YMC Triart C18 5 µm, 20.0 mm ∅, MeOH in H_2O = 80:20 isocratic over 20 min, 15 mL/min, λ = 210 nm, t = 9.91 min)] to give analytically pure **28**, which analysed as follows: colorless gum; $[\alpha]_D^{20}$ = +3.8 (c = 0.8, $CHCl_3$). ^1H NMR (600 MHz, $CDCl_3$) δ = 6.67 (ddd, *J* = 9.6, 3.9, 1.9 Hz, 1H), 4.86 (td, *J* = 8.3, 1.3 Hz, 1H), 4.83 (s, 1H), 4.70 (s, 1H), 3.60 (dd, *J* = 11.9, 9.8 Hz, 1H), 2.90 (dtd, *J* = 13.7, 5.4, 1.4 Hz, 1H), 2.82 (dt, *J* = 9.7, 8.4 Hz, 1H), 2.76-2.69 (m, 2H), 2.68-2.59 (m, 2H), 2.45 (dd, *J* = 15.6, 5.0 Hz, 1H), 2.35 (ddd, *J* = 15.5, 1.4, 0.6 Hz, 1H), 2.28 (ddd, *J* = 16.0, 9.6, 7.5 Hz, 1H), 2.16 (dt, *J* = 10.0, 7.8 Hz, 1H), 1.91 (dd, *J* = 15.5, 8.1 Hz, 1H), 1.78 (s, 3H), 1.76 (ddd, *J* = 13.7, 11.9, 6.7 Hz, 1H), 1.28 (s, 3H), 0.15 (s, 9H); ^{13}C NMR (151 MHz, $CDCl_3$) δ = 203.3, 177.8, 147.3, 141.8, 134.5, 111.5, 82.7, 80.4, 50.7, 48.6, 47.6, 44.0, 42.1, 40.4, 38.7, 31.2, 26.6, 21.4, 21.3, 2.2; IR (film) ν/cm^{-1}: 2959, 1763, 1695, 1611, 1451, 1377, 1301, 1251, 1232, 1194, 1109, 1011, 890, 841, 754; HRMS (ESI): calcd for $C_{22}H_{32}O_4SiNa$ $[M+Na]^+$: 411.19620; found: 411.19654.

Hydroxylation: 1,8-Diazabicyclo[5.4.0]undec-7-ene (320 µL, 2.13 mmol) was added to a solution of trimethyl phosphite (180 µL, 1.60 mmol) in acetonitrile (10 mL). The solution was purged with O_2 for 40 min. This solution was then transferred via canula into the reaction flask that had been charged with the crude enone **28** and purged with O_2 for 30 min. The resulting mixture was stirred at room temperature under O_2 atmosphere (balloon) for 70 h. The mixture was poured into a saturated aqueous solution of $NaHCO_3$ (40 mL) at 0 °C, which was extracted with EtOAc (3 x 70 mL). The combined organic layers were washed with brine (10 mL), dried over $MgSO_4$, filtered and concentrated under reduced pressure. The residue was purified by flash chromatography on silica, eluting with a gradient of 0-20% EtOAc in hexanes, to afford the desired product **30** as a yellowish gum (170 mg, 39% yield over two steps). $[\alpha]_D^{20}$ = +35.5 (c = 0.79, $CHCl_3$). ^{1}H NMR (600 MHz, CD_2Cl_2) δ = 5.90 (ddd, *J* = 11.6, 2.3, 0.6 Hz, 1H), 5.60 (dd, *J* = 11.7, 2.6 Hz, 1H), 4.88 (q, *J* = 1.5 Hz, 1H), 4.86 (td, *J* = 8.7, 2.0 Hz, 1H), 4.69 (dp, *J* = 1.7, 0.8 Hz, 1H), 4.39 (s, 1H), 4.09 (ddd, *J* = 11.2, 8.7, 7.2 Hz, 1H), 3.60 (dd, *J* = 11.2, 10.4 Hz, 1H), 2.85 – 2.82 (m, 2H), 2.81 – 2.76 (m, 1H), 2.68 – 2.61 (m, 1H), 2.61 (dt, *J* = 7.3, 2.5 Hz, 1H), 2.27 (ddd, *J* = 15.8, 2.0, 0.6 Hz, 1H), 2.18 (td, *J* = 10.8, 4.5 Hz, 1H), 2.08 (dd, *J* = 15.8, 8.7 Hz, 1H), 1.80 (dt, *J* = 1.5, 0.7 Hz, 3H), 1.65 (ddd, *J* = 14.8, 11.3, 4.0 Hz, 1H), 1.43 (s, 3H), 0.13 (s, 9H); ^{13}C NMR (151 MHz, CD_2Cl_2) δ = 212.9, 179.0, 146.2, 134.8, 127.9, 112.5, 84.7, 81.0, 78.6, 48.0, 45.2, 44.7, 43.7, 41.4, 40.5, 32.1, 27.9, 22.1, 2.2; IR (film) ν/cm^{-1}: 3461, 2962, 1761, 1712, 1644, 1449, 1378, 1338, 1263, 1250, 1178, 1142, 1101, 1010, 895, 840, 756; HRMS (ESI): calcd for $C_{22}H_{32}O_5SiNa$ [M+Na]$^+$: 427.19112; found: 427.19102.

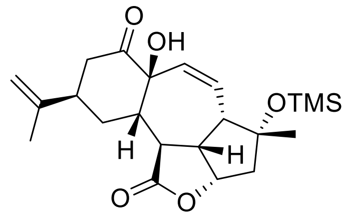

Dimer 29. $[\alpha]_D^{20}$ = +18.8 (c = 0.4, $CHCl_3$). ^{1}H NMR (400 MHz, $CDCl_3$) δ = 6.38 (dd, *J* = 3.3, 2.0 Hz, 1H), 5.90 (dd, *J* = 11.1, 8.2 Hz, 1H), 5.71 (ddd, 1H), 4.94 – 4.85 (m, 3H), 4.85 – 4.83 (m, 1H), 4.70 (s, 1H), 4.66 (s, 1H), 3.86 – 3.78 (m, 1H), 3.56 (dd, *J* = 12.1, 9.5 Hz, 1H), 2.97 – 2.82 (m, 3H), 2.81 - 2.63 (m, 5H), 2.61 – 2.49 (m, 3H), 2.47 – 2.38 (m, 2H), 2.36 – 2.20 (m, 4H), 2.09 (t, *J* = 8.4 Hz, 1H), 1.96 (dd, *J* = 16.0, 8.3 Hz, 1H), 1.92 – 1.85 (m, 1H), 1.83 – 1.77 (m, 5H), 1.75 - 1.69 (m, 4H), 1.33 (s, 3H), 1.31 (s, 3H), 0.15 (s, 9H), 0.13 (s, 9H); ^{13}C NMR (101 MHz, $CDCl_3$) δ = 210.3, 201.7, 178.2, 177.2, 146.9, 146.1, 141.2, 140.5, 139.9, 126.5, 112.9, 111.5, 83.2, 83.1, 82.8, 79.8, 57.7, 51.5, 48.9, 47.1, 47.0, 46.3, 46.1, 44.3, 43.7, 43.2, 42.5, 40.5, 40.0, 38.4, 35.1, 32.5, 30.8, 30.7, 27.9, 25.9, 22.2, 21.0, 2.4, 2.2; IR (film) ν/cm^{-1}: 2954, 1763, 1712, 1603, 1450, 1377, 1320, 1251, 1194, 1117, 1007, 902, 841, 756; HRMS (ESI): calcd for $C_{44}H_{64}NaO_8Si_2$ [M+Na]$^+$: 799.40319; found: 799.40293.

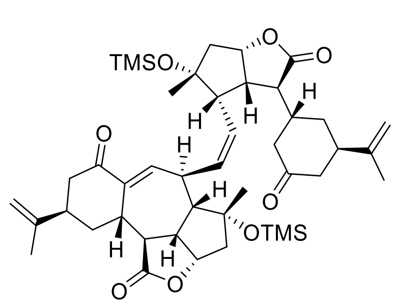

Analytical Data of the Side Products Identified during Optimization of the Protecting Group Strategy (Scheme S3) and the Intramolecular Alkenylation/Isomerization Step (Table S5):

Alkyne 26. ^{1}H NMR (400 MHz, $CDCl_3$) δ = 4.94 – 4.86 (m, 2H), 4.68 (s, 1H), 3.08 (t, *J* = 4.6 Hz, 1H), 2.89 – 2.79 (m, 2H), 2.69 – 2.57 (m, 2H), 2.52 – 2.35 (m, 3H), 2.28 – 2.11 (m, 4H), 1.75 (s, 3H), 1.71 (dd, *J* = 14.7, 6.7 Hz, 2H). 1.44 (s, 3H), 0.13 (s, 9H). ^{13}C NMR (101 MHz, $CDCl_3$) δ = 210.6, 178.1, 146.1, 113.1, 83.1, 82.1, 80.8, 73.8, 48.0, 47.9, 47.0, 46.0, 44.8, 44.7, 40.2, 35.5, 30.0, 29.9, 25.1, 22.3, 2.2. HRMS (ESI): calcd for $C_{22}H_{32}O_4NaSi$ [M+Na]$^+$: 411.19621; found: 411.19635.

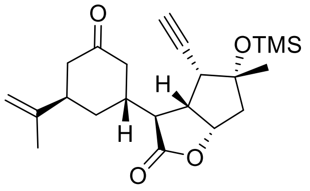

Alkyne S9. ^{1}H NMR (400 MHz, $CDCl_3$) δ = 4.94 (dd, *J* = 7.1, 6.1 Hz, 1H), 4.90 (d, *J* = 1.3 Hz, 1H), 4.68 (dt, *J* = 1.7, 0.8 Hz, 1H), 3.05 (dd, *J* = 5.0, 3.4 Hz, 1H), 2.90 – 2.79 (m, 3H), 2.62 (ddt, *J* = 15.2, 3.6, 1.8 Hz, 1H), 2.54 – 2.43 (m, 3H), 2.40 (d, *J* = 2.1 Hz, 1H), 2.33 (d, *J* = 15.1 Hz, 1H), 2.24 – 2.14 (m, 1H), 2.12 (dtt, *J* = 13.7, 3.8, 2.1 Hz, 1H), 1.84 – 1.69 (m, 6H), 1.39 (s, 3H). ^{13}C NMR (101 MHz, $CDCl_3$) δ = 210.1, 177.6, 145.9, 113.1, 82.0, 80.1, 79.7, 75.8, 48.1, 46.8, 45.8, 45.2, 44.8, 44.5, 40.0, 35.8, 29.8, 25.5, 22.2. HRMS (ESI): calcd for $C_{29}H_{24}O_4Na$ $[M+Na]^+$: 339.15668; found: 339.15660.

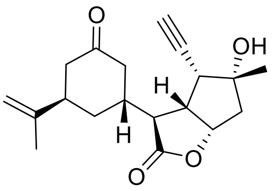

Compound S10. ^{1}H NMR (400 MHz, $CDCl_3$) δ = 5.87 (ddd, *J* = 17.2, 10.3, 9.0 Hz, 1H), 5.28 (dd, *J* = 10.3, 2.1 Hz, 1H), 5.17 (ddd, *J* = 17.3, 2.1, 0.8 Hz, 1H), 4.90 (dd, *J* = 7.7, 6.7 Hz, 1H), 4.87 (s, 1H), 4.65 (s, 1H), 2.82 – 2.74 (m, 3H), 2.59 (ddt, *J* = 15.3, 3.6, 1.8 Hz, 1H), 2.47 – 2.38 (m, 2H), 2.36 – 2.25 (m, 3H), 2.12 (tt, *J* = 7.4, 3.5 Hz, 1H), 2.05 (dtt, *J* = 13.7, 3.8, 1.9 Hz, 1H), 1.78 – 1.67 (m, 5H), 1.27 (s, 3H), 0.10 (s, 9H). ^{13}C NMR (101 MHz, $CDCl_3$) δ = 210.5, 178.4, 146.1, 134.0, 119.9, 113.0, 83.1, 82.6, 57.7, 47.6, 47.4, 46.0, 44.8, 44.7, 44.6, 42.1, 40.4, 40.2, 35.7, 30.9, 29.9, 25.1, 22.3, 21.3, 2.3, 2.2, 1.2. HRMS (ESI): calcd for $C_{22}H_{34}O_4NaSi$ $[M+Na]^+$: 413.21186; found: 413.21210.

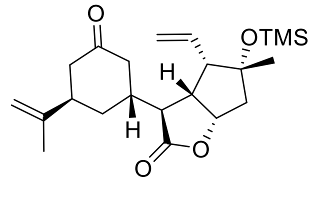

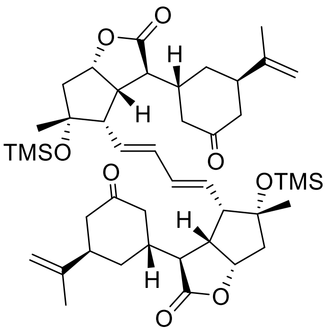

Dimer S11. ^{1}H NMR (400 MHz, $CDCl_3$) δ = 6.44 (dd, *J* = 7.7, 2.2 Hz, 1H), 5.61 (t, *J* = 8.7 Hz, 1H) 4.97 (t, *J* = 6.9 Hz, 1H), 4.82 (q, *J* = 1.2 Hz, 1H), 4.64 (dt, *J* = 1.7, 0.9 Hz, 1H), 2.89 – 2.77 (m, 2H), 2.77 – 2.70 (m, 2H), 2.56 – 2.24 (m, 5H), 2.06 – 1.97 (m, 2H), 1.86 – 1.75 (m, 2H), 1.72 (s, 3H), 1.25 (s, 3H), 0.11 (s, 9H). ^{13}C NMR (101 MHz, $CDCl_3$) δ = 210.5, 177.9, 146.5, 128.6, 127.7, 112.4, 83.8, 82.6, 50.7, 47.9, 47.4, 45.5, 44.9, 44.8, 40.0, 36.5, 30.3, 25.3, 22.1, 2.2. HRMS (ESI): calcd for $C_{44}H_{66}O_8NaSi_2$ $[M+Na]^+$: 801.41884; found: 801.41915.

Allylic alcohol 31. *Note: It proved necessary to perform the reaction in air.* In a round-bottomed flask under air atmosphere methyltrioxorhenium(VII) (3.7 mg, 15 µmol) was added to a solution of compound **30** (15 mg, 37 µmol) in toluene (0.8 mL). The mixture was stirred at 60 °C (bath temperature) for 26 h before it was cooled to room temperature, and filtered through a plug of Celite®, rinsing with EtOAc (5 x 2 mL). The combined filtrates were concentrated under reduced pressure, and the residue was purified by flash chromatography on silica, eluting with 50% EtOAc in hexanes, to give product **31** (6.4 mg, 52% yield) and a second fraction comprised of scabrolide B (**3**) as a white solid (0.9 mg, 7% yield).

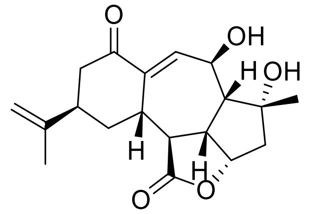

Analytical and spectral data of **31**: $[\alpha]_D^{20}$= –73.6 (c = 0.07, $CHCl_3$). ^{1}H NMR (600 MHz, CD_2Cl_2) δ = 6.37 (dd, *J* = 2.9, 1.9 Hz, 1H), 5.11 (dq, *J* = 10.0, 3.5 Hz, 1H), 4.84 (td, *J* = 1.4, 0.7 Hz, 1H), 4.78 (ddd, *J* = 8.3, 7.6, 6.2 Hz, 1H), 4.69 (tt, *J* = 1.3, 0.7 Hz, 1H), 3.20 (t, *J* = 11.5 Hz, 1H), 2.86 (dtd, *J* = 13.6, 5.5, 1.5 Hz, 1H), 2.83 (dt, *J* = 11.3, 8.6 Hz, 1H), 2.74 – 2.69 (m, 1H), 2.68 (tddd, *J* = 11.8, 5.3, 3.2, 2.0 Hz, 1H), 2.67 – 2.65 (m, 1H) 2.61 (d, *J* = 4.9 Hz, 1H), 2.44 (dd, *J* = 15.1, 4.3 Hz, 1H), 2.28 (t, *J* = 9.5 Hz, 1H), 2.25 (dd, *J* = 14.4, 7.7 Hz, 1H), 2.17 (dd, *J* = 14.4, 6.3 Hz, 1H), 2.14 (s, 1H), 1.81 (ddd, *J* = 13.8, 11.9, 6.7 Hz, 1H), 1.78 (dt, *J* = 1.3, 0.6 Hz, 3H), 1.42 (s, 3H); ^{13}C NMR (151 MHz, CD_2Cl_2) δ = 201.8, 176.9, 147.8, 140.6, 139.2, 111.3, 79.8, 79.5, 67.6, 56.1, 47.4, 47.0, 44.2, 42.6, 40.5, 38.9, 31.5, 31.3, 21.1; IR (film) ν/cm^{-1}: 3423, 2961, 2925, 1760, 1689, 1616, 1450, 1378, 1310, 1240, 1215, 1196, 1111, 1049, 1020, 895, 757; HRMS (ESI): calcd for $C_{19}H_{24}O_5Na$ $[M+Na]^+$: 355.15159; found: 355.15169.

(–)-Scabrolide B ((–)-3). MnO_2 (255 mg, 2.91 mmol) was added to a solution of alcohol **31** (19.0 mg, 57.2 µmol) in dichloromethane (3 mL) and the resulting suspension was stirred at room temperature for 30 min. The mixture was filtered through a short pad of Celite®, rinsing with dichloromethane (5 x 3 mL) and EtOAc (5 x 3 mL). The combined filtrates were concentrated under reduced pressure, and the residue was purified by flash chromatography on silica, eluting with a gradient of 30-40% EtOAc in hexanes, to give scabrolide B (9.6 mg, 51% yield) as a white solid. $[\alpha]_D^{20}$ = –56.4 (c = 0.5, $CHCl_3$), $[\alpha]_D^{25}$ = –33.2 (c = 0.34, MeOH) (lit: $[\alpha]_D^{29}$ = –80.0 (c = 0.33, $CHCl_3$);[1] $[\alpha]_D^{25}$ = –60.0 (c = 0.10, MeOH)[27]). ^{1}H NMR (600 MHz, $CDCl_3$) δ = 6.34 (d, *J* = 2.8 Hz, 1H), 4.98 (ddd, *J* = 9.2, 8.1, 2.3 Hz, 1H), 4.94 (q, *J* = 1.2, 1H), 4.71 (dt, *J* = 1.6, 0.8 Hz, 1H), 3.44 (dd, *J* = 11.0, 10.2 Hz, 1H), 3.30 (dtd, *J* = 13.8, 4.1, 2.3 Hz, 1H), 3.14 (dt, *J* = 10.2, 8.1 Hz, 1H), 2.93 (d, *J* = 8.1 Hz, 1H), 2.90 (ddd, *J* = 16.2, 3.9, 2.3 Hz, 1H), 2.81 (m, 1H), 2.78 (dddd, *J* = 12.3, 11.0, 4.1, 2.8 Hz, 1H), 2.58 (dd, *J* = 16.2, 6.3 Hz, 1H), 2.40 (d, *J* = 2.0 Hz, 1H), 2.36 (dd, *J* = 16.2, 2.3 Hz, 1H), 2.19 (ddd, *J* = 16.2, 9.2, 2.0 Hz, 1H), 1.82 (dt, *J* = 1.2, 0.8 Hz, 3H), 1.71 (ddd, *J* = 13.8, 12.3, 5.2 Hz, 1H), 1.62 (s, 3H); ^{13}C NMR (151 MHz, $CDCl_3$) δ = 202.6, 202.3, 176.0 , 150.9, 146.5, 130.6, 112.8, 81.4, 79.6, 62.5, 47.5, 45.4, 45.4, 45.1, 41.7, 39.0, 30.5, 30.1, 22.0; IR (film) ν/cm^{-1}: 3498, 2964, 2927, 1763, 1697, 1667, 1451, 1376, 1334, 1210, 1191, 1166, 1110, 1054, 1018, 943, 899, 755; HRMS (ESI): calcd for $C_{19}H_{23}O_5$ $[M+H]^+$: 331.15400; found: 331.15415.

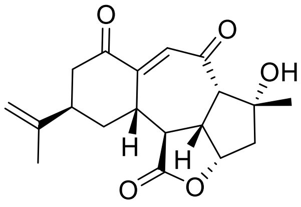

(–)-Sinuscalide C (4). In an MS vial under a flow of Ar, Burgess Reagent (4.3 mg, 18 µmol) was added to a solution of **3** (3.0 mg, 9.1 µmol) in toluene (0.2 mL). The mixture was stirred at 60 °C (bath temperature) for 2 h before it was concentrated under reduced pressure. The residue was purified by flash chromatography on silica, eluting with 30% EtOAc in hexanes, to afford the title compound as a yellowish solid (2.1 mg, 74% yield). $[\alpha]_D^{20}$ = –42.1 (c = 0.2, $CHCl_3$) (lit: $[\alpha]_D^{25}$ = –40.0 (c = 0.10, MeOH).[27,28] ^{1}H NMR (600 MHz, $CDCl_3$) δ = 6.47 (d, *J* = 2.8 Hz, 1H), 5.15 (td, *J* = 8.8, 5.3 Hz, 1H), 4.95 (s, 1H), 4.75 (dt, *J* = 1.6, 0.8 2.6 Hz, 1H), 3.52 (ddp, *J* = 10.5, 8.7, 1.5 Hz, 1H), 3.25 (dtdd, *J* = 13.7, 4.2, 2.5, 0.5 Hz, 1H), 3.14 (ddq, *J* = 20.1, 8.8, 1.2 Hz, 1H), 2.95 (ddd, *J* = 16.0, 3.6, 2.5 Hz, 1H), 2.90 (dddd, *J* = 12.4, 11.3, 4.1, 2.6 Hz, 1H), 2.82 (m, 1H), 2.79 (dddd, *J* = 20.1, 6.6, 3.2, 1.4 Hz, 1H), 2.64 (dd, *J* = 11.3, 10.5 Hz, 1H), 2.60 (dd, *J* = 16.0, 6.4 Hz, 1H), 2.14 (q, *J* = 1.5 Hz, 3H), 1.84 (dt, *J* = 1.4, 0.7 Hz, 3H), 1.72 (ddd, *J* = 13.7, 12.4, 5.0 Hz, 1H); ^{13}C NMR (151 MHz, $CDCl_3$) δ = 202.8, 187.5, 175.7, 153.1, 149.9, 146.2, 134.7, 132.8, 113.1, 77.6, 50.9, 48.8, 46.2, 45.5, 39.7, 38.9, 31.0, 22.0, 16.0; IR (film) ν/cm^{-1}: 2924, 2855, 1770, 1698, 1647, 1611, 1428, 1355, 1212, 1137, 1030, 959, 888; HRMS (GC-EI): calcd for $C_{19}H_{20}O_4$ $[M]^+$: 312.13561; found: 312.13562.

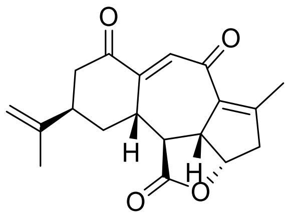

(+)-Ineleganolide (6). Triethylamine (67 µL, 484 µmol) was added to a solution of **3** (8.0 mg, 24.2 µmol) in acetonitrile (0.8 mL) and methanol (0.2 mL). The mixture was stirred at 60 °C (bath temperature) for 22 h before it was concentrated. The residue was purified by flash chromatography on silica, eluting with a gradient of 30-50% EtOAc in hexanes, to give a fraction containing unreacted **3** (2.8 mg, 35% yield) and a second fraction consisting of a mixture that contained ineleganolide. This fraction was purified by preparative HPLC (150 mm YMC Triart C18 5 µm, 10.0 mm ∅, MeOH in H_2O = 40:60 isocratic over 30 min, 4.7 mL/min, λ = 205 nm, t = 8.74 min) to give ineleganolide (**6**) as a white solid (1.01 mg, 20% yield brsm). $[\alpha]_D^{25}$ = +75.2 (c = 0.11, $CHCl_3$) (ref.:[29] $[\alpha]_D^{25}$ = +26.4 (c = 0.05, $CHCl_3$)); ^{1}H NMR (600 MHz, $CDCl_3$) δ = 5.13 (t, *J* = 7.5 Hz, 1H), 5.07 (d, *J* = 1.2 Hz, 1H), 4.94 (qd, *J* = 1.4, 0.7 Hz, 1H), 4.62 (p, *J* = 1.4 Hz, 1H), 3.42 (ddd, *J* = 12.0, 9.3, 7.6 Hz, 1H), 3.02 (dd, *J* = 12.0, 2.4 Hz, 1H), 3.01 (ddd, *J* = 14.0, 12.1, 5.3 Hz, 1H), 2.78 (m, 1H), 2.70 (dt, *J* = 12.4, 1.1 Hz, 1H), 2.67 (dt, *J* = 15.2, 2.4

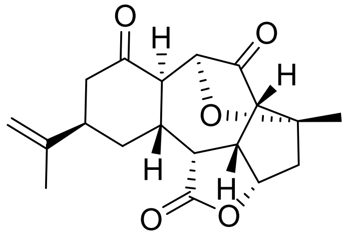

Hz, 1H), 2.59 (d, *J* = 9.3 Hz, 1H), 2.58 (ddd, *J* = 15.2, 6.5, 1.0 Hz, 1H), 2.52 (d, *J* = 15.5 Hz, 1H), 2.25 (tt, *J* = 12.3, 3.2 Hz, 1H), 2.10 (dd, *J* = 15.5, 7.3 Hz, 1H), 1.78 (dq, *J* = 13.9, 2.9 Hz, 1H), 1.71 (dt, *J* = 1.4, 0.7 Hz, 3H), 1.28 (s, 3H); ^{13}C NMR (151 MHz, $CDCl_3$) δ = 212.2, 206.5, 176.1, 146, 113.8, 91.1, 83.1, 77.5, 62.5, 49.8, 47.1, 45.5, 44.4, 43.8, 40.4, 33.2, 32.7, 22.7, 20.2; IR (film) ν/cm^{-1}: 2964, 2929, 1756, 1707, 1464, 1376, 1322, 1216, 1184, 1171, 1066, 1024, 898, 837, 754; HRMS (ESI): calcd for $C_{19}H_{22}O_5Na$ [M+Na$^+$]: 353.13594; found: 353.13577.

(+)-Horiolide (34). Triethylamine (47 µL, 333 µmol) was added to a solution of **3** (5.0 mg, 17 µmol) in acetonitrile (0.6 mL) and methanol (138 µL). The mixture was stirred at 60 °C for 44 h before it was concentrated. The residue was purified by flash chromatography on silica, eluting with a gradient of 30-50% EtOAc in hexanes to give a fraction containing unreacted scabrolide B (**3**) (1.3 mg, 24% yield), a fraction containing a diastereoisomeric mixture of horiolide andan isomer thereof (presumably 9-*epi*-horiolide, dr ≈ 1:1), and fraction containing impure ineleganolide. That latter fraction was purified by preparative HPLC (150 mm YMC Triart C18 5 µm, 10.0 mm ∅, MeOH in H_2O = 40:60 isocratic over 30 min, 4.7 mL/min, λ = 205 nm, t = 8.74 min) to give ineleganolide (**6**) (0.4 mg, 10% yield brsm) as a white solid.

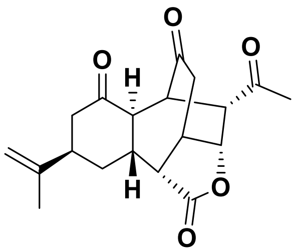

The residue containing the diastereoisomeric mixture of horiolide was purified by preparative HPLC (150 mm YMC Triart C18 5 µm, 10.0 mm ∅, MeOH in H_2O = 40:60 isocratic over 20 min, 4.7 mL/min, λ = 205 nm, t = 7.36 min) to give horiolide (**34**) (1.0 mg, 25% yield brsm) as a white solid. *Note*: *During the purification by preparative HPLC, the second diastereoisomer visible in the NMR spectra of the crude material must have epimerized into horiolide and was present in ≤3%.* $[\alpha]_D^{20}$ = +71.5 (c = 0.08, MeOH) (lit:[30] $[\alpha]_D$ = +81 (c = 0.037, MeOH; the temperature is not specified in the literature)). ^1H NMR (600 MHz, $CDCl_3$) δ = 5.42 (ddd, *J* = 8.3, 6.2, 1.5 Hz, 1H), 4.88 (q, *J* = 0.7 Hz, 1H), 4.60 (dq, *J* = 0.7, 0.7 Hz, 1H), 4.12 (dd, *J* = 3.0, 0.9 Hz, 1H), 3.37 (dddd, *J* = 8.5, 8.3, 4.6, 2.6 Hz, 1H), 3.00 (dd, *J* = 6.2, 3.0 Hz, 1H), 2.80 (bm, 1H), 2.74 (ddd, *J* = 15.2, 2.5, 2.3 Hz, 1H), 2.72 (dd, *J* = 8.5, 4.0 Hz, 1H), 2.65 (ddd, *J* = 13.6, 0.9, 0.9 Hz, 1H), 2.52 (dd, *J* = 19.7, 2.6 Hz, 1H), 2.51 (ddd, *J* = 15.2, 6.7, 0.9 Hz, 1H), 2.47 (dd, *J* = 19.7, 4.6 Hz, 1H), 2.31 (s, 3H), 2.20 (ddd, *J* = 14.0, 11.9, 4.9 Hz, 1H), 2.03 (dddd, *J* = 14.0, 3.0, 2.7, 2.5 Hz, 1H), 1.97 (dddd, *J* = 13.6, 11.9, 3.8, 3.0 Hz, 1H), 1.70 (ddd, *J* = 1.4, 0.7, 0.7 Hz, 3H); ^{13}C NMR (151 MHz, $CDCl_3$) δ = 206.8, 204.4, 201.9, 175.3, 145.8, 114.0, 76.4, 51.7, 50.0, 47.0, 43.7, 43.7, 40.4, 36.1, 36.1, 35.4, 31.7, 29.0, 22.6; IR (film) ν/cm^{-1}: 2922, 1772, 1725, 1707, 1453, 1366, 1293, 1222, 1164, 1097, 1067, 1011, 993, 915, 899, 741; HRMS (GC-EI): calcd for $C_{19}H_{22}O_5$[M$^+$]: 330.14618; found: 330.14600.

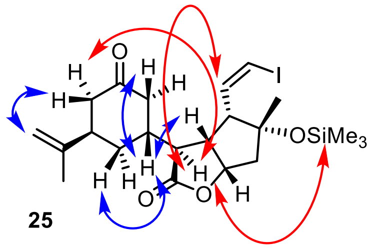

25

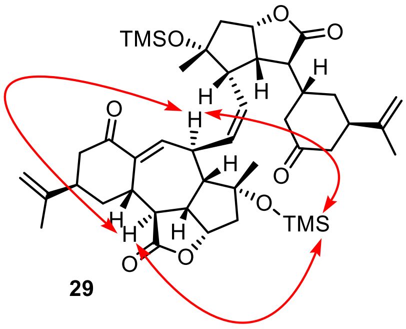

29

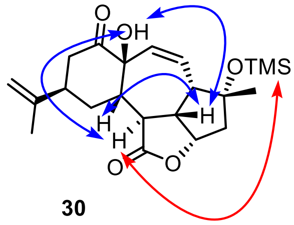

30

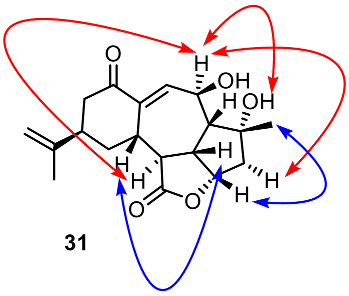

31

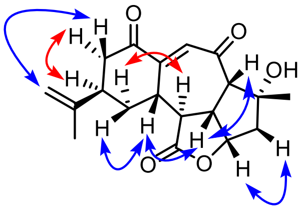

(-)-Scabrolide B (3)

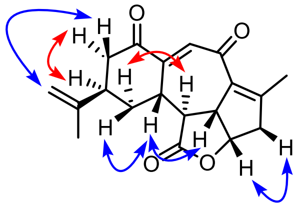

(-)-Sinuscalide C (4)

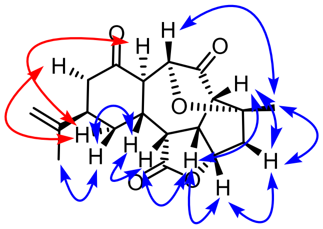

(+)-Ineleganolide (6)

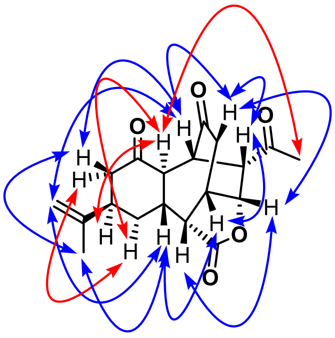

(+)-Horiolide (34)

Figure S7. Characteristic NOE interactions of key compounds (red: alpha-face; blue: beta-face)

Comparison of Natural and Synthetic Scabrolide B

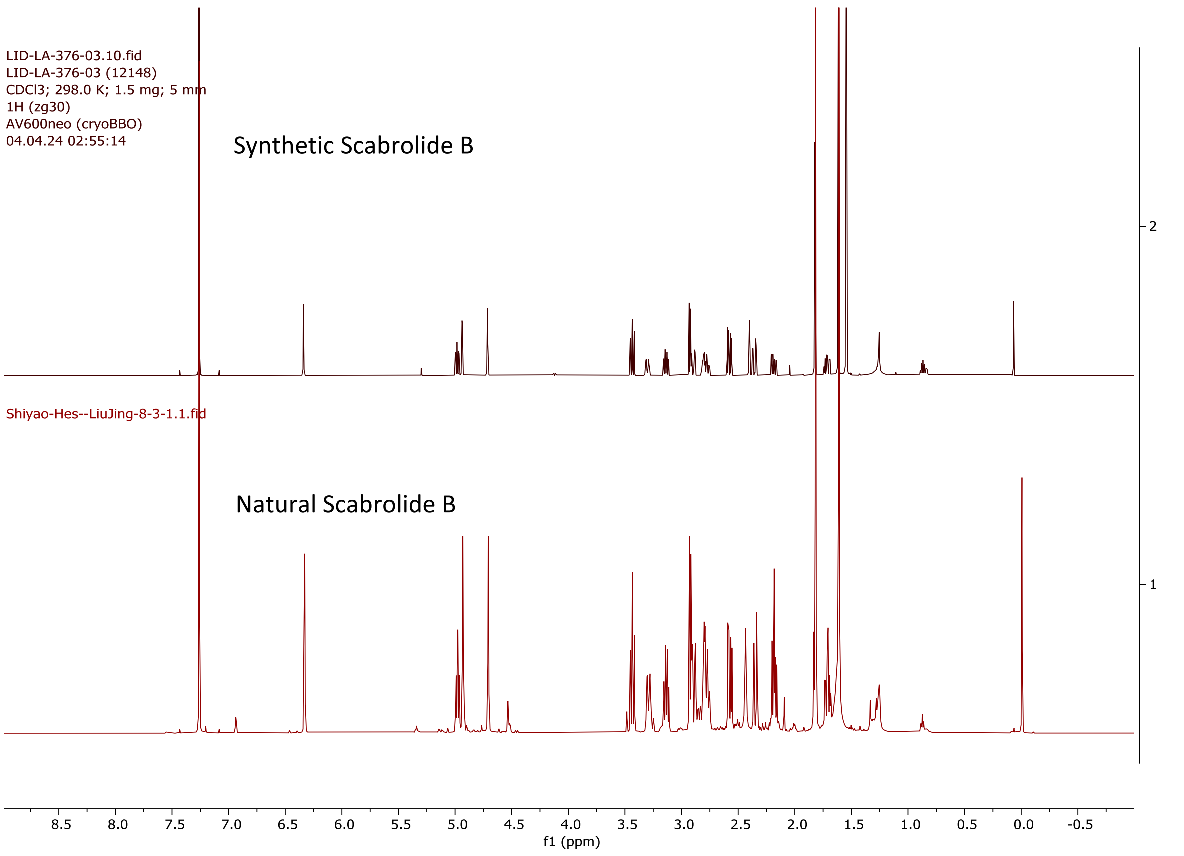

Figure S8. Visual comparison of the ^{1}H NMR spectra of natural and synthetic scabrolide B (**3**); top: synthetic **3**; bottom: ^{1}H NMR spectrum of the natural product published by He et al.[27]

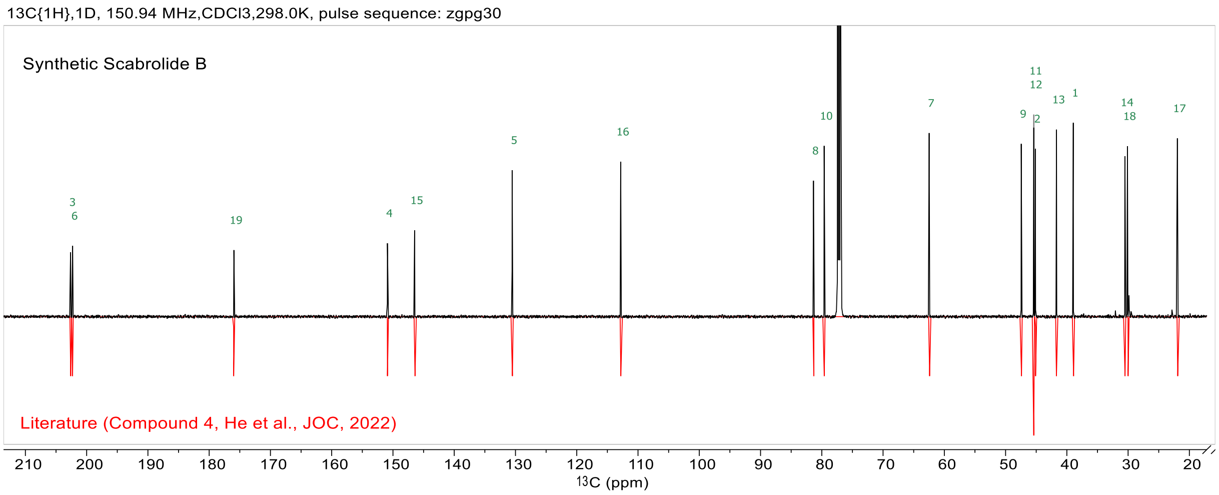

Figure S9. Visual Comparison of the ^{13}C NMR spectra of natural and synthetic scabrolide B (**3**); top: synthetic **3**; bottom: spectrum generated (MestReNova) by converting the literature data reported by He et al.[27] into a formal spectrum; the intensity of the signals is arbitrarily set to be identical.

Table S6. Comparison of the ^{1}H NMR data ($CDCl_3$) of natural and synthetic scabrolide B (**3**); numbering scheme as shown in the Insert.

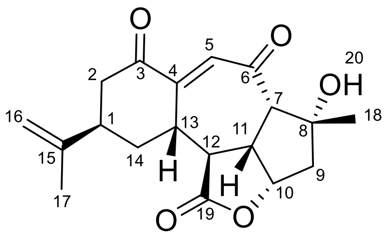

Position	Ref.[1]	Ref.[27]	This work
	δ (ppm) [*J* (Hz), 400 MHz]	δ (ppm) [*J* (Hz), 600 MHz]	
1	2.81 (m)	2.80 (m)	2.81 (m)
2a	2.90 (ddd, 16.0, 4.0, 2.5)	2.89 (dt, 16.4, 3.6)	2.90 (ddd, 16.2, 3.9, 2.3)
2b	2.60 (dd, 16.0, 6.0)	2.57 (dd, 16.2, 6.3)	2.58 (dd, 16.2, 6.3)
3			
4			
5	6.34 (d, 3.0)	6.33 (d, 2.7)	6.34 (d, 2.8)
6			
7	2.93 (d, 7.2)	2.92 (d, 7.5)	2.93 (d, 8.1)
8			
9a	2.36 (dd, 16.0, 2.5)	2.35 (dd, 16.2, 2.3)	2.36 (dd, 16.2, 2.3)
9b	2.21 (dd, 16.0, 9.0, 1.5)	2.19 (m)	2.19 (ddd, 16.2, 9.2, 2.0)
10	4.98 (td, 9.0, 2.5)	4.98 (td, 8.8, 2.3)	4.98 (ddd, 9.2, 8.1, 2.3)
11*	3.14 (dt, 10.5, 7.8)	3.13 (m)	3.14 (dt, 10.2, 8.1)
12*	3.45 (t, 10.5)	3.43 (t, 10.6)	3.44 (dd, 11.0, 10.2)
13	2.75 (m)	2.76 (m)	2.78 (dddd, 12.3, 11.0, 4.1, 2.8)
14a	3.30 (dtd, 10.0, 4.0, 2.5)	3.29 (dq, 13.9, 2.3)	3.30 (dtd, 13.8, 4.1, 2.3)
14b	1.71 (tdd, 10.0, 5.0, 1.2)	1.71 (td, 13.5, 5.2)	1.71 (ddd, 13.8, 12.3, 5.2)
15			
16a	4.95 (s)	4.93 (s)	4.94 (q, 1.2)
16b	4.72 (s)	4.71 (s)	4.71 (dt, 1.6, 0.8)
17	1.83 (s)	1.82 (s)	1.82 (dt, 1.2, 0.8)
18	1.63 (s)	1.61 (s)	1.62 (s)
19			
20			2.40 (d, 2.0)

* In both reported datasets of scabrolide B, protons H11 and H12 were interchanged. The new assignment was confirmed by COSY and the positions were therefore corrected in our table.

Table S7. Comparison of the ^{13}C NMR data ($CDCl_3$) of natural and synthetic scabrolide B (**3**).

**Position**	**Ref.**[1] δ (ppm) [75 MHz]	**Ref.**[27] δ (ppm) [150 MHz]	**This work** δ (ppm) [151 MHz]
1	38.9	38.9	39.0
2	45.0	45.1	45.1
3	202.2	202.3	202.6
4	130.5	150.9	150.9
5	150.8	130.5	130.6
6	202.5	202.6	202.3
7	62.4	62.4	62.5
8	81.3	81.3	81.4
9	47.4	47.4	47.5
10	79.5	79.6	79.6
11	45.3	45.4	45.4
12	45.3	45.4	45.4
13	41.6	41.7	41.7
14	30.5	30.5	30.5
15	146.4	146.4	146.5
16	112.7	112.8	112.8
17	21.8	21.9	22.0
18	30.0	30.0	30.1
19	175.9	176.0	176.0

Comparison of Natural and Synthetic Sinuscalide C

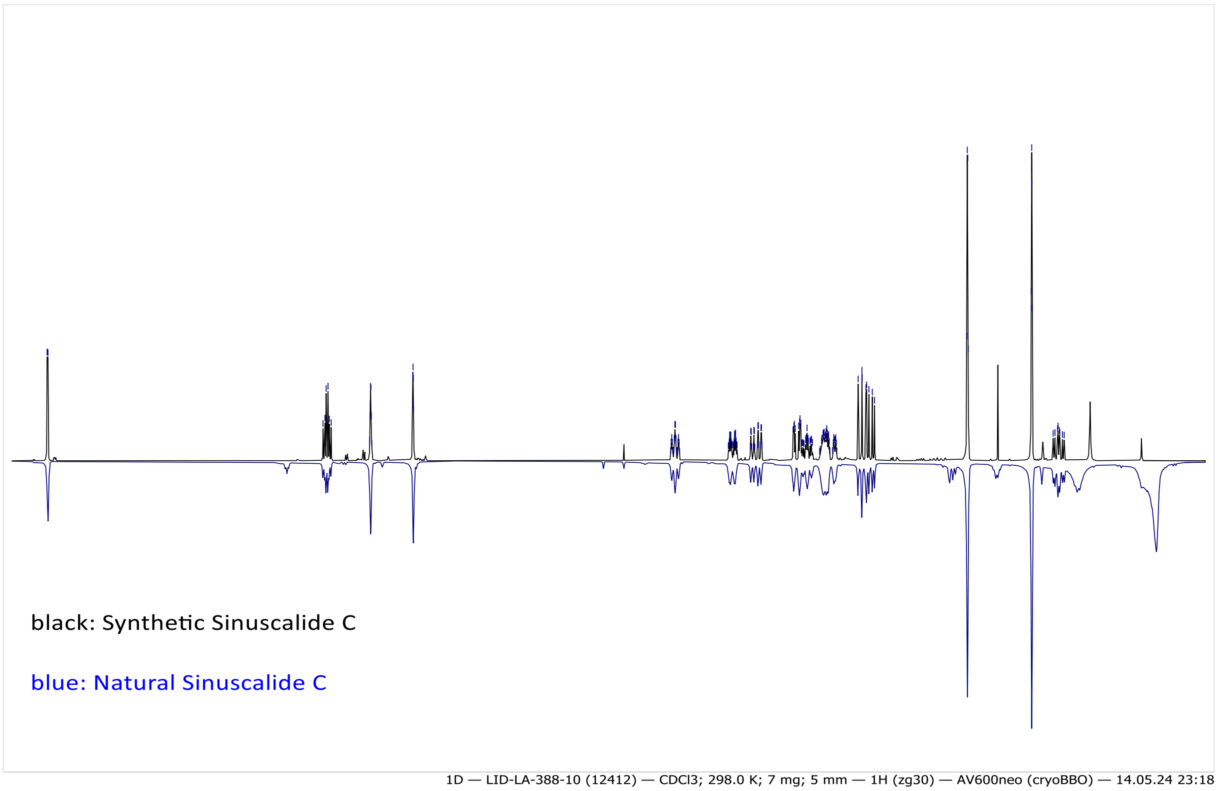

Figure S10. Visual comparison of the ^{1}H NMR spectra of natural and synthetic sinuscalide C (**4**); top: synthetic **4**; bottom: ^{1}H NMR spectrum of the natural product published by He et al.[27]

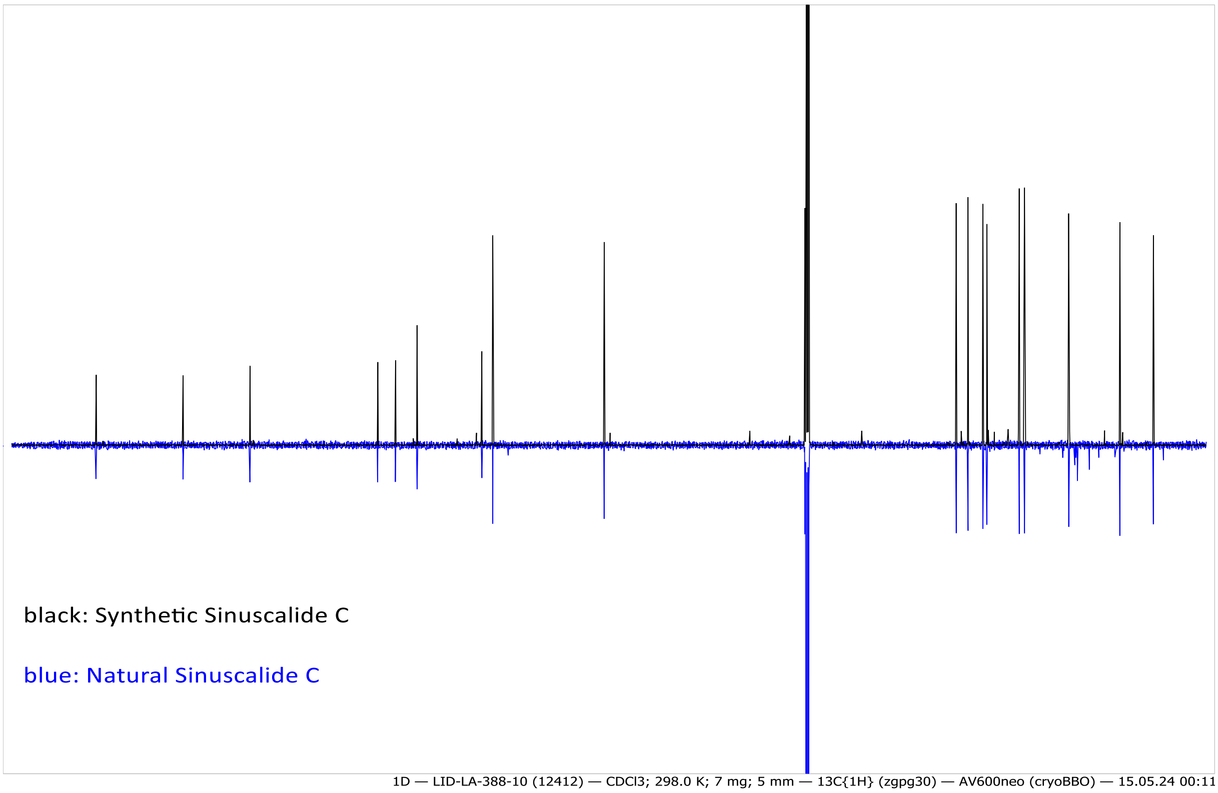

Figure S11. Visual Comparison of the ^{13}C NMR spectra of natural and synthetic sinuscalide C (**4**); top: synthetic **3**; bottom: spectrum of the natural product reported by He et al.[27]

Table S8. Comparison of the ^1H NMR data ($CDCl_3$) of natural and synthetic sinuscalide C (**4**); numbering scheme as shown in the Insert.

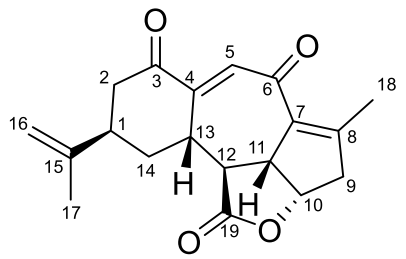

Position	Ref.[27]	This work
	δ (ppm) [*J* (Hz), 600 MHz]	
1	2.82 (m)	2.82 (m)
2a	2.95 (dt, 16.0, 3.0)	2.95 (ddd, 16.0, 3.6, 2.5)
2b	2.60 (dd, 16.2, 6.5)	2.60 (dd, 16.0, 6.4)
3		
4		
5	6.47 (d, 2.6)	6.47 (d, 2.8)
6		
7*		
8*		
9a	3.14 (m)	3.14 (ddq, 20.1, 8.8, 1.2)
9b	2.79 (m)	2.79 (dddd, 20.1, 6.6, 3.2, 1.4)
10	5.16 (td, 8.8, 5.3)	5.15 (td, 8.8, 5.3)
11	3.52 (t, 8.3)	3.52 (ddp, 10.5, 8.7, 1.5)
12	2.64 (t, 11.1)	2.64 (dd, 11.3, 10.5)
13	2.90 (m)	2.90 (dddd, 12.4, 11.3, 4.12, 2.6)
14a	3.25 (dq, 13.8, 3.5)	3.25 (dtdd, 13.7, 4.2, 2.5, 0.5)
14b	1.72 (td, 13.2, 4.9)	1.72 (ddd, 13.7, 12.4, 5.0)
15		
16a	4.95 (s)	4.95 (s)
16b	4.75 (s)	4.75 (dt, 1.6, 0.8)
17	1.85 (s)	1.84 (dt, 1.4, 0.7)
18	2.15 (s)	2.14 (q, 1.5)
19		

* In the reported data for sinuscalide C, C7 and C8 were interchanged. The new assignment of these signals was confirmed by 2D NMR spectroscopy and the positions were therefore corrected in our Table.

Table S9. Comparison of the ^{13}C NMR data ($CDCl_3$) of natural and synthetic sinuscalide C (**4**).

**Position**	**Ref.**[27] ppm [150 MHz]	**This work** ppm [151 MHz]	**Δδ** ppm
1	38.8	38.9	0.1
2	45.5	45.5	0
3	202.9	202.8	0.1
4	150.0	149.9	0.1
5	132.8	132.8	0
6	187.5	187.5	0
7	134.7	134.7	0
8	153.1	153.1	0
9	46.2	46.2	0
10	77.6	77.6	0
11	50.9	50.9	0
12	48.8	48.8	0
13	39.7	39.7	0
14	31.0	31.0	0
15	146.1	146.2	0.1
16	113.1	113.1	0
17	22.0	22.0	0
18	16.0	16.0	0
19	175.7	175.7	0

Comparison of Natural and Synthetic Ineleganolide

Table S10. Comparison of the ^{1}H NMR data ($CDCl_3$) of natural and synthetic ineleganolide (**6**); numbering scheme as shown in the Insert.

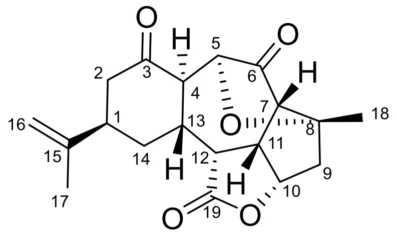

Position	Ref.[29]	Ref.[31] ppm [*J* (Hz), 400 MHz]	Ref.[32]	This work ppm [*J* (Hz), 600 MHz]
1	2.78 (br s)	2.79 (br s)	2.78 (br s)	2.78 (m)
2a	2.63 (m)	2.64 (m)	2.64 (m)	2.67 (dt, 15.2, 2.4)
2b	2.63 (m)	2.64 (m)	2.64 (m)	2.58 (ddd, 15.2, 6.5, 1.0)
3				
4	2.70 (d, 13.0)	2.70 (d, 13.1)	2.70 (d, 13.0)	2.70 (dt, 12.4, 1.1)
5	5.07 (s)	5.07 (s)	5.07 (s)	5.07 (d, 1.2)
6				
7	2.59 (d, 9.3)	2.59 (d, 9.3)	2.59 (d, 9.3)	2.59 (d, 9.3)
8				
9a	2.10 (dd, 15.6, 7.2)	2.10 (dd, 15.7, 7.3)	2.10 (dd, 15.5, 7.3)	2.10 (dd, 15.5, 7.3)
9b	2.51 (d, 15.6)	2.52 (d, 15.5)	2.52 (d, 15.6)	2.52 (d, 15.5)
10	5.13 (t, 7.2)	5.12 (t, 7.3)	5.12 (t, 7.4)	5.13 (t, 7.5)
11	3.42 (ddd, 12.3, 9.3, 7.2)	3.42 (ddd, 12.2, 9.3, 7.5)	3.42 (ddd, 12.1, 9.2, 7.6)	3.42 (ddd, 12.0, 9.3, 7.6)
12	3.02 (dd, 12.3, 2.5)	3.02 (dd, 12.2, 2.5)	3.02 (dd, 12.0, 2.4)	3.02 (dd, 12.0, 2.4)
13	2.24 (tt, 13.0, 2.5)	2.25 (tt, 12.6, 2.7)	2.25 (tt, 12.4, 2.8)	2.25 (tt, 12.3, 3.2)
14a	3.00 (m)	3.00 (m)	3.00 (m)	3.01 (ddd, 14.0, 12.1, 5.3)
14b	1.79 (m)	1.79 (dq, 13.0, 2.6)	1.78 (dq, 13.0, 2.8)	1.78 (dq, 13.9, 2.9)
15				
16a	4.62 (s)	4.62 (s)	4.62 (s)	4.62 (p, 1.4)
16b	4.94 (s)	4.94 (s)	4.94 (s)	4.94 (qd, 1.4, 0.7)
17	1.71 (s)	1.71 (s)	1.71 (s)	1.71 (dt, 1.4, 0.7)
18	1.28 (s)	1.28 (s)	1.28 (s)	1.28 (s)

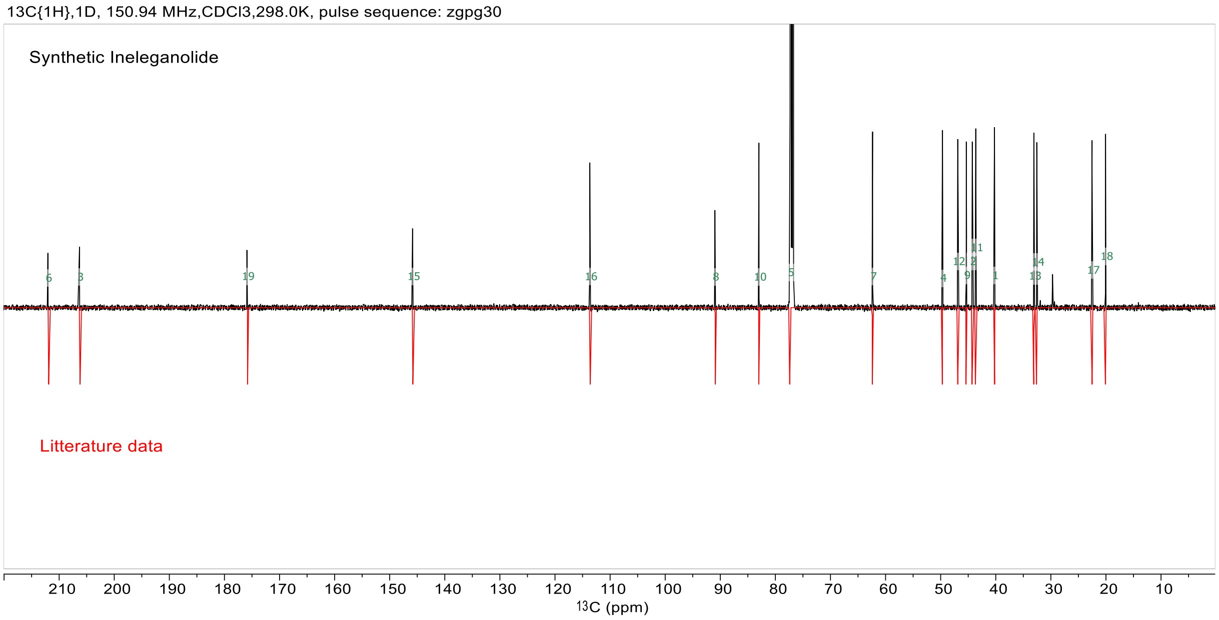

Figure S12. Visual Comparison of the ^{13}C NMR spectra of natural and synthetic ineleganolide (**6**); top: synthetic **6**; bottom: spectrum generated (MestReNova) by converting the literature data[29] into a formal spectrum; the intensity of the signals is arbitrarily set to be identical.

Table S11. Comparison of the ^{13}C NMR data ($CDCl_3$) of natural and synthetic ineleganolide (**6**).

Position	Ref.[29] ppm [100.6 MHz]	Ref.[31] ppm [100 MHz]	Ref.[32]	This work ppm [151 MHz]
1	40.2	40.3	40.4	40.4
2	44.3	44.3	44.4	44.4
3	206.2	206.3	206.4	206.5
4	49.7	49.7	49.8	49.8
5	77.4	77.3	77.4	77.5
6	211.9	212.1	212.2	212.2
7	62.4	62.4	62.5	62.5
8	90.9	91.0	91.1	91.1
9	45.4	45.4	45.5	45.5
10	83.0	83.0	83.1	83.1
11	43.7	43.6	43.8	43.8
12	46.9	46.9	47.0	47.1
13	33.1	33.1	33.2	33.2
14	32.6	32.6	32.7	32.7
15	145.8	145.9	146.0	146.0
16	113.6	113.7	113.8	113.8
17	22.5	22.5	22.7	22.7
18	20.1	20.1	20.2	20.2
19	175.8	175.9	176.1	176.1

Comparison of Natural and Synthetic Horiolide

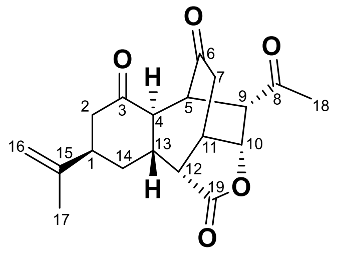

Table S12. Comparison of the ^{1}H NMR data ($CDCl_3$) of natural and synthetic horiolide (**34**); numbering scheme as shown in the Insert

Position	Ref.[30] δ (ppm) [*J* (Hz), 500 MHz]	This work δ (ppm) [*J* (Hz), 600 MHz]	Δδ (ppm)
1	2.78	2.80 (bm)	0.02
2a	2.46	2.51 (ddd, 15.2, 6.7, 0.9)	0.05
2b	2.71	2.74 (ddd, 15.2, 2.5, 2.3)	0.03
3			
4	2.61	2.65 (ddd, 13.6, 0.9, 0.9)	0.04
5	4.08	4.12 (dd, 3.0, 0.9)	0.04
6			
7a	2.20	2.52 (dd, 19.7, 2.6)	0.32
7b	2.44	2.47 (dd, 19.7, 4.6)	0.03
8			
9	2.99 (dd, 6, 3)	3.00 (dd, 6.2, 1.5)	0.01
10	5.40 (dd, 8, 8)	5.42 (ddd, 8.3, 6.2, 1.5)	0.02
11	3.34	3.37 (dddd, 8.5, 8.3, 4.6, 2.6)	0.03
12	2.69	2.72 (dd, 8.5, 3.8)	0.03
13	1.96	1.97 (dddd, 13.6, 11.9, 3.8, 3.0)	0.01
14a	2.16	2.20 (ddd, 14.0, 11.9, 4.9)	0.04
14b	1.99	2.03 (dddd, 14.0, 3.0, 2.7, 2.5)	0.04
15			
16a	4.57	4.60 (dq, 0.7, 0.7)	0.03
16b	4.85	4.88 (q, 0.7)	0.03
17	1.65	1.70 (ddd, 1.4, 0.7, 0.7)	0.05
18	2.30	2.31 (s)	0.01

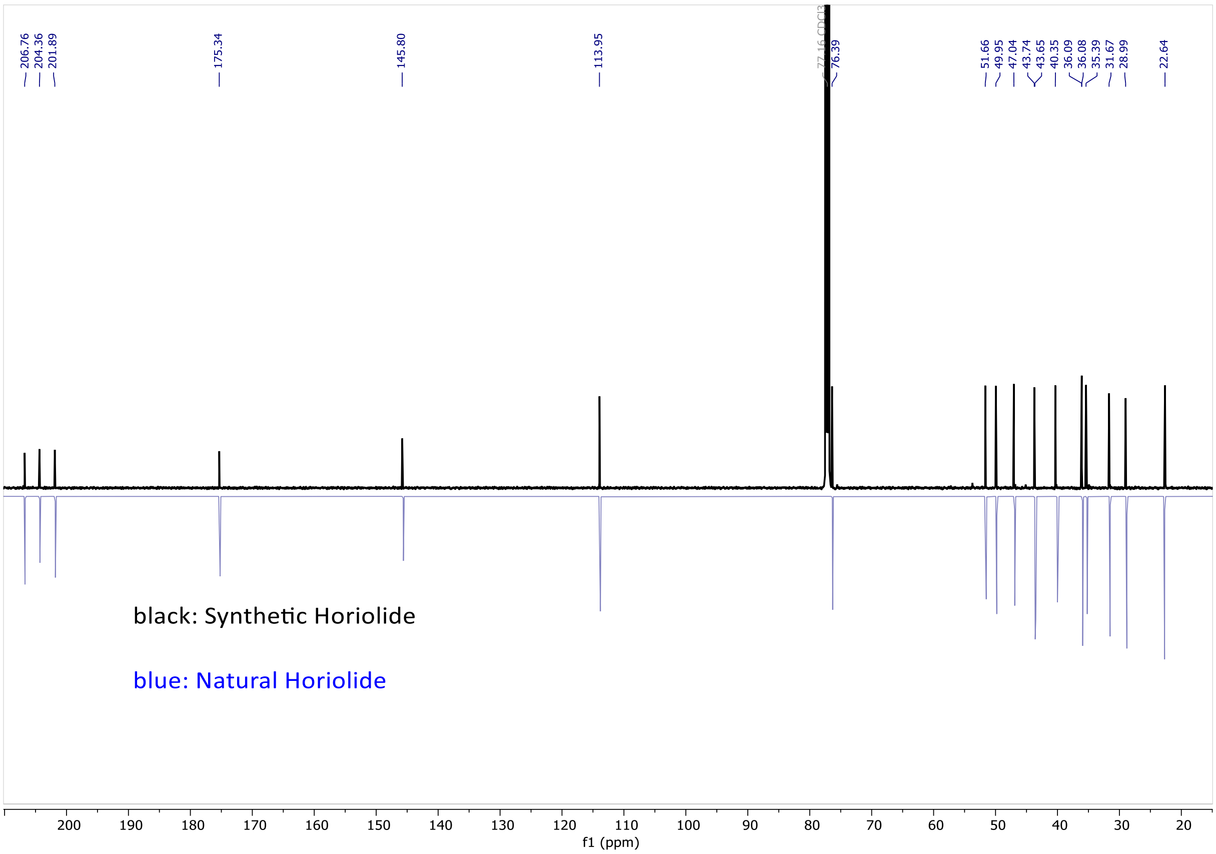

Figure S13. Visual Comparison of the ^{13}C NMR spectra of natural and synthetic horiolide (**34**); top: synthetic **34**; bottom: spectrum generated (MestReNova) by converting the literature data[30] into a formal spectrum.

Table S13. Comparison of the ^{13}C NMR data ($CDCl_3$) of natural and synthetic horiolide (**34**)

**Position**	**Ref.[30]** δ (ppm) [125 MHz]	**This work** δ (ppm) [150 MHz]	**Δδ** (ppm)
1	40.2	40.4	0.2
2	43.6	43.7	0.1
3	204.3	204.4	0.1
4	49.8	50.0	0.2
5	43.5	43.7	0.2
6	206.7	206.8	0.1
7	35.9	36.1	0.2
8	201.8	201.9	0.1
9	51.5	51.7	0.2
10	76.3	76.4	0.1
11	35.2	36.1	0.9
12	46.9	47.0	0.1
13	35.9	35.4	0.5
14	31.5	31.7	0.2
15	145.6	145.8	0.2
16	113.8	114.0	0.2
17	22.7	22.6	0.1
18	28.8	29.0	0.2
19	175.2	175.3	0.1

Copies of NMR Spectra of New Compounds

^{1}H NMR of S2 (400 MHz, $CDCl_3$)

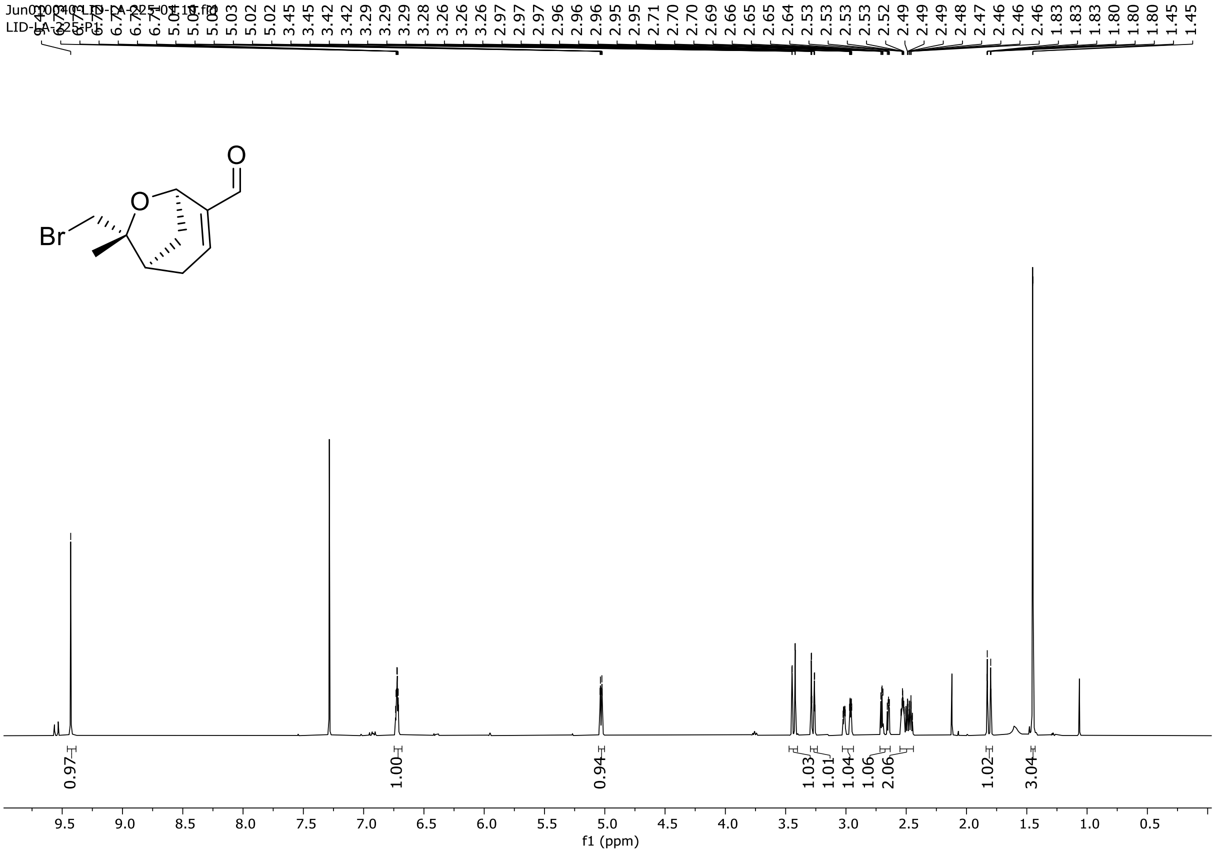

^{13}C NMR of S2 (101 MHz, $CDCl_3$)

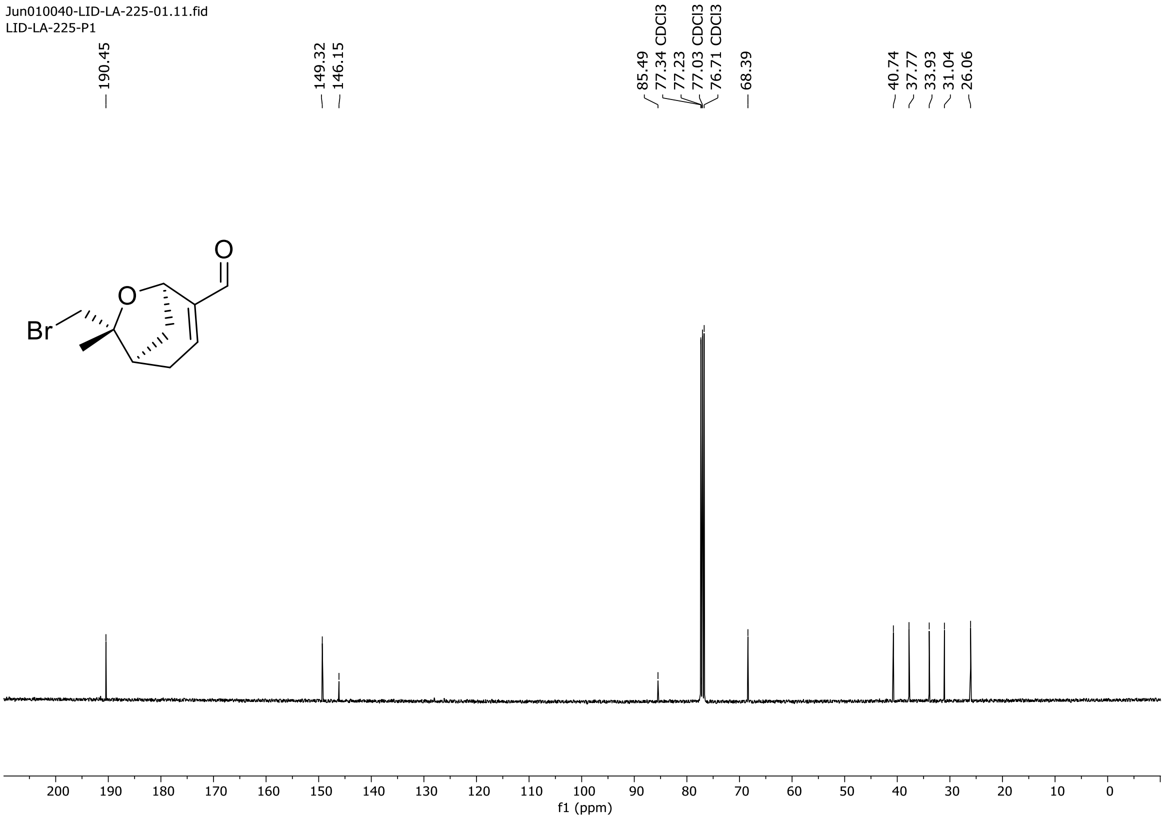

^{1}H NMR of S4 (400 MHz, $CDCl_3$)

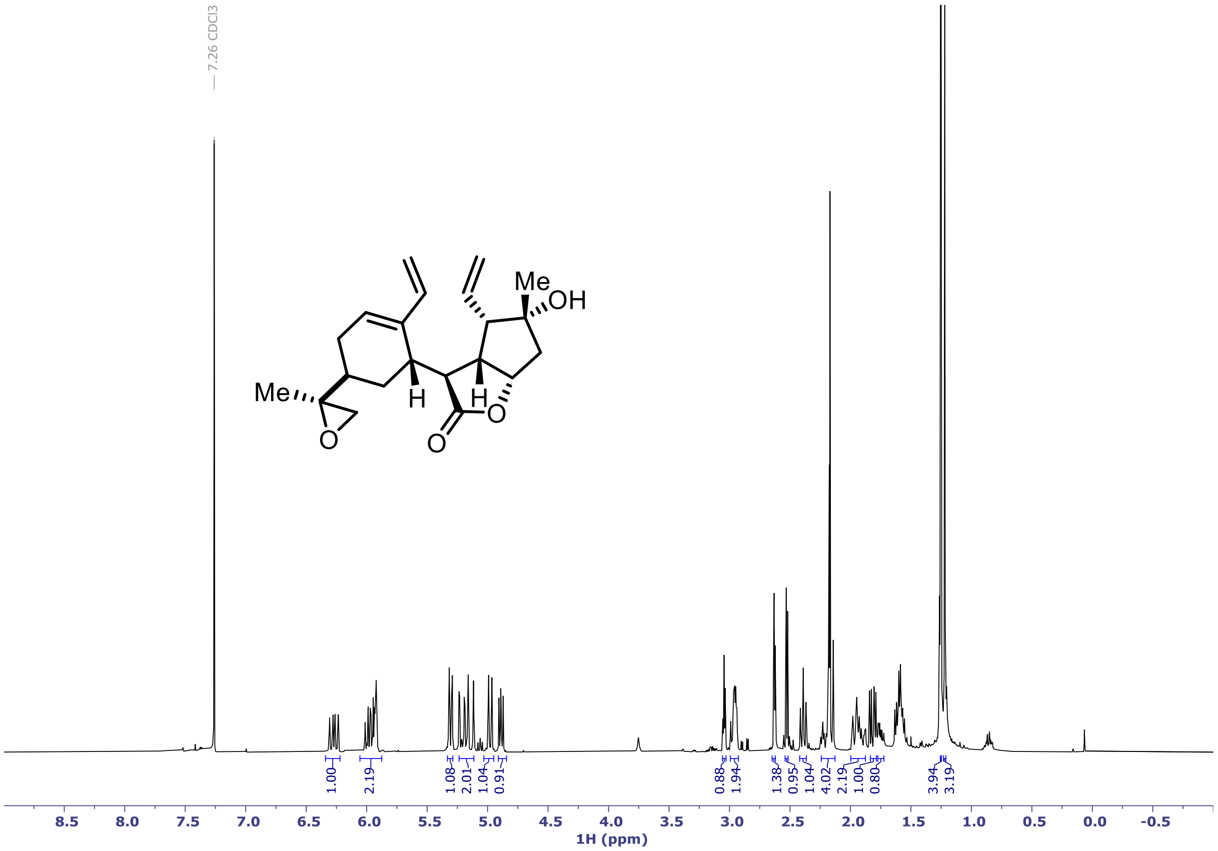

^{13}C NMR of S4 (101 MHz, $CDCl_3$)

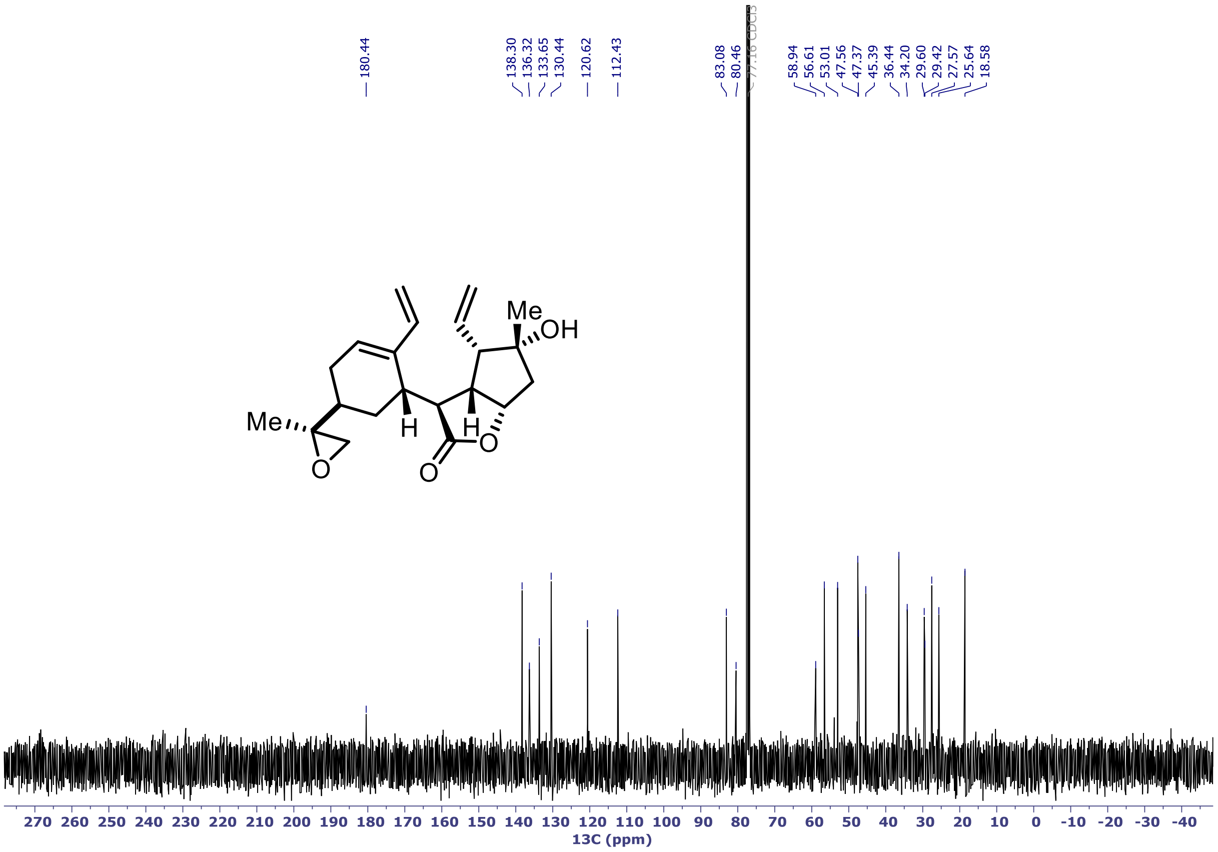

^{1}H NMR of 11 (400 MHz, $CDCl_3$)

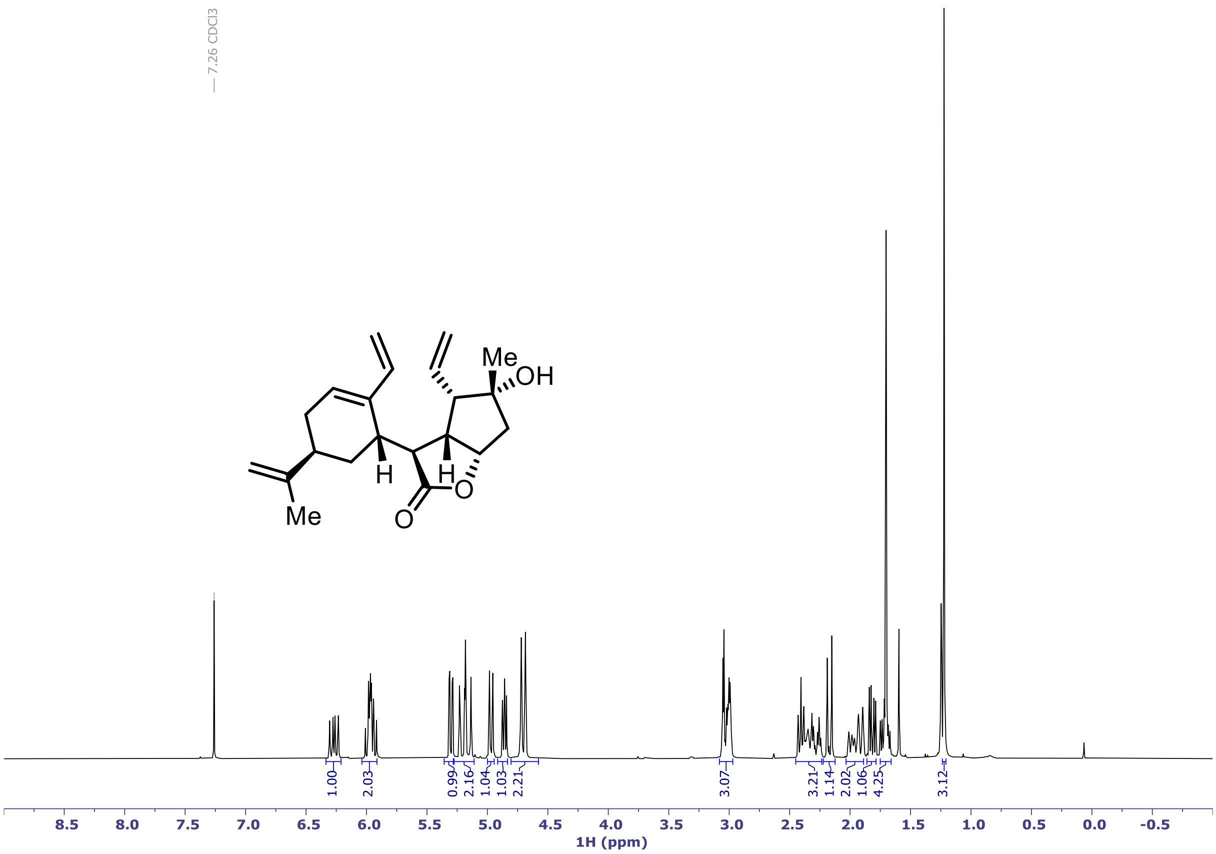

^{13}C NMR of 11 (101 MHz, $CDCl_3$)

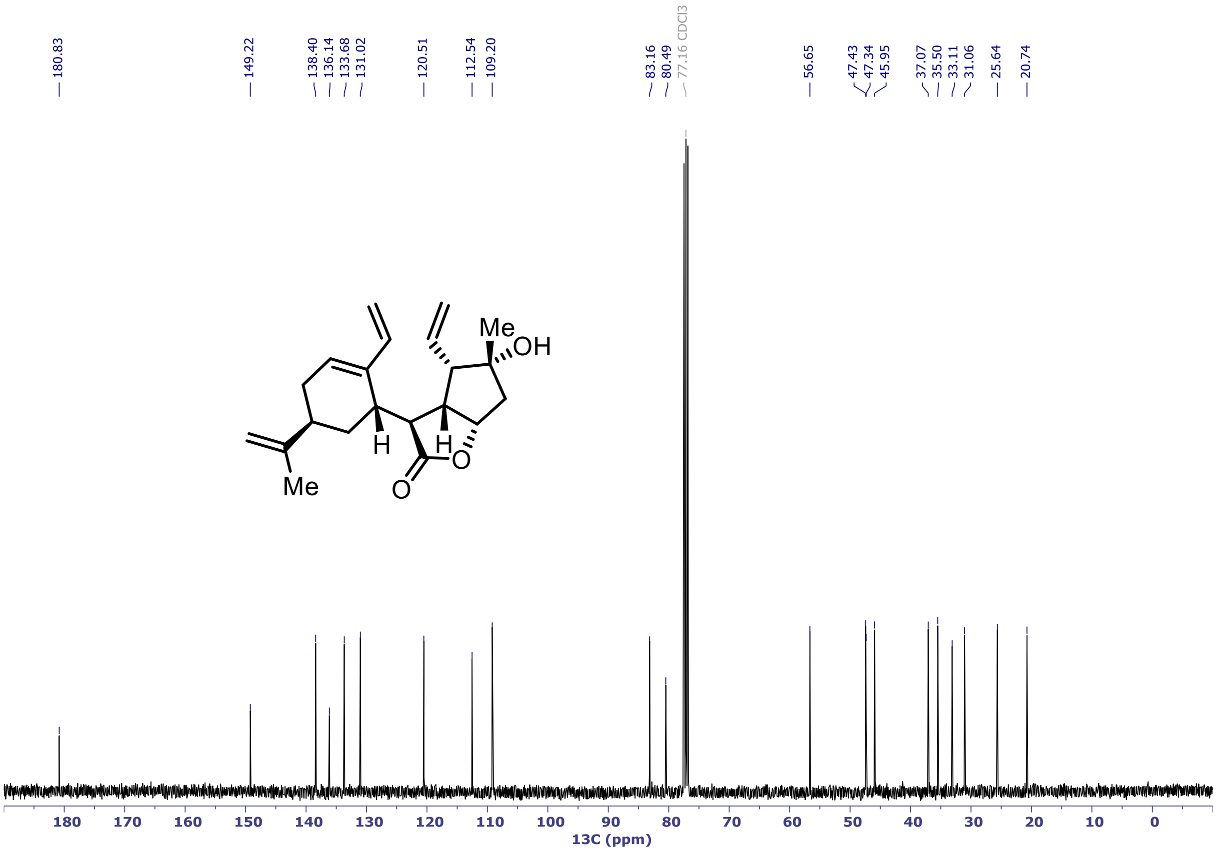

^{1}H NMR of 12 (400 MHz, $CDCl_3$)

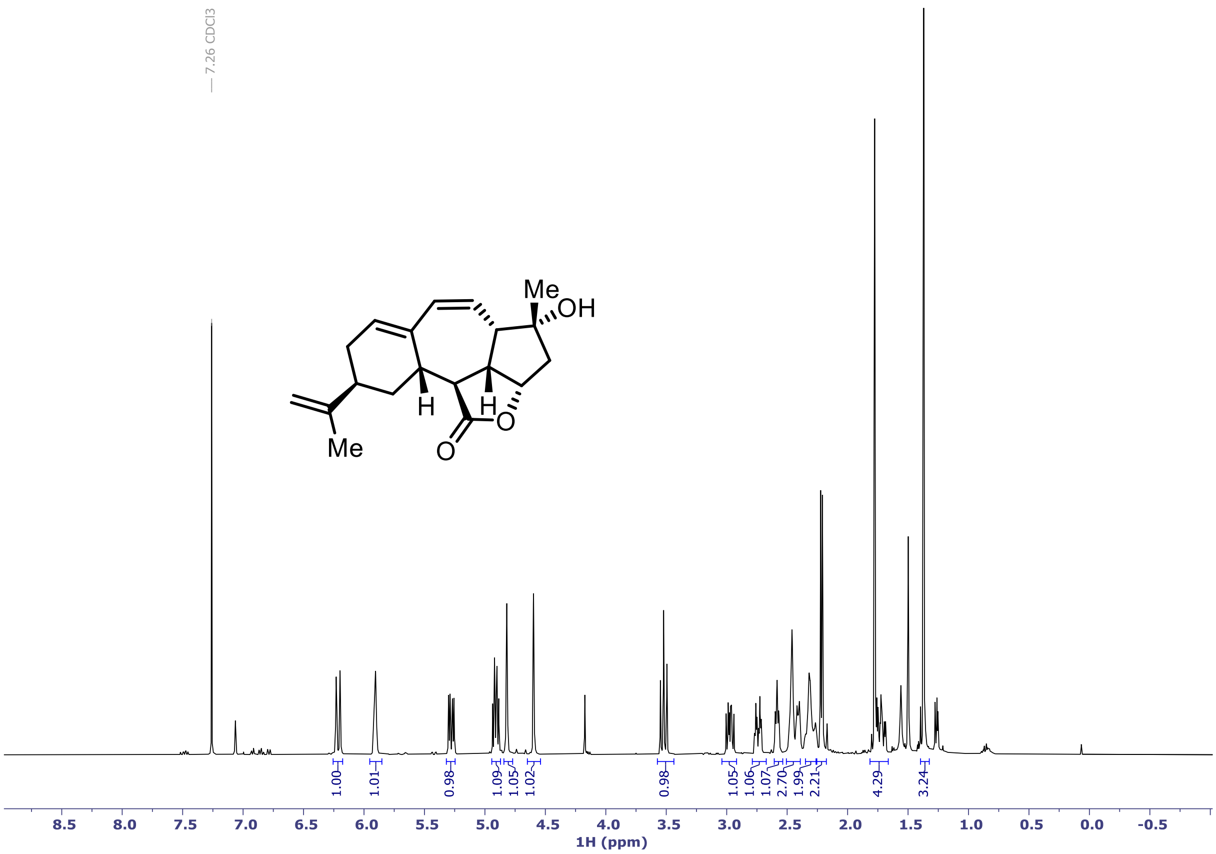

^{13}C NMR of 12 (101 MHz, $CDCl_3$)

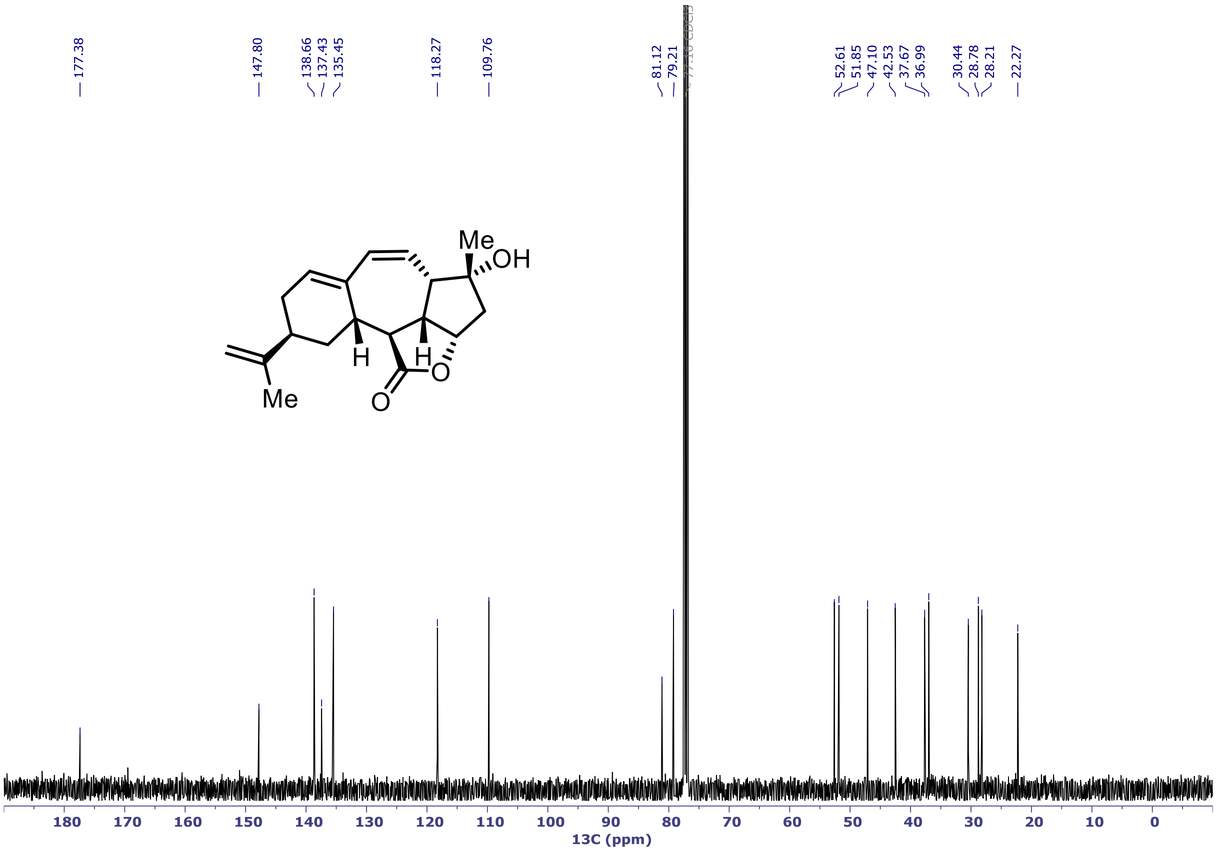

^{1}H NMR of S5 (400 MHz, $CDCl_3$)

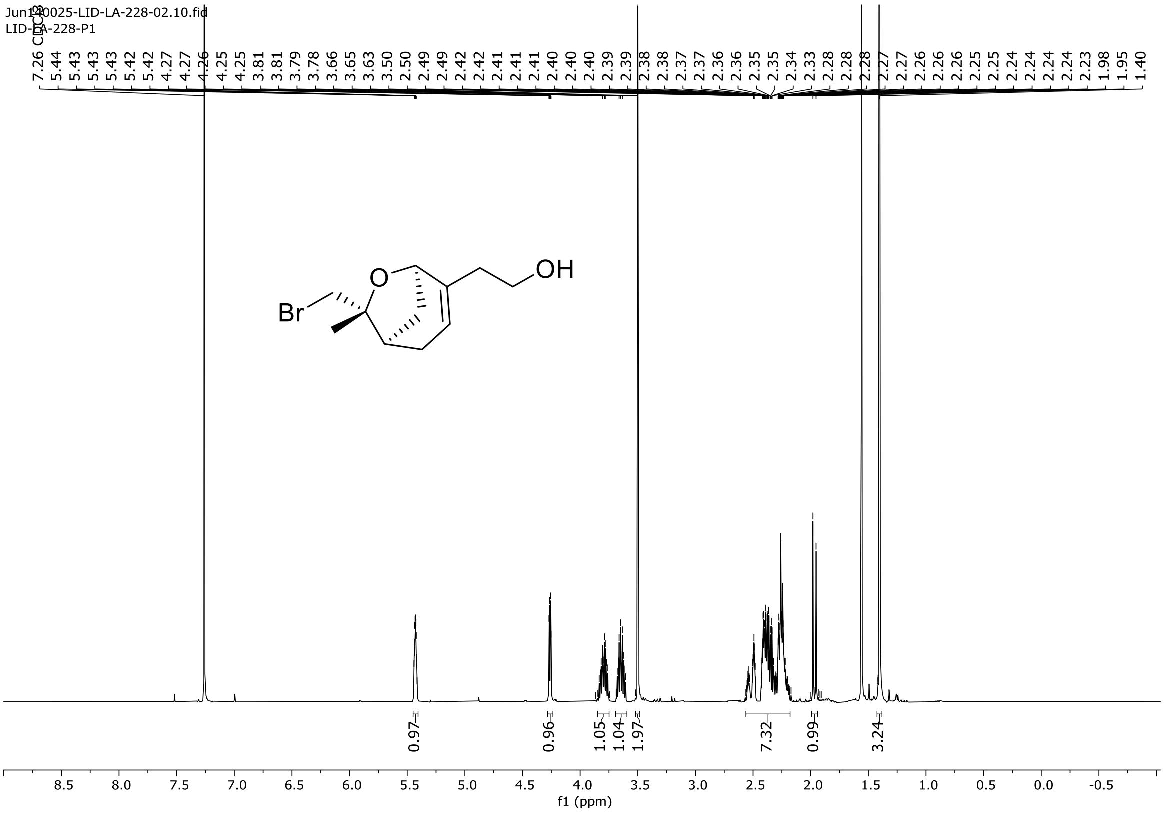

^{13}C NMR of S5 (101 MHz, $CDCl_3$)

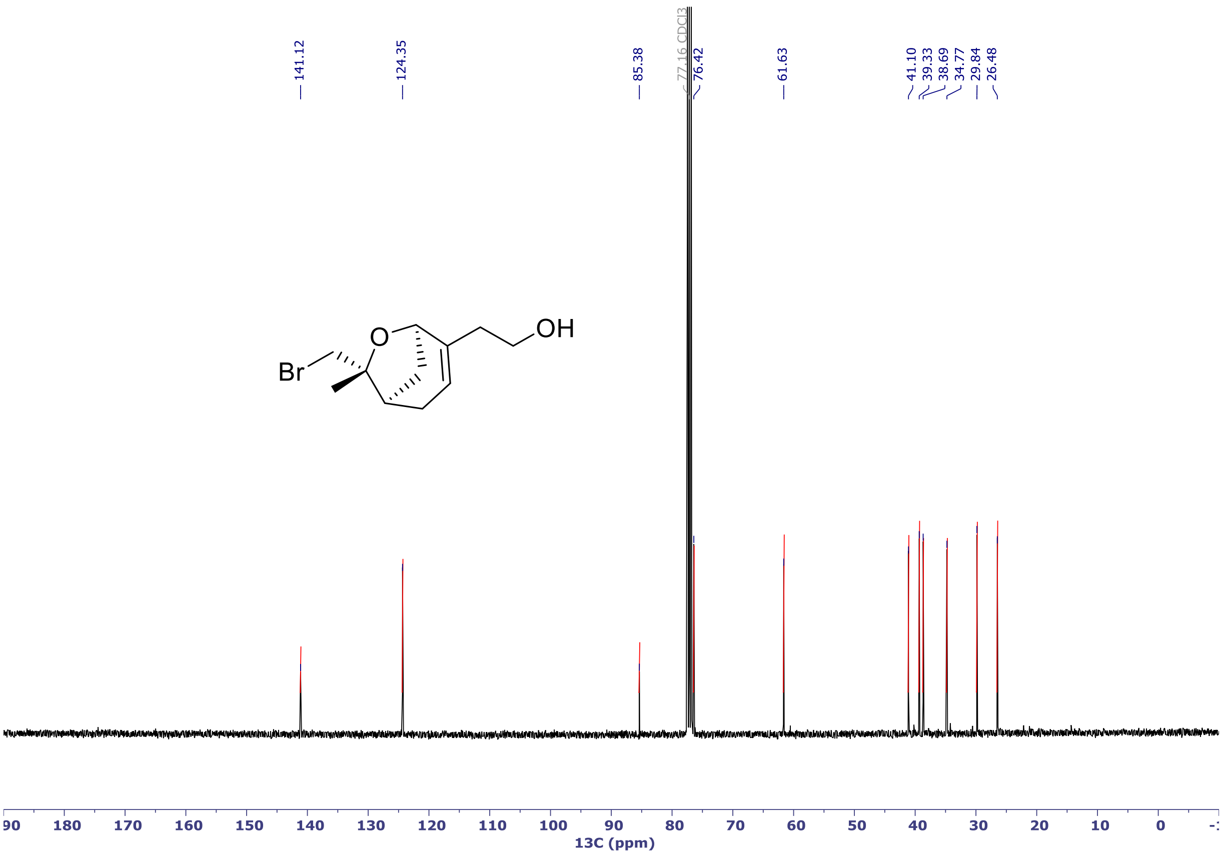

^{1}H NMR of S6 (400 MHz, CDCl$_3$)

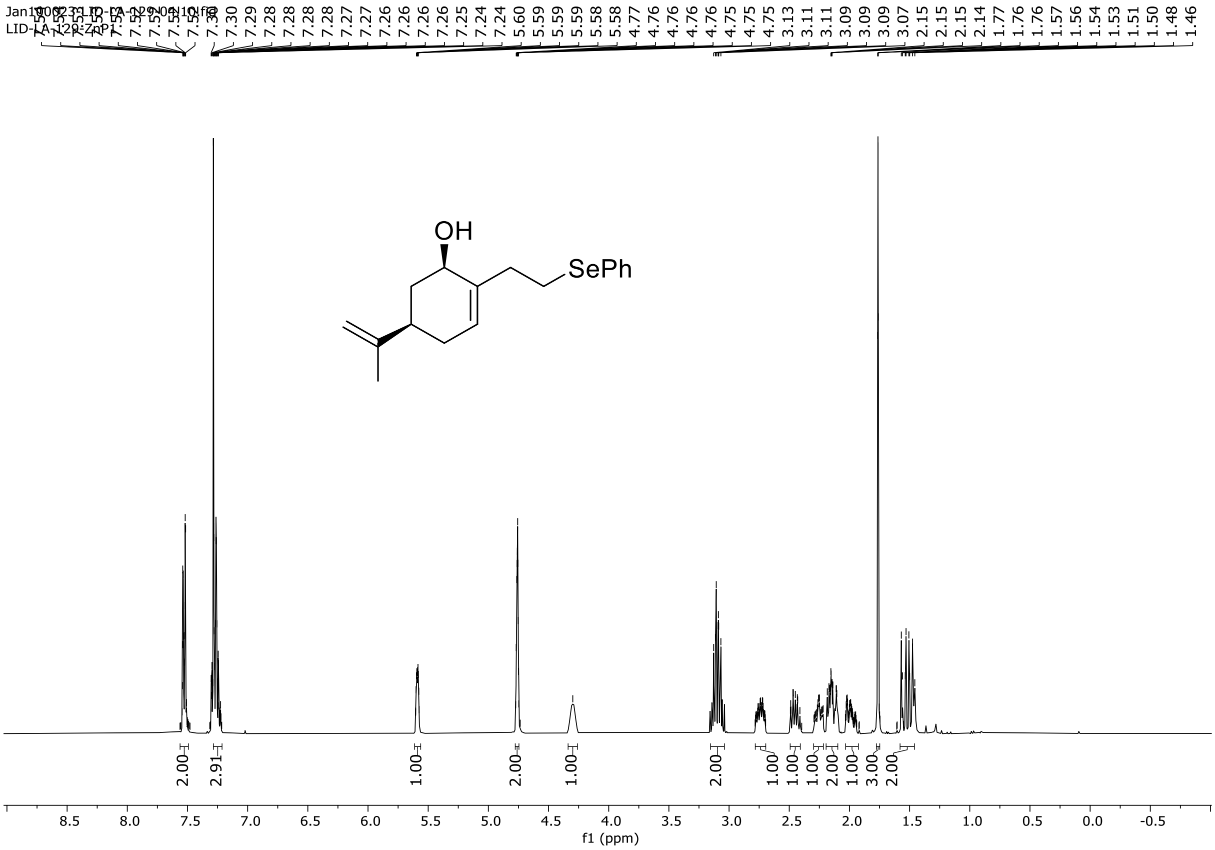

^{13}C NMR of S6 (101 MHz, CDCl$_3$)

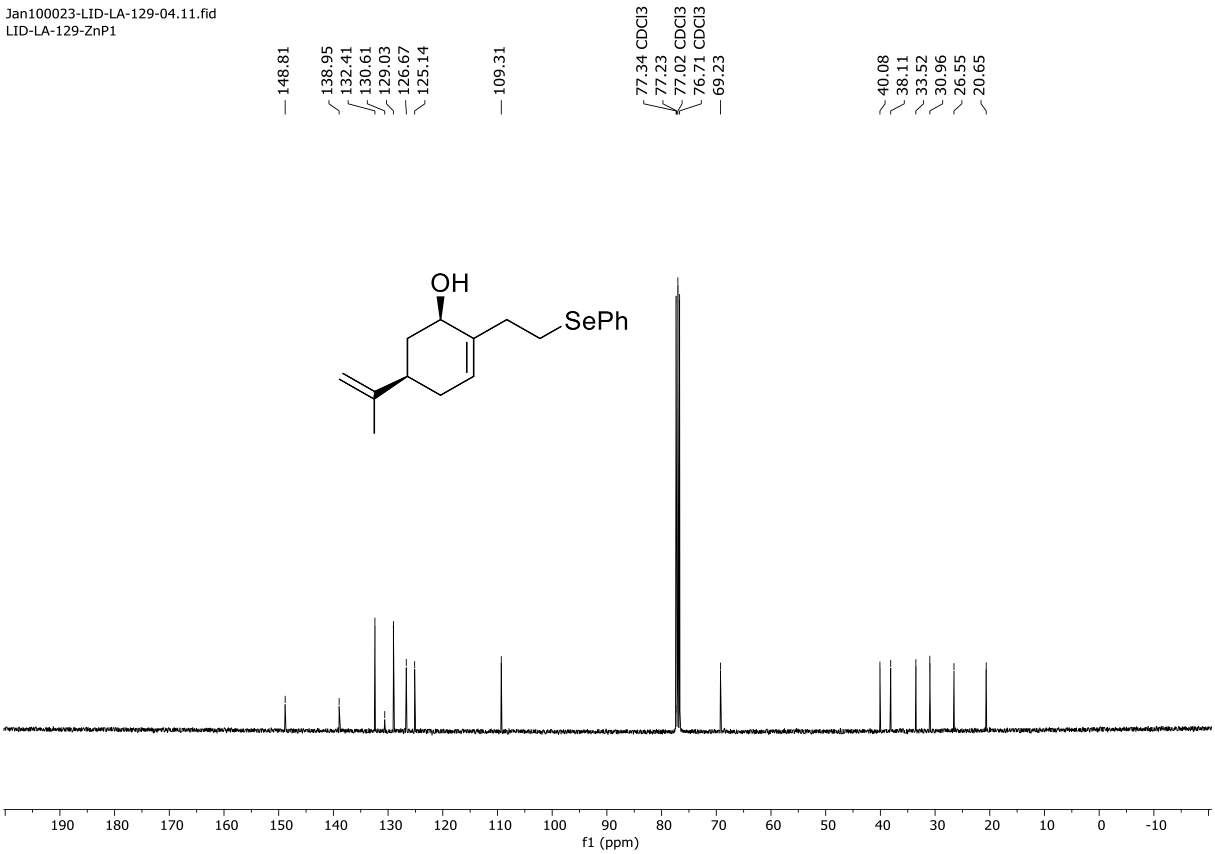

^{1}H NMR of S7 (400 MHz, CDCl$_3$)

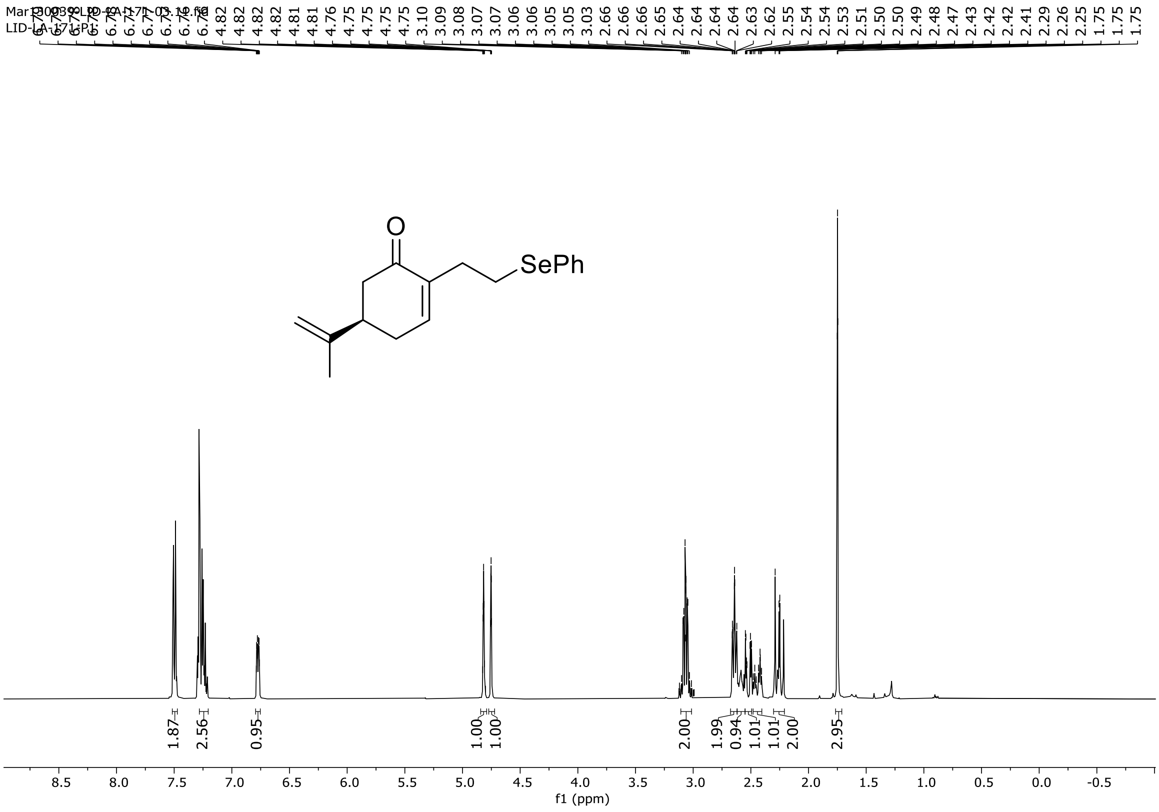

^{13}C NMR of S7 (101 MHz, CDCl$_3$)

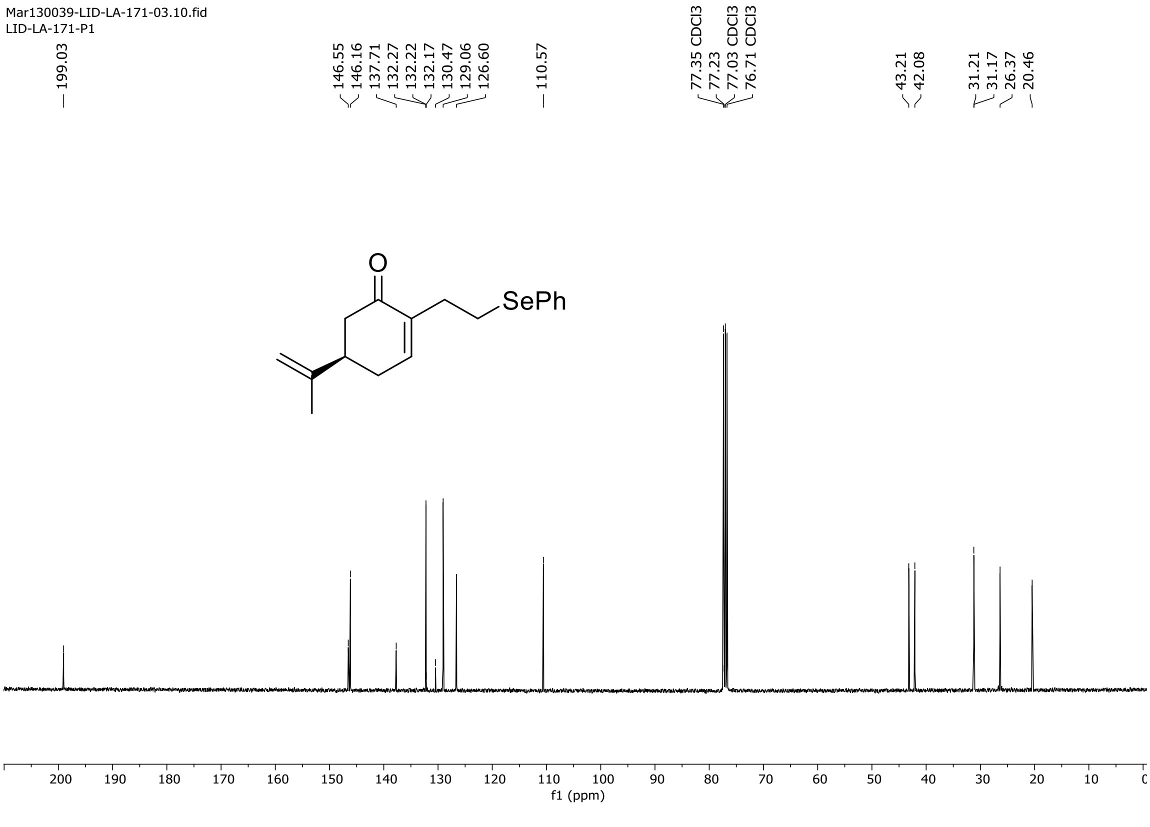

^{1}H NMR of 13 (600 MHz, CD_2Cl_2)

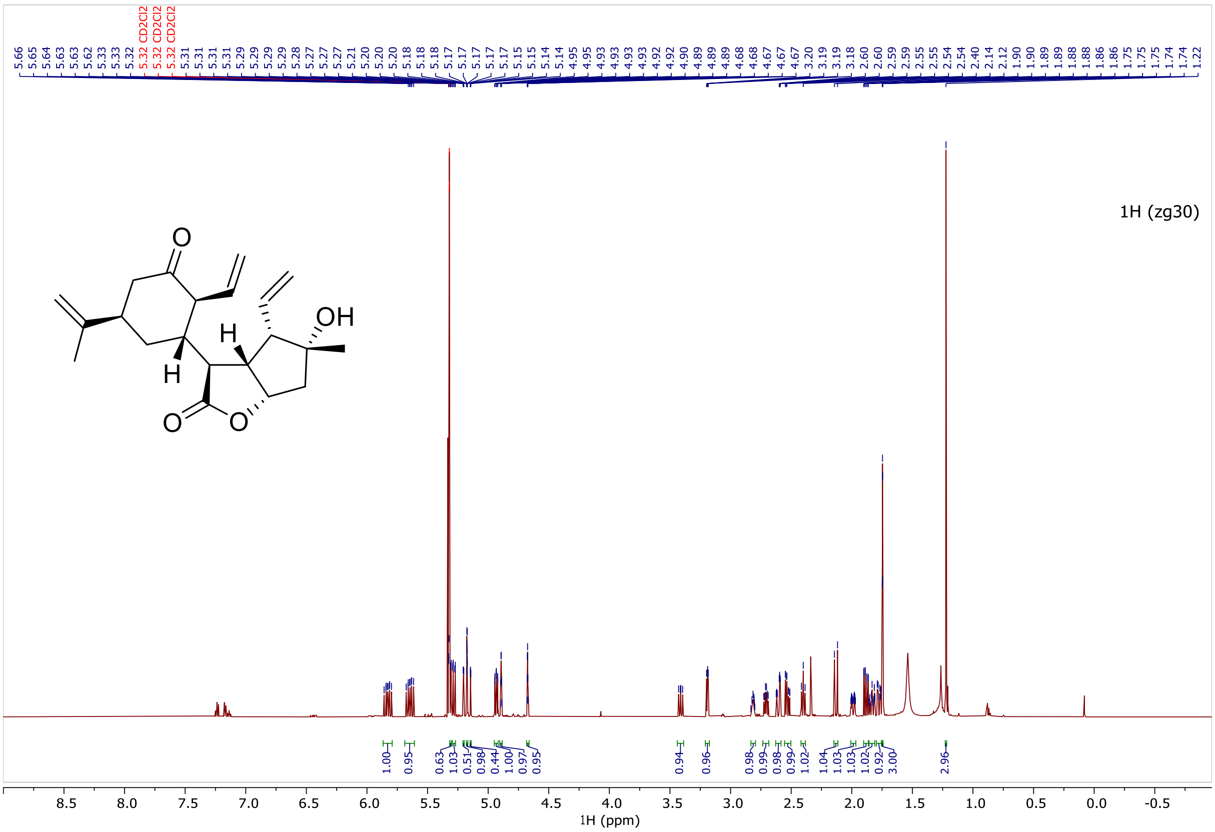

1D — lidla16304 (9874) — 5 mm, CD2Cl2, 0.9 mg — 1H (zg30) @ 298.0 K — AV600a, cryoTCI — 23.02.23 13:55

^{13}C NMR of 13 (101 MHz, CD_2Cl_2)

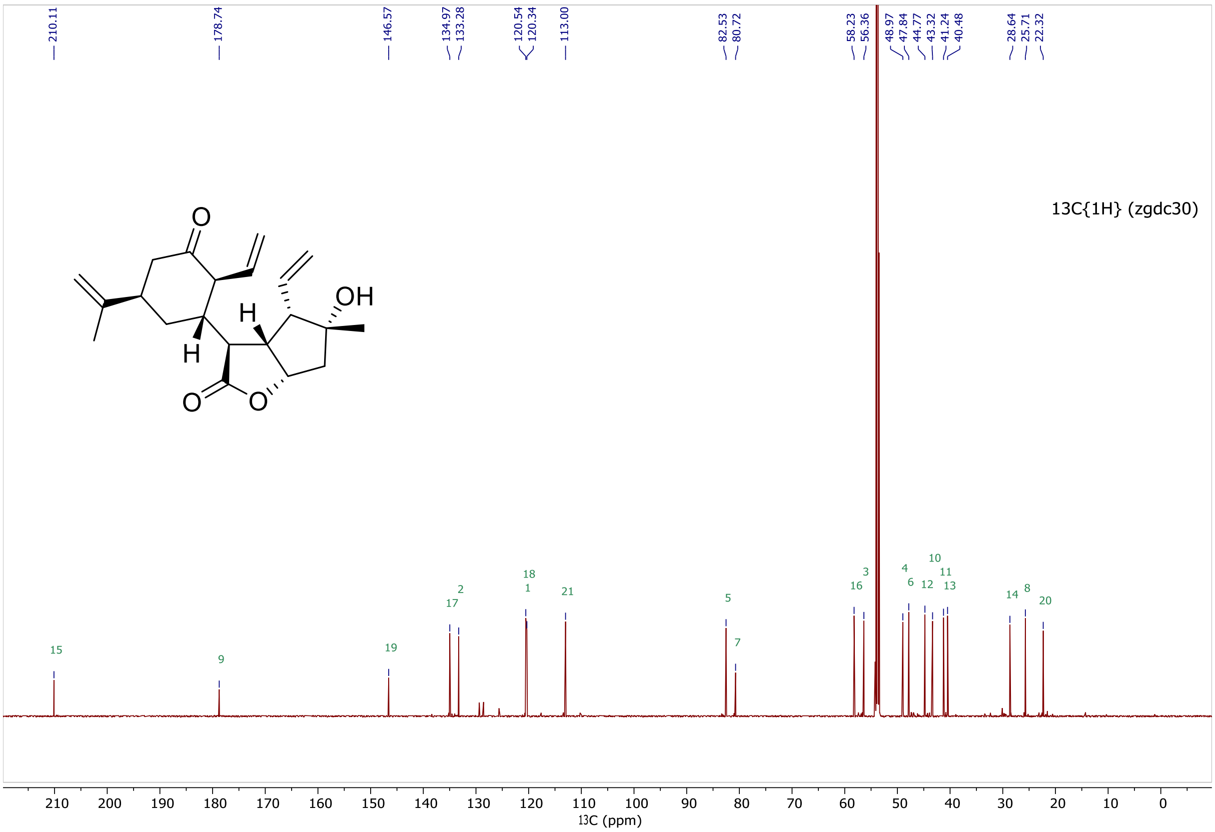

1D — lidla16304 (9874) — 5 mm, CD2Cl2, 0.9 mg — 13C{1H} (zgdc30) @ 298.0 K — AV600a, cryoTCI — 24.02.23 08:08

^{1}H NMR of 22 (400 MHz, CDCl$_3$)

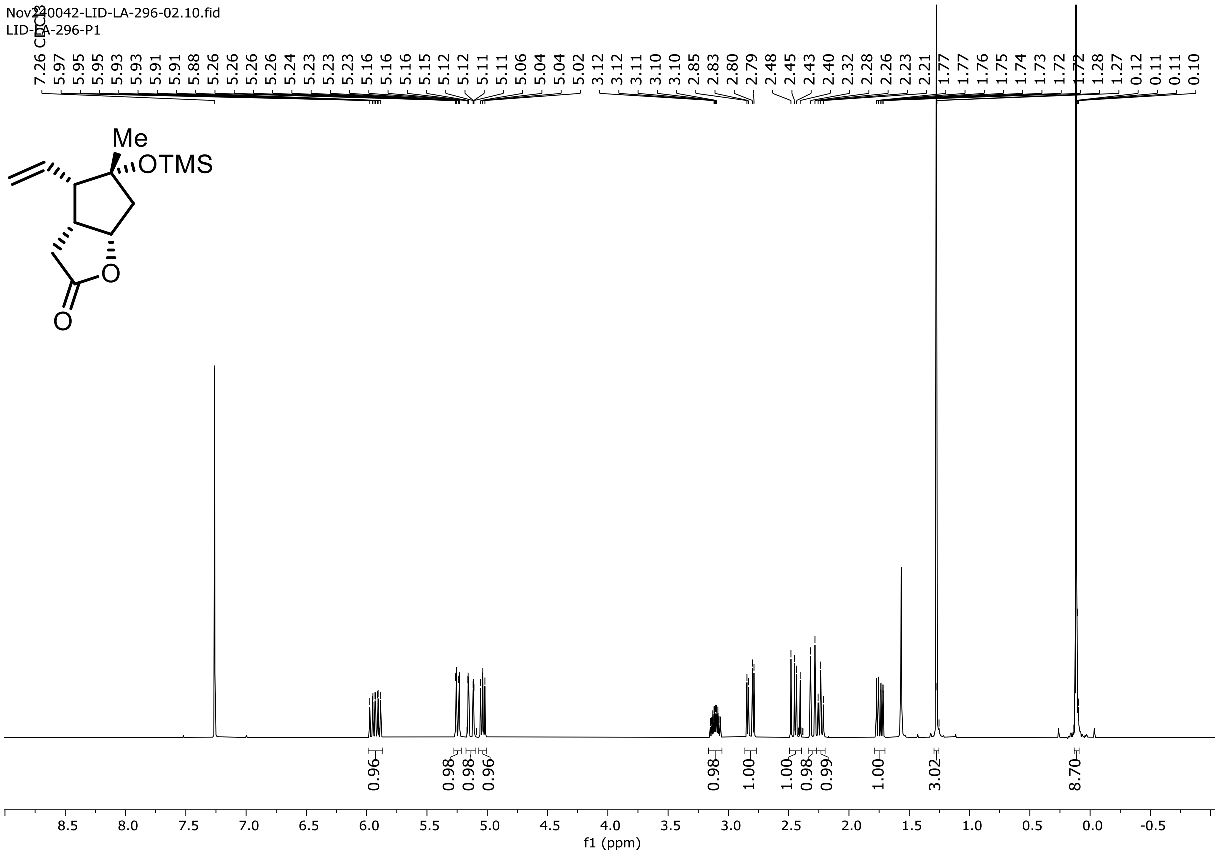

^{13}C NMR of 22 (101 MHz, CDCl$_3$)

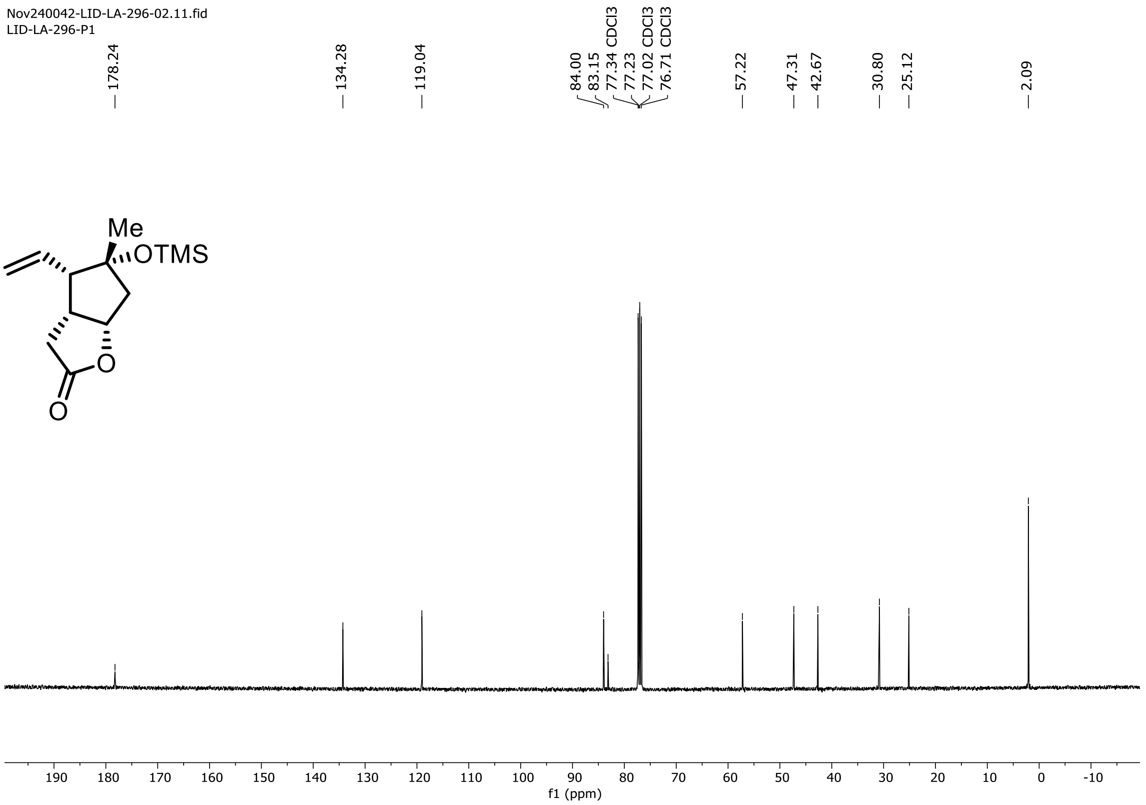

^{1}H NMR of 23 (400 MHz, $CDCl_3$)

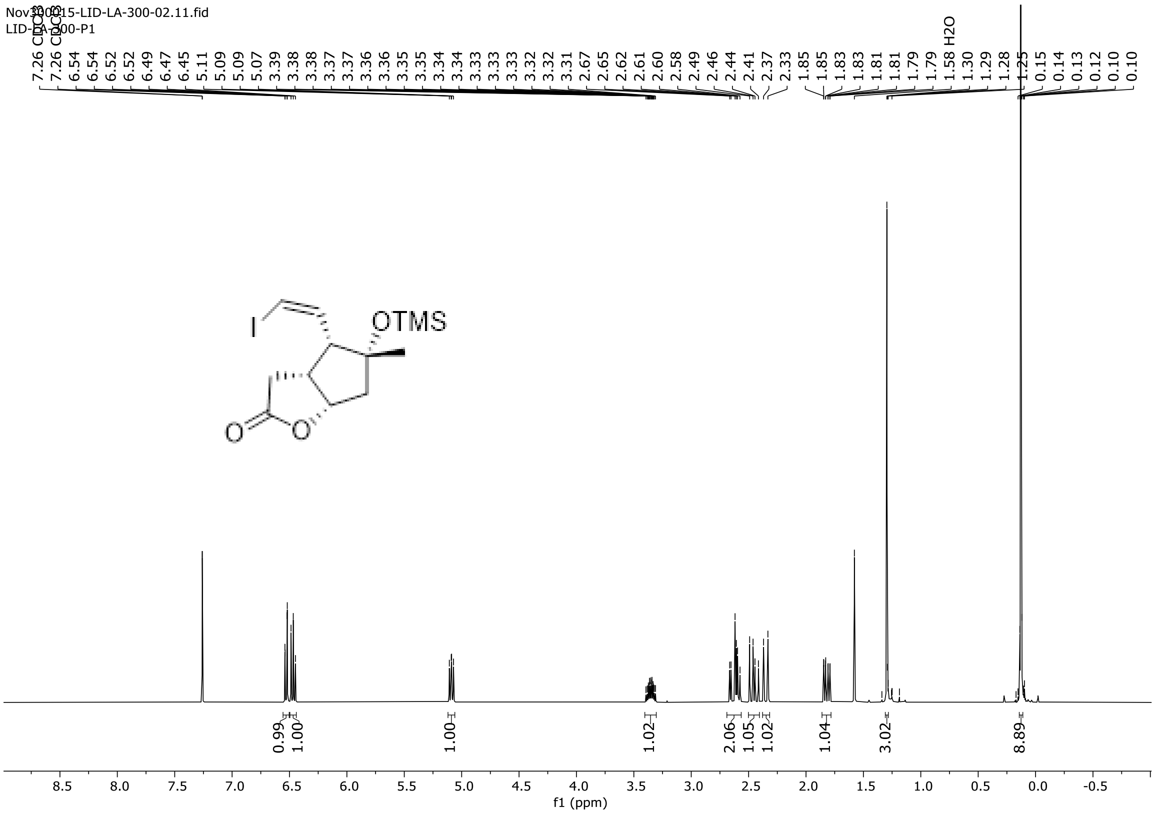

^{13}C NMR of 23 (101 MHz, $CDCl_3$)

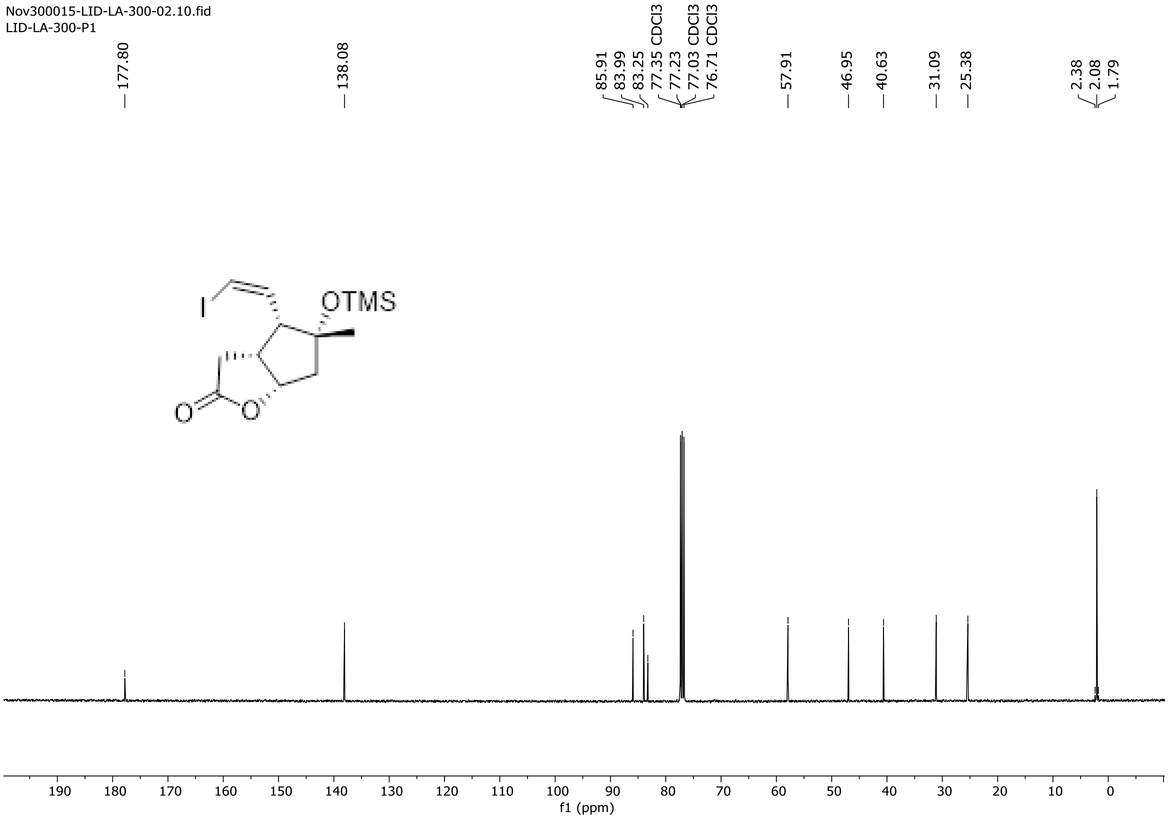

^{1}H NMR of 25 (600 MHz, $CDCl_3$)

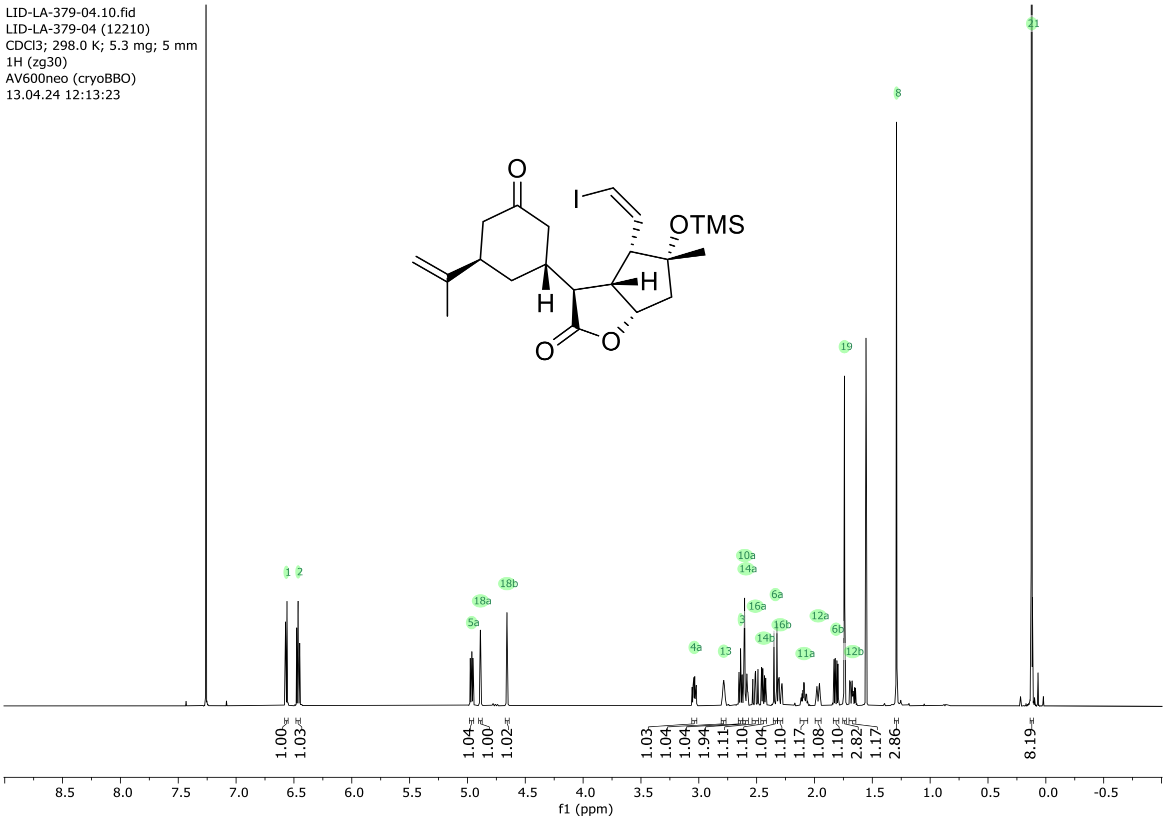

^{13}C NMR of 25 (151 MHz, $CDCl_3$)

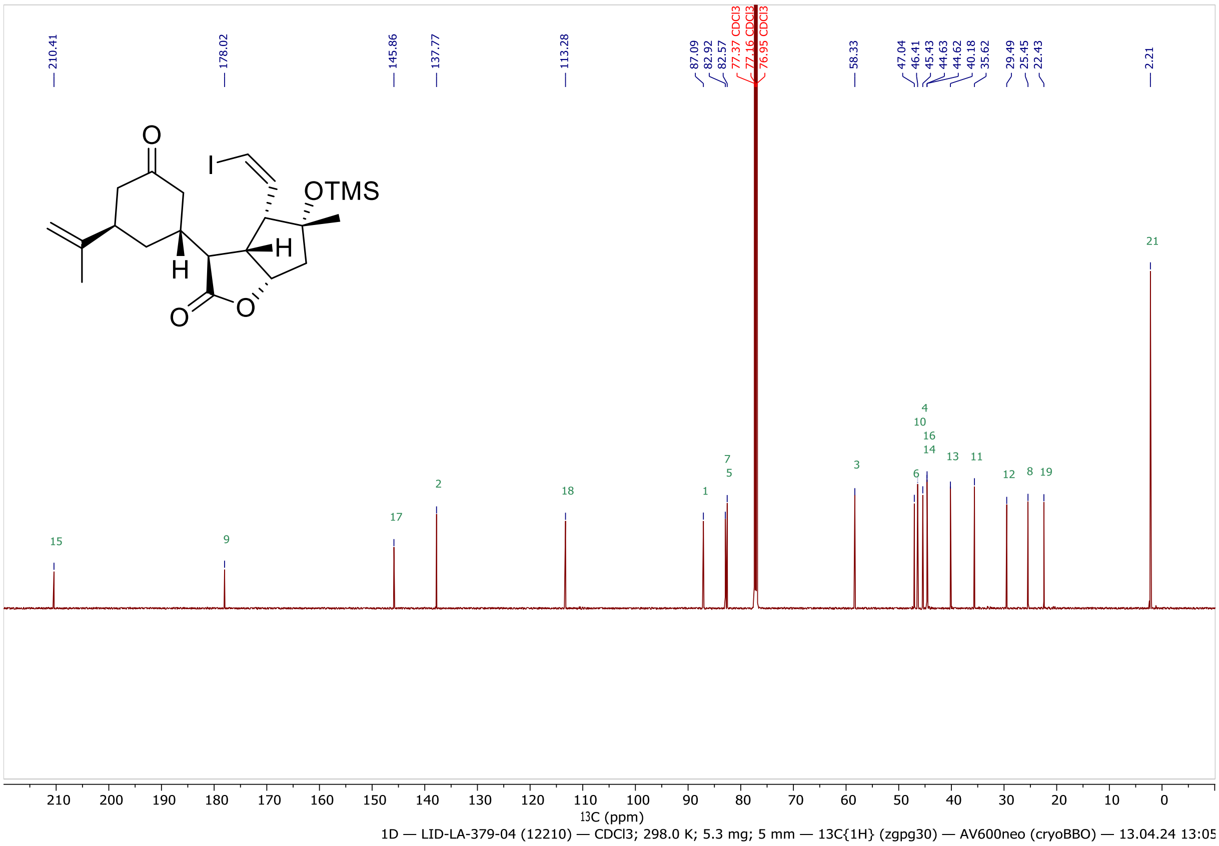

^{1}H COSY of 25 (600 MHz, $CDCl_3$)

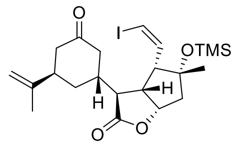

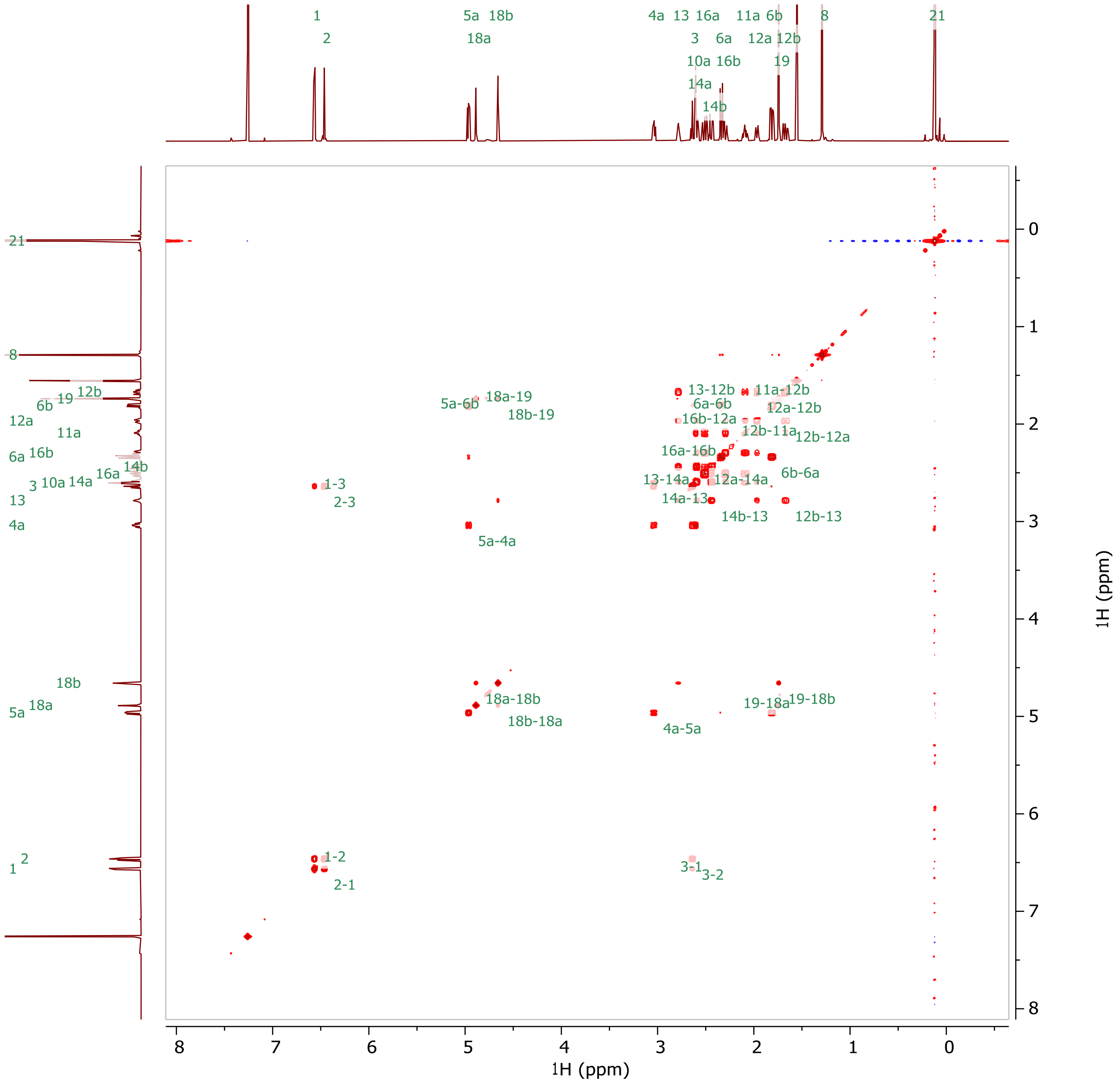

COSY — LID-LA-379-04 (12210) — CDCl3; 298.0 K; 5.3 mg; 5 mm — 1H (cosygpppqf) — AV600neo (cryoBBO) — 13.04.24 13:54

HSQC spectrum of 25

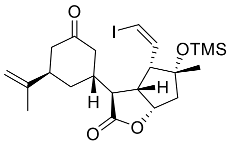

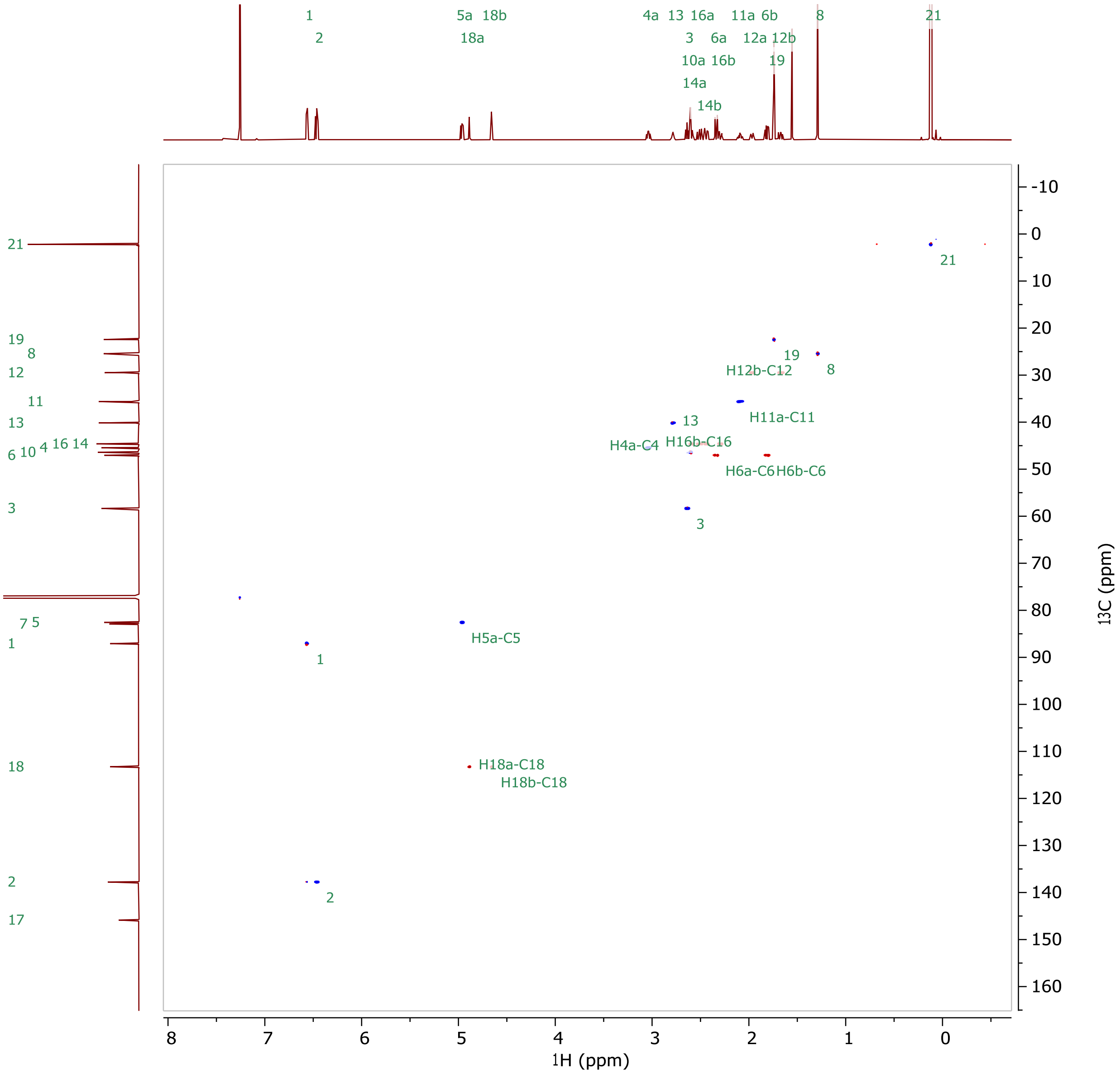

- LID-LA-379-04 (12210) — CDCl3; 298.0 K; 5.3 mg; 5 mm — 1H-13C (hsqcedetgpsisp2.3) — AV600neo (cryoBBO) — 13.04.24 13:1

HMBC spectrum of 25

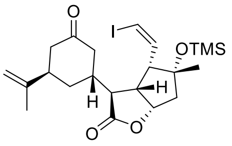

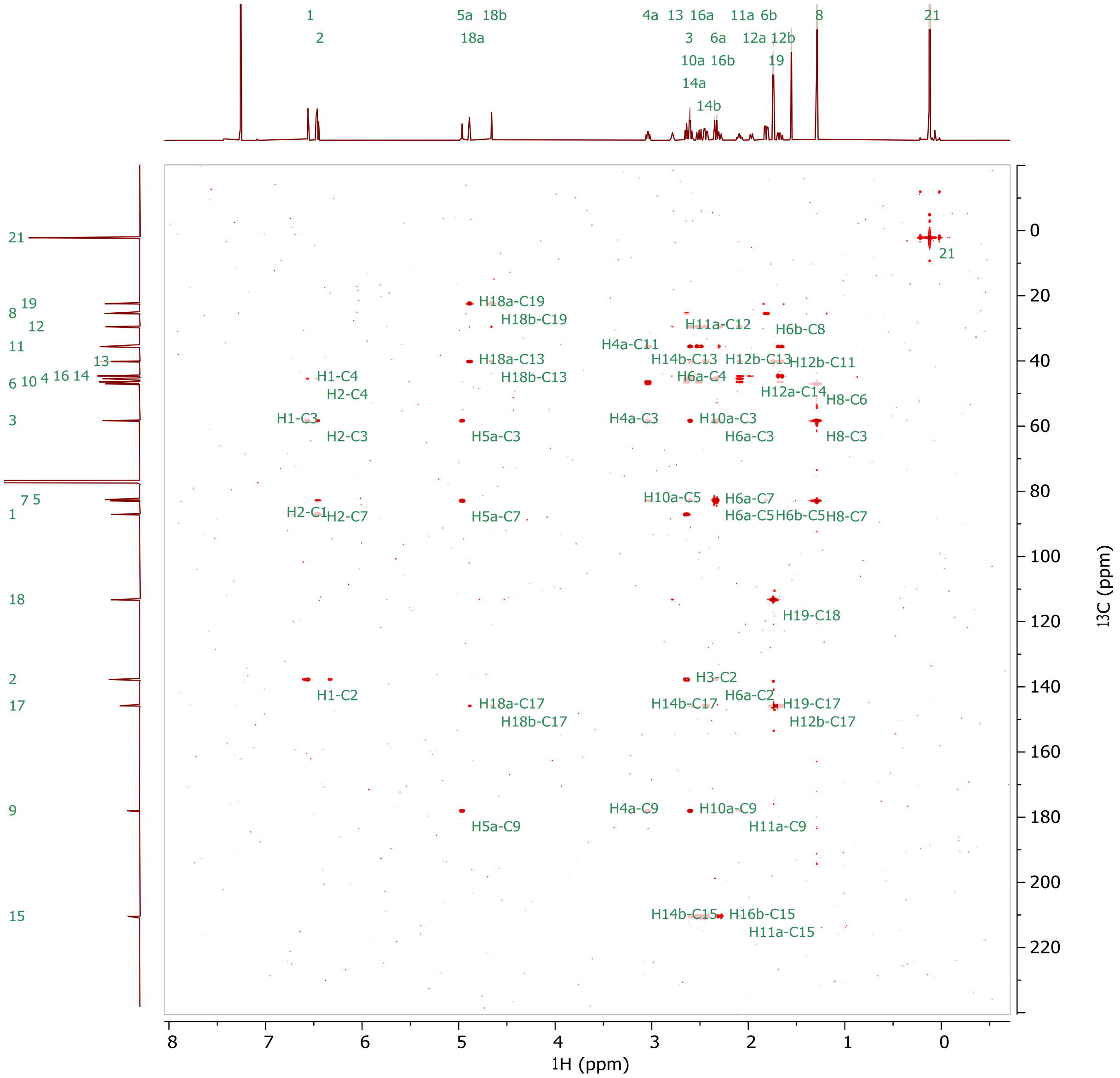

C — LID-LA-379-04 (12210) — CDCl3; 298.0 K; 5.3 mg; 5 mm — 1H-13C (hmbcetgpl3nd) — AV600neo (cryoBBO) — 13.04.24 13:41

NOESY spectrum of 25

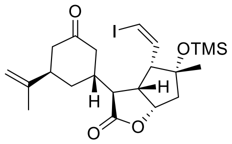

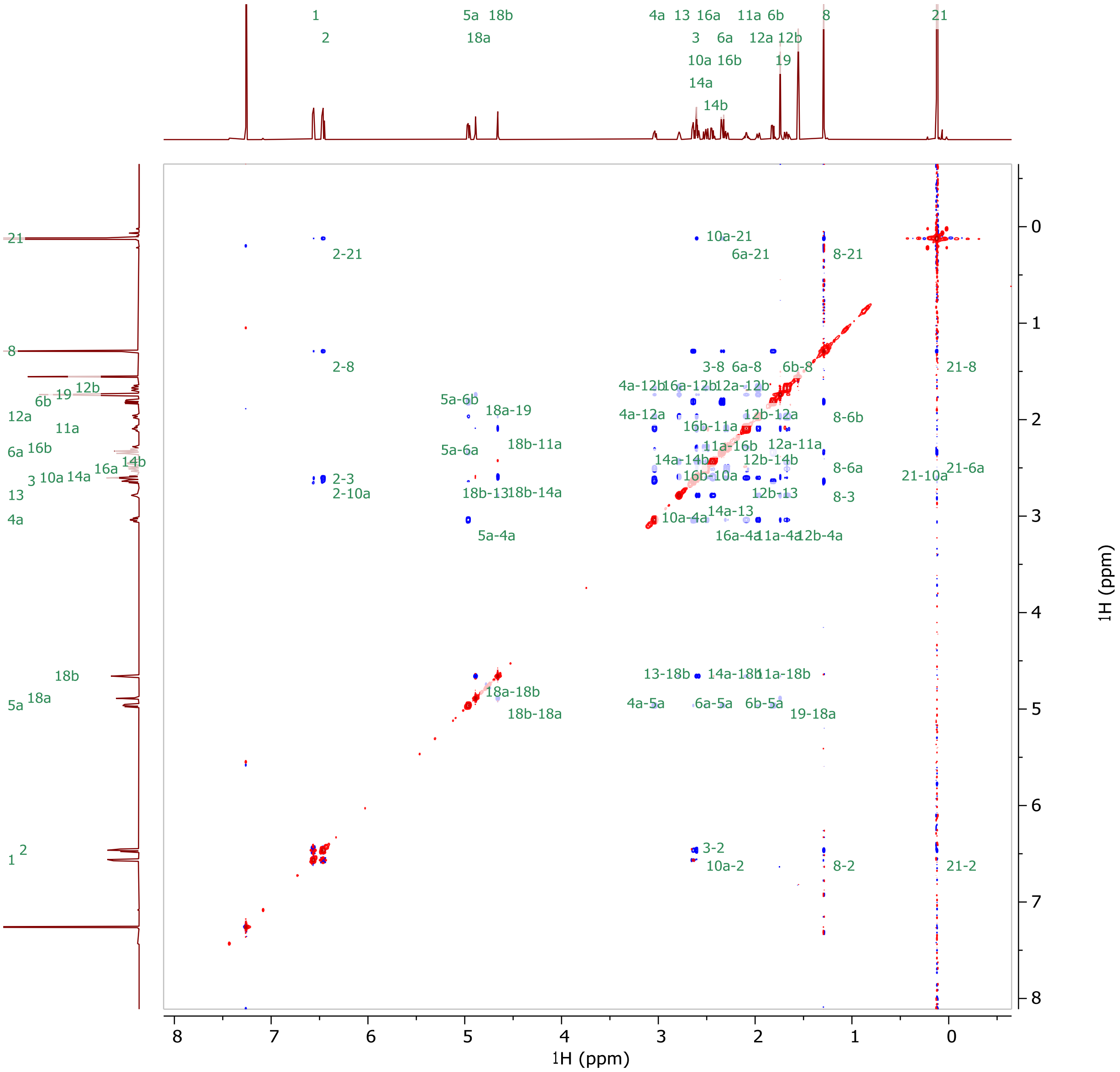

NOESY — LID-LA-379-04 (12210) — CDCl3; 298.0 K; 5.3 mg; 5 mm — 1H (noesygpphpp) — AV600neo (cryoBBO) — 13.04.24 15:51

^{1}H NMR of 26 (400 MHz, $CDCl_3$)

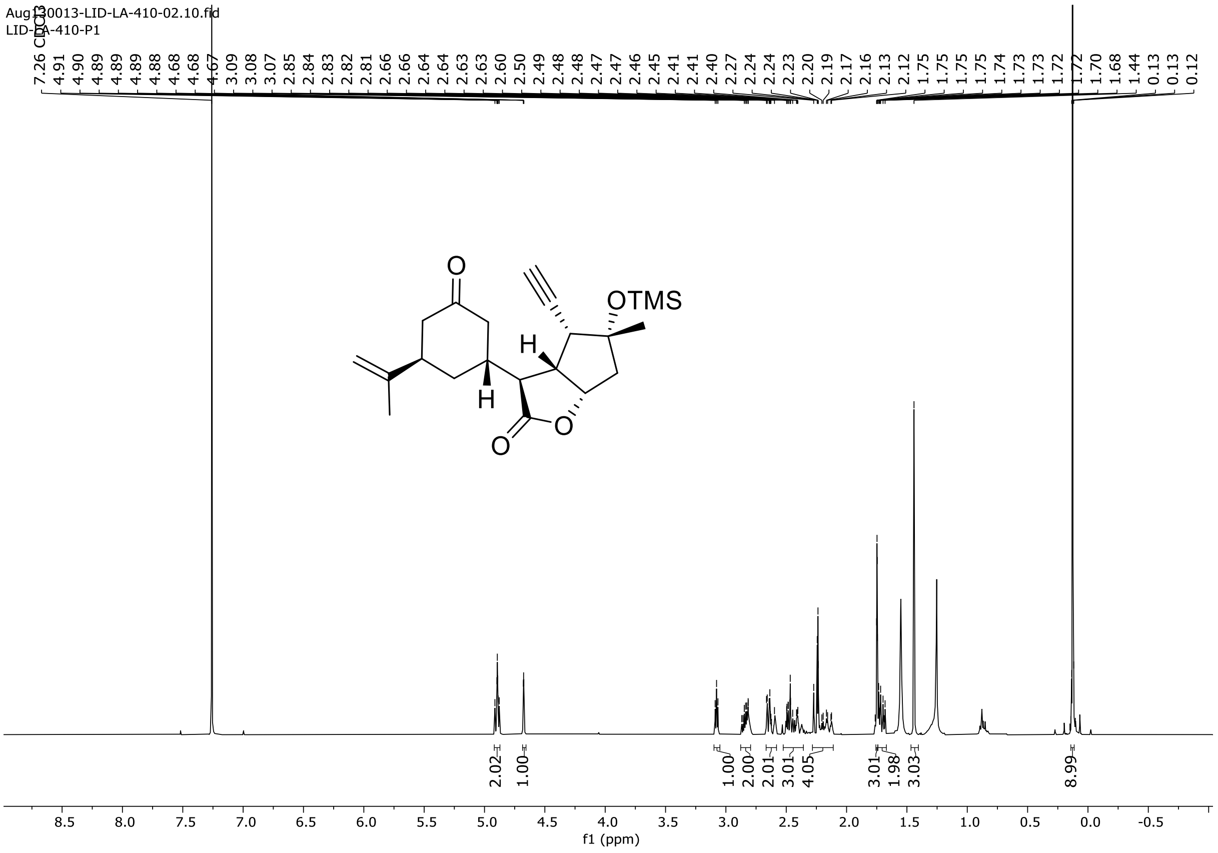

^{13}C NMR of 26 (101 MHz, $CDCl_3$)

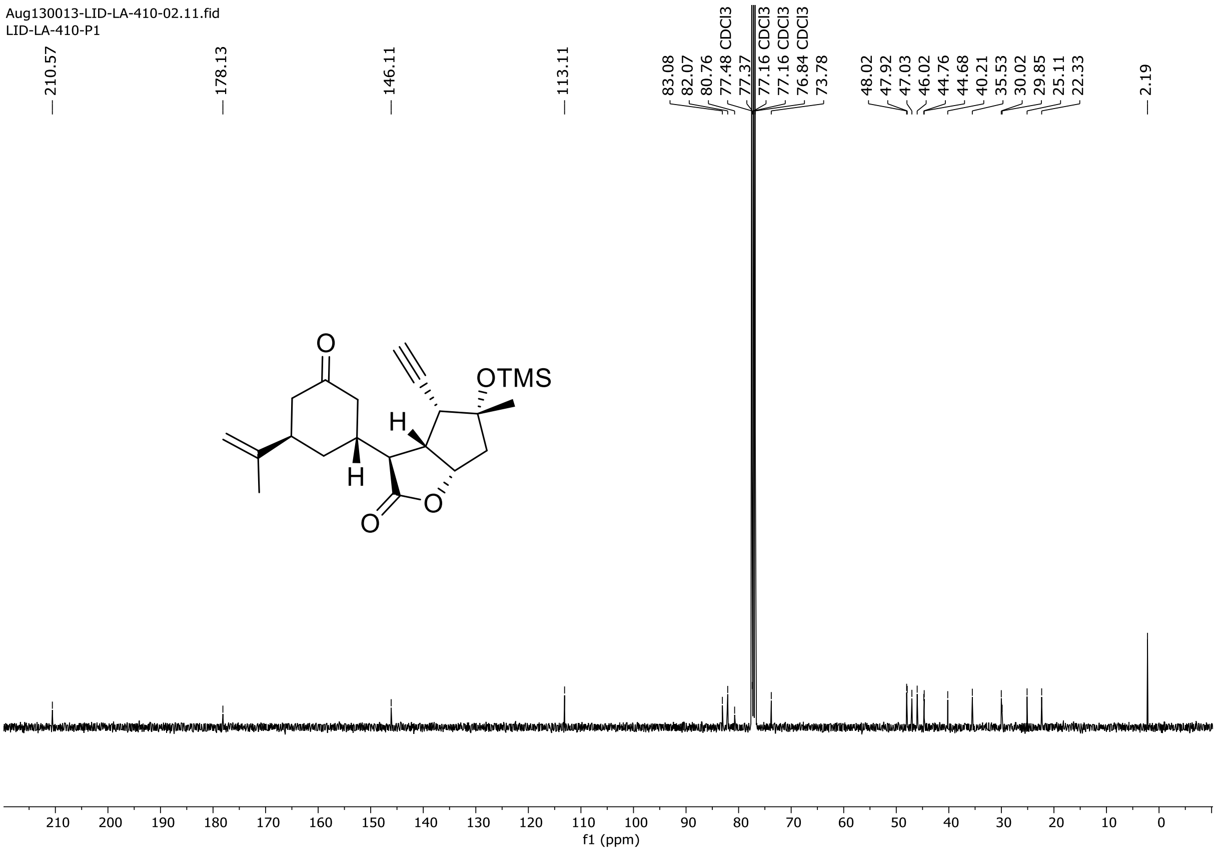

^{1}H NMR of 28 (600 MHz, $CDCl_3$)

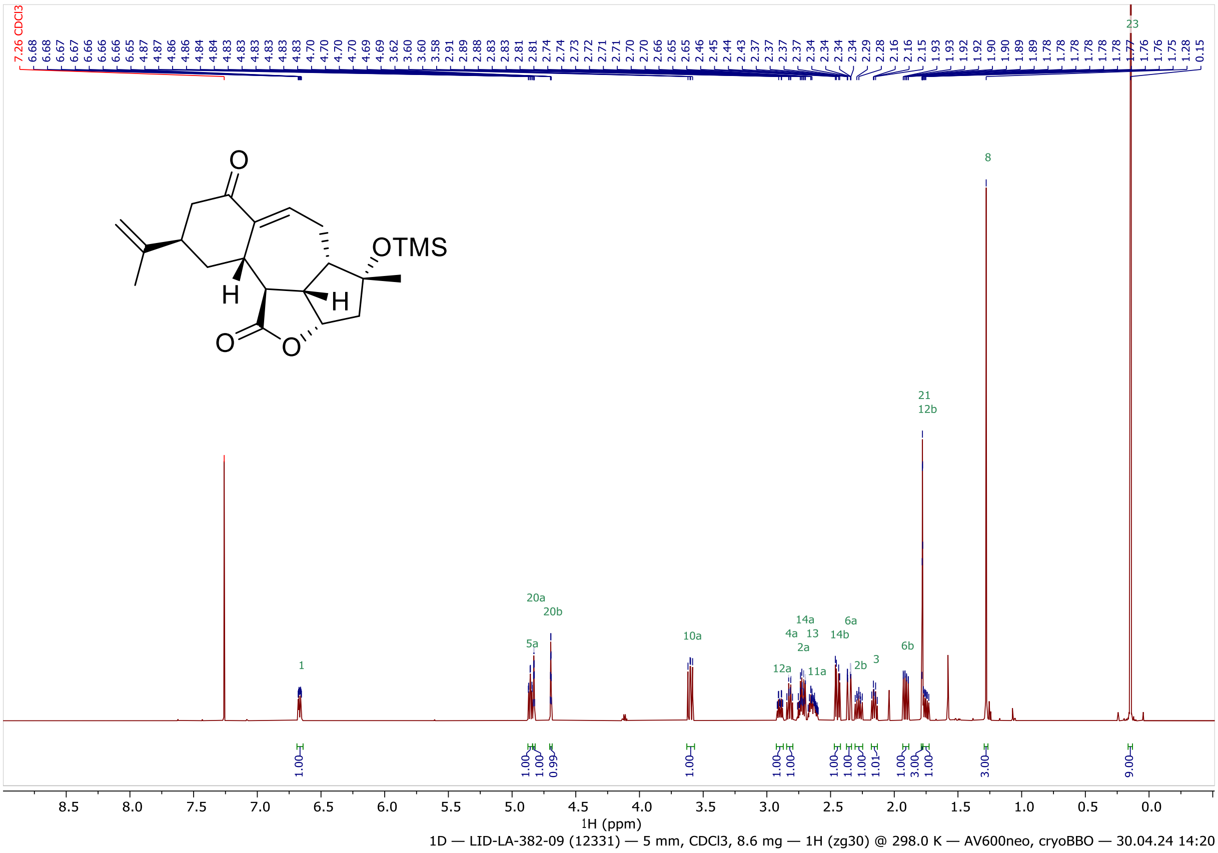

^{13}C NMR of 28 (151 MHz, $CDCl_3$)

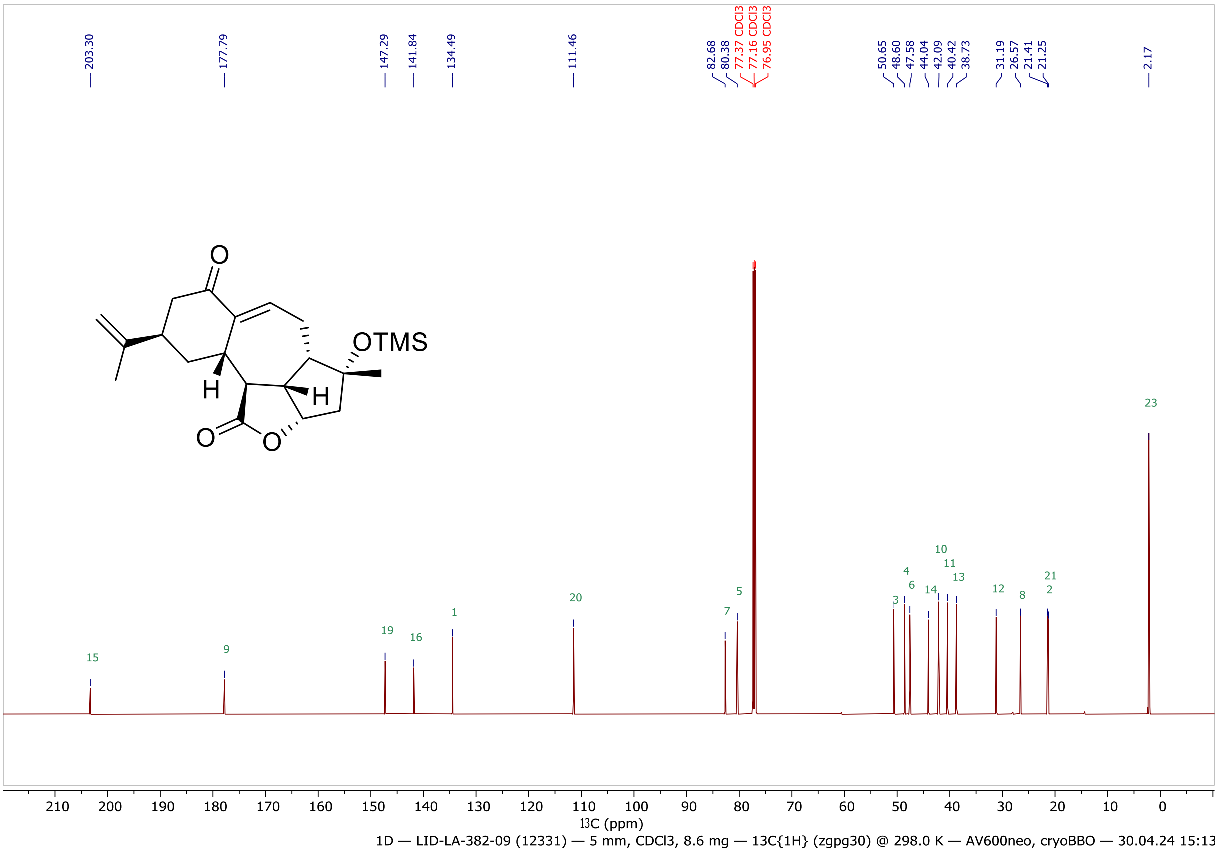

^{1}H COSY spectrum of 28 (600 MHz, $CDCl_3$)

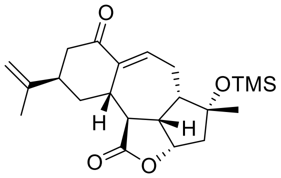

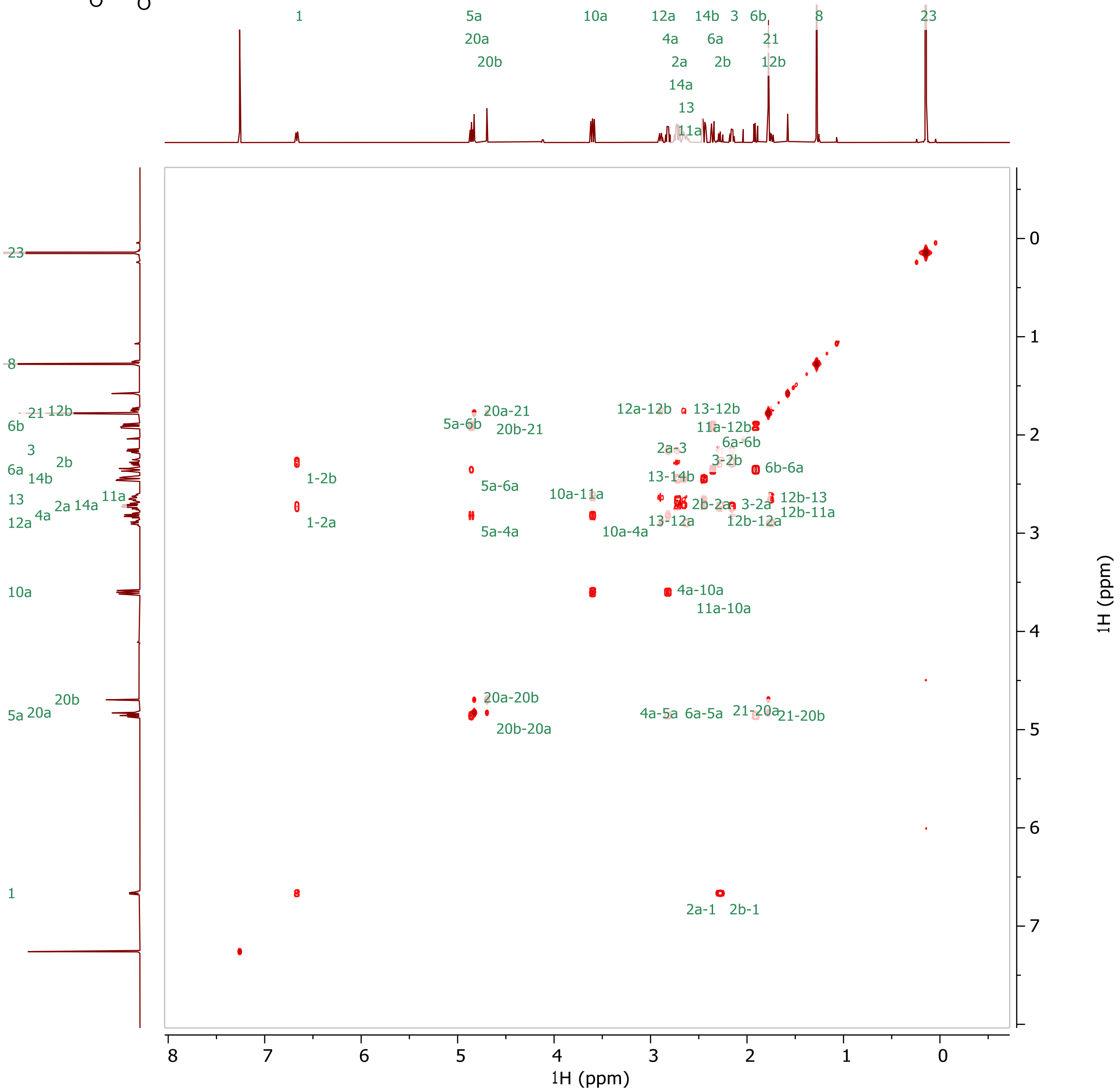

COSY — LID-LA-382-09 (12331) — 5 mm, CDCl3, 8.6 mg — 1H (cosygpppqf) @ 298.0 K — AV600neo, cryoBBO — 30.04.24 15:47

HSQC spectrum of 28

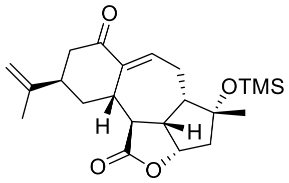

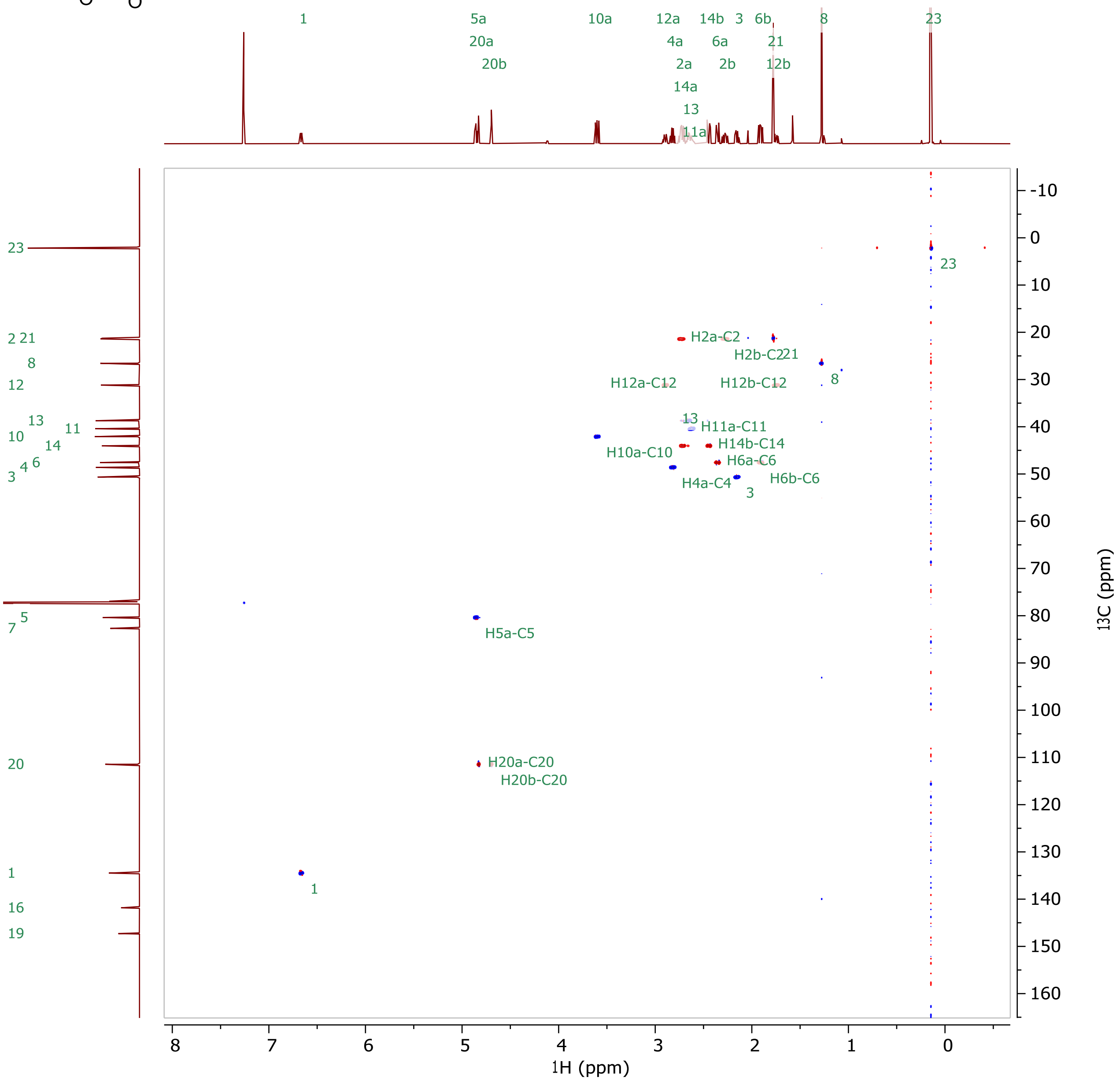

LID-LA-382-09 (12331) — 5 mm, CDCl3, 8.6 mg — 1H-13C (hsqcedetgpsisp2.3) @ 298.0 K — AV600neo, cryoBBO — 30.04.24 15:2

HMBC spectrum of 28

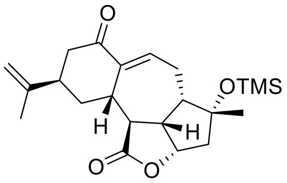

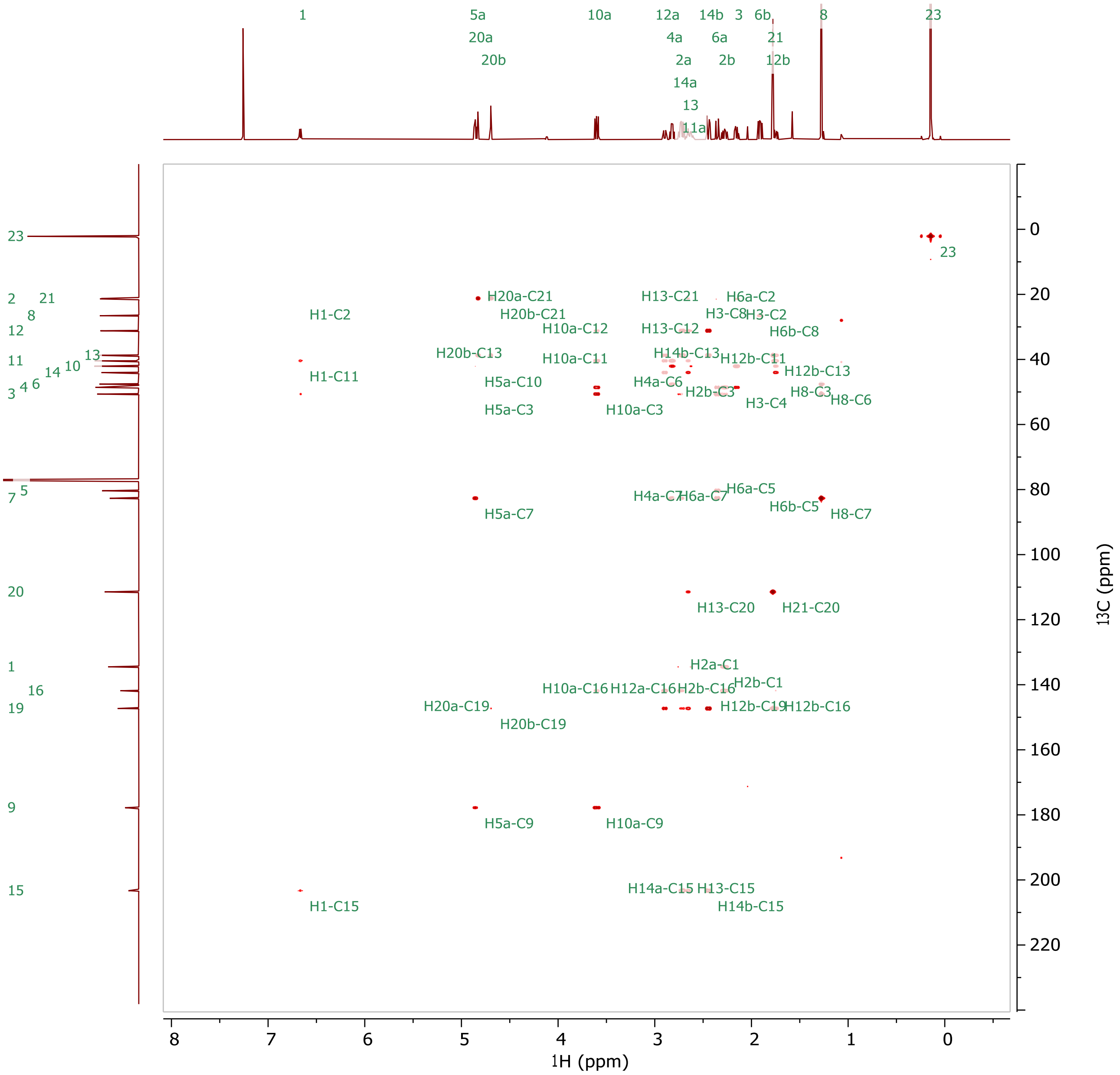

NOESY spectrum of 28

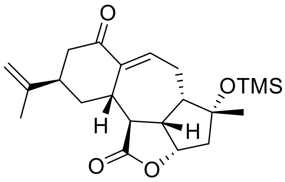

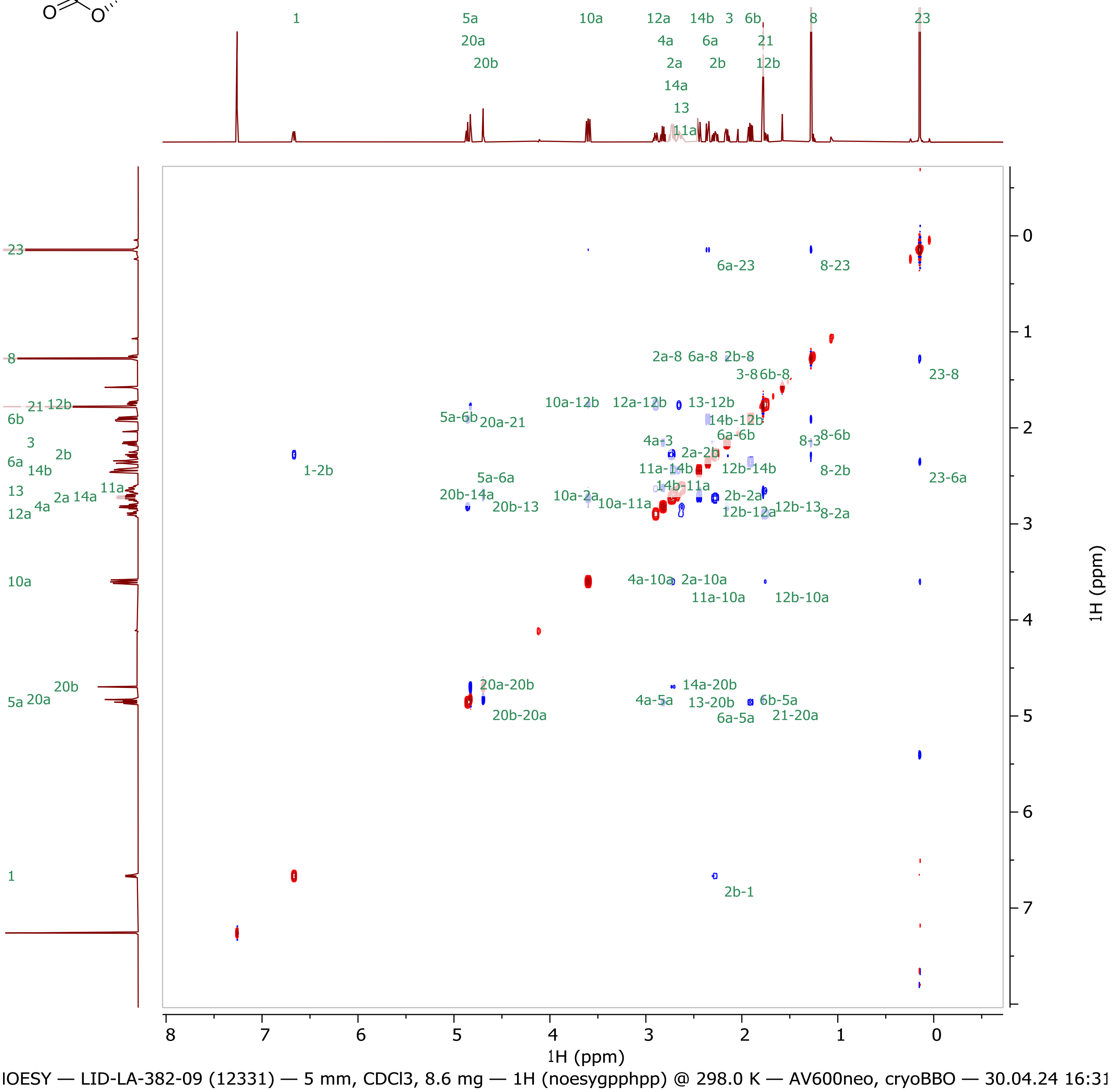

^{1}H NMR of dimer 29 (400 MHz, $CDCl_3$)

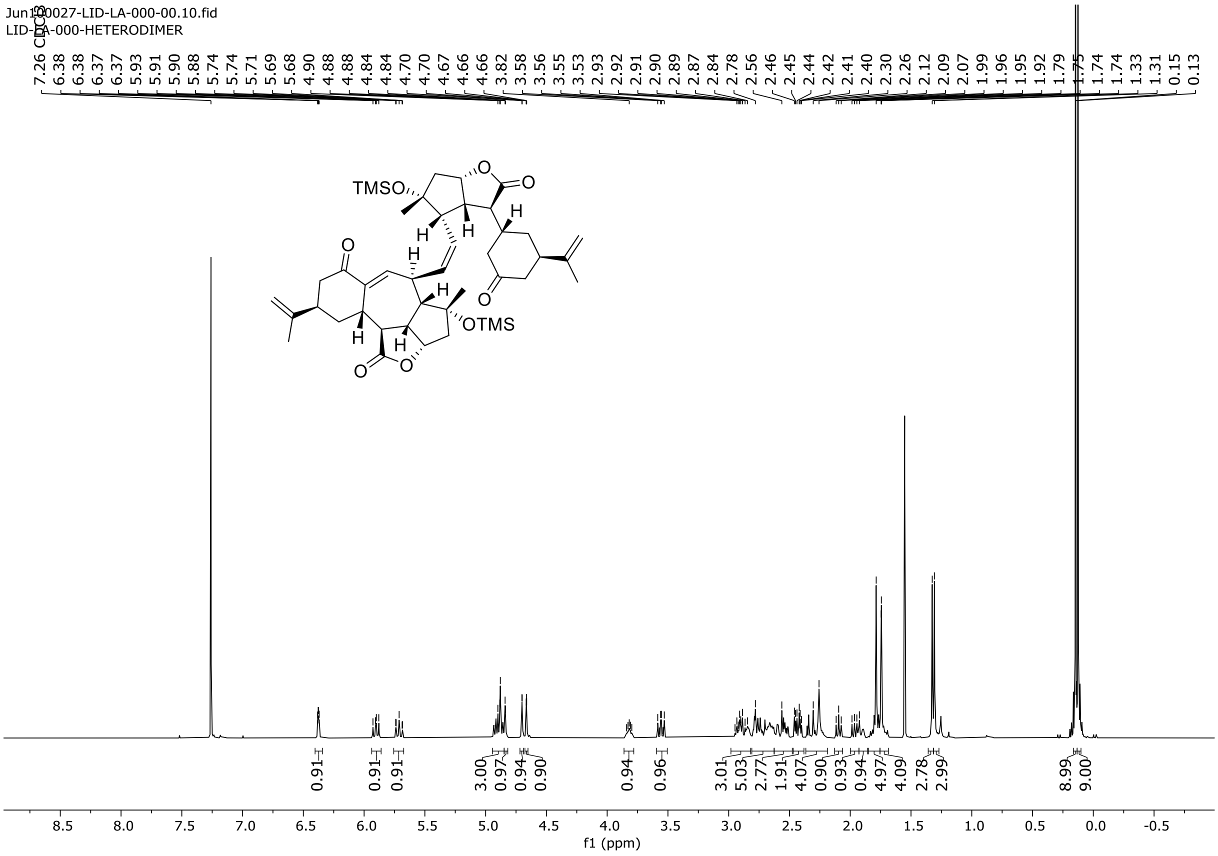

^{13}C NMR of dimer 29 (101 MHz, $CDCl_3$)

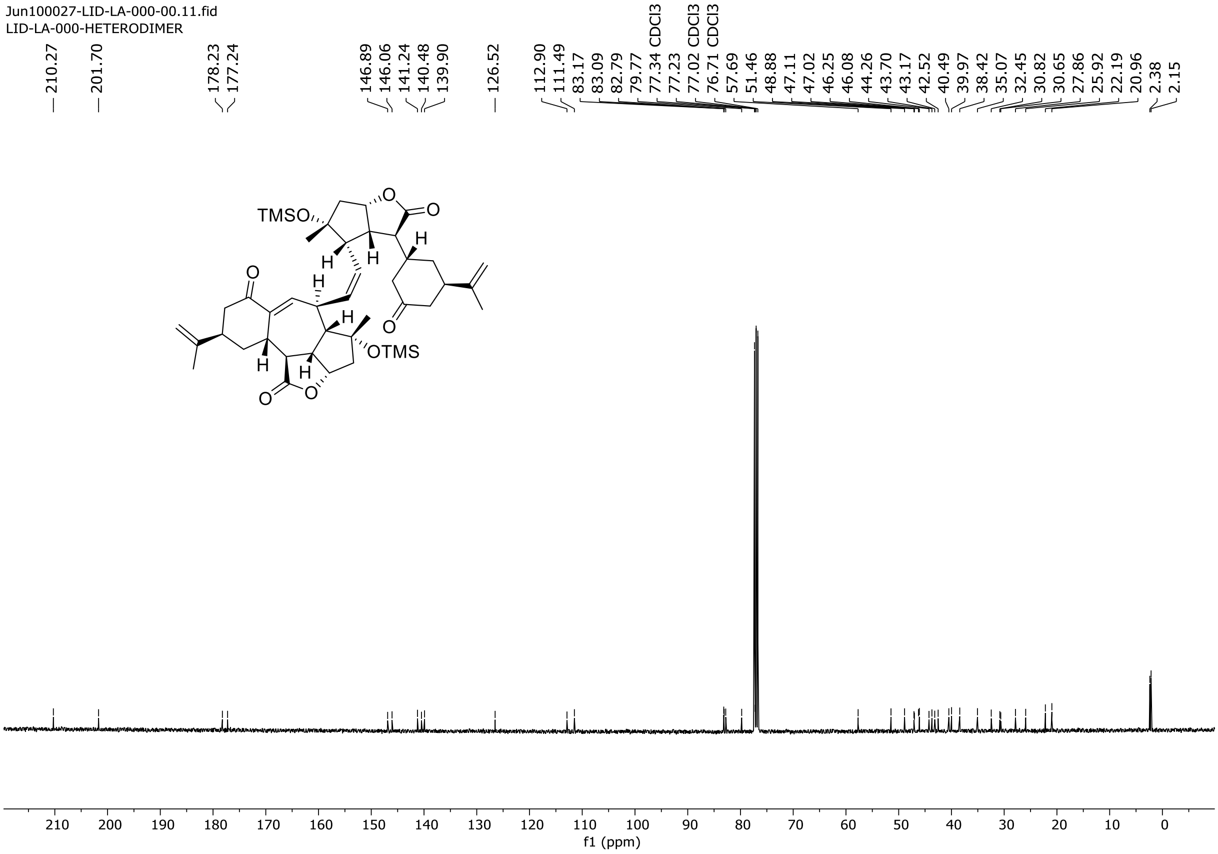

^{1}H NMR of S9 (400 MHz, $CDCl_3$)

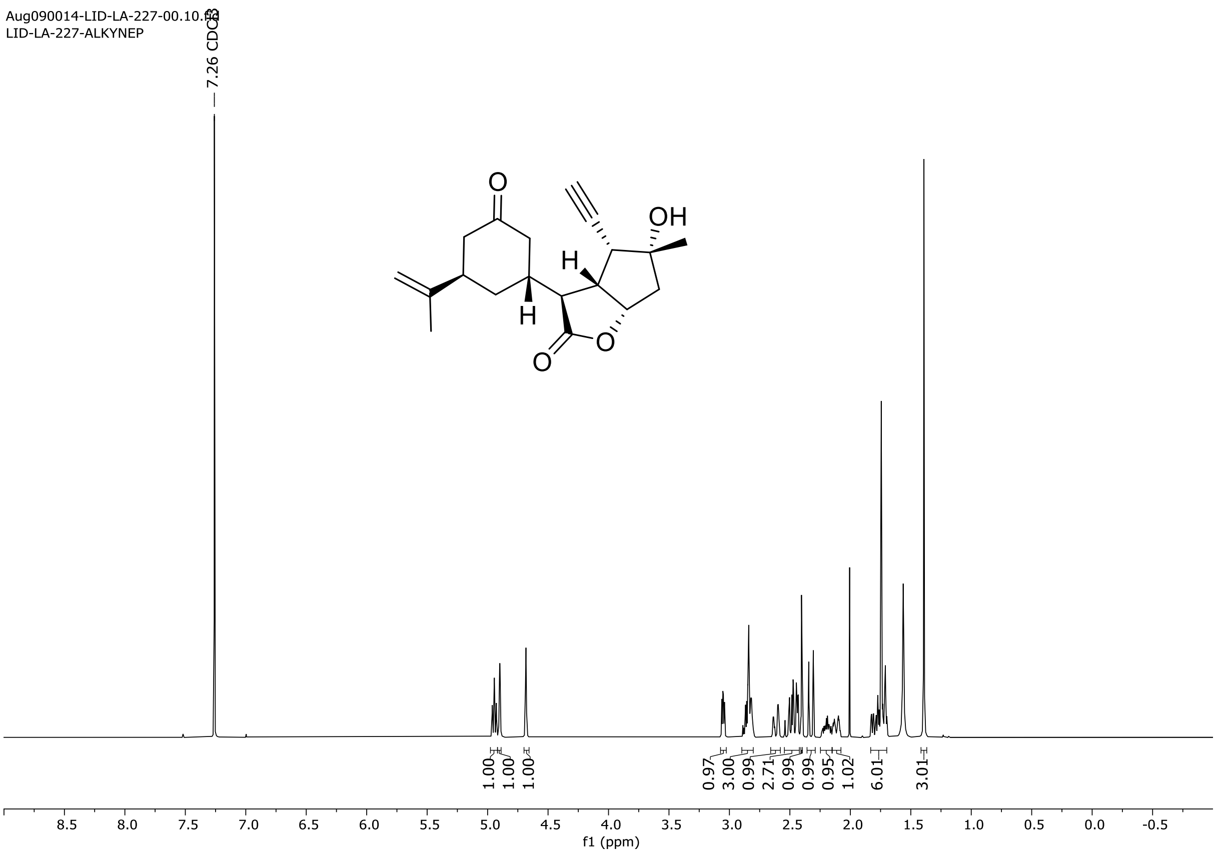

^{13}C NMR of S9 (101 MHz, $CDCl_3$)

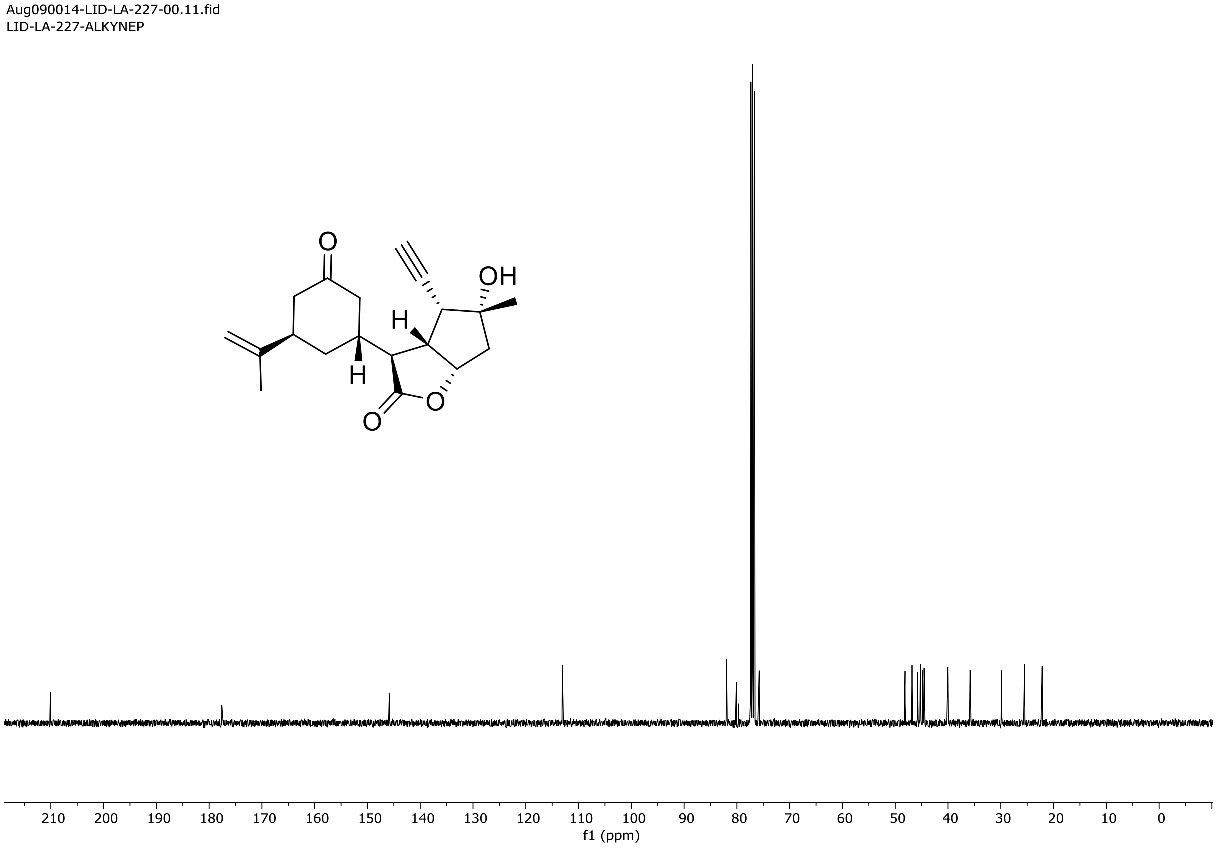

^{1}H NMR of S10 (400 MHz, $CDCl_3$)

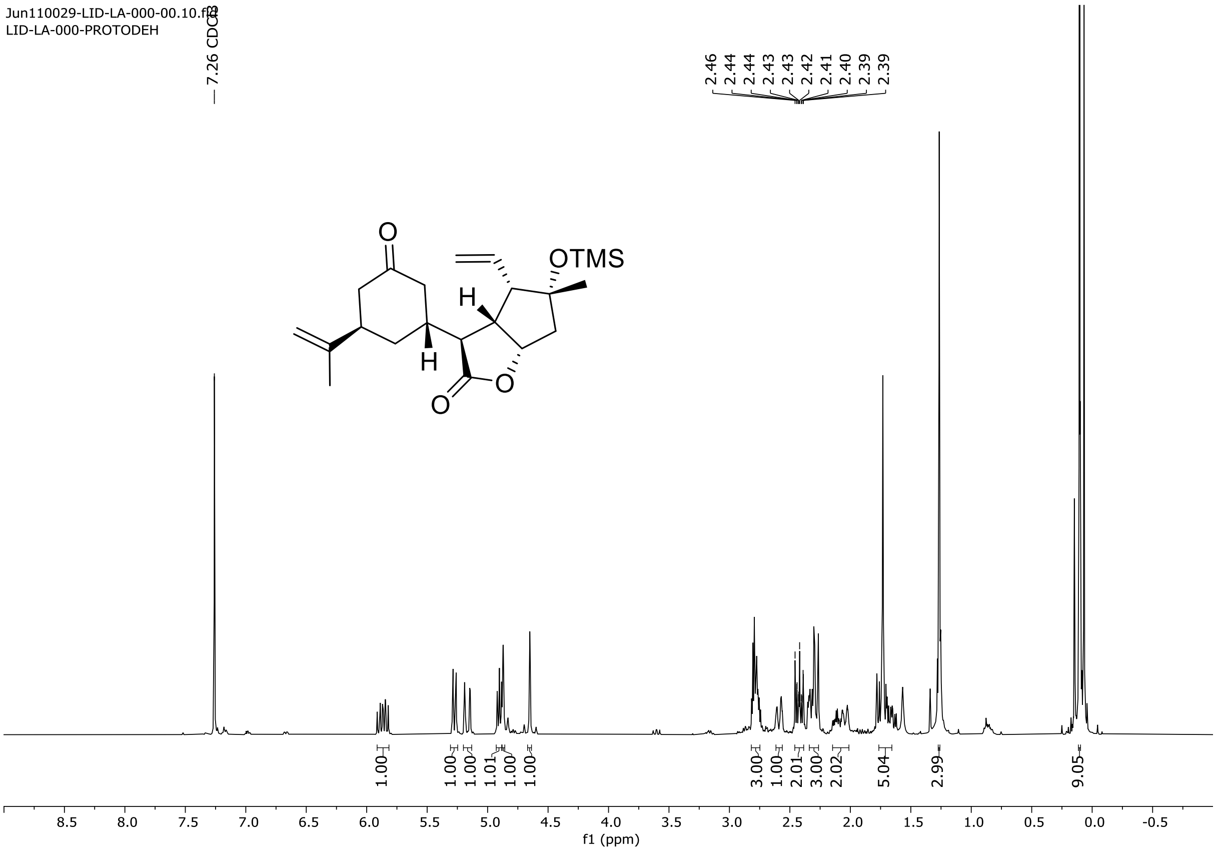

^{13}C NMR of S10 (101 MHz, $CDCl_3$)

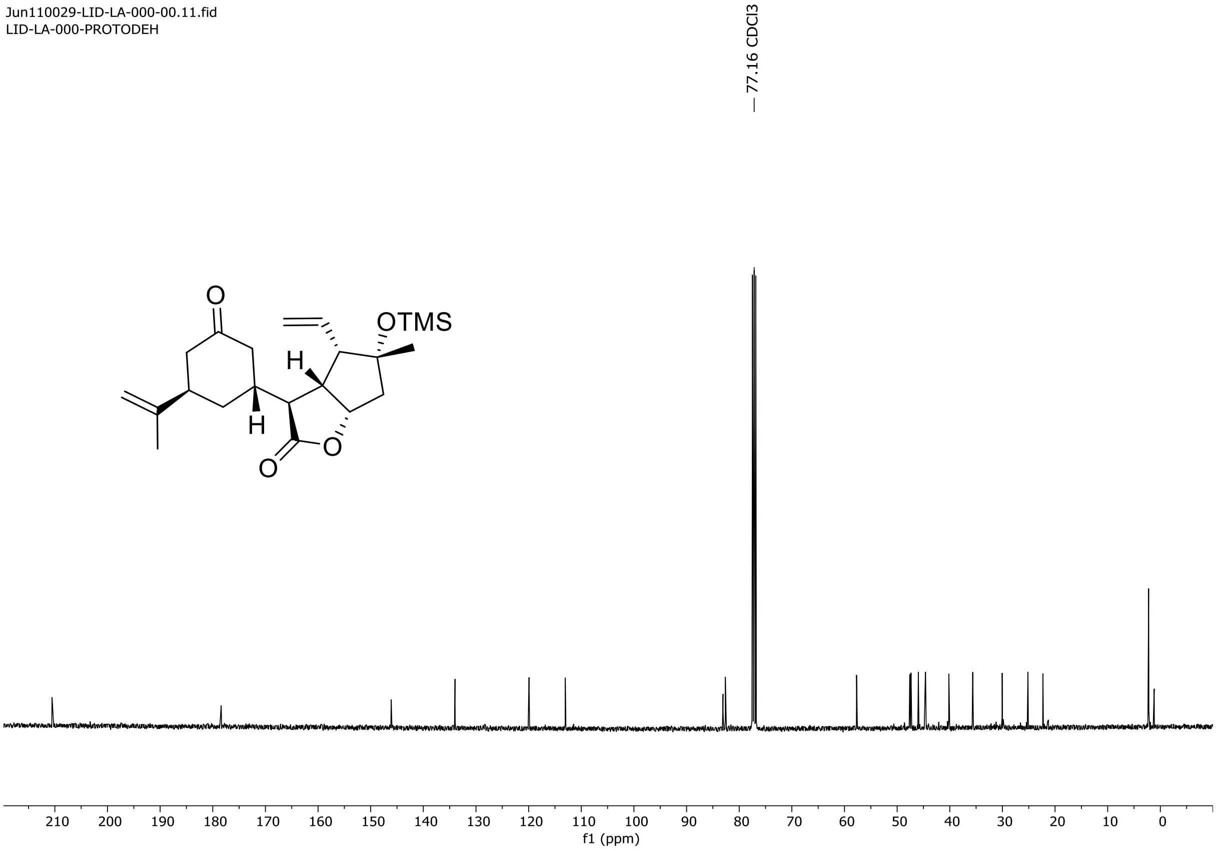

^{1}H NMR of S11 (400 MHz, $CDCl_3$)

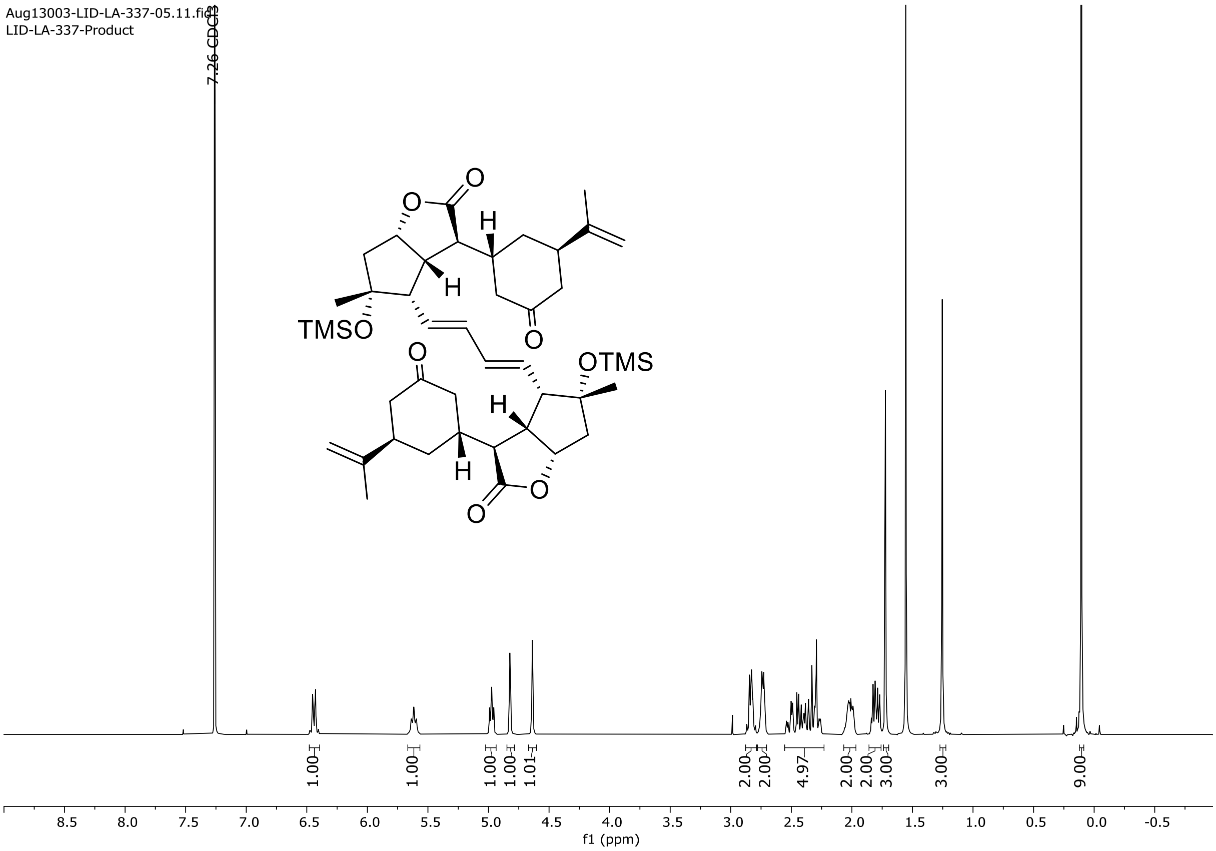

^{13}C NMR of S11 (101 MHz, $CDCl_3$)

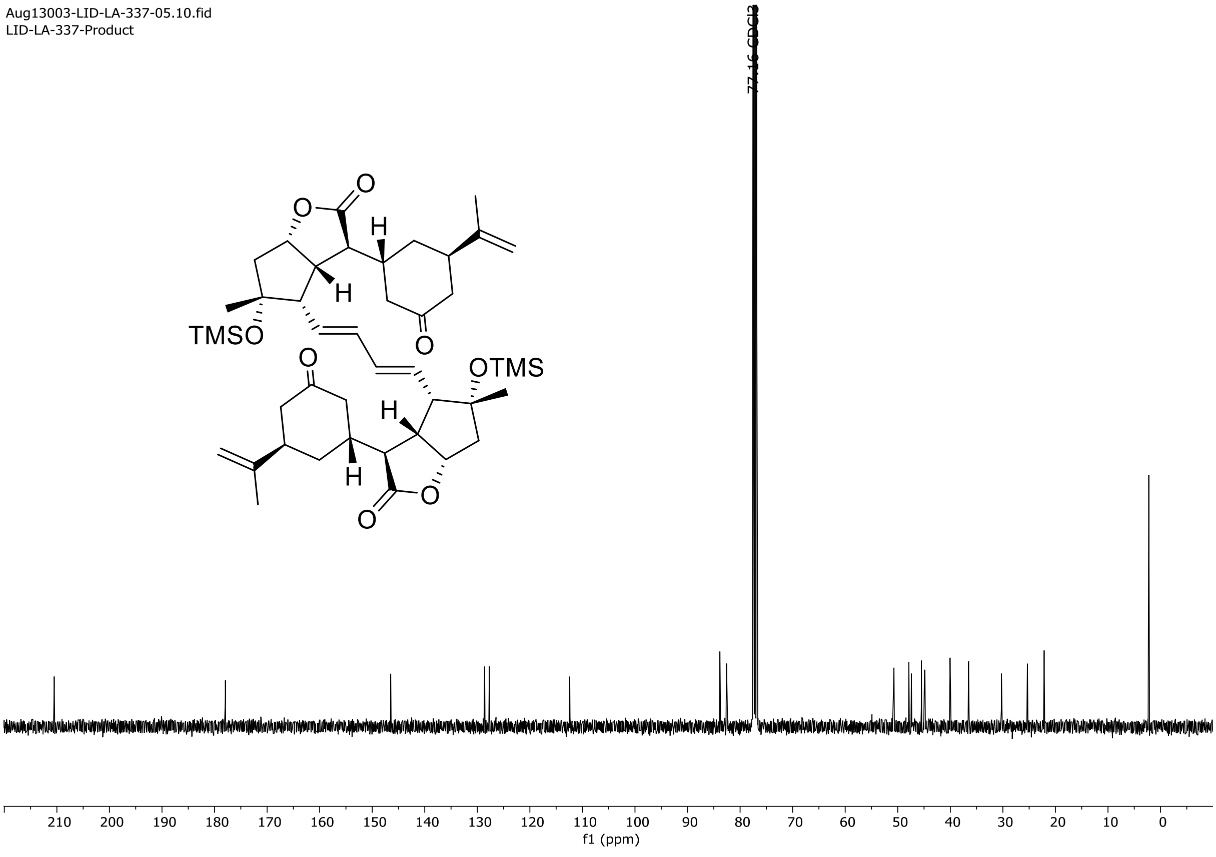

^{1}H NMR of 30 (600 MHz, CD_2Cl_2)

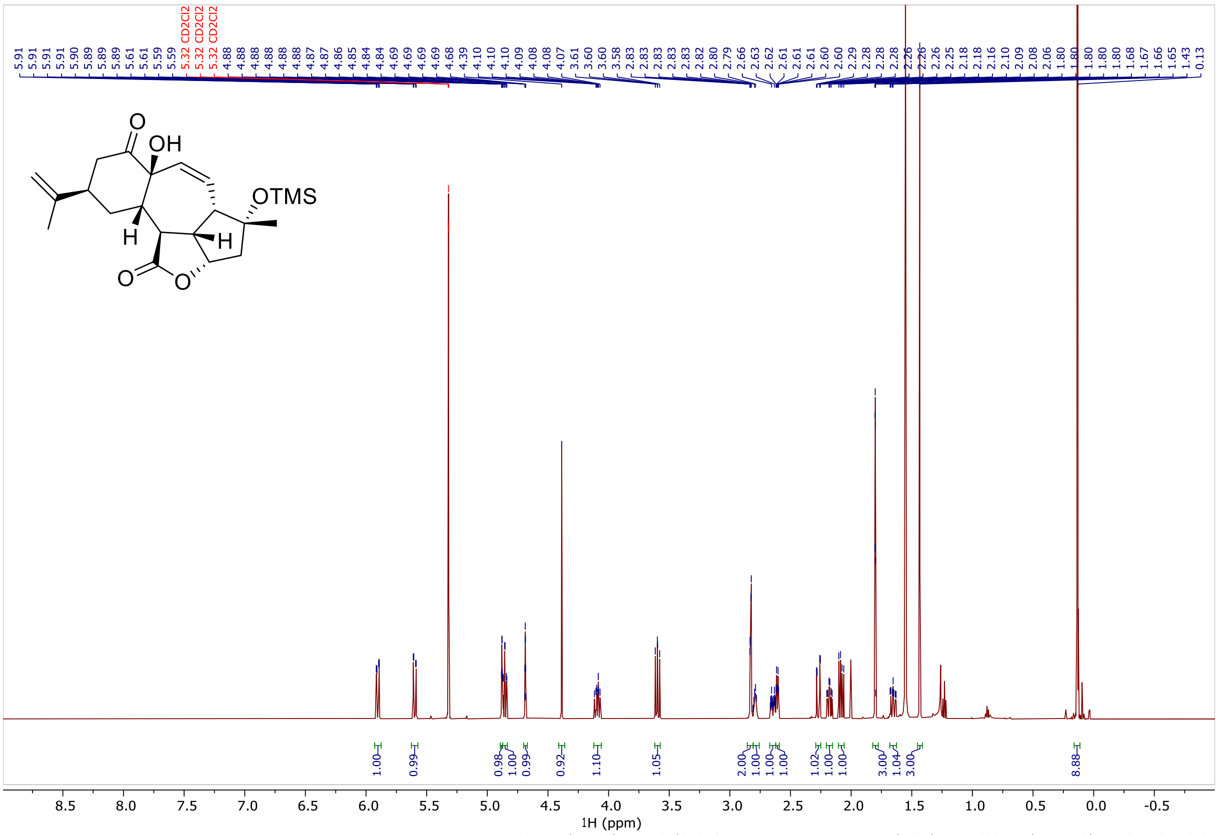

1D — LID-LA-365-02 (12139) — CD2Cl2; 298.0 K; 7.9 mg; 5 mm — 1H (zg30) — AV600neo (cryoBBO) — 03.04.24 03:36

^{13}C NMR of 30 (151 MHz, CD_2Cl_2)

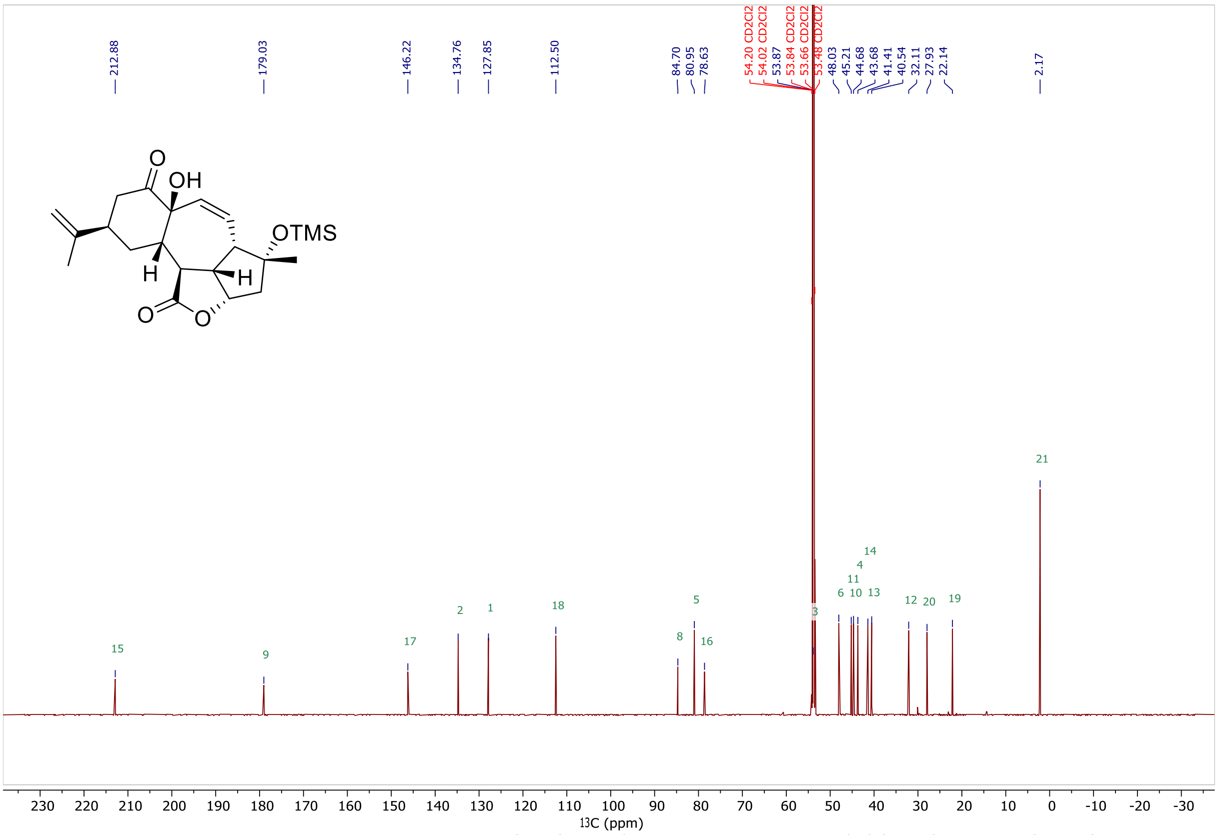

1D — LID-LA-365-02 (12139) — CD2Cl2; 298.0 K; 7.9 mg; 5 mm — 13C{1H} (zgpg30) — AV600neo (cryoBBO) — 03.04.24 04:2[illegible]

^{1}H COSY spectrum of 30 (600 MHz, CD_2Cl_2)

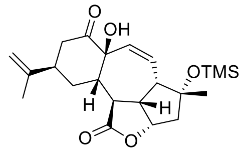

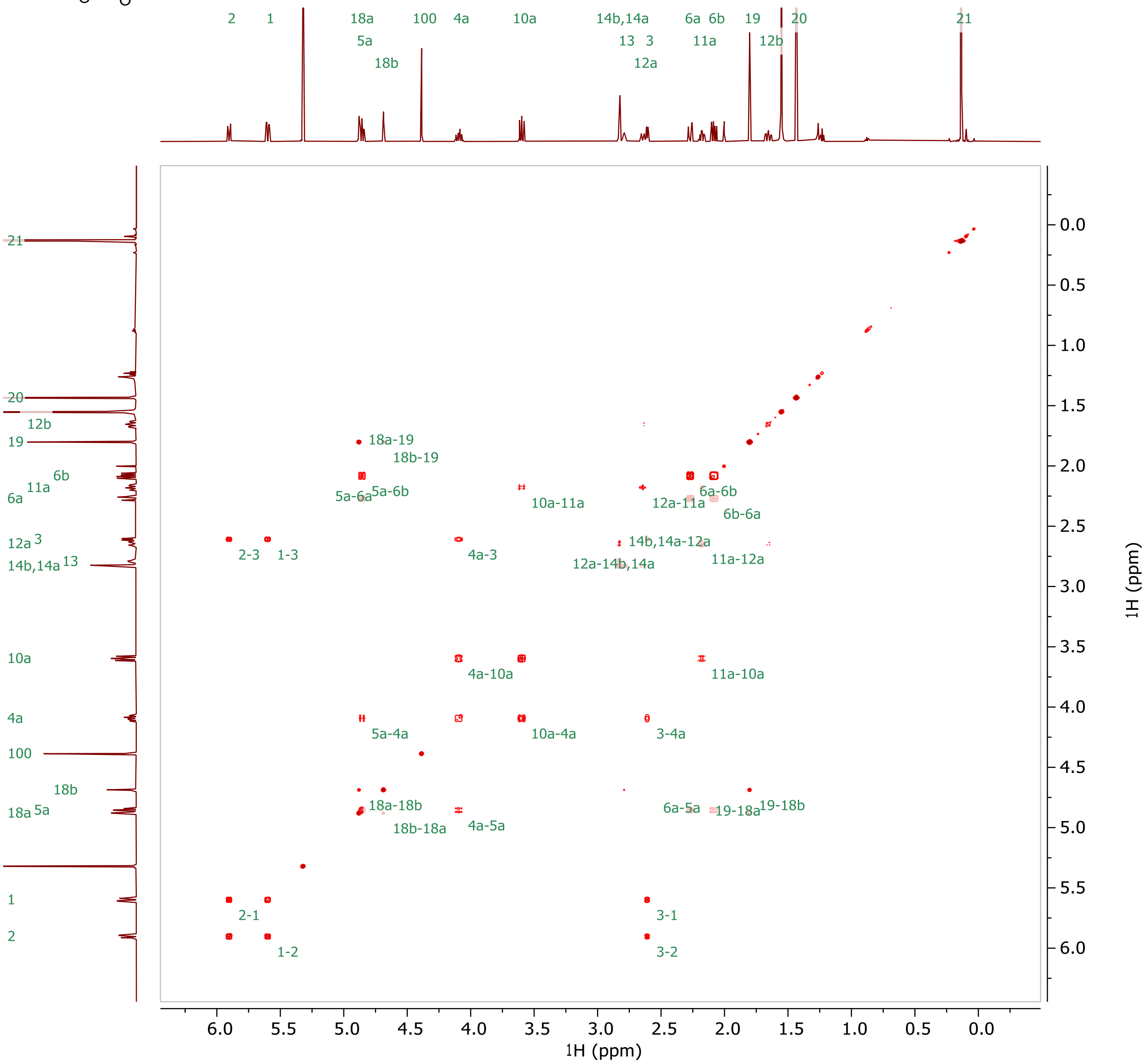

COSY — LID-LA-365-02 (12139) — CD2Cl2; 298.0 K; 7.9 mg; 5 mm — 1H (cosygpppqf) — AV600neo (cryoBBO) — 03.04.24 05:17

HSQC spectrum of 30

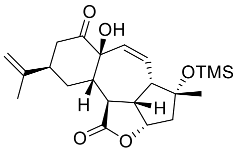

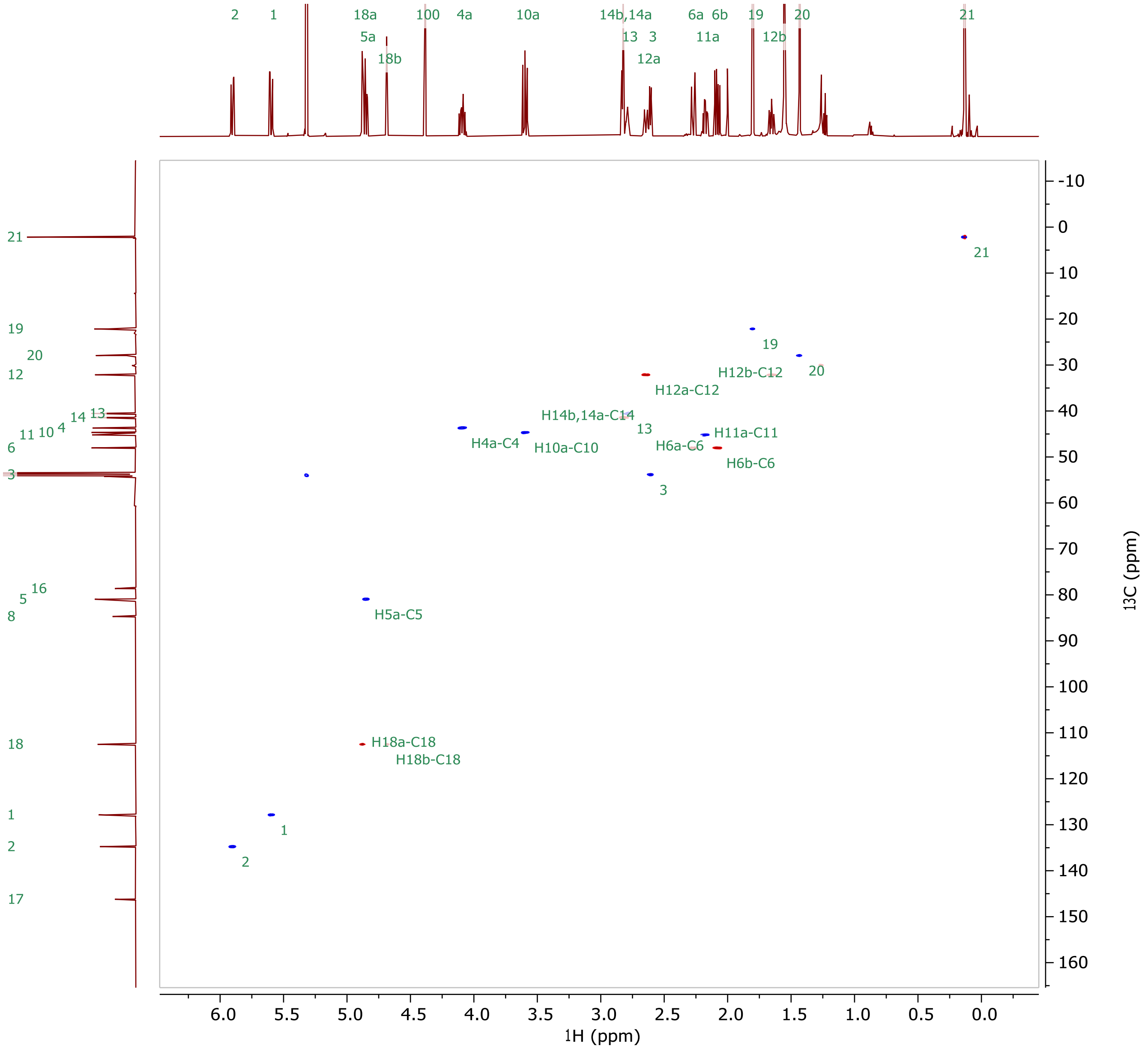

ED — LID-LA-365-02 (12139) — CD2Cl2; 298.0 K; 7.9 mg; 5 mm — 1H-13C (hsqcedetgpsisp2.3) — AV600neo (cryoBBO) — 03.04.24 04:4

HMBC spectrum of 30

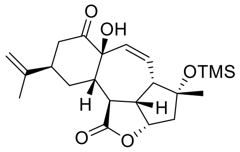

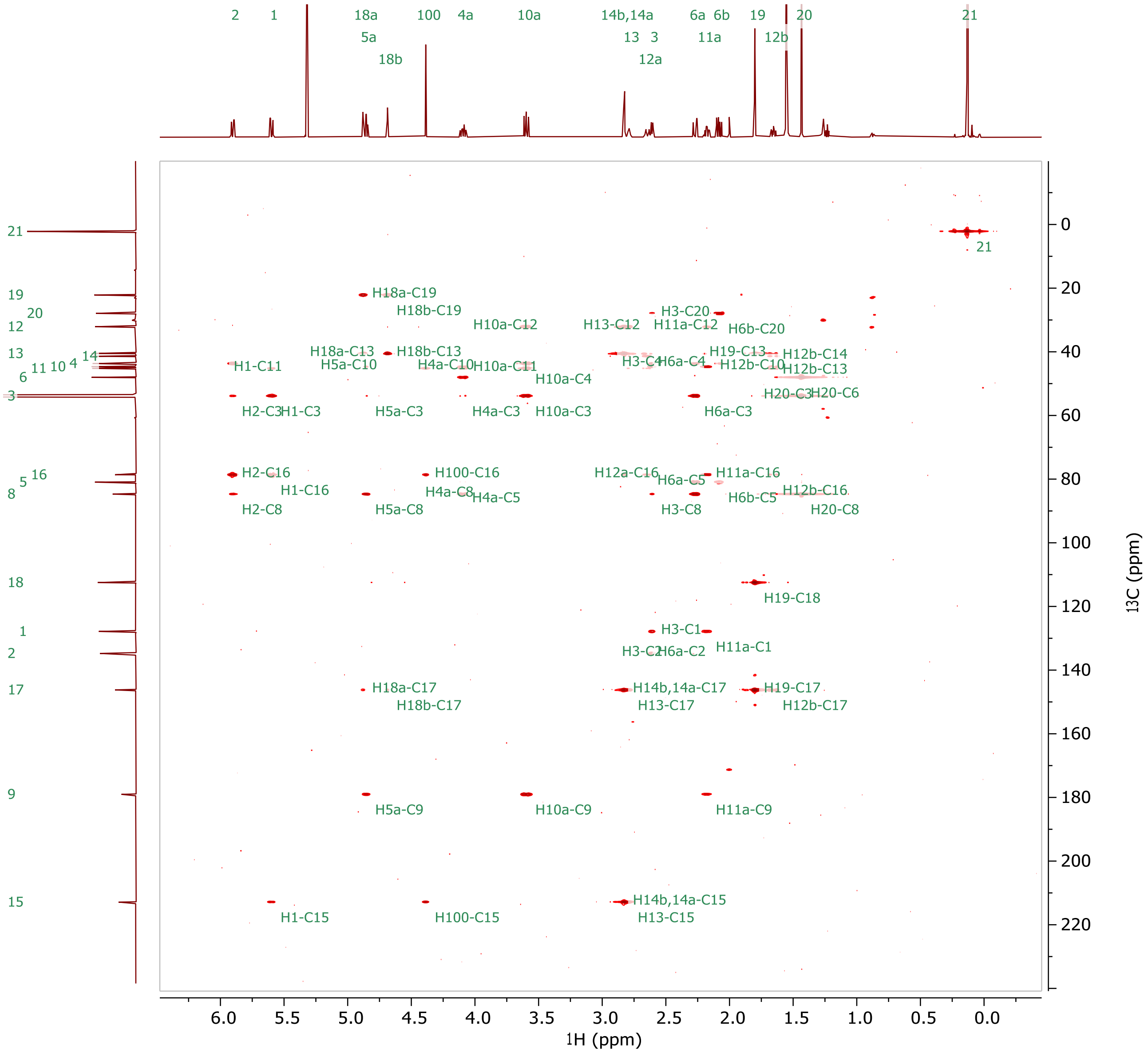

HMBC — LID-LA-365-02 (12139) — CD2Cl2; 298.0 K; 7.9 mg; 5 mm — 1H-13C (hmbcetgpl3nd) — AV600neo (cryoBBO) — 03.04.24 05:0[illegible]

NOESY spectrum of 30

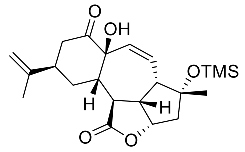

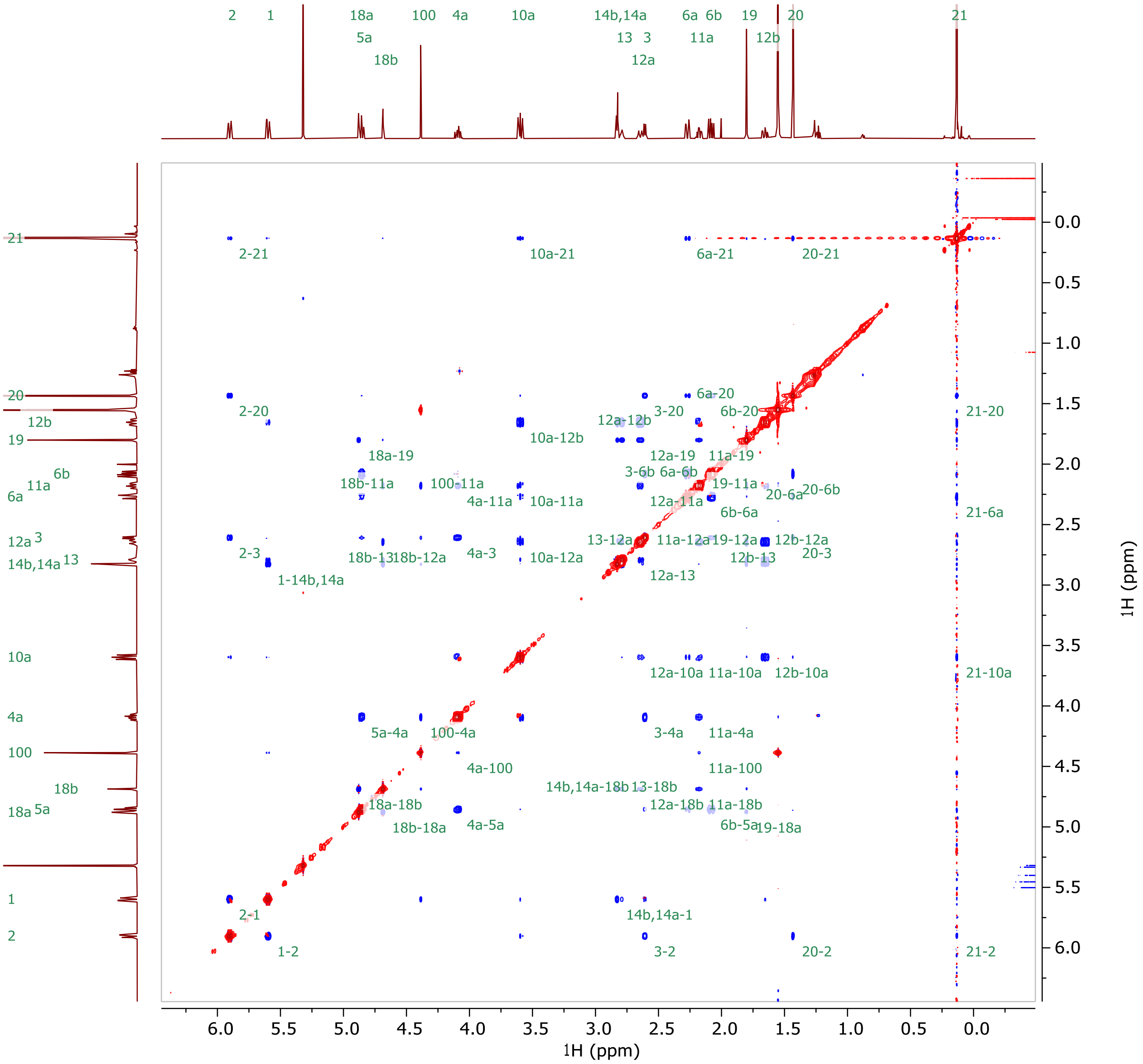

NOESY — LID-LA-365-02 (12139) — CD2Cl2; 298.0 K; 7.9 mg; 5 mm — 1H (noesygpphpp) — AV600neo (cryoBBO) — 03.04.24 06:5:

^{1}H NMR of 31 (600 MHz, CD_2Cl_2)

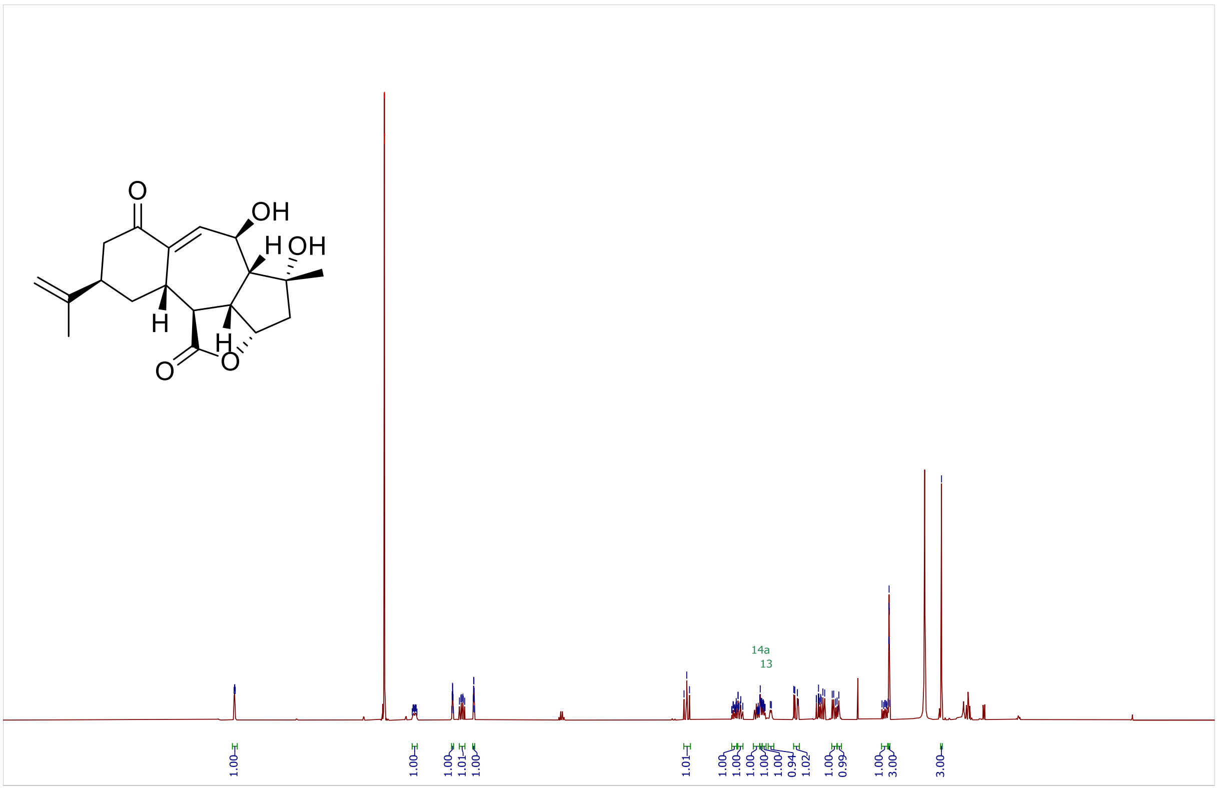

^{13}C NMR of 31 (151 MHz, CD_2Cl_2)

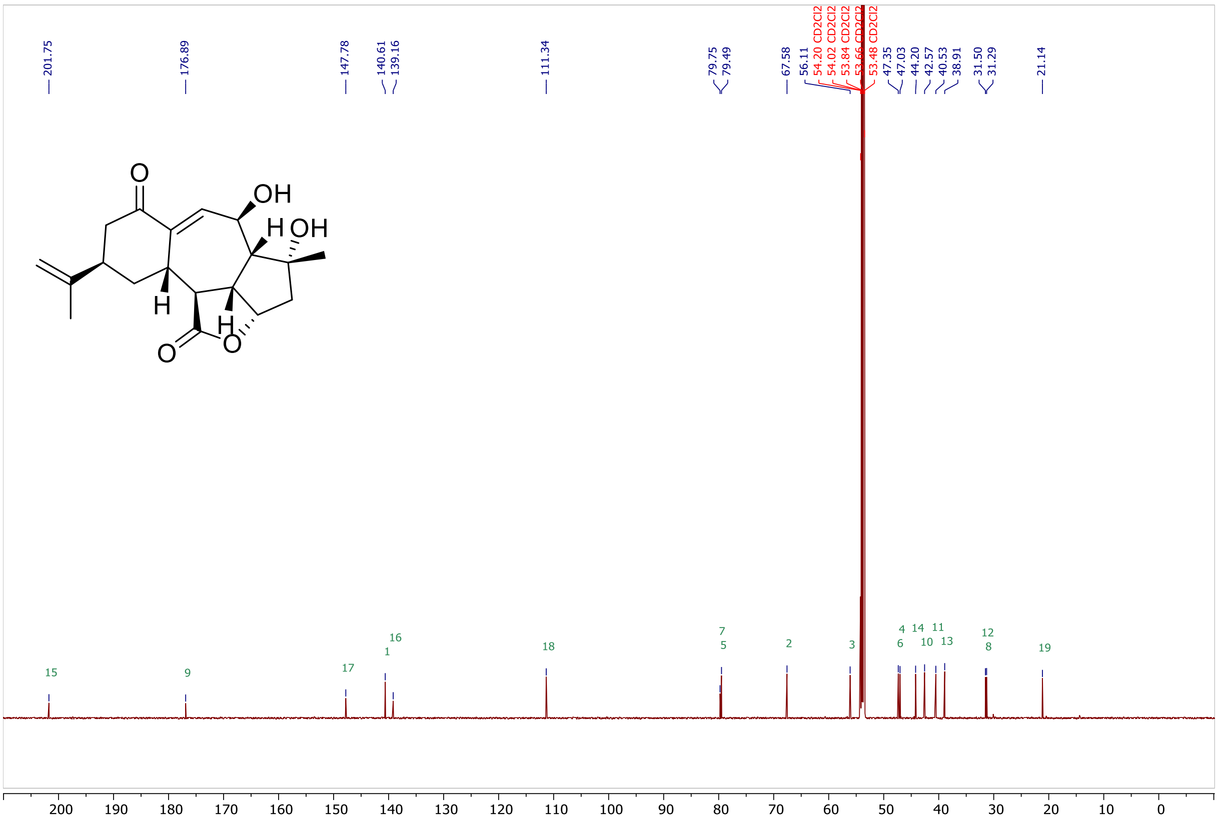

^{1}H COSY spectrum of 31 (600 MHz, CD_2Cl_2)

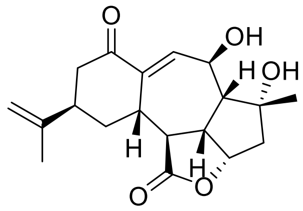

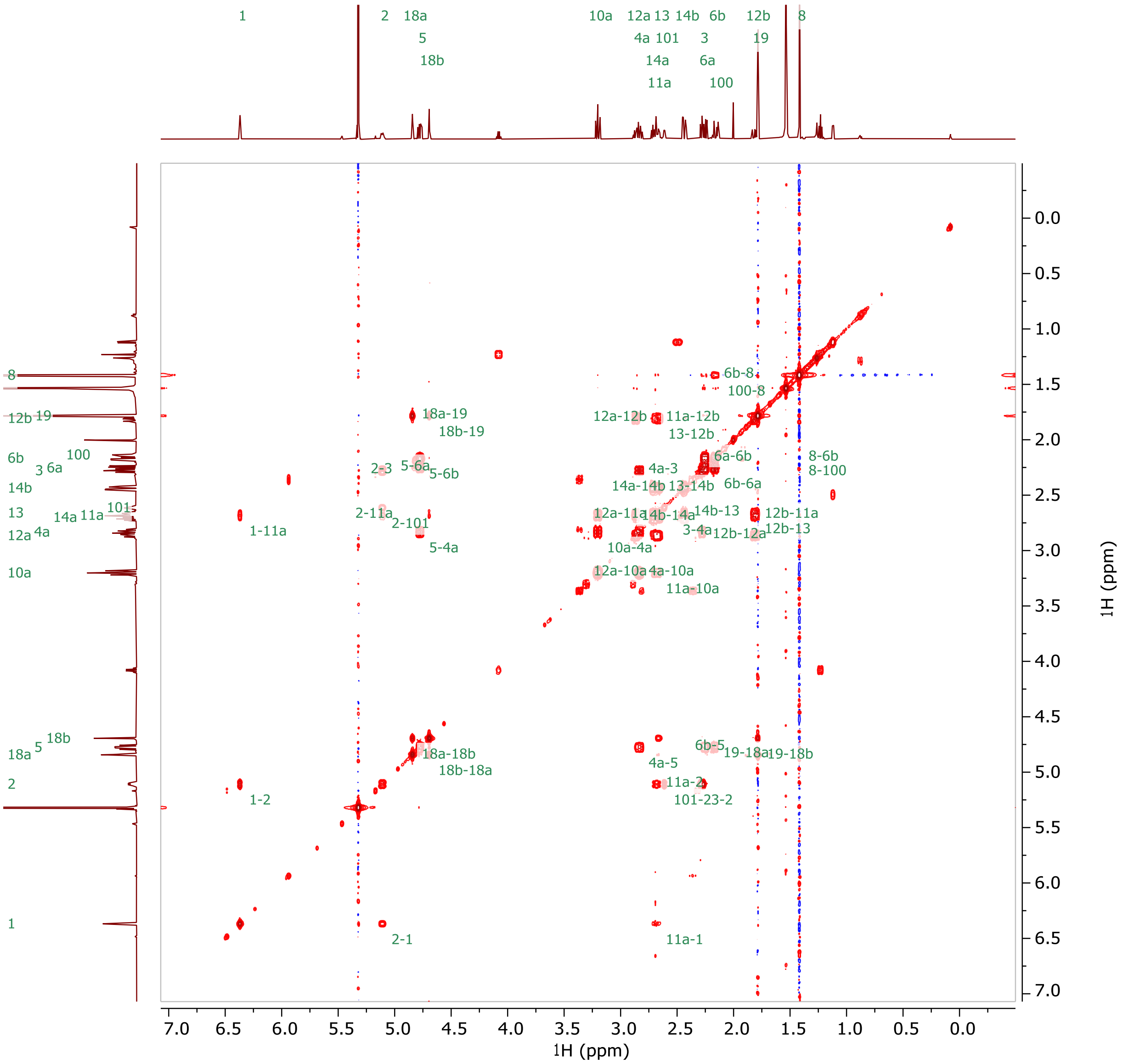

COSY — LID-LA-388-08 (12333) — 5 mm, CD2Cl2, 2.1 mg — 1H (cosygpppqf) @ 298.0 K — AV600neo, cryoBBO — 30.04.24 18:23

HSQC spectrum of 31 (600 MHz, CD_2Cl_2)

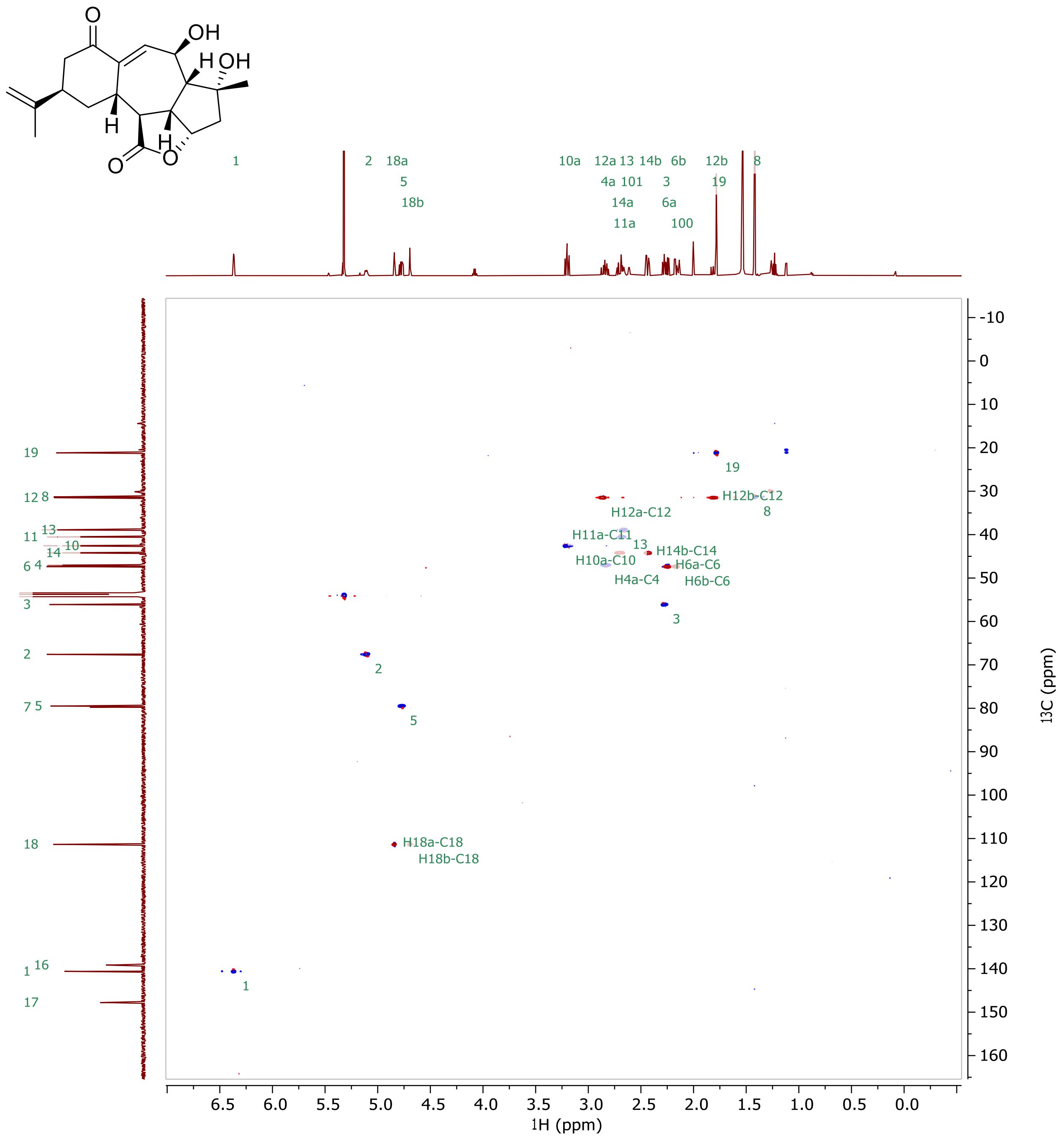

– LID-LA-388-08 (12333) — 5 mm, CD2Cl2, 2.1 mg — 1H-13C (hsqcedetgpsisp2.3) @ 298.0 K — AV600neo, cryoBBO — 30.04.24 17:5

HMBC spectrum of 31 (600 MHz, CD_2Cl_2)

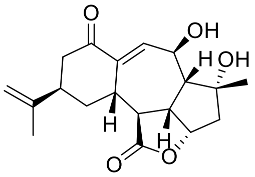

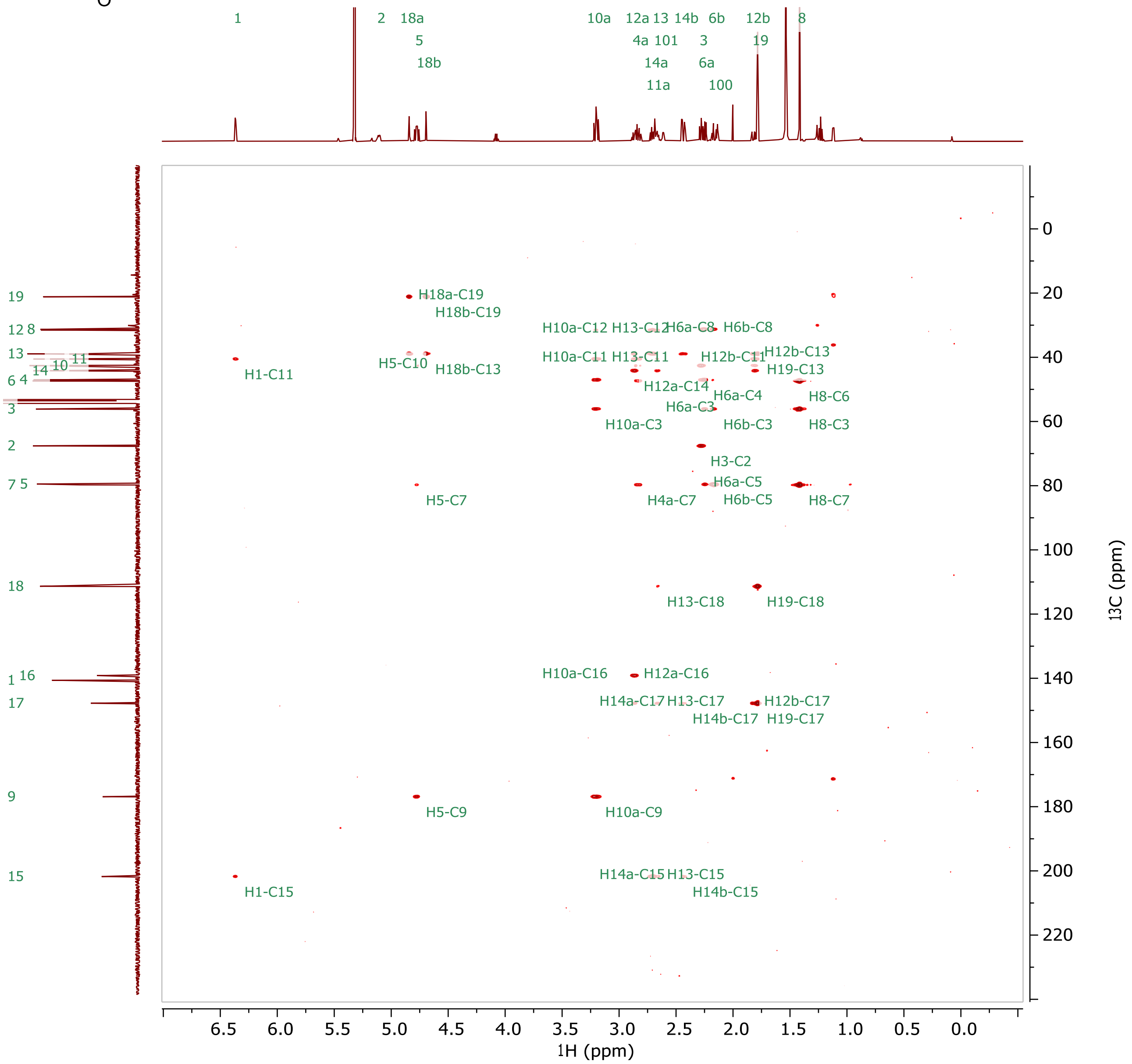

3C — LID-LA-388-08 (12333) — 5 mm, CD2Cl2, 2.1 mg — 1H-13C (hmbcetgpl3nd) @ 298.0 K — AV600neo, cryoBBO — 30.04.24 18:15

NOESY spectrum of 31 (600 MHz, CD_2Cl_2)

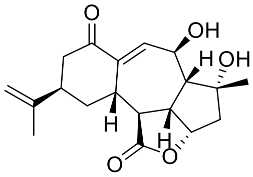

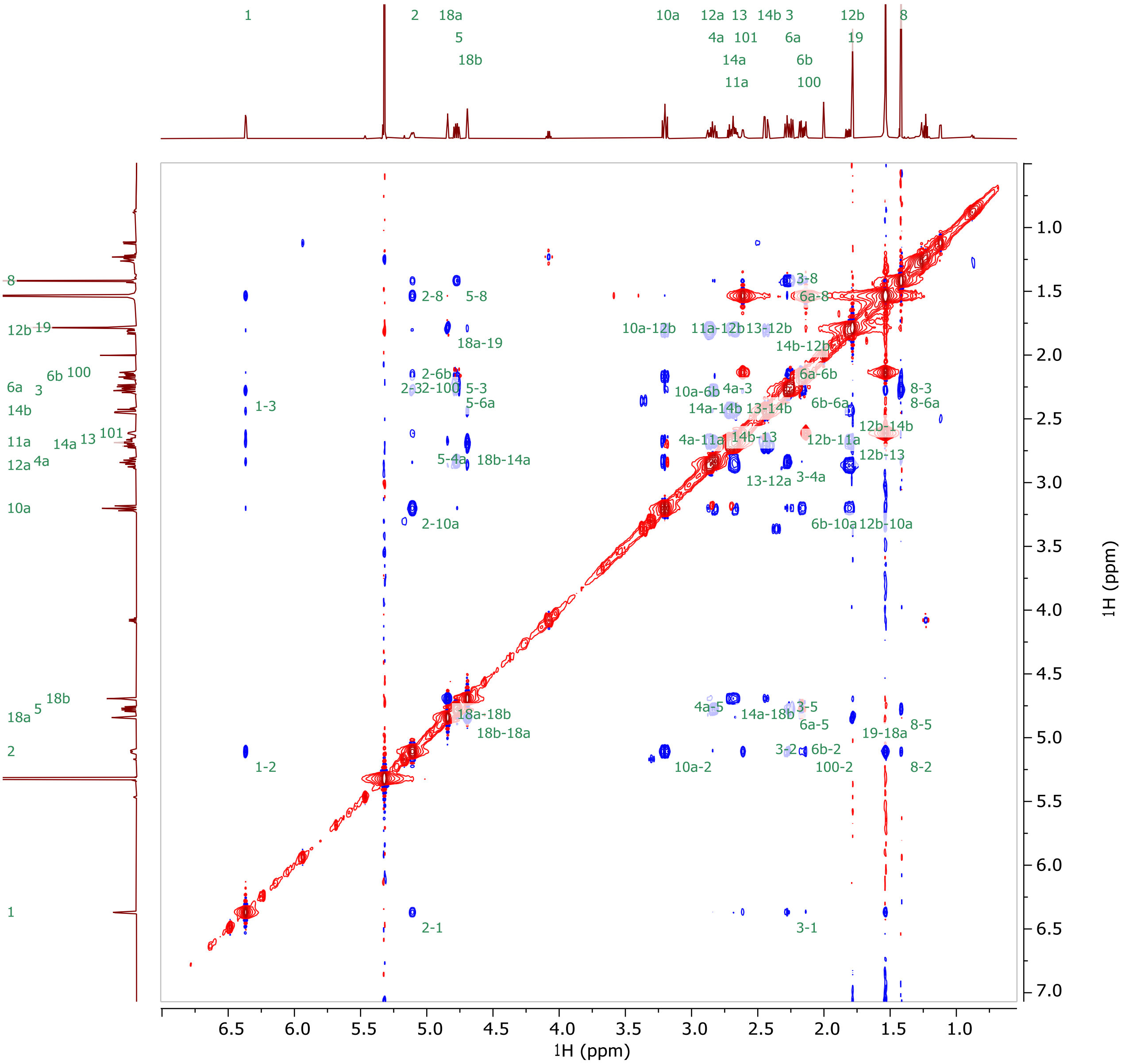

NOESY — LID-LA-388-08 (12333) — 5 mm, CD2Cl2, 2.1 mg — 1H (noesygpphpp) @ 298.0 K — AV600neo, cryoBBO — 30.04.24 19:08

^{1}H NMR of (-)-Scabrolide B (3) (600 MHz, CDCl$_3$)

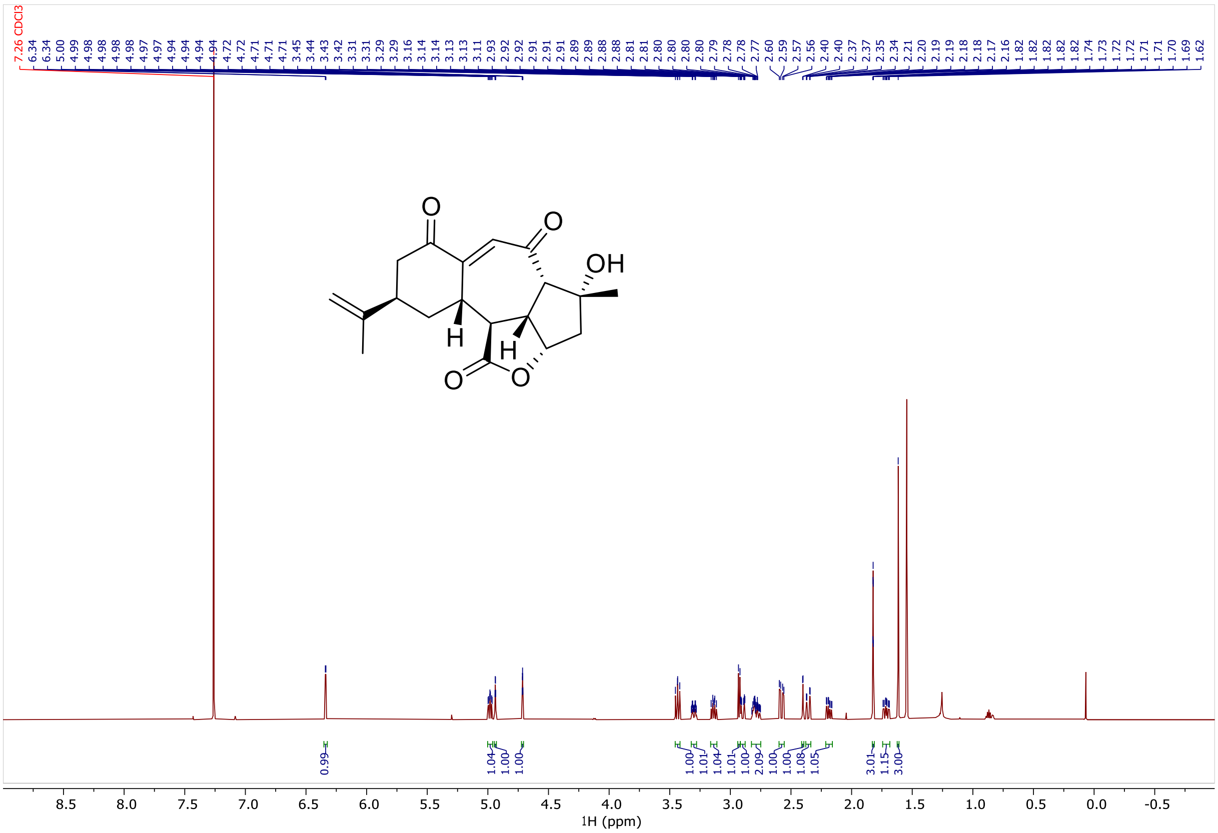

^{13}C NMR of Scabrolide B (3) (151 MHz, CDCl$_3$)

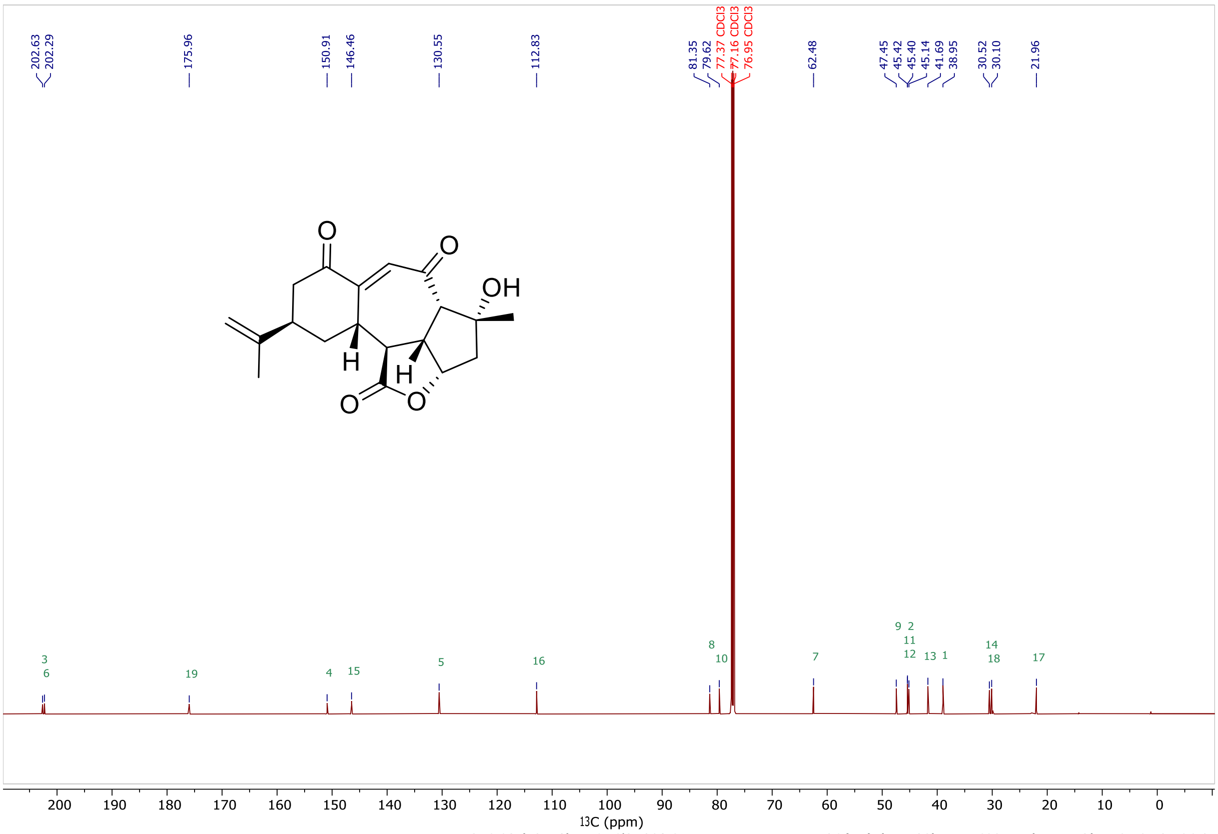

^{1}H COSY spectrum of (-)-Scabrolide B (3) (600 MHz, CDCl3)

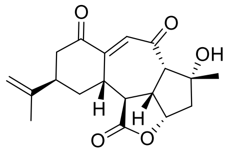

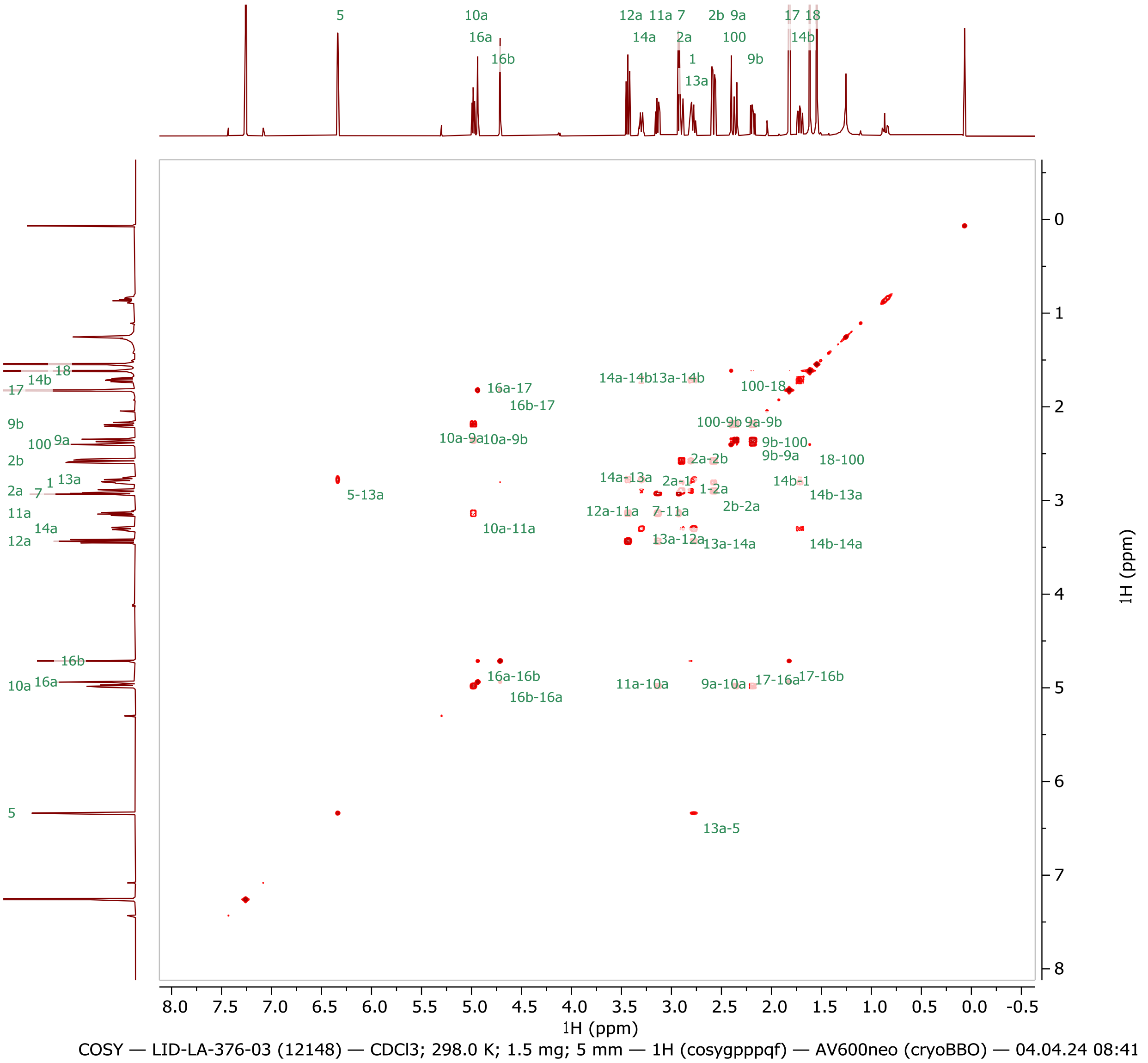

HSQC spectrum of (-)-Scabrolide B (3)

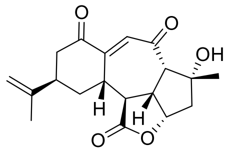

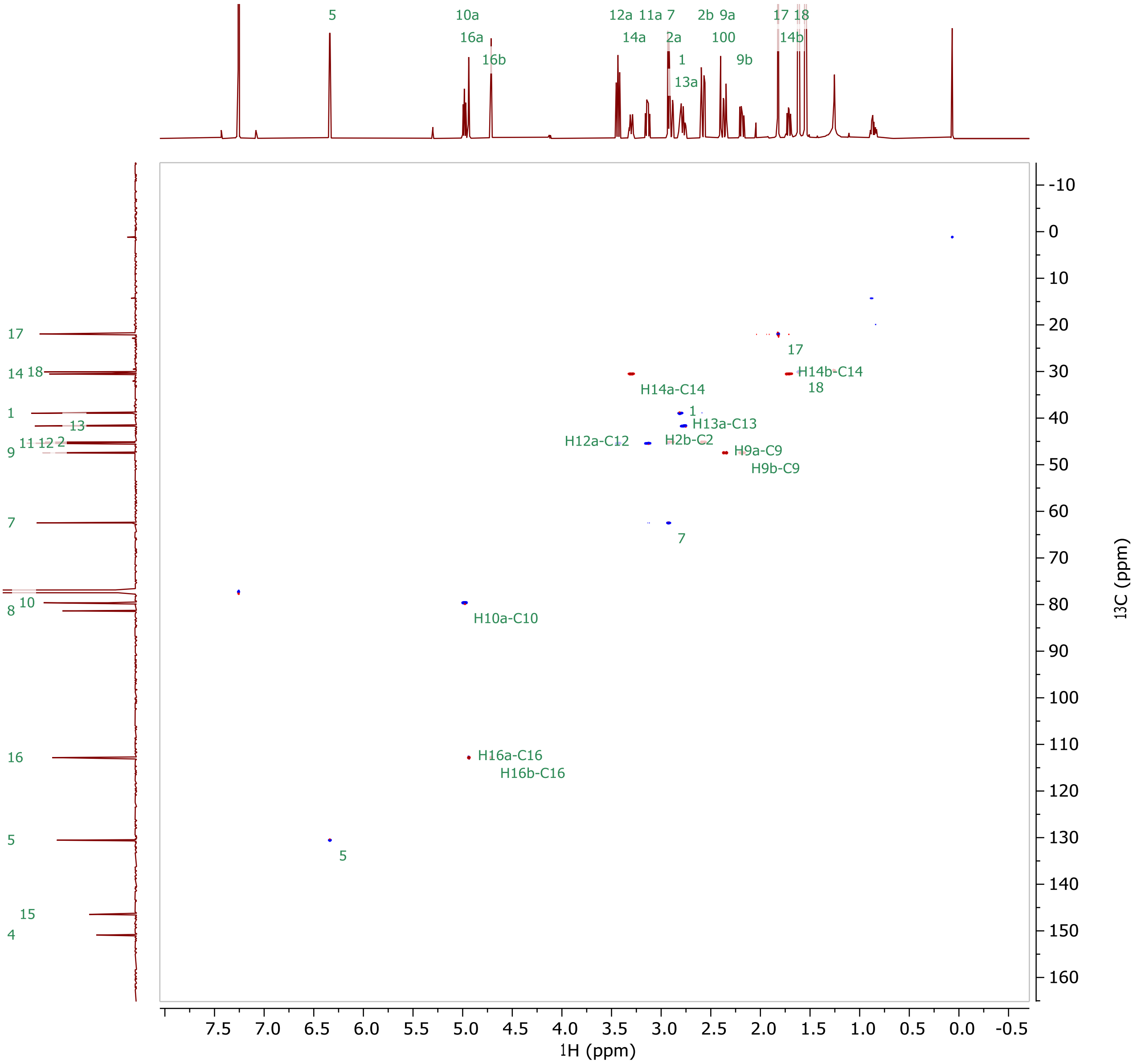

ED — LID-LA-376-03 (12148) — CDCl3; 298.0 K; 1.5 mg; 5 mm — 1H-13C (hsqcedetgpsisp2.3) — AV600neo (cryoBBO) — 04.04.24 06:4

HMBC spectrum of (-)-Scabrolide B (3)

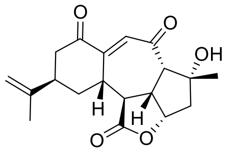

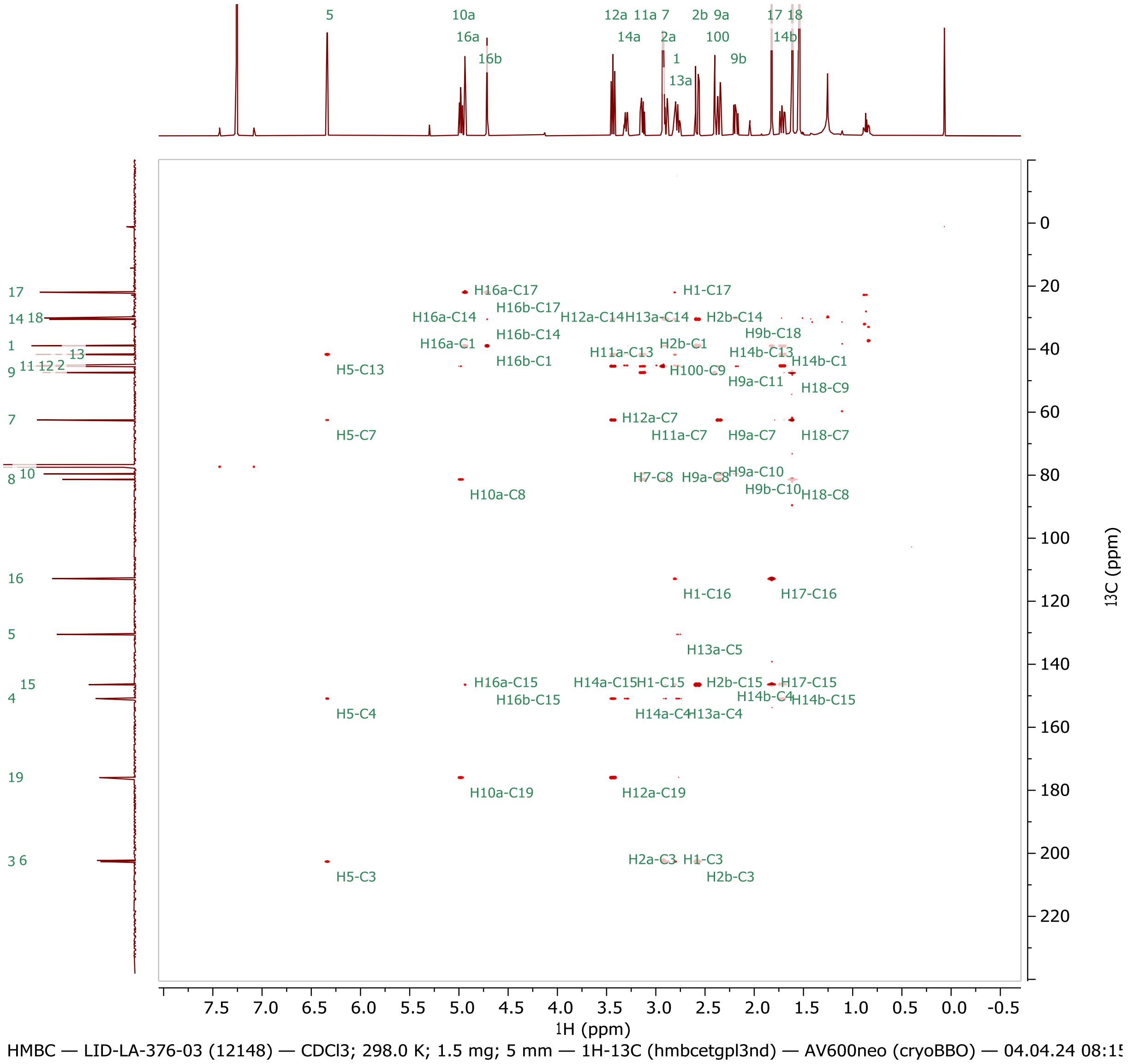

NOESY spectrum of (-)-Scabrolide B (3)

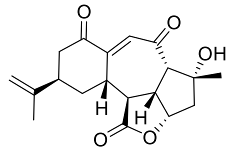

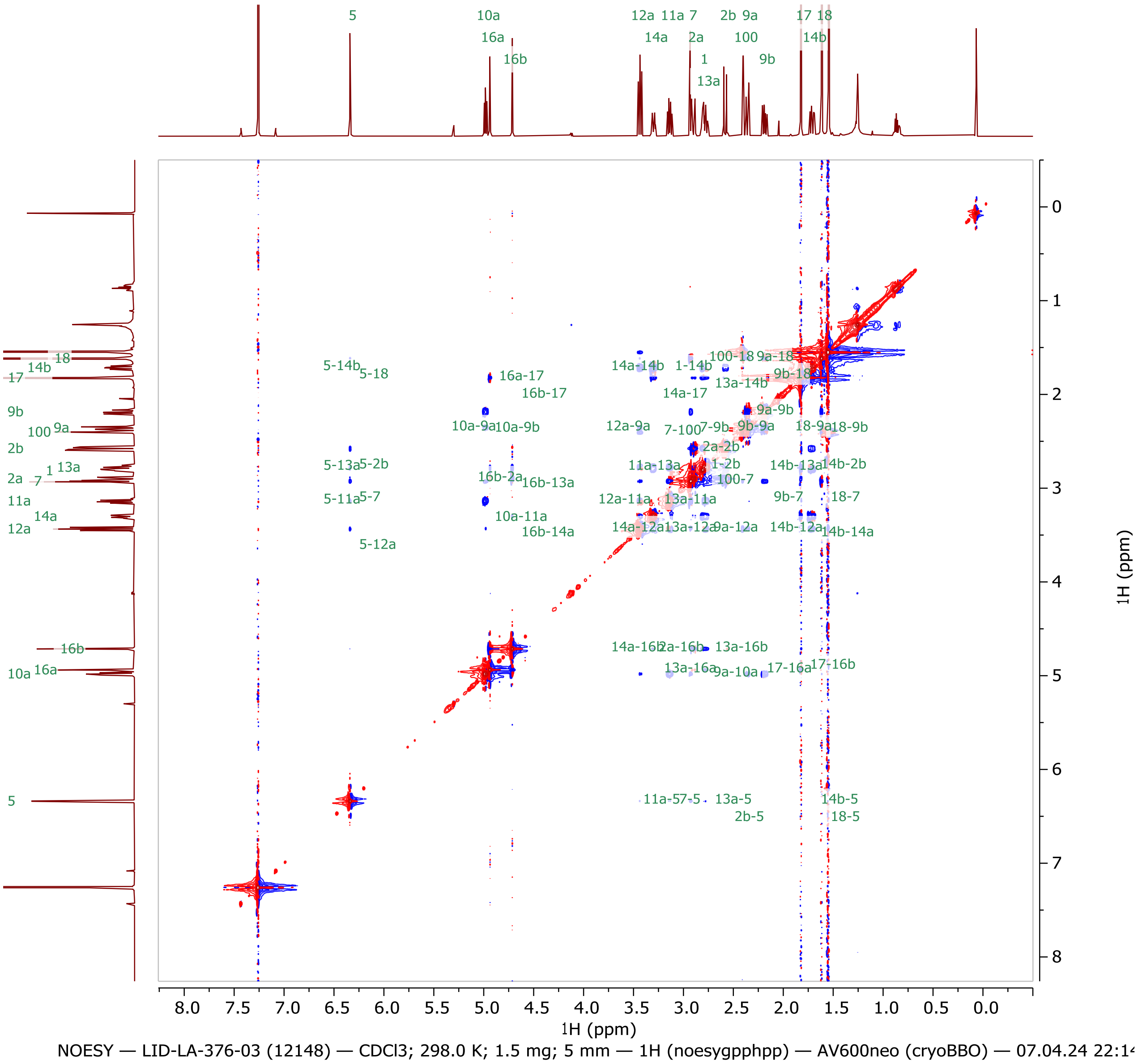

^{1}H NMR of Sinuscalide C (4) (600 MHz, $CDCl_3$)

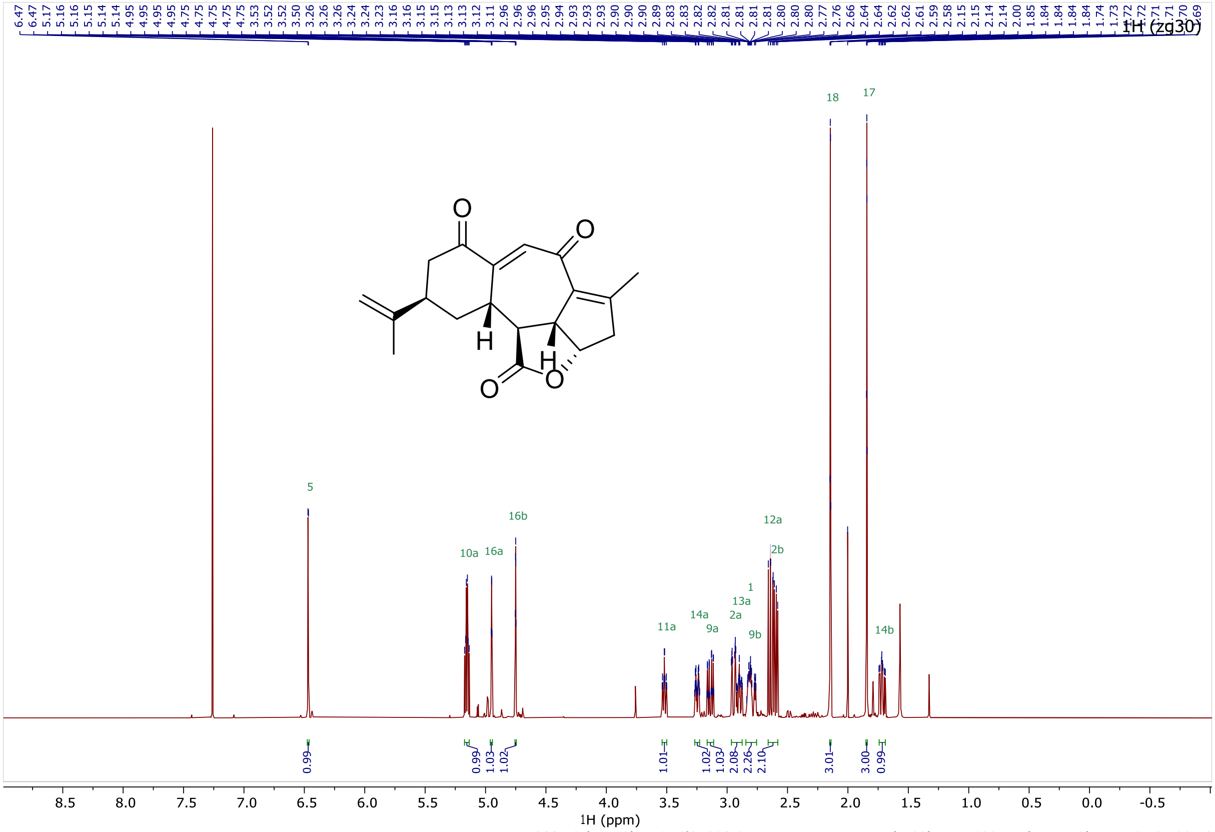

1D — LID-LA-388-10 (12412) — CDCl3; 298.0 K; 7 mg; 5 mm — 1H (zg30) — AV600neo (cryoBBO) — 14.05.24 23:18

^{13}C NMR of Sinuscalide C (4) (151 MHz, $CDCl_3$)

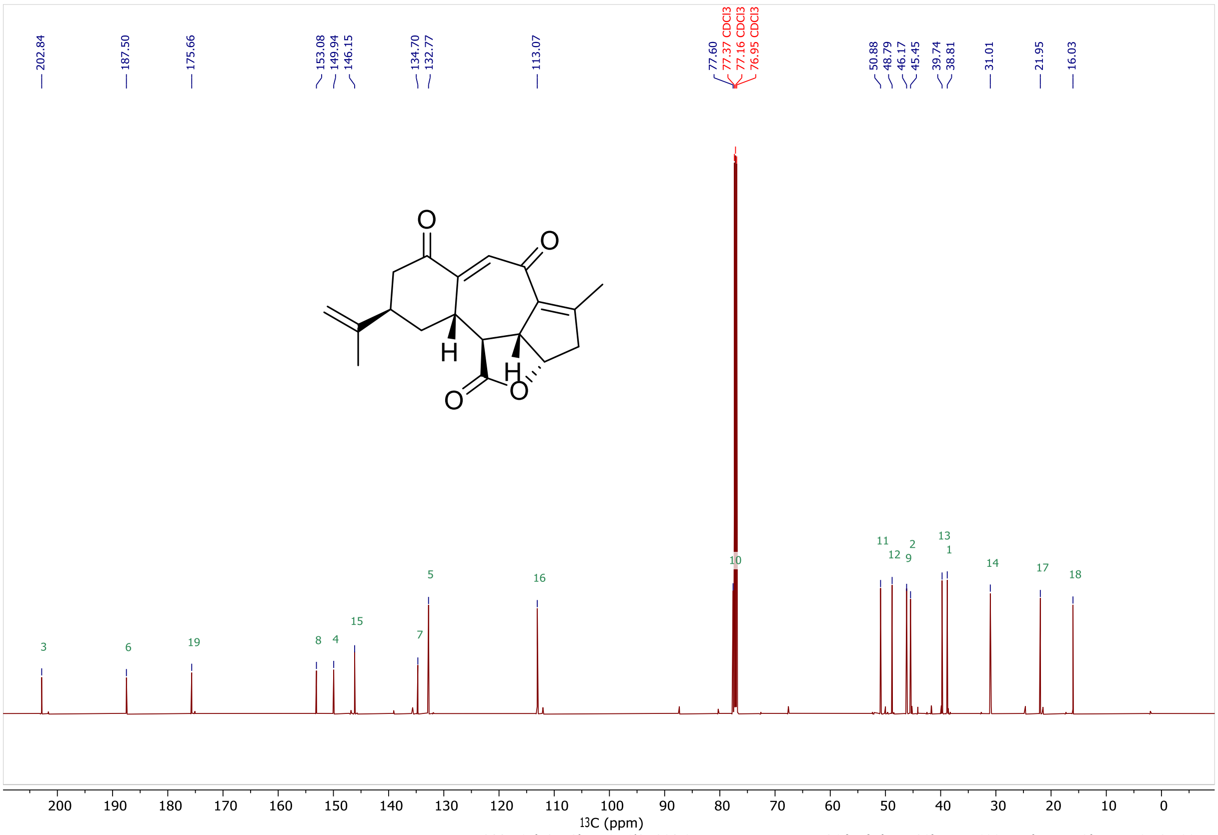

1D — LID-LA-388-10 (12412) — CDCl3; 298.0 K; 7 mg; 5 mm — 13C{1H} (zgpg30) — AV600neo (cryoBBO) — 15.05.24 00:11

^{1}H COSY spectrum of Sinuscalide C (4) (600 MHz, CDCl$_3$)

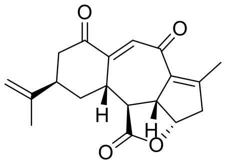

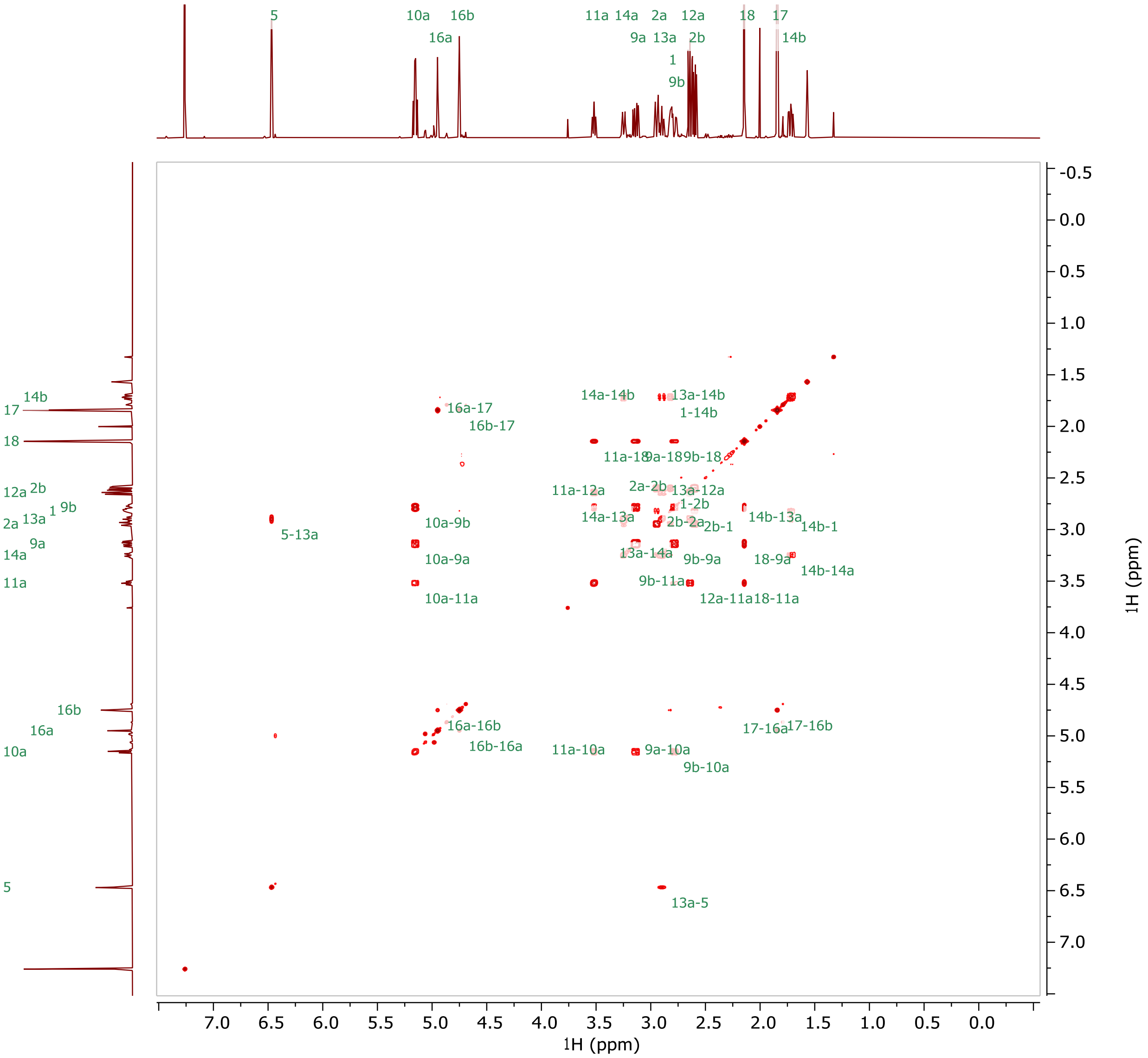

COSY — LID-LA-388-10 (12412) — CDCl3; 298.0 K; 7 mg; 5 mm — 1H (cosygpppqf) — AV600neo (cryoBBO) — 15.05.24 01:35

HSQC spectrum of Sinuscalide C (4)

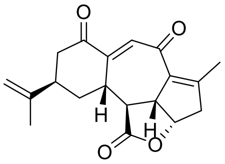

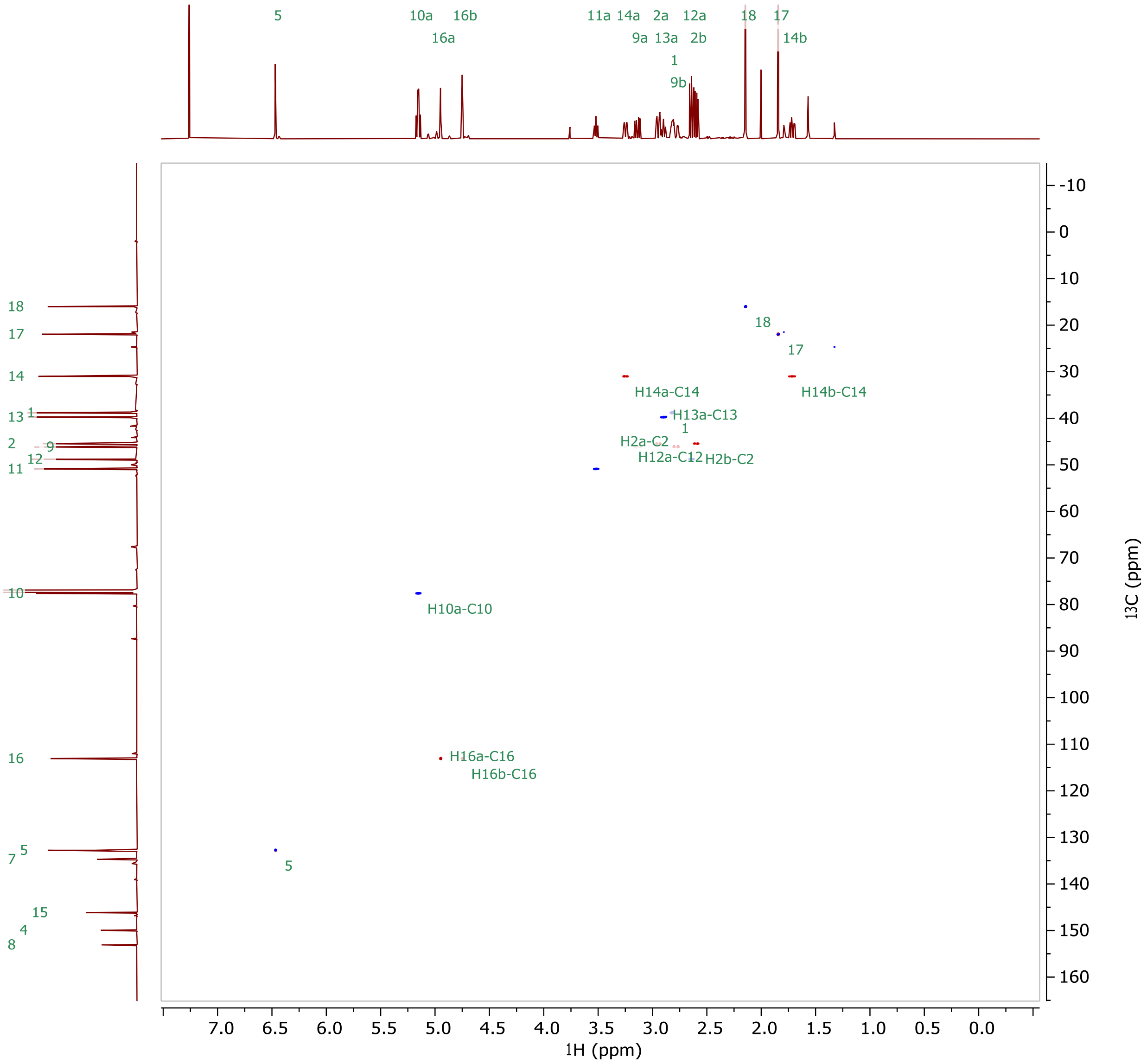

ITED — LID-LA-388-10 (12412) — CDCl3; 298.0 K; 7 mg; 5 mm — 1H-13C (hsqcedetgpsisp2.3) — AV600neo (cryoBBO) — 15.05.24 00:2

HMBC spectrum of Sinuscalide C (4)

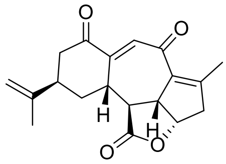

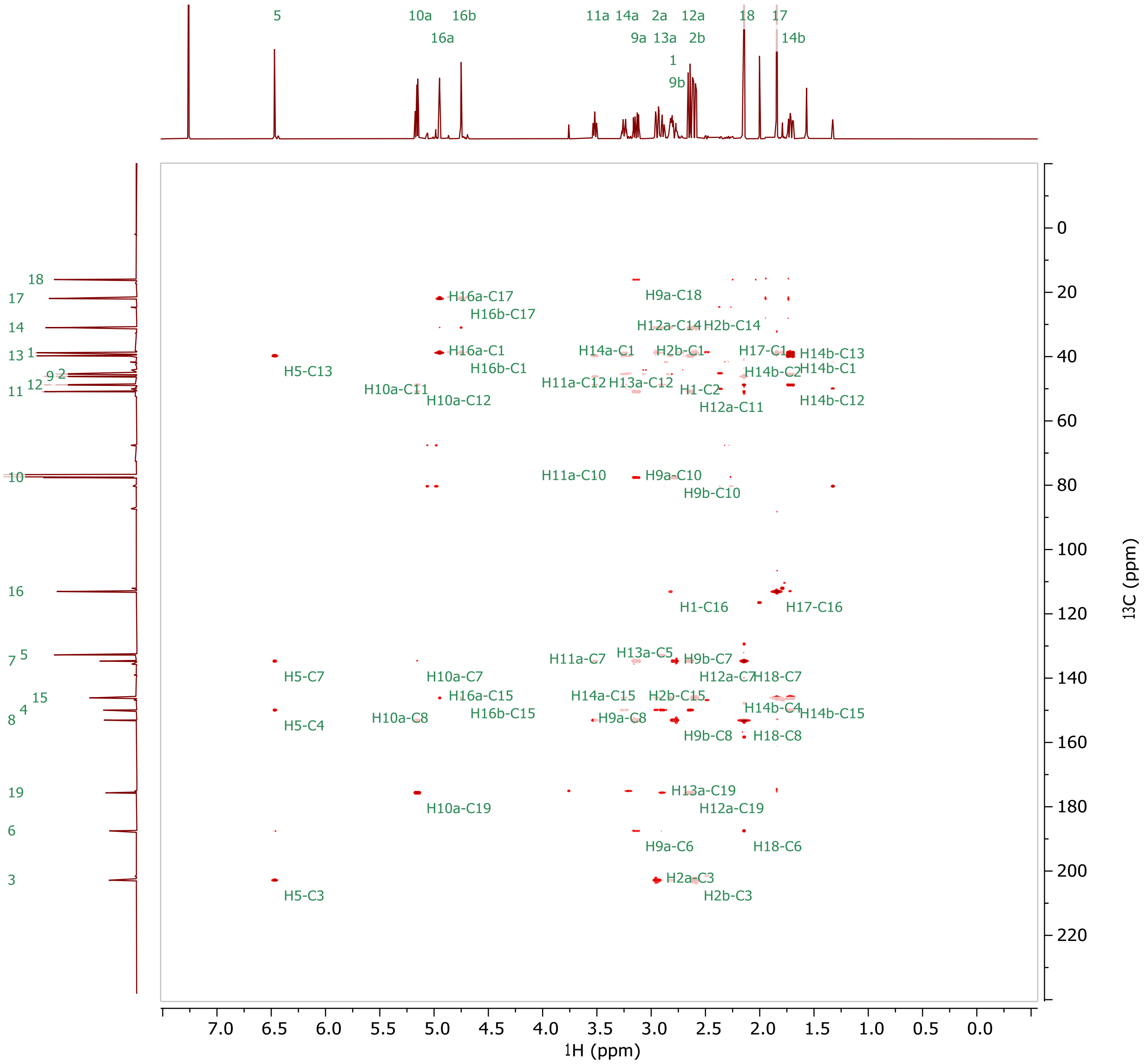

HMBC — LID-LA-388-10 (12412) — CDCl3; 298.0 K; 7 mg; 5 mm — 1H-13C (hmbcetgpl3nd) — AV600neo (cryoBBO) — 15.05.24 01:1(

NOESY spectrum of Sinuscalide C (4)

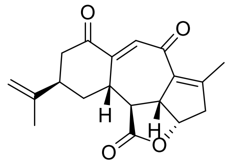

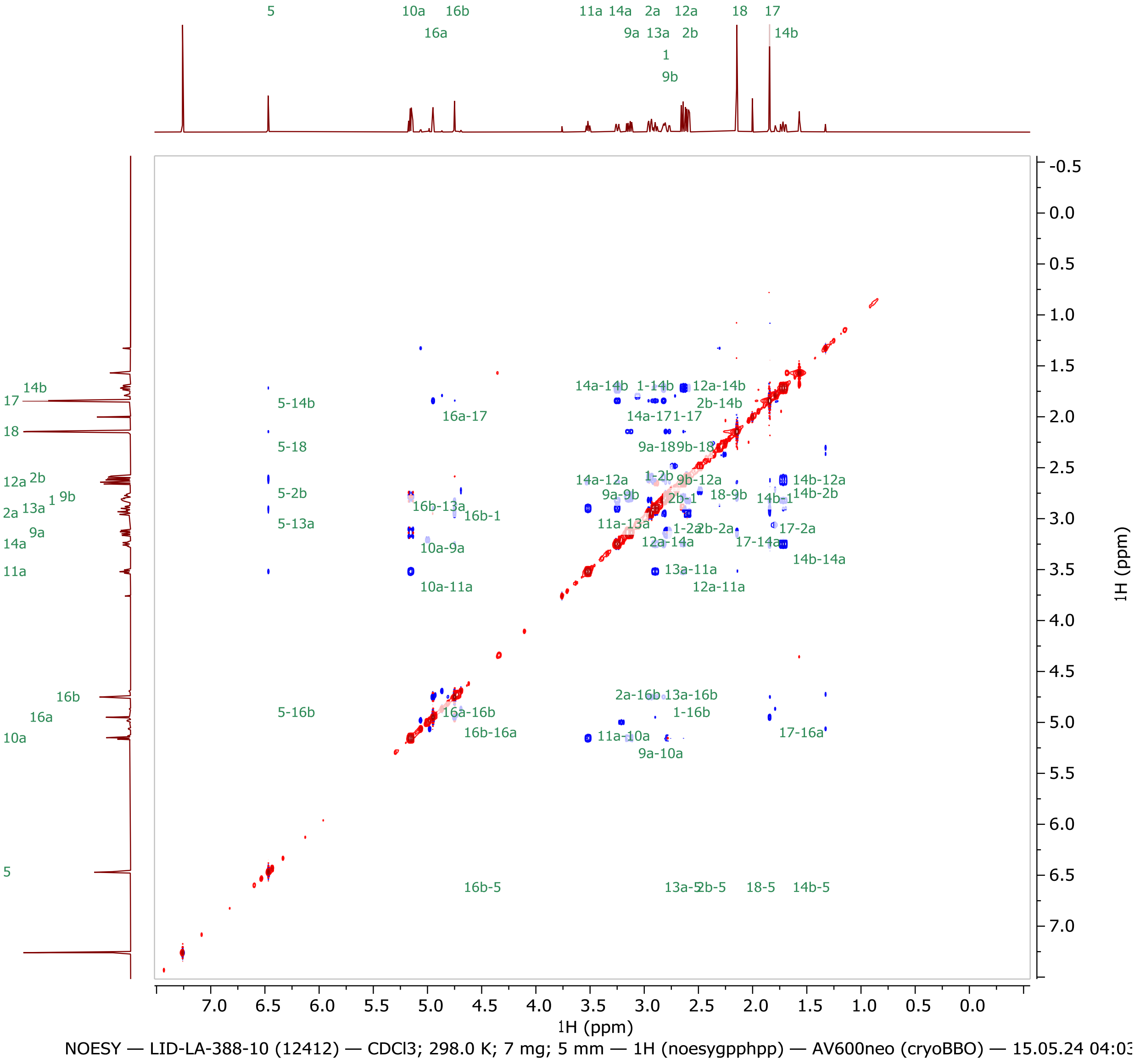

^{1}H NMR of Ineleganolide (6) (600 MHz, $CDCl_3$)

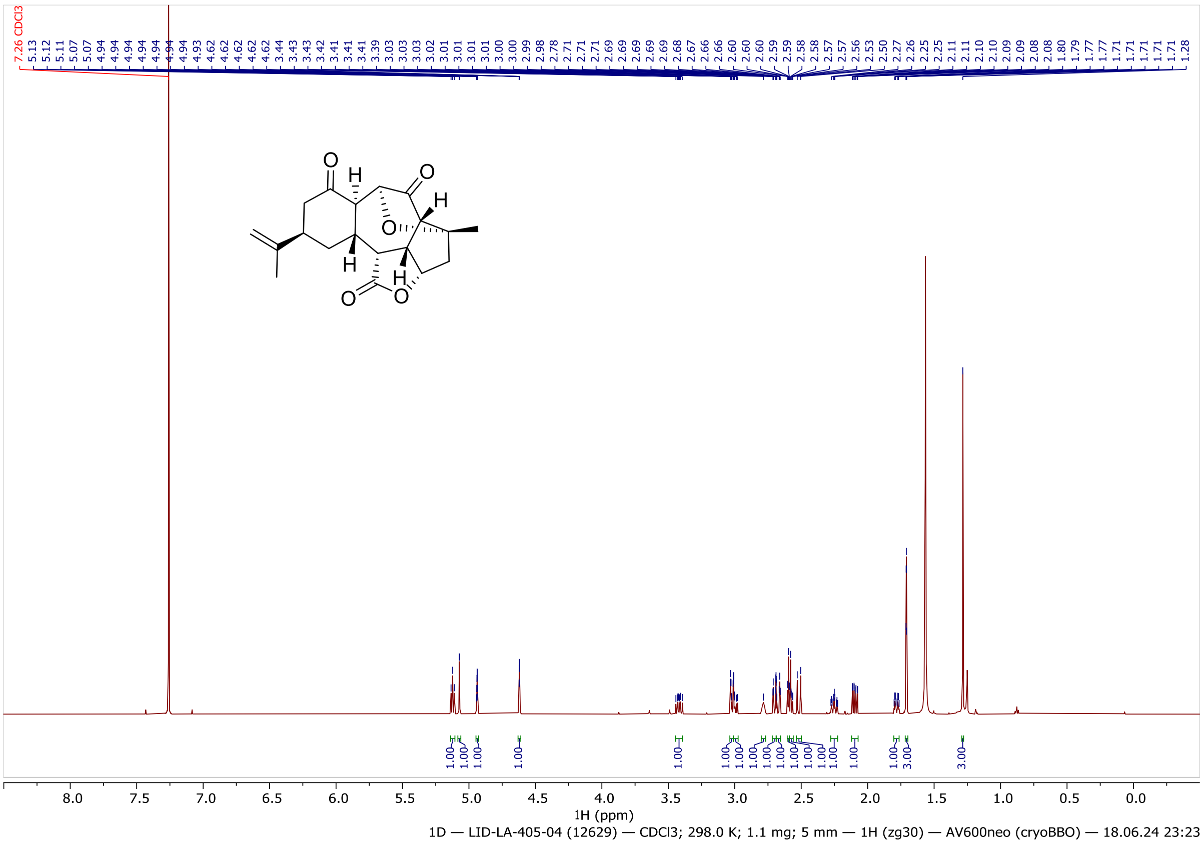

^{13}C NMR of Ineleganolide (6) (151 MHz, $CDCl_3$)

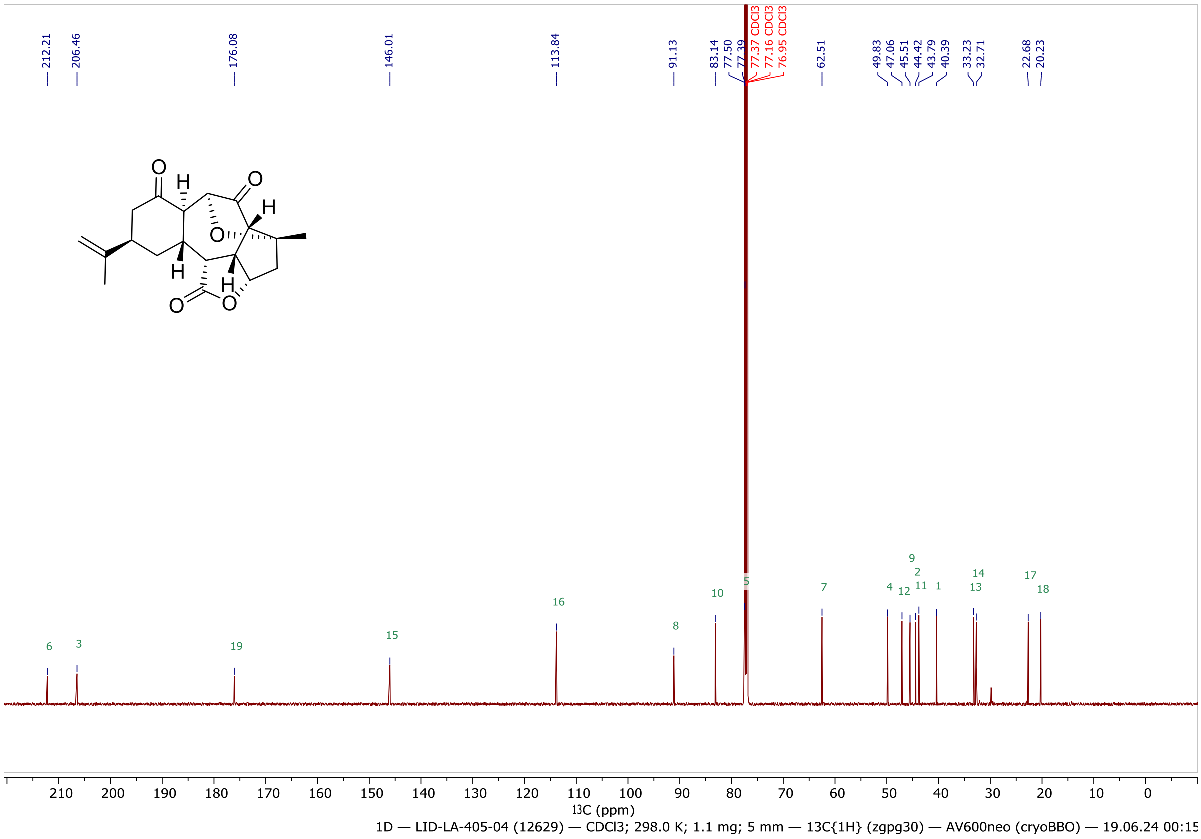

^{1}H COSY spectrum of Ineleganolide (6) (600 MHz, $CDCl_3$)

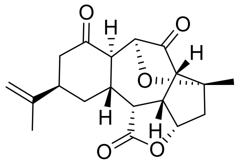

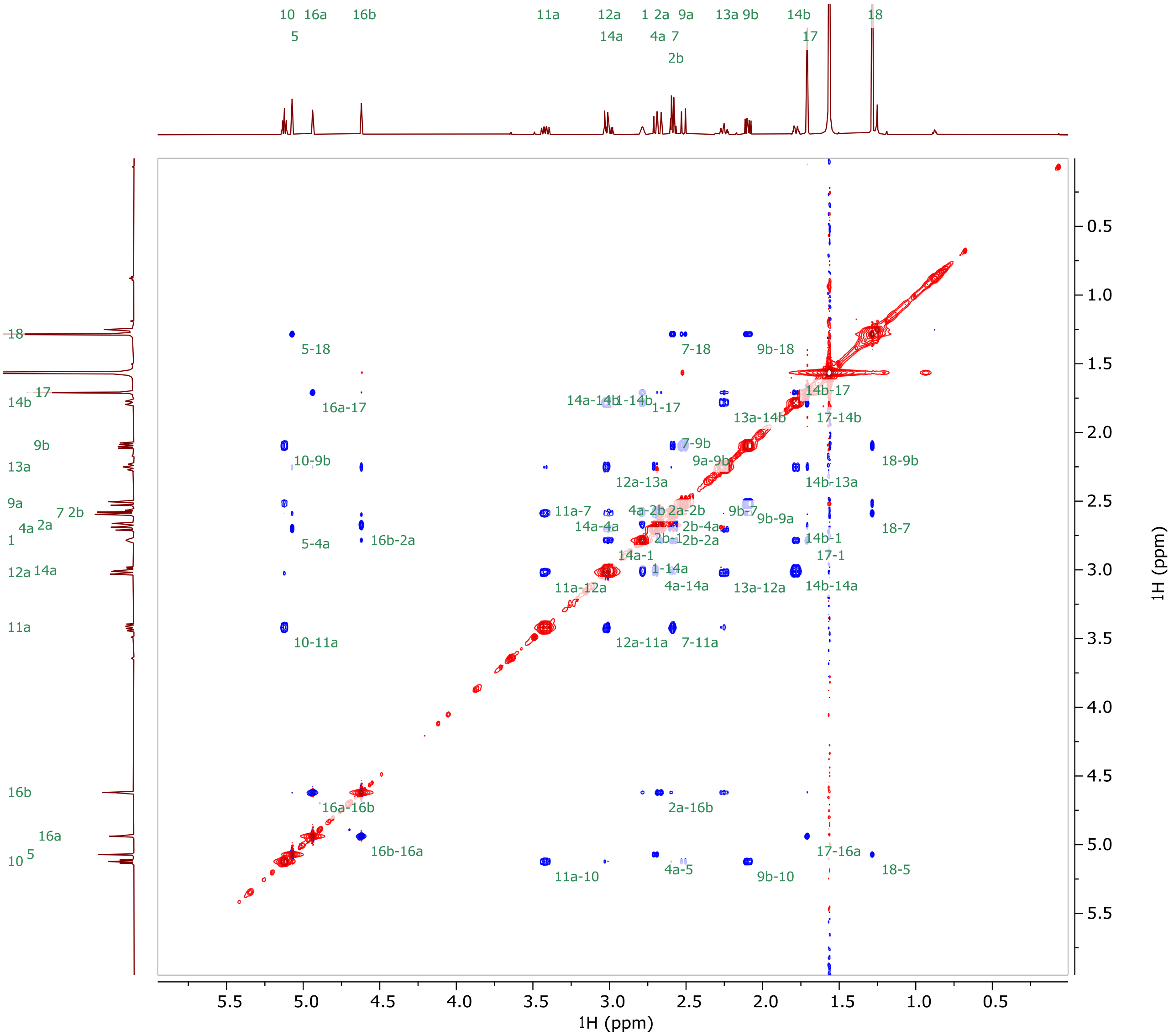

HSQC spectrum of Ineleganolide (6)

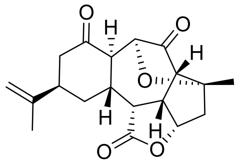

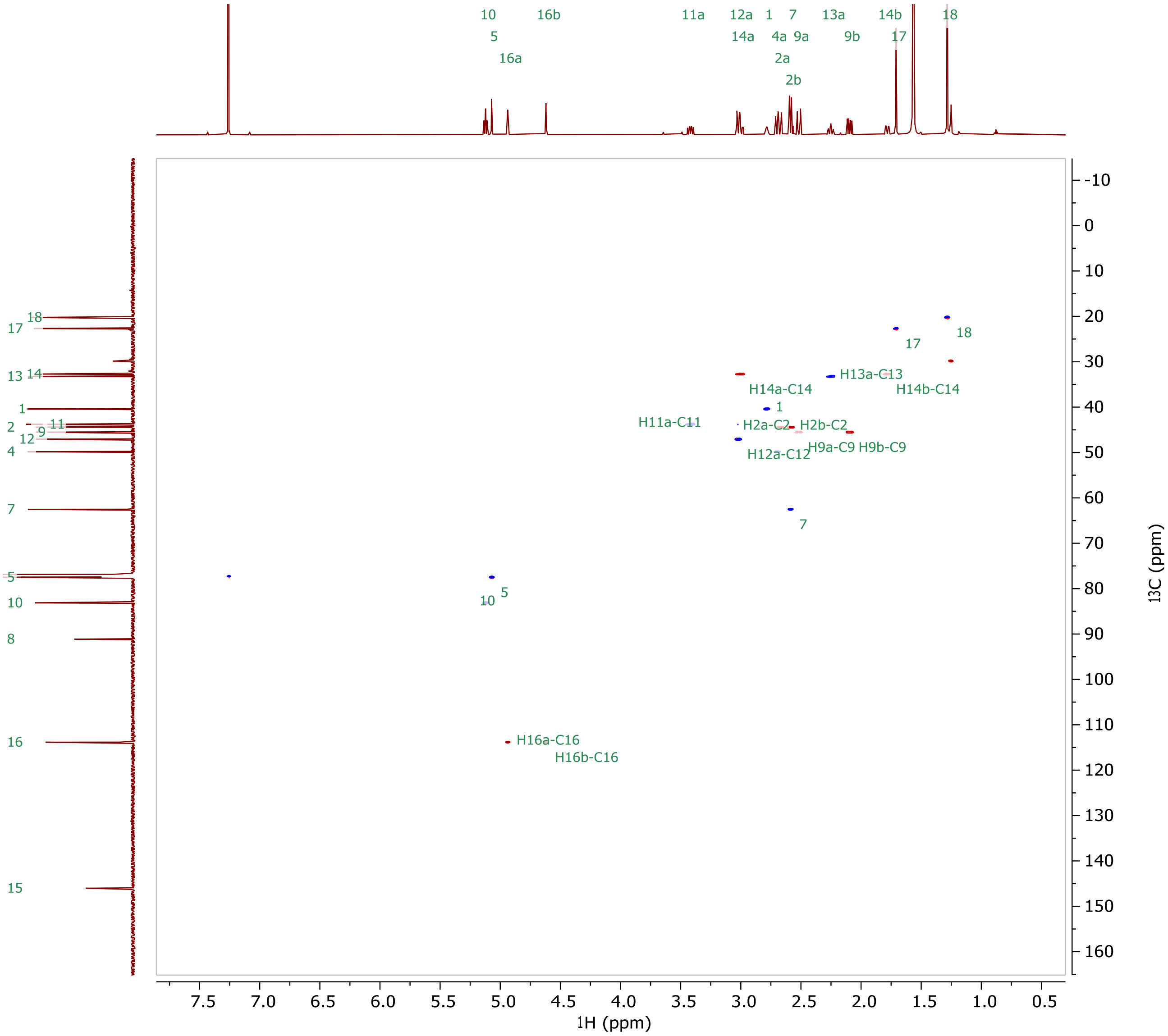

C-EDITED — LID-LA-405-04 (12629) — CDCl3; 298.0 K; 1.1 mg; 5 mm — 1H-13C (hsqcedetgpsisp2.3) — AV600neo (cryoBBO) — 19.06.24 00:3

HMBC spectrum of Ineleganolide (6)

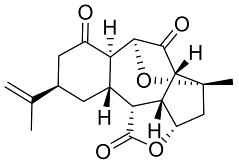

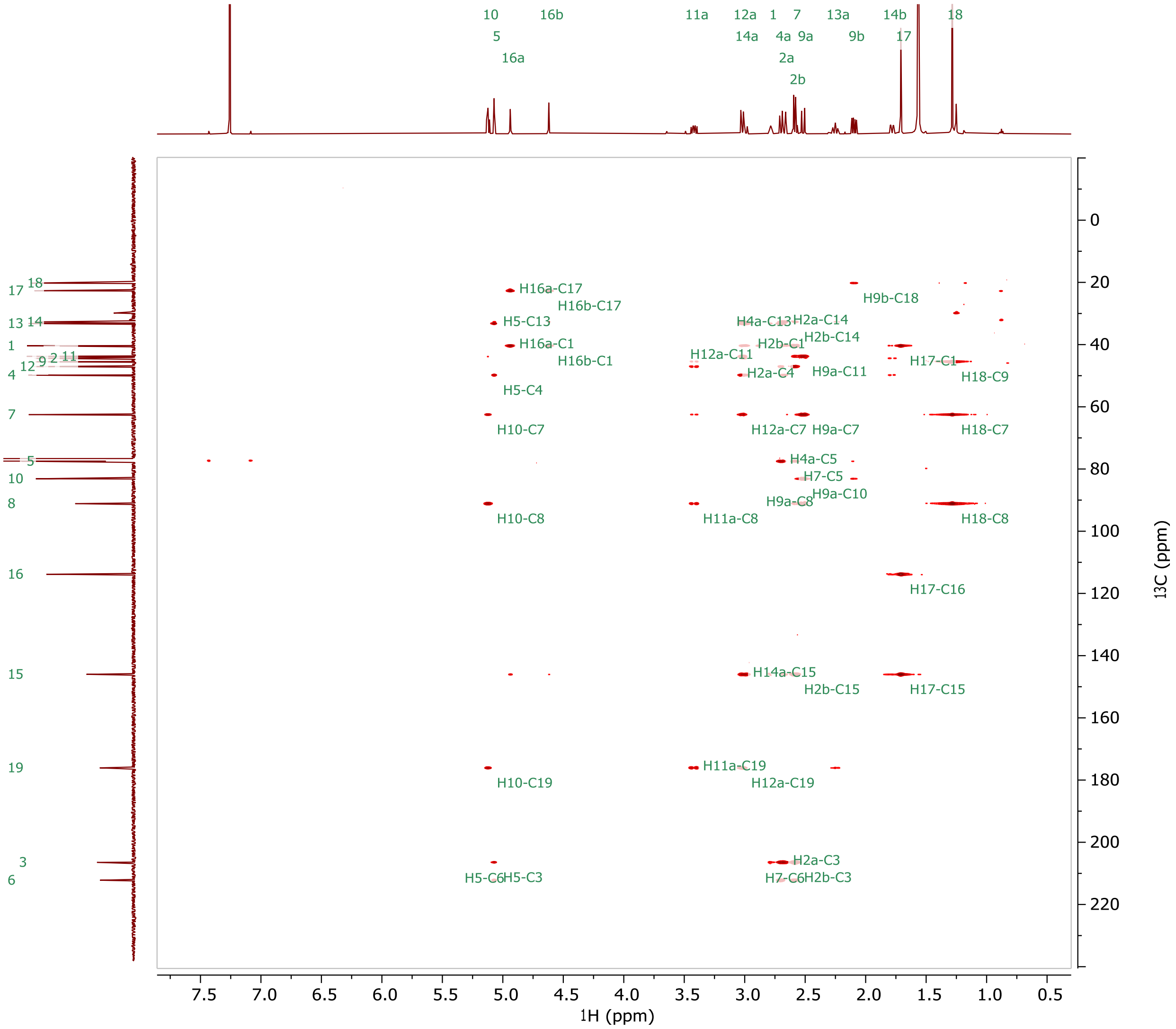

HMBC — LID-LA-405-04 (12629) — CDCl3; 298.0 K; 1.1 mg; 5 mm — 1H-13C (hmbcetgpl3nd) — AV600neo (cryoBBO) — 19.06.24 02:27

NOESY spectrum of Ineleganolide (6)

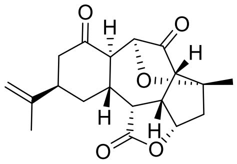

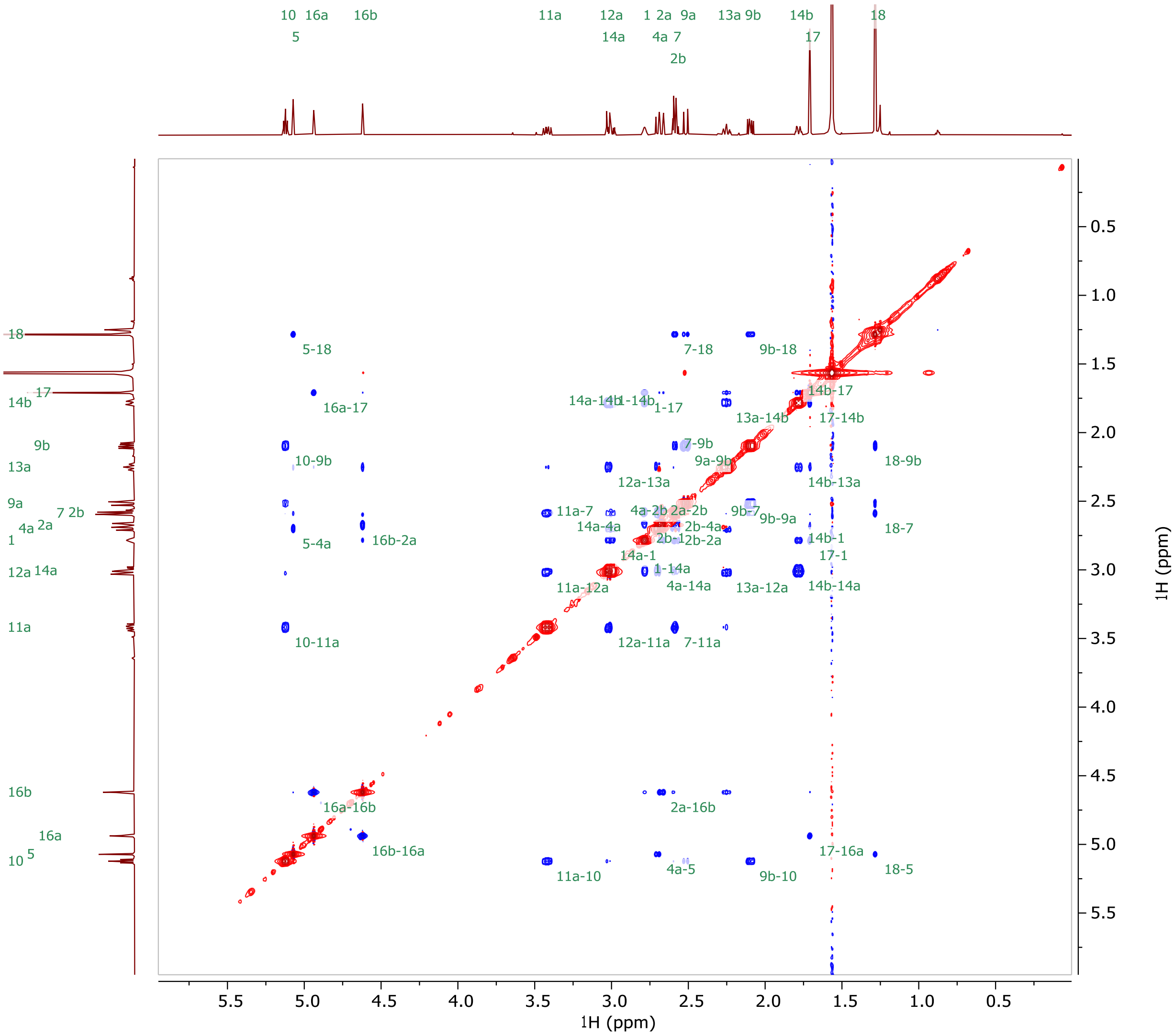

NOESY — LID-LA-405-04 (12629) — CDCl3; 298.0 K; 1.1 mg; 5 mm — 1H (noesygpphpp) — AV600neo (cryoBBO) — 19.06.24 04:57

^{1}H NMR of Horiolide (34) (600 MHz, $CDCl_3$)

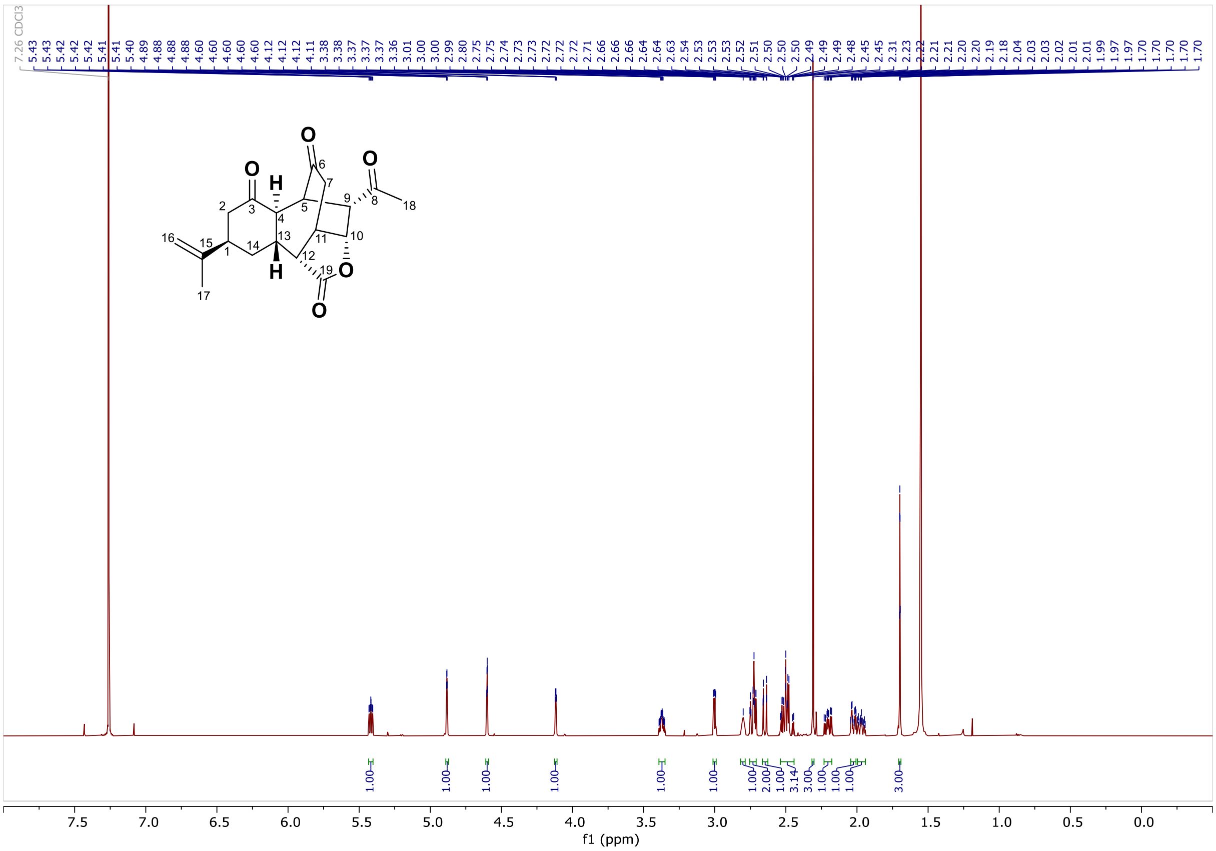

^{13}C NMR of Horiolide (34) (150 MHz, $CDCl_3$)

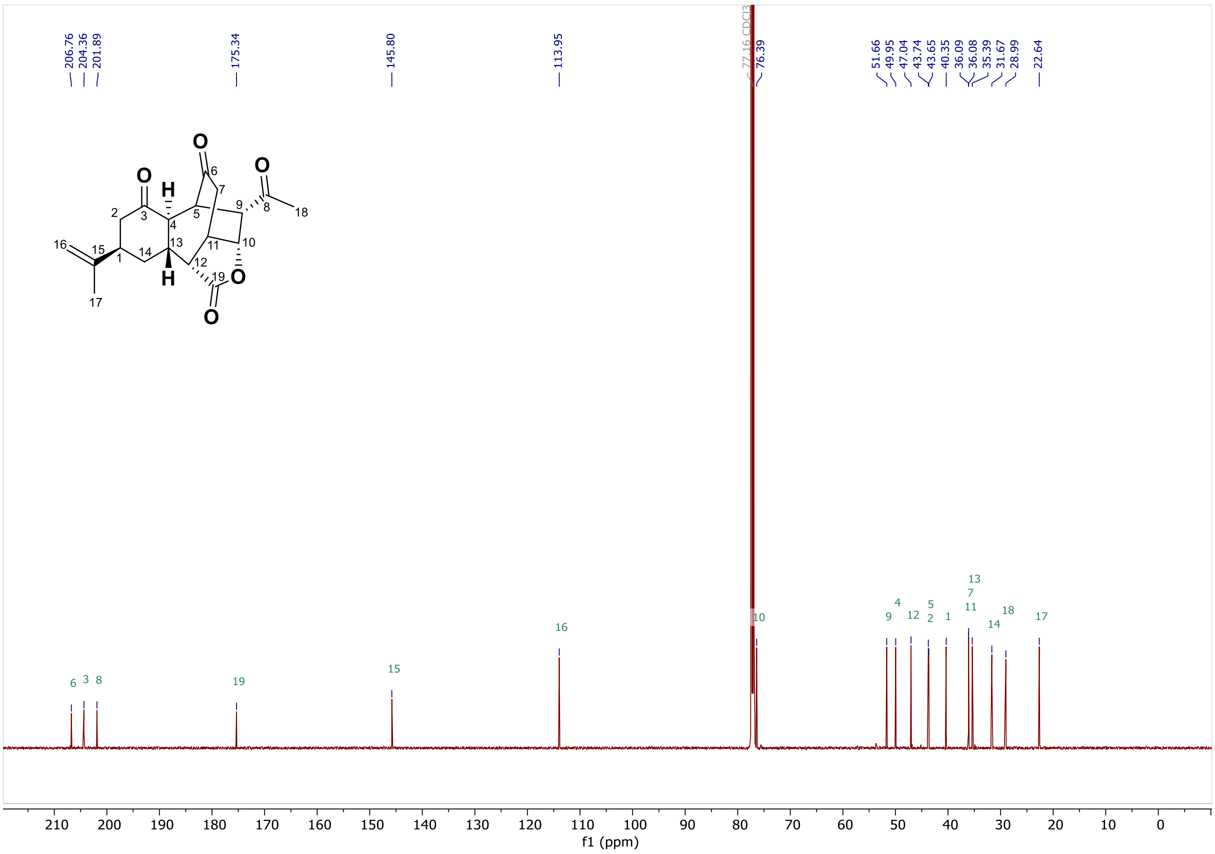

^{1}H COSY spectrum of Horiolide (34) (600 MHz, $CDCl_3$)

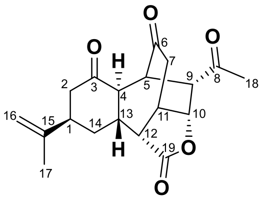

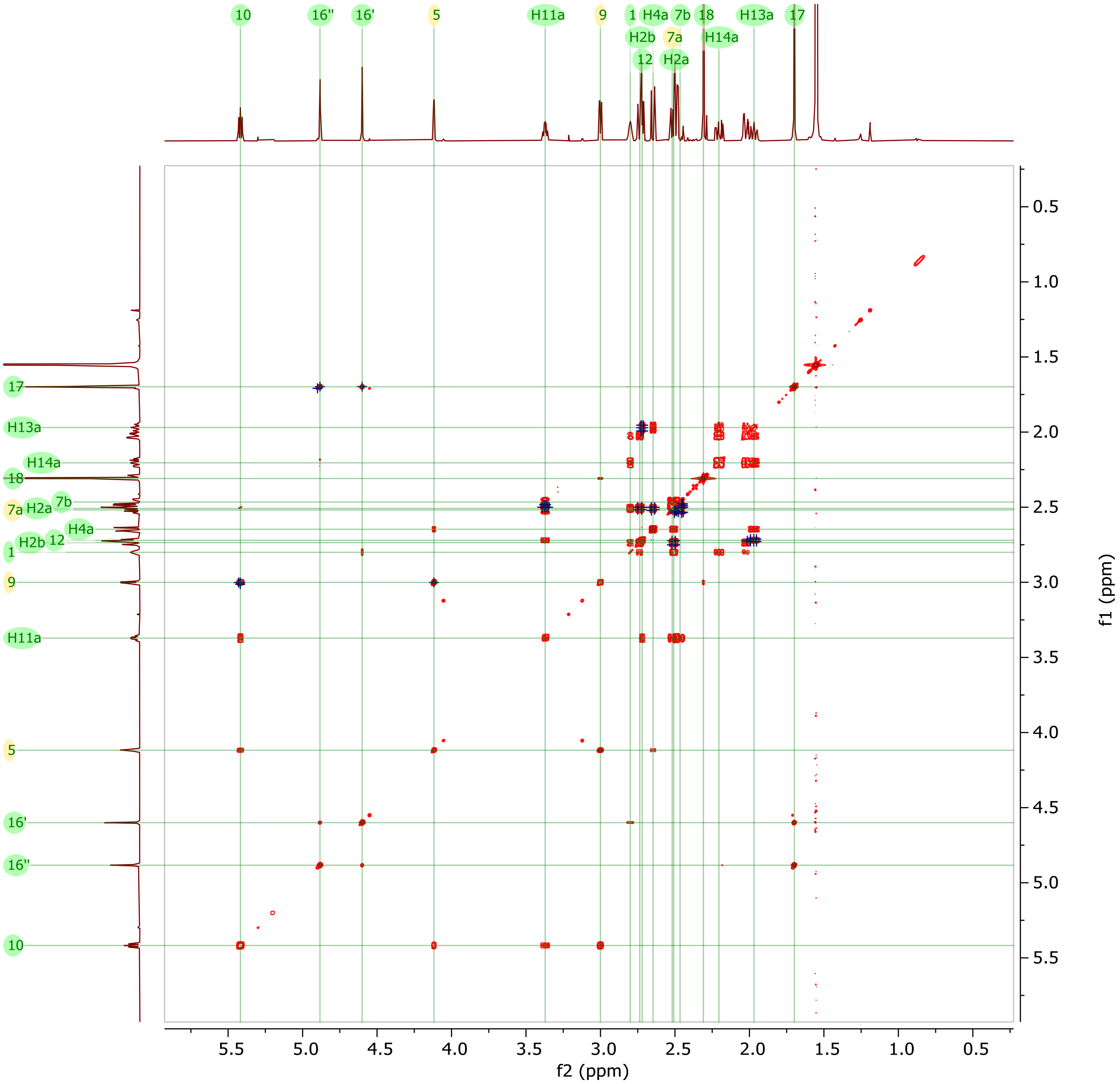

HSQC spectrum of Horiolide (34)

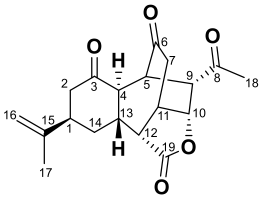

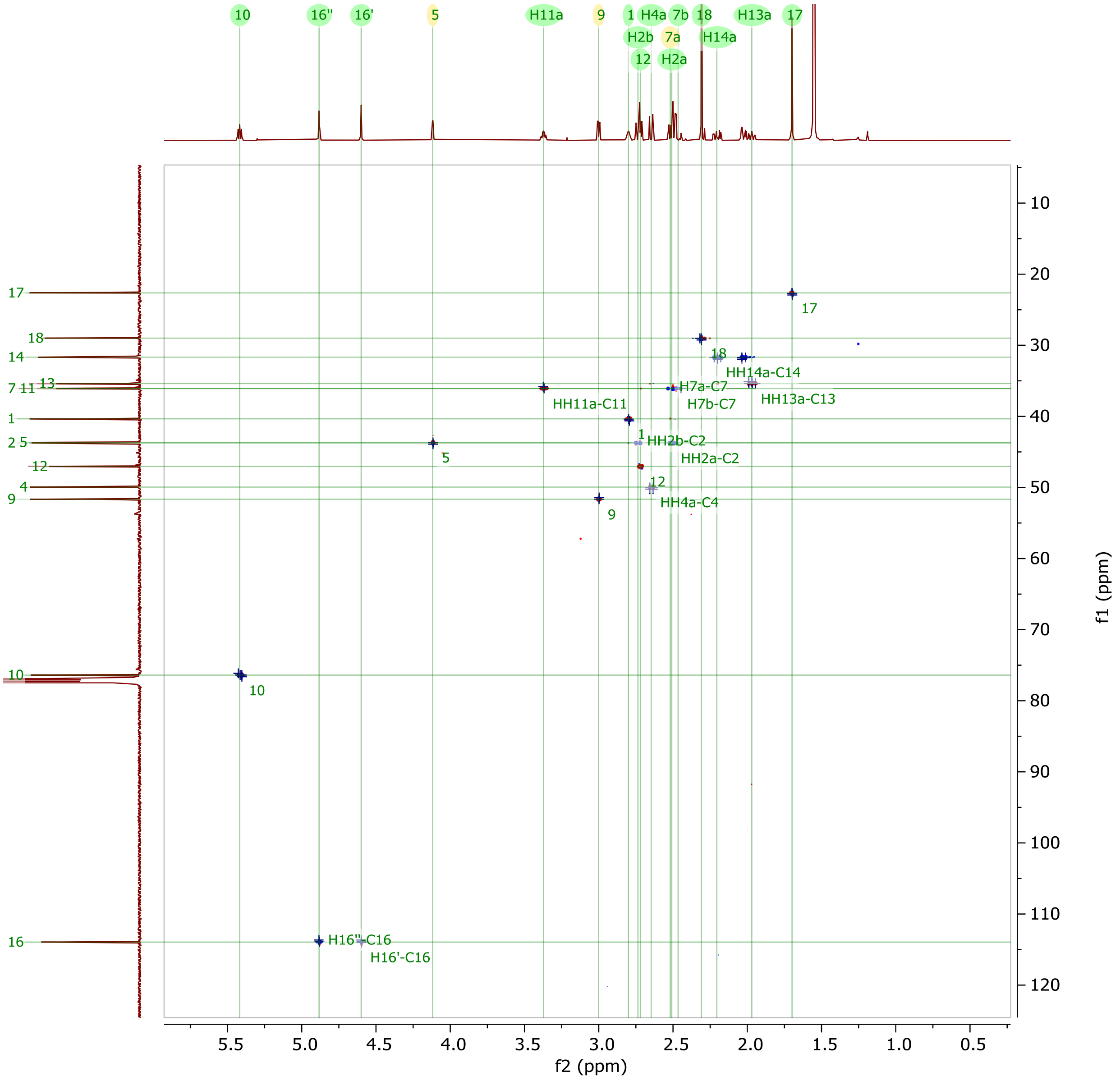

HMBC spectrum of Horiolide (34)

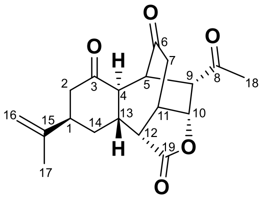

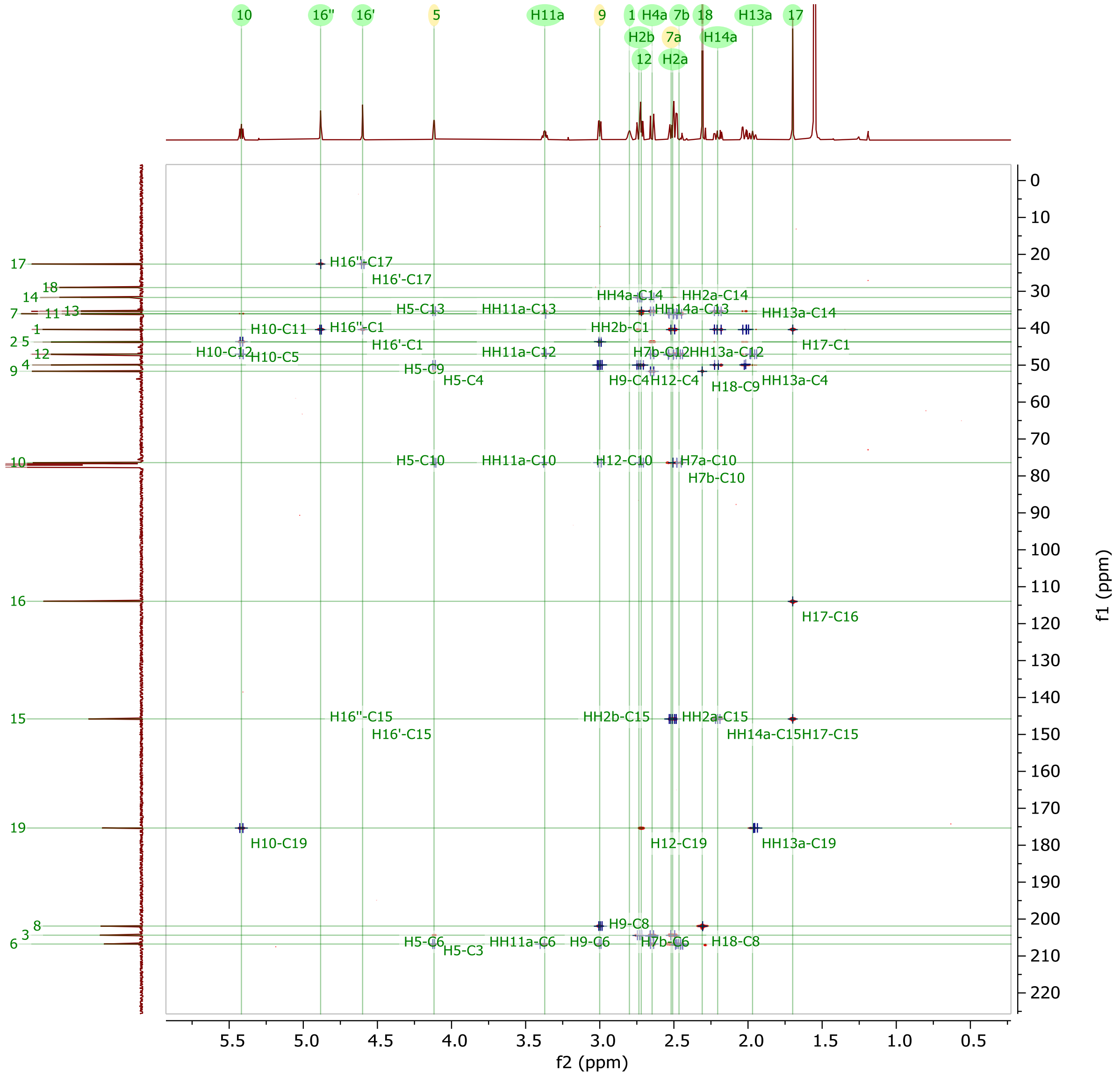

NOESY spectrum of Horiolide (34)

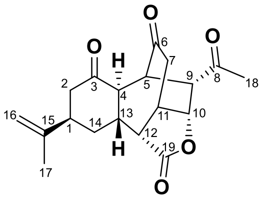

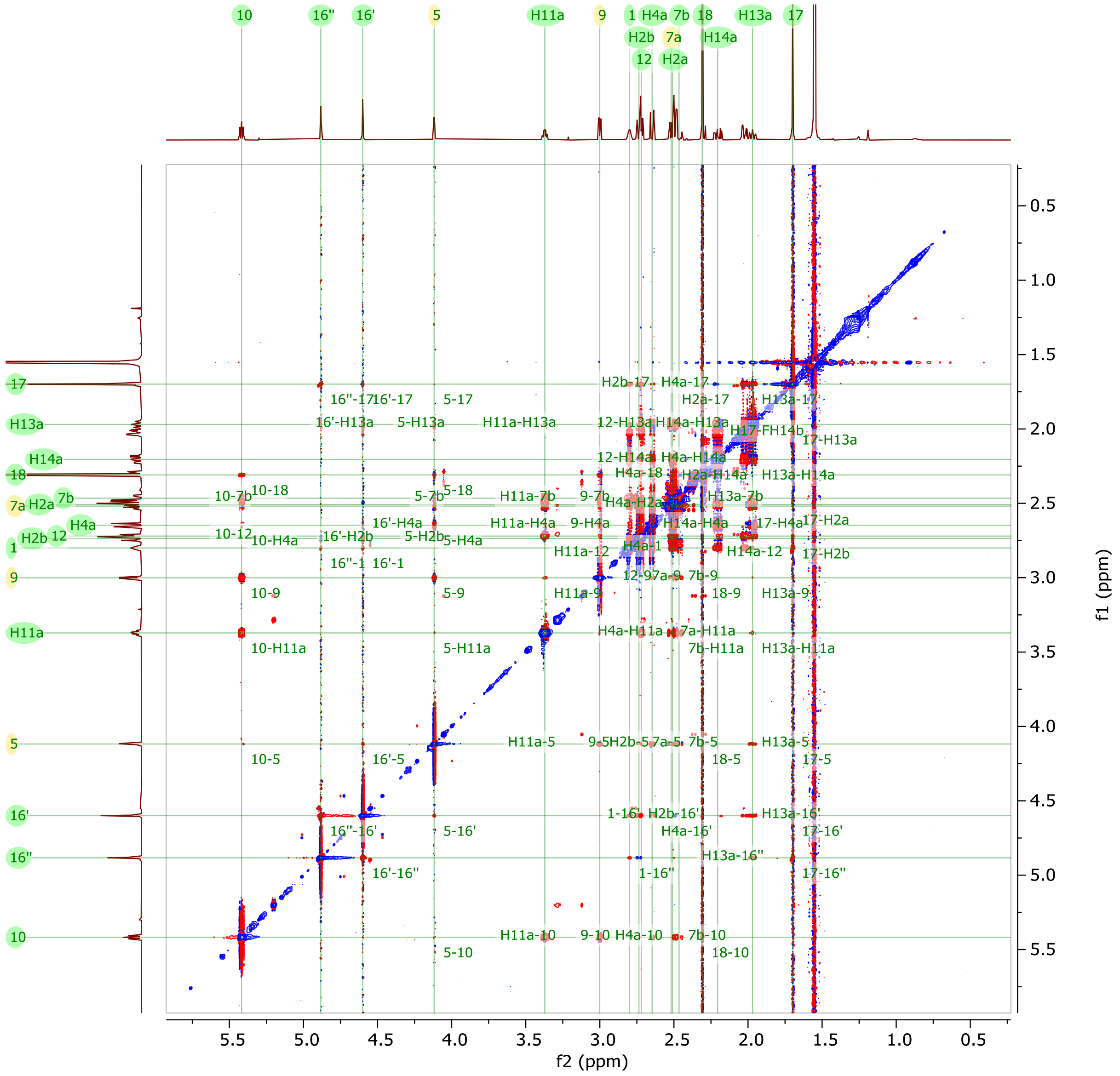

References

1. Sheu, J. H.; Ahmed, A. F.; Shiue, R.-T.; Dai, C.-F.; Kuo, Y.-H., Scabrolides A–D, Four New Norditerpenoids Isolated from the Soft Coral Sinularia scabra. *J. Nat. Prod.* **2002,** *65* (12), 1904-1908.
2. Meng, Z.; Fürstner, A., Total Syntheses of Scabrolide A and Nominal Scabrolide B. *J. Am. Chem. Soc.* **2022,** *144* (4), 1528-1533.
3. Chhetri, B. K.; Lavoie, S.; Sweeney-Jones, A. M.; Kubanek, J., Recent trends in the structural revision of natural products. *Nat. Prod. Rep.* **2018,** *35* (6), 514-531.
4. Maier, M. E., Structural revisions of natural products by total synthesis. *Nat. Prod. Rep.* **2009,** *26* (9), 1105-1124.
5. Nicolaou, K. C.; Snyder, S. A., Chasing molecules that were never there: misassigned natural products and the role of chemical synthesis in modern structure elucidation. *Angew. Chem. Int. Ed. Engl.* **2005,** *44* (7), 1012-1044.
6. Grimblat, N.; Zanardi, M. M.; Sarotti, A. M., Beyond DP4: an Improved Probability for the Stereochemical Assignment of Isomeric Compounds using Quantum Chemical Calculations of NMR Shifts. *J. Org. Chem.* **2015,** *80* (24), 12526-12534.
7. Smith, S. G.; Goodman, J. M., Assigning Stereochemistry to Single Diastereoisomers by GIAO NMR Calculation: The DP4 Probability. *J. Am. Chem. Soc.* **2010,** *132* (37), 12946-12959.
8. Du, Y.; Yao, L.; Li, X.; Guo, Y., Yonarolide A, an unprecedented furanobutenolide-containing norcembranoid derivative formed by photoinduced intramolecular [2+2] cycloaddition. *Chin. Chem. Lett.* **2023,** *34* (2), 107512.
9. Schrödinger Release 2023-3: Maestro, S., LLC, New York, NY., 2023.
10. Mohamadi, F.; Richards, N. G. J.; Guida, W. C.; Liskamp, R.; Lipton, M.; Caufield, C.; Chang, G.; Hendrickson, T.; Still, W. C., Macromodel—an integrated software system for modeling organic and bioorganic molecules using molecular mechanics. *J. Comput. Chem.* **1990,** *11* (4), 440-467.
11. Watts, K. S.; Dalal, P.; Tebben, A. J.; Cheney, D. L.; Shelley, J. C., Macrocycle Conformational Sampling with MacroModel. *J. Chem. Inform. Modeling* **2014,** *54* (10), 2680-2696.
12. MacoModel in Vol. Schrödinger, LLC, New York, NY. 2023.
13. Grimme, S.; Bannwarth, C.; Shushkov, P., A Robust and Accurate Tight-Binding Quantum Chemical Method for Structures, Vibrational Frequencies, and Noncovalent Interactions of Large Molecular Systems Parametrized for All spd-Block Elements (Z = 1–86). *J. Chem. Theory Comput.* **2017,** *13* (5), 1989-2009.
14. Bannwarth, C.; Ehlert, S.; Grimme, S., GFN2-xTB—An Accurate and Broadly Parametrized Self-Consistent Tight-Binding Quantum Chemical Method with Multipole Electrostatics and Density-Dependent Dispersion Contributions. *J. Chem. Theory Comput.* **2019,** *15* (3), 1652-1671.
15. Barone, V.; Cossi, M., Quantum Calculation of Molecular Energies and Energy Gradients in Solution by a Conductor Solvent Model. *J. Phys. Chem. A* **1998,** *102* (11), 1995-2001.
16. Grimme, S.; Bohle, F.; Hansen, A.; Pracht, P.; Spicher, S.; Stahn, M., Efficient Quantum Chemical Calculation of Structure Ensembles and Free Energies for Nonrigid Molecules. *J. Phys. Chem. A* **2021,** *125* (19), 4039-4054.
17. Neese, F., Software update: The ORCA program system—Version 5.0. *WIREs Computational Molecular Science* **2022,** *12* (5), e1606.

18. Neese, F.; Wennmohs, F.; Becker, U.; Riplinger, C., The ORCA quantum chemistry program package. *J. Chem. Phys.* **2020,** *152* (22).

19. Grimme, S.; Bannwarth, C.; Shushkov, P., A Robust and Accurate Tight-Binding Quantum Chemical Method for Structures, Vibrational Frequencies, and Noncovalent Interactions of Large Molecular Systems Parametrized for All spd-Block Elements (Z = 1-86). *J Chem Theory Comput* **2017,** *13* (5), 1989-2009.

20. Zanardi, M. M.; Sarotti, A. M., Sensitivity Analysis of DP4+ with the Probability Distribution Terms: Development of a Universal and Customizable Method. *J. Org. Chem.* **2021,** *86* (12), 8544-8548.

21. Cheng, W.; Ji, M.; Li, X.; Ren, J.; Yin, F.; van Ofwegen, L.; Yu, S.; Chen, X.; Lin, W., Fragilolides A-Q, norditerpenoid and briarane diterpenoids from the gorgonian coral Junceella fragilis. *Tetrahedron* **2017,** *73* (17), 2518-2528.

22. Yun, S. Y.; Zheng, J.-C.; Lee, D., Stereoelectronic Effect for the Selectivity in C−H Insertion of Alkylidene Carbenes and Its Application to the Synthesis of Platensimycin. *J. Am. Chem. Soc.* **2009,** *131* (24), 8413-8415.

23. Fürstner, A.; Langemann, K., Total Syntheses of (+)-Ricinelaidic Acid Lactone and of (−)-Gloeosporone Based on Transition-Metal-Catalyzed C−C Bond Formations. *J. Am. Chem. Soc.* **1997,** *119* (39), 9130-9136.

24. Serrano, R.; Boyko, Y. D.; Hernandez, L. W.; Lotuzas, A.; Sarlah, D., Total Syntheses of Scabrolide A and Yonarolide. *J. Am. Chem. Soc.* **2023,** *145* (16), 8805-8809.

25. Robert, T.; Velder, J.; Schmalz, H.-G., Enantioselective Cu-Catalyzed 1,4-Addition of Grignard Reagents to Cyclohexenone Using Taddol-Derived Phosphine–Phosphite Ligands and 2-Methyl-THF as a Solvent. *Angew. Chem. Int. Ed.* **2008,** *47* (40), 7718-7721.

26. Gamba, D.; Pisoni, D. S.; Costa, J. S.; Petzhold, C. L.; Borges, A. C. A.; Ceschi, M. A., Enantioselective Synthesis of (R)-Isocarvone from (S)-Perillaldehyde *J. Braz. Chem. Soc*. **2008**, *19*, 1270

27. Liu, J.; Tang, Q.; Huang, J.; Li, T.; Ouyang, H.; Lin, W.-h.; Yan, X.-j.; Yan, X.; He, S., Sinuscalide A: An Antiviral Norcembranoid with an 8/8-Fused Carbon Scaffold from the South China Sea Soft Coral Sinularia scabra. *J. Org. Chem.* **2022,** *87* (15), 9806-9814.

28. The optical rotation for Sinuscalide C is reported in MeOH. We we not able to dissolve our sample in MeOH, even at higher dilution. We therefore report the optical rotation in $CHCl_3$.

29. Duh, C.-Y.; Wang, S.-K.; Chia, M.-C.; Chiang, M. Y., A novel cytotoxic norditerpenoid from the Formosan soft coral Sinularia inelegans. *Tetrahedron Lett.* **1999,** *40* (33), 6033-6035.

30. Radhika, P.; Subba Rao, P. V.; Anjaneyulu, V.; Asolkar, R. N.; Laatsch, H., Horiolide, a Novel Norditerpenoid from Indian Ocean Soft Coral of the Genus Sinularia. *J. Nat. Prod.* **2002,** *65* (5), 737-739.

31. Tuccinardi, J. P.; Wood, J. L., Total Syntheses of (+)-Ineleganolide and (−)-Sinulochmodin C. *J. Am. Chem. Soc.* **2022,** *144* (44), 20539-20547.

32. Gross, B. M.; Han, S.-J.; Virgil, S. C.; Stoltz, B. M., A Convergent Total Synthesis of (+)-Ineleganolide. *J. Am. Chem. Soc.* **2023,** *145* (14), 7763-7767.
